# Supplementary figures and images for: Role of vitamins in the pathogenesis and treatment of restless leg syndrome: A systematic review and meta-analysis
Source: PLoS One. 2025 Mar 10;20(3):e0313571. doi: 10.1371/journal.pone.0313571 (PMC11892881; doi:10.1371/journal.pone.0313571)

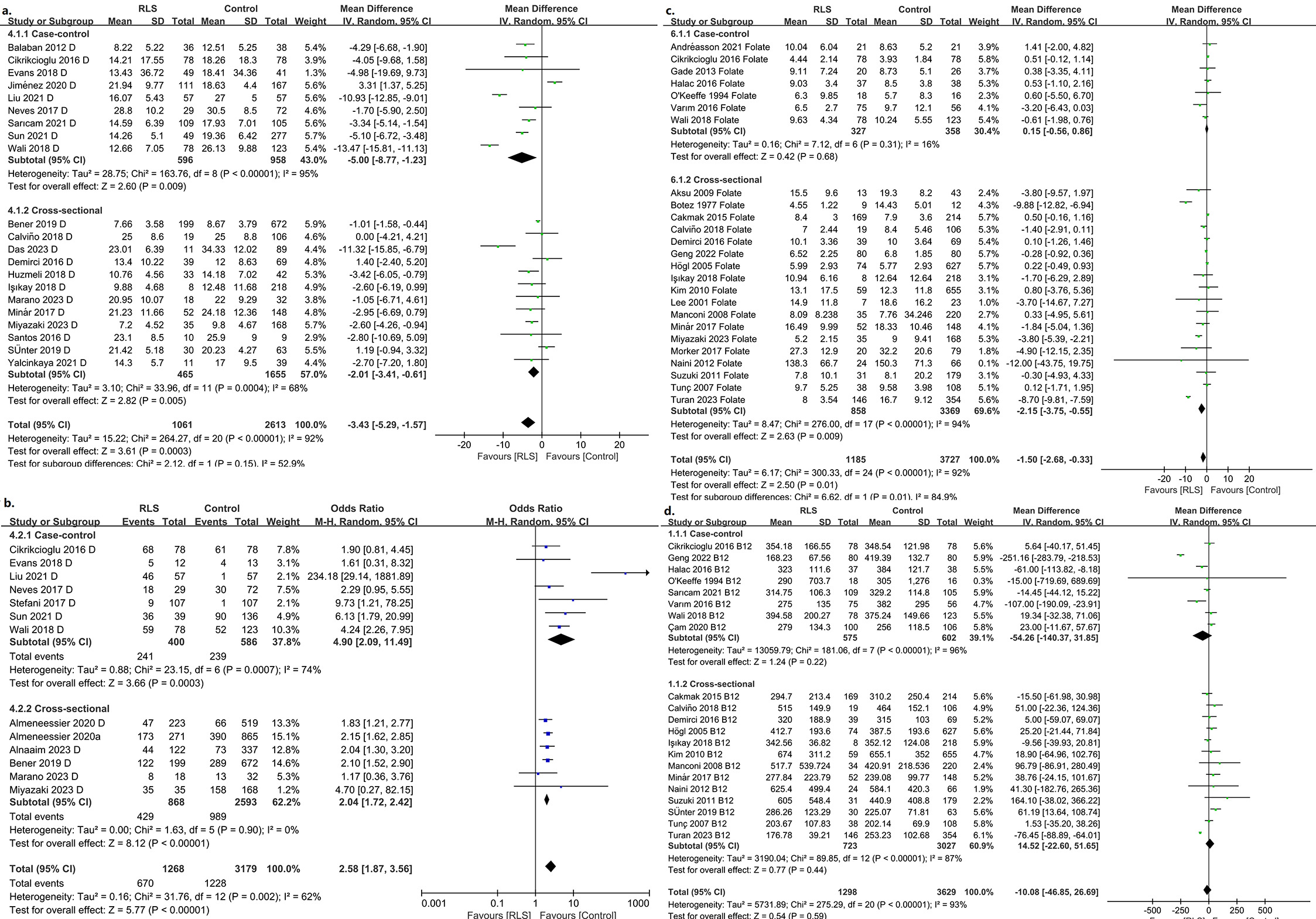

Supplement: Supplement Fig 1 — b. Rate of vitamin D deficiency/insufficient. c. Folate levels. d. Vitamin B12. (TIF) [file pone.0313571.s001.tif]

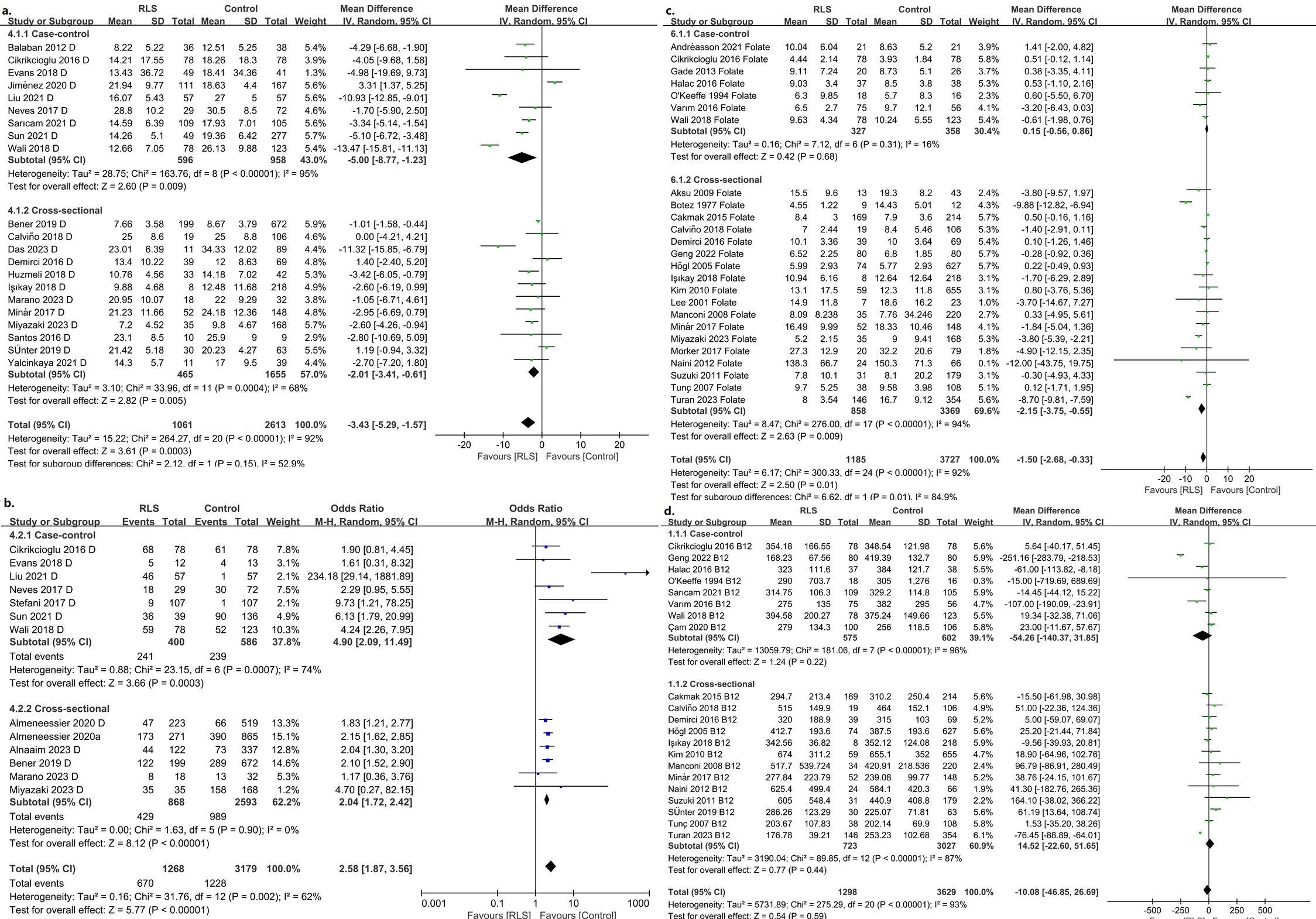

Supplement: Supplement Fig 2 — Rate of vitamin D deficiency/insufficient. b. Folate levels. (TIF) [file pone.0313571.s002.tif]

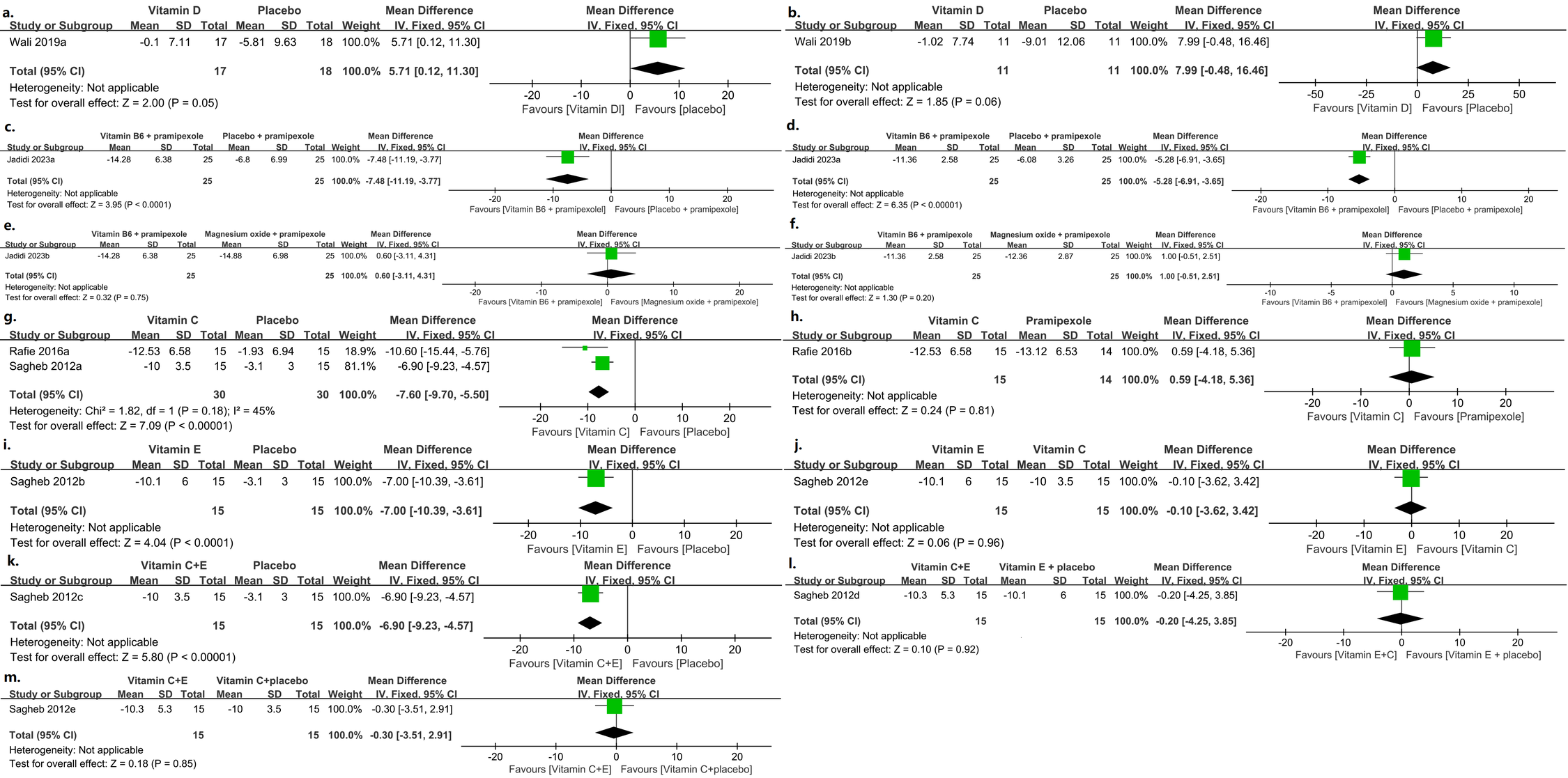

Supplement: Supplement Fig 3 — Vitamin D did not reduce patients’ RLS severity score compared to placebo, regardless of vitamin D deficiency; c-f. Vitamin B6 significantly reduced RLS patients’ IRLS scores and PSQI scores compared to placebo, and was comparable to magnesium oxide; g-h. Oral vitamin C could significantly reduce IRLS scores in hemodialysis associated RLS patients compared to placebo, and was comparable to pramipexole; i-j. Oral vitamin E could significantly reduce IRLS scores in hemodialysis associated RLS patients compared to placebo, and was equivalent to vitamin C; k-m. Oral vitamin C + E significantly reduced IRLS scores in hemodialysis associated patient compared with double placebo, but was not better than vitamin E + placebo or vitamin C + placebo. (TIF) [file pone.0313571.s003.tif]

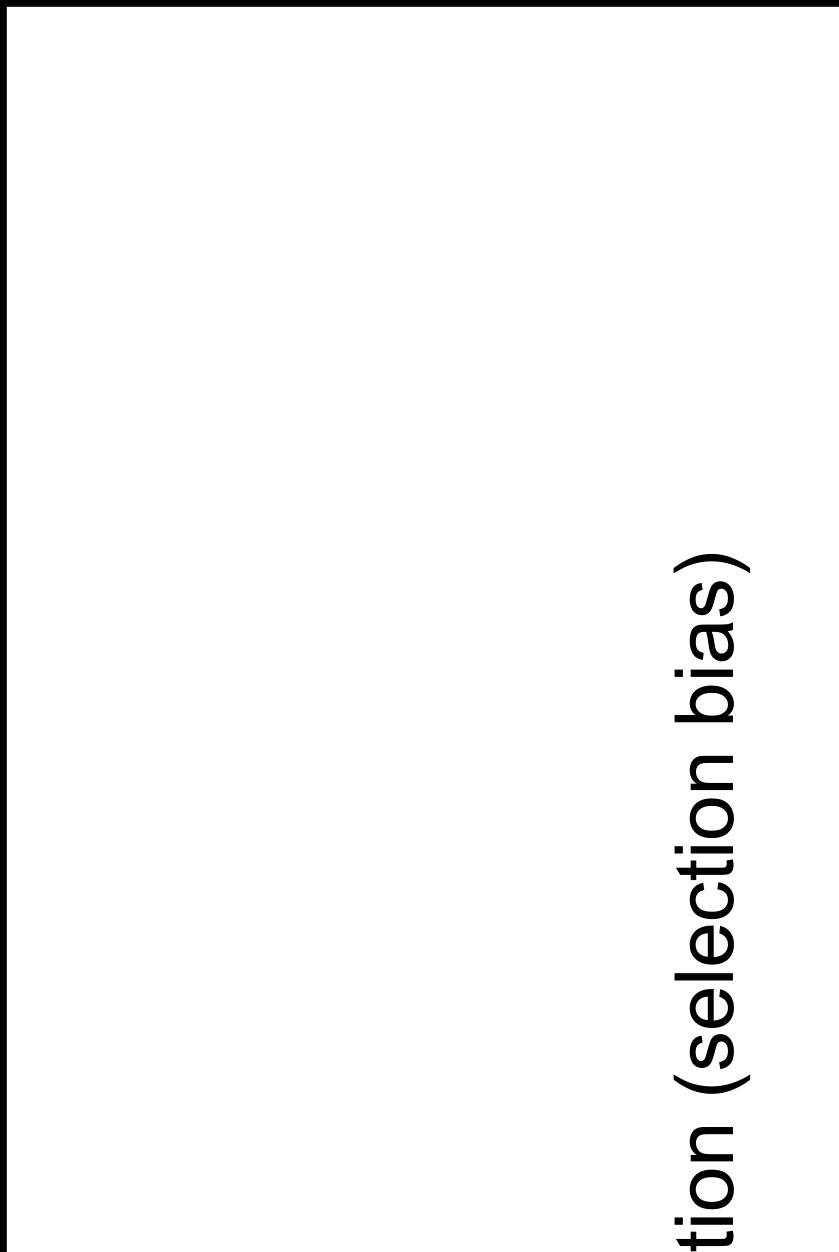

Supplement: Supplement Fig 4a — b.‘Risk of bias’ graph: review authors’ judgements about each risk of bias item presented as percentages across all included trials, with the majority of treatment trials having a low risk of bias. (TIF) [file pone.0313571.s004.tif]
