# Supplementary material for: Role of vitamins in the pathogenesis and treatment of restless leg syndrome: A systematic review and meta-analysis
Source: PLoS One. 2025 Mar 10;20(3):e0313571. doi: 10.1371/journal.pone.0313571 (PMC11892881; doi:10.1371/journal.pone.0313571)
Supplement: Supplemental Table 1 — (DOCX) [file pone.0313571.s005.docx]

Supplemental Table 1. Results of the systematic search strategy.*

Database No. of Citations

PubMed 147

Cochrane 397

Web of Science 527

Embase 675

Total 1746

*Explicit search strategy (pubmed):

**#1**  **‘restless legs syndrome*’[Title/Abstract] OR ‘RLS’[Title/Abstract] OR ‘Willis Ekbom’[Title/Abstract] OR ‘Ekbom syndrome’[Title/Abstract] OR ‘sleep leg movement*’[Title/Abstract] OR ‘sleep-related movement disorder*’[Title/Abstract]**

**#2** **‘Restless Legs Syndrome’ [MeSH Terms]**

**#3** #1 or #2

**#4** **vitamin*[Title/Abstract] OR ‘folic acid’[Title/Abstract] OR folate[Title/Abstract] OR methyltetrahydrofolic[Title/Abstract] OR methyltetrahydrofolate[Title/Abstract] OR ‘methyl tetrahydrofolic’[Title/Abstract] OR ‘methyl tetrahydrofolate’[Title/Abstract] OR mthf[Title/Abstract] OR thiamin*[Title/Abstract] OR aneurin[Title/Abstract] OR riboflavin[Title/Abstract] OR pyridoxin[Title/Abstract] OR cobalamin[Title/Abstract] OR cyanocobalamin[Title/Abstract] OR cobamide[Title/Abstract] OR hydroxocobalamin[Title/Abstract] OR cholecalciferol[Title/Abstract] OR dihydroxycholecalciferol[Title/Abstract] OR ergocalciferol[Title/Abstract] OR tocopherol[Title/Abstract] OR tocotrienol[Title/Abstract] OR ‘ascorbic acid’[Title/Abstract] OR ascorbate[Title/Abstract] OR dehydroascorbate[Title/Abstract] OR ‘beta carotene’[Title/Abstract] OR betacarotene[Title/Abstract] OR retinol[Title/Abstract] OR niacin[Title/Abstract] OR ‘nicotinic acid’[Title/Abstract] OR multivitamin*[Title/Abstract] OR multi-vitamin*[Title/Abstract]**

**#5** #3 and #4

*Explicit search strategy (Cochrane):

#**1 (‘restless legs syndrome*’ or ‘RLS’ or ‘Willis Ekbom’ or ‘Ekbom syndrome’ or ‘sleep leg movement*’ or ‘sleep-related movement disorder*’ ):ti,ab,kw**

**#2 (vitamin* or ‘folic acid’ or folate or methyltetrahydrofolic or methyltetrahydrofolate or ‘methyl tetrahydrofolic’ or ‘methyl tetrahydrofolate’ or mthf or thiamin* or aneurin or riboflavin or pyridoxin or cobalamin or cyanocobalamin or cobamide or hydroxocobalamin or cholecalciferol or dihydroxycholecalciferol or ergocalciferol or tocopherol or tocotrienol or ‘ascorbic acid’ or ascorbate or dehydroascorbate or ‘beta carotene’ or betacarotene or retinol or niacin or ‘nicotinic acid’ or multivitamin* or multi-vitamin*):ti,ab,kw**

**#3 #1 and #2**

***Explicit search strategy (Web of science):**

**#1 TS=(‘restless legs syndrome*’ or ‘RLS’ or ‘Willis Ekbom’ or ‘Ekbom syndrome’ or ‘sleep leg movement*’ or ‘sleep-related movement disorder*’)**

**#2 TS=(vitamin* or ‘folic acid’ or folate or methyltetrahydrofolic or methyltetrahydrofolate or ‘methyl tetrahydrofolic’ or ‘methyl tetrahydrofolate’ or mthf or thiamin* or aneurin or riboflavin or pyridoxin or cobalamin or cyanocobalamin or cobamide or hydroxocobalamin or cholecalciferol or dihydroxycholecalciferol or ergocalciferol or tocopherol or tocotrienol or ‘ascorbic acid’ or ascorbate or dehydroascorbate or ‘beta carotene’ or betacarotene or retinol or niacin or ‘nicotinic acid’ or multivitamin* or multi-vitamin*)**

**#3 #1 and #2**

***Explicit search strategy (Embase):**

**#1 'restless legs syndrome*' OR 'rls' OR 'willis ekbom' OR 'ekbom syndrome'/exp OR 'ekbom syndrome' OR 'sleep leg movement*' OR 'sleep-related movement disorder*':ab,ti**

**#2 vitamin* OR 'folic acid' OR folate OR methyltetrahydrofolic OR methyltetrahydrofolate OR 'methyl tetrahydrofolic' OR 'methyl tetrahydrofolate' OR mthf OR thiamin* OR aneurin OR riboflavin OR pyridoxin OR cobalamin OR cyan ocobalamin OR cobamide OR hydroxocobalamin OR cholecalciferol OR dihydroxycholecalciferol OR ergocalciferol OR tocopherol OR tocotrienol OR 'ascorbic acid' OR ascorbate OR dehydroascorbate OR 'beta carotene' OR betacarotene OR retinol OR niacin OR 'nicotinic acid' OR multivitamin* OR 'multi  vitamin*':ab,ti**

**#3 #1 and #2**
