# Supplementary material for: Role of vitamins in the pathogenesis and treatment of restless leg syndrome: A systematic review and meta-analysis
Source: PLoS One. 2025 Mar 10;20(3):e0313571. doi: 10.1371/journal.pone.0313571 (PMC11892881; doi:10.1371/journal.pone.0313571)
Supplement: Supplemental Table 2 — (DOCX) [file pone.0313571.s006.docx]

| **Supplemental Table 2.** Results of the Newcastle-Ottawa scale assessment. | | | | | | | | | | |
| --- | --- | --- | --- | --- | --- | --- | --- | --- | --- | --- |
| Study | Selection | | | | Comparability | | Exposure | | | Scores |
|  | Adequate definition of cases | Representativeness of the cases | Selection of controls | Definition of controls | Control for important factor | Control for other important factor | Ascertainment of method | Same method of ascertainment for cases | Non-response rate |  |
| Botez 1977[43] | unclear | unclear | unclear | * | * | unclear | unclear | * | * | unclear |
| O'Keeffe 1994[49] |  |  |  | * | * | * |  | * |  | 4 |
| Lee 2001[44] |  | * | * | * | * | * |  | * |  | 6 |
| Manconi 2004[61] | * | * |  | * | * |  |  | * | * | 6 |
| Högl 2005[63] | * | * | * | * | * | * |  | * |  | 7 |
| Kemlink 2007[50] | * |  |  | * | * |  |  | * | * | 5 |
| Tunç 2007[64] |  | * | * | * | * | * | * | * | * | 8 |
| Manconi 2008[51] | * | * |  | * | * |  |  | ***** | ***** | 6 |
| Aksu 2009[52] | unclear | unclear |  | * | unclear | unclear | unclear | unclear | unclear | unclear |
| Kim 2010[65] | * | * | * | * | * | * |  | * | * | 8 |
| Suzuki 2011[62] |  | * |  | * | * | * |  | * |  | 5 |
| Balaban 2012[14] |  | * | * | * | * | * |  | * |  | 6 |
| Civi 2012[59] |  | * | * | * | * |  |  | * | * | 6 |
| Naini 2012[53] |  | * |  | * | * |  |  | * | * | 5 |
| Gade 2013[54] |  |  |  | * | * | * |  | * | * | 5 |
| Oran 2014[21] | * | * |  | * | * | * |  | * | * | 7 |
| Cakmak 2015[55] | * | * | * | * | * | * |  | * | * | 8 |
| Çakır 2015[20] |  | * |  | * | * | * |  | * | * | 6 |
| Cikrikcioglu 2016 [25] | * | * | * | * | * | * |  | * | * | 8 |
| Demirci 2016[27] |  | * |  | * | * | * |  | * | * | 6 |
| Halac 2016[56] |  |  | * | * | * | * |  | * | * | 6 |
| Helou 2016[67] | unclear | unclear |  | * | * | * | unclear | unclear | unclear | unclear |
| Santos 2016[30] |  | * |  | * | * | * |  | * |  | 5 |
| Stefani 2016[41] | unclear | unclear | unclear | unclear | * | unclear | unclear | unclear | unclear | unclear |
| Varım 2016[57] |  |  | * | * | * | * |  | * | * | 6 |
| Minár 2017[30] |  | * |  | * | * | * | * | * | * | 7 |
| Morker 2017[47] |  | * | * | * | * | * |  | * |  | 6 |
| Atar 2017 [23] | unclear | unclear | unclear | unclear | unclear | unclear | unclear | unclear | unclear | unclear |
| Neves 2017[13] | * |  |  | * | * | * | * | * | * | 7 |
| Stefani 2017[16] | unclear | unclear | unclear | unclear | * | unclear | unclear | unclear | unclear | unclear |
| Becker 2018[58] | * | * |  | * |  |  |  | * | * | 5 |
| Calviño 2018[31] | * | * |  | * | * | * |  | * | * | 7 |
| Evans 2018[36] | * |  | * | * |  |  |  | * | * | 5 |
| Huzmeli 2018[26] |  | * |  | * | * | * |  | * | * | 6 |
| Işıkay 2018[28] |  | * |  | * | * | * |  | * | * | 6 |
| Wali 2018[12] | * | * | * | * | * | * |  | * | * | 8 |
| Atalar 2019[22] |  |  |  | * | * | * |  | * | * | 5 |
| Aynacı 2019[46] |  |  | * | * | * | * |  | * | * | 6 |
| Bener 2019[29] |  | * |  | * |  |  |  | * | * | 4 |
| SÜnter 2019[42] |  |  |  | * |  | * |  | * | * | 4 |
| Tutuncu 2020[24] | * |  |  | * | * | * |  | * | * | 6 |
| Almeneessier 2020[38] |  | * | * | * |  |  |  | * | * | 5 |
| Almeneessier 2020a [37] |  | * | * | * |  |  |  | * | * | 5 |
| Çam 2020[66] | * |  |  | * | * | * |  | * | * | 6 |
| Jiménez 2020[15] | * |  |  | * | * |  |  | * | * | 5 |
| Sağlam 2020[18] |  |  | * | * | * | * |  | * | * | 6 |
| Andréasson2021[60] |  | * | * |  | * | * |  | * | * | 6 |
| Liu 2021[11] | * |  | * | * | * |  |  | * | * | 6 |
| Sarıcam 2021[39] |  |  | * | * | * |  |  | * | * | 5 |
| Sun 2021[17] |  |  | * | * | * | * |  | * | * | 6 |
| Yalcinkaya 2021[40] |  | * |  | * | * | * |  | * |  | 5 |
| Geng 2022 [45] | * |  | * | * | * | * |  | * | * | 7 |
| Alnaaim 2023[35] |  |  | * | * | * | * |  | * | * | 6 |
| Marano 2023[33] |  | * |  | * | * | * |  | * | * | 6 |
| Miyazaki 2023[18] |  | * | * | * | * | * | * | * | * | 8 |
| Das 2023[34] |  | * |  | * | * | * |  | * | * | 6 |
| Turan 2023[48] | * | * | * | * |  |  |  | * | * | 6 |
