# Supplementary material for: Role of vitamins in the pathogenesis and treatment of restless leg syndrome: A systematic review and meta-analysis
Source: PLoS One. 2025 Mar 10;20(3):e0313571. doi: 10.1371/journal.pone.0313571 (PMC11892881; doi:10.1371/journal.pone.0313571)
Supplement: Supplemental Table 4 — (DOCX) [file pone.0313571.s008.docx]

| **Full text** | **Included/Excluded** | **According to title/abstract or full text** | **Reason** |
| --- | --- | --- | --- |
| 1] 5th Biennial Congress of the European Society of Endocrine Surgeons, ESES 2012, Langenbeck's Archives of Surgery 397(5) (2012). | Excluded | Title and abstract | Irrelevant |
| 2] 5th International Congress on Psychopharmacology & International Symposium on Child and Adolescent Psychopharmacology, Bulletin of Clinical Psychopharmacology 23 (2013) S1. | Excluded | Title and abstract | Irrelevant |
| 3] M. Hawkins, A 9-year-old female with iron deficiency has severe periodic limb movements while taking mirtazapine for insomnia, Journal of Clinical Sleep Medicine 19(7) (2023) 1369-1373. | Excluded | Title and abstract | Irrelevant |
| 4] eudract_number, A 12 week, multicentre, study investigating the efficacy of ORM-12741 on agitation/aggression symptoms in patients with Alzheimer's Disease, https://www.clinicaltrialsregister.eu/ctr-search/search?query=eudract_number:2014-000217-30 (2015). | Excluded | Title and abstract | Irrelevant |
| 5] L. Webster, R. Tummala, U. Diva, J. Lappalainen, A 12-week extension study to assess the safety and tolerability of naloxegol in patients with noncancer pain and opioid-induced constipation, Journal of opioid management 12(6) (2016) 405‐419. | Excluded | Title and abstract | Irrelevant |
| 6] A. Skalska, A. Galas, T. Grodzicki, 25-hydroxyvitamin D and physical and cognitive performance in older people with chronic conditions, Polskie Archiwum Medycyny Wewnetrznej-Polish Archives of Internal Medicine 122(4) (2012) 162-169. | Excluded | Title and abstract | Irrelevant |
| 7] Anonymous, 39th Annual Meeting of the Swiss-Society-of-Nephrology, Lausanne, SWITZERLAND, December 05 -07, 2007, Swiss medical weekly 137(Suppl. 161) (2007) S3-S24. | Excluded | Title and abstract | Irrelevant |
| 8] Anonymous, 82nd Congress of the Deutschen-Gesellschaft-fur-Neurologie, Nurnberg, GERMANY, September 23 -26, 2009, Aktuelle Neurologie 36(Suppl. 2) (2009) S47-S215. | Excluded | Title and abstract | Irrelevant |
| 9] S.L. Peng, 184. Denosumab protects against bone loss and maintains function in osteopenic patients with lumbar degenerative diseases after lumbar fusion surgery: a randomized controlled trial, Spine journal 23(9) (2023) S94. | Excluded | Title and abstract | Irrelevant |
| 10] Y.C. Chen, K.T. Huang, M.C. Su, P.Y. Hsu, C.H. Chin, I.C. Lin, C.W. Liou, T.Y. Wang, Y.Y. Lin, C.C. Hsiao, M.C. Lin, Aberrant DNA methylation levels of the formyl peptide receptor 1/2/3 genes are associated with obstructive sleep apnea and its clinical phenotypes, American Journal of Translational Research 12(6) (2020) 2521-2537. | Excluded | Title and abstract | Irrelevant |
| 11] S. Happe, J. Zeitlhofer, Abnormal cutaneous thermal thresholds in patients with restless legs syndrome, Journal of neurology 250(3) (2003) 362-365. | Excluded | Title and abstract | Irrelevant |
| 12] Calvio, Jesús, Cigarrán, Secundino, Gonzalez-Tabares L ,et al. Restless Legs Syndrome: An Unresolved Uremic Disorder after Renal Transplantation [J]. Nephron, 2018.DOI:10.1159/000486401. | Included | Full text | Relationship between vitamins and RLS |
| 13] L.E. Krahn, A. Petersen, B.W. Miller, M. Lizak, P. Lyng, Abnormal Pulse Oximetry Signal, Journal of Clinical Sleep Medicine 14(7) (2018) 1255-1256. | Excluded | Title and abstract | Irrelevant |
| 14] M.A. Carvalho, M.S. Schwartz, Abnormalities in motor conduction in vitamin B12 deficiency, Electromyography and clinical neurophysiology 36(5) (1996) 275-8. | Excluded | Title and abstract | Irrelevant |
| 15] J. Engert, D. Adelt, F. Bidlingmaier, Absorptiometry in the investigation of immobilisation osteoporosis in children and the use of drugs in its treatment, Monatsschrift fur Kinderheilkunde 125(10) (1977) 874‐880. | Excluded | Title and abstract | Irrelevant |
| 16] C.N. Homann, B. Homann, G. Ivanic, T. Urbanic-Purkart, Accidental Falls in Patients with Hyperkinetic Movement Disorders: A Systematic Review, Tremor and other hyperkinetic movements (New York, N.Y.) 12 (2022) 30-30. | Excluded | Title and abstract | Irrelevant |
| 17] S. Throm, Activities of the CHMP, Pharmazeutische Industrie 70(5) (2008) 613-619. | Excluded | Title and abstract | Irrelevant |
| 18] M.A. Wyon, R. Wolman, A.M. Nevill, R. Cloak, G.S. Metsios, D. Gould, A. Ingham, Y. Koutedakis, Acute Effects of Vitamin D3 Supplementation on Muscle Strength in Judoka Athletes: a Randomized Placebo-Controlled, Double-Blind Trial, Clinical journal of sport medicine 26(4) (2016) 279‐284. | Excluded | Title and abstract | Irrelevant |
| 19] A. Martínez Muelas, D. Paiva Pajares, M. López Isern, P. Ivanov, M. Sánchez Pérez, Acute Ekbom's syndrome in a patient with acute urethritis, European Psychiatry 65 (2022) S481. | Excluded | Title and abstract | Irrelevant |
| 20] K. Boskovic, B.P. Gava, M. Grajic, D. Madic, B. Obradovic, S.T. Todorovic, Adapted physical activity in the prevention and therapy of osteoporosis, Medicinski pregled 66(5-6) (2013) 221-4. | Excluded | Title and abstract | Irrelevant |
| 21] R. Von Berget, P. Ditzel, P. Jungmayr, B. Hellwig, Advanced trainig in view: Report of the 46th international training congress for practical and scientific pharmacy of the Federal Pharmacist Chamber in Merano, Deutsche Apotheker Zeitung 148(22) (2008) 44-68. | Excluded | Title and abstract | Irrelevant |
| 22] S. de Biase, G. Pellitteri, G.L. Gigli, M. Valente, Advancing synthetic therapies for the treatment of restless legs syndrome, Expert opinion on pharmacotherapy 20(16) (2019) 1971-1980. | Excluded | Title and abstract | Irrelevant |
| 23] J.L. Pedroso, P. Braga-Neto, A.C. Felício, O.G.P. Barsottini, L.B. Jardim, M.L. Saraiva-Pereira, Akathisia: An unusual movement disorder in Machado-Joseph disease, Parkinsonism and Related Disorders 17(9) (2011) 712-713. | Excluded | Title and abstract | Irrelevant |
| 24] Nct, Alimentation and Diabetes in Lanzarote - ADILAN: a Pilot Trial, https://clinicaltrials.gov/show/NCT01891955 (2013). | Excluded | Title and abstract | Irrelevant |
| 25] S. Hannam, R. Nixon, Allergic contact dermatitis to a N eupro patch used for restless legs syndrome, Australasian Journal of Dermatology 54 (2013) 6. | Excluded | Title and abstract | Irrelevant |
| 26] C. Chomienne, N. Balitrand, P. Ballerini, S. Castaigne, H. de Thé, L. Degos, All-trans retinoic acid modulates the retinoic acid receptor-alpha in promyelocytic cells, J Clin Invest 88(6) (1991) 2150-4. | Excluded | Title and abstract | Irrelevant |
| 27] C. Chomienne, N. Balitrand, P. Ballerini, S. Castaigne, H. de The, L. Degos, All-trans retinoic acid modulates the retinoic acid receptor-α in promyelocytic cells, Journal of Clinical Investigation 88(6) (1991) 2150-2154. | Excluded | Title and abstract | Irrelevant |
| 28] Nct, Almond Supplementation and Exercise Recovery, https://clinicaltrials.gov/show/NCT04958018 (2021). | Excluded | Title and abstract | Irrelevant |
| 29] P. Congiu, N. Ronzano, F. Cera, M. Tanca, C. Garau, A. Zuddas, A. Gagliano, M.M.F. Puligheddu, Altered sleep in a group of patients affected by Pediatric acute-onset neuropsychiatric syndrome (PANS), European journal of neurology 27 (2020) 517-517. | Excluded | Title and abstract | Irrelevant |
| 30] R.D. Chervin, F.B. Consens, E. Kutluay, Alternating leg muscle activation during sleep and arousals: A new sleep-related motor phenomenon?, Movement Disorders 18(5) (2003) 551-559. | Excluded | Title and abstract | Irrelevant |
| 31] D. Bega, R. Malkani, Alternative treatment of restless legs syndrome: an overview of the evidence for mind-body interventions, lifestyle interventions, and neutraceuticals, Sleep medicine 17 (2016) 99-105. | Excluded | Title and abstract | Irrelevant |
| 32] M.K. Samanta, B. Wilson, K. Santhi, K.P. Sampath Kumar, B. Suresh, Alzheimer disease and its management: A review, American journal of therapeutics 13(6) (2006) 516-526. | Excluded | Title and abstract | Irrelevant |
| 33] J.P. Brockes, Amphibian limb regeneration: Rebuilding a complex structure, Science (Washington D C) 276(5309) (1997) 81-87. | Excluded | Title and abstract | Irrelevant |
| 34] M. de Lourdes Samaniego-Vaesken, E. Alonso-Aperte, G. Varela-Moreiras, Analysis and evaluation of voluntary folic acid fortification of breakfast cereals in the Spanish market, Journal of Food Composition and Analysis 23(5) (2010) 419-423. | Excluded | Title and abstract | Irrelevant |
| 35] C.L. Howe, T.C. Keller, 3rd, M.S. Mooseker, R.H. Wasserman, Analysis of cytoskeletal proteins and Ca2+-dependent regulation of structure in intestinal brush borders from rachitic chicks, Proceedings of the National Academy of Sciences of the United States of America 79(4) (1982) 1134-8. | Excluded | Title and abstract | Irrelevant |
| 36] L.M. Panasenko, Z.V. Nefedova, T.V. Kartseva, K.A. Fadeeva, D.A. Oladele, N.V. Leonova, Analysis of risk factors for the formation of insufficiency and deficiency of vitamin D in children, Rossiyskiy Vestnik Perinatologii i Pediatrii 68(1) (2023) 91-96. | Excluded | Title and abstract | Irrelevant |
| 37] H.M. Liu, M. Chu, C.F. Liu, T. Zhang, P. Gu, Analysis of Serum Vitamin D Level and Related Factors in Patients With Restless Legs Syndrome, Front Neurol 12 (2021) 782565. | Included | Full text | Relationship between vitamins and RLS |
| 38] A. Arikanoglu, M. Demir, M.U. Aluclu, Analysis of YouTube as a source of information for restless leg syndrome, Arquivos de neuro-psiquiatria 78(10) (2020) 611-616. | Excluded | Title and abstract | Irrelevant |
| 39] D.J. Powell, M.O. Achebe, Anemia for the Primary Care Physician, Primary care 43(4) (2016) 527-+. | Excluded | Title and abstract | Irrelevant |
| 40] R. Ferrando, Animal feed additive regulation in the Common Market: rules, principles and ideas, Folia veterinaria Latina 7(3) (1977) 183-97. | Excluded | Title and abstract | Irrelevant |
| 41] R. Forster, M. Stewart, Anticoagulants (extended duration) for prevention of venous thromboembolism following total hip or knee replacement or hip fracture repair, Cochrane Database of Systematic Reviews (3) (2016). | Excluded | Title and abstract | Irrelevant |
| 42] R.L.G. Flumignan, V.T. Civile, J.D. Tinôco, P.I.F. Pascoal, L.L. Areias, C.F. Matar, B. Tendal, V.F.M. Trevisani, Á. Atallah, L.C.U. Nakano, Anticoagulants for people hospitalised with COVID‐19, Cochrane Database of Systematic Reviews (3) (2022). | Excluded | Title and abstract | Irrelevant |
| 43] M.R. Pranzatelli, Antidyskinetic drug therapy for pediatric movement disorders, Journal of Child Neurology 11(5) (1996) 355-369. | Excluded | Title and abstract | Irrelevant |
| 44] P. Howard, R. Twycross, J. Shuster, M. Mihalyo, J. Rémi, A. Wilcock, Anti-epileptic Drugs, Journal of Pain and Symptom Management 42(5) (2011) 788-804. | Excluded | Title and abstract | Irrelevant |
| 45] T. Roengrit, P. Wannanon, P. Prasertsri, Y. Kanpetta, B.O. Sripanidkulchai, J. Wattanathorn, N. Leelayuwat, Antioxidant effect of Phyllanthus amarus after moderate-intensity exercise in sedentary males: a randomized crossover (double-blind) study, Journal of physical therapy science 27(4) (2015) 1181‐1186. | Excluded | Title and abstract | Irrelevant |
| 46] E.R. Grela, M. Wesolowska-Trijanowska, A. Czech, Antioxidant status in the blood, liver, and muscle tissue of turkey hens receiving a diet with alfalfa protein concentrate, Poultry Science 102(4) (2023). | Excluded | Title and abstract | Irrelevant |
| 47] H. Rabl, G. Khoschsorur, W. Petek, Antioxidative vitamin treatment: effect on lipid peroxidation and limb swelling after revascularization operations, World journal of surgery 19(5) (1995) 738‐744. | Excluded | Title and abstract | Irrelevant |
| 48] L. Robertson, M.A. Ghouri, F. Kovacs, Antiplatelet and anticoagulant drugs for prevention of restenosis/reocclusion following peripheral endovascular treatment, Cochrane Database of Systematic Reviews (8) (2012). | Excluded | Title and abstract | Irrelevant |
| 49] H.R. Buller, G. Agnelli, R.D. Hull, T.M. Hyers, M.H. Prins, G.E. Raskob, Antithrombotic therapy for venous thromboembolic disease: the Seventh ACCP Conference on Antithrombotic and Thrombolytic Therapy, Chest 126(3 Suppl) (2004) 401S‐428S. | Excluded | Title and abstract | Irrelevant |
| 50] X. Su, Apparatus for two-dimensional separation of biomolecules e.g. proteins, comprises two separation modules for separating biomolecules in two different dimensions, and linear valve for controllably isolating the two separation modules, Intel Corp. | Excluded | Title and abstract | Irrelevant |
| 51] M. Burrows, C. Cole, J. Cronin, D. Gubernick, R. Hull, J. Menke, T. Narsana, J.F. Rytel, G. Skover, E. Lukenbach, M. Berross, J. Koning, J. Merk, T. Nasana, J.F. Lvturn, R.J. Hull, B. Mark, C. Curtis, G. David, H. Raymond, M. James, N. Tushar, F.R. John, S. Gregory, Apparatus to deliver mechanical energy for administering skin benefit agent to skin comprises skin-contactable element with the agent, motor, and transfer member to transfer mechanical energy for providing periodic motion to element surface, Johnson & Johnson Consumers Co Inc; Johnson & Johnson Consumer Co Inc; Johnson & Johnson Consumer Prod; Skover G; Cole C; Lukenbach E; Hull R J; Menke J. | Excluded | Title and abstract | Irrelevant |
| 52] G.F. Combs, N. Hassan, N. Dellagana, D. Staab, P. Fischer, C. Hunt, J. Watts, Apparent efficacy of food-based calcium supplementation in preventing rickets in Bangladesh, Biological trace element research 121(3) (2008) 193‐204. | Excluded | Title and abstract | Irrelevant |
| 53] H. Blain, A. Jaussent, E. Thomas, J.-P. Micallef, A.-M. Dupuy, P.L. Bernard, D. Mariano-Goulart, J.-P. Cristol, C. Sultan, M. Rossi, M.-C. Picot, Appendicular skeletal muscle mass is the strongest independent factor associated with femoral neck bone mineral density in adult and older men, Experimental Gerontology 45(9) (2010) 679-684. | Excluded | Title and abstract | Irrelevant |
| 54] T.R. Bajgai, F. Hashinaga, S. Isobe, G.S.V. Raghavan, M.O. Ngadi, Application of high electric field (HEF) on the shelf-life extension of emblic fruit (<i>Phyllanthus emblica</i> L.), Journal of Food Engineering 74(3) (2006) 308-313. | Excluded | Title and abstract | Irrelevant |
| 55] Nct, The Application of Microcurrent in Athletes, https://clinicaltrials.gov/show/NCT03477747 (2018). | Excluded | Title and abstract | Irrelevant |
| 56] P. Hepburn, J. Howlett, H. Boeing, A. Cockburn, A. Constable, A. Davi, N. de Jong, B. Moseley, R. Oberdörfer, C. Robertson, J.M. Wal, F. Samuels, The application of post-market monitoring to novel foods, Food and Chemical Toxicology 46(1) (2008) 9-33. | Excluded | Title and abstract | Irrelevant |
| 57] I. Beyitler, S. Kavukcu, Approach to growing pains in childhood in a familial mediterranean fever endemic region, Iranian Journal of Pediatrics 27(6) (2017). | Excluded | Title and abstract | Irrelevant |
| 58] M. Mansourian, N. Rafie, F. Khorvash, A. Hadi, A. Arab, Are serum vitamin D, calcium and phosphorous associated with restless leg syndrome? A systematic review and meta-analysis, Sleep medicine 75 (2020) 326-334. | Excluded | Title and abstract | Irrelevant |
| 59] G. Aras, F. Kadakal, S. Purisa, D. Kanmaz, A. Aynaci, E. Isik, Are We Aware of Restless Legs Syndrome in COPD Patients Who Are in an Exacerbation Period? Frequency and Probable Factors Related to Underlying Mechanism, Copd-Journal of Chronic Obstructive Pulmonary Disease 8(6) (2011) 437-443. | Excluded | Full text | No relevant outcome |
| 60] M.A. García-Jiménez, Arousal and motor activity during sleep, Revista de neurologia 28(6) (1999) 559-565. | Excluded | Title and abstract | Irrelevant |
| 61] N. Yamaguchi, T. Nakamura, D. Dong, Y. Takahashi, S. Amachi, T. Makino, Arsenic release from flooded paddy soils is influenced by speciation, Eh, pH, and iron dissolution, Chemosphere 83(7) (2011) 925-32. | Excluded | Title and abstract | Irrelevant |
| 62] S. Medical Advisory, Arthroscopic lavage and debridement for osteoarthritis of the knee: an evidence-based analysis, Ontario health technology assessment series 5(12) (2005) 1-37. | Excluded | Title and abstract | Irrelevant |
| 63] B. Haezeleer, U. Boettger, J.-P. de Vera, F. Hanke, S. Fox, H. Strasdeit, Artifact formation during Raman measurements and its relevance to the search for chemical biosignatures on Mars, Planetary and Space Science 179 (2019). | Excluded | Title and abstract | Irrelevant |
| 64] A. Gagliano, M. Puligheddu, N. Ronzano, P. Congiu, M.G. Tanca, I. Cursio, S. Carucci, S. Sotgiu, E. Grossi, A. Zuddas, Artificial Neural Networks Analysis of polysomnographic and clinical features in Pediatric Acute-Onset Neuropsychiatric Syndrome (PANS): from sleep alteration to "Brain Fog", Nature and science of sleep 13 (2021) 1209-1224. | Excluded | Title and abstract | Irrelevant |
| 65] T.J. Hureau, J.C. Weavil, S.K. Sidhu, T.S. Thurston, V.R. Reese, J. Zhao, A.D. Nelson, N.M. Birgenheier, R.S. Richardson, M. Amann, Ascorbate attenuates cycling exercise-induced neuromuscular fatigue but fails to improve exertional dyspnea and exercise tolerance in COPD, Journal of Applied Physiology 130(1) (2021) 69-79. | Excluded | Title and abstract | Irrelevant |
| 66] Nct, Ascorbic Acid Treatment in CMT1A Trial (AATIC), https://clinicaltrials.gov/show/NCT00271635 (2006). | Excluded | Title and abstract | Irrelevant |
| 67] U.C. Ghoshal, S. Sachdeva, U. Ghoshal, A. Misra, A.S. Puri, N. Pratap, A. Shah, M.M. Rahman, K.A. Gwee, V.P.Y. Tan, T. Ahmed, Y.Y. Lee, B.S. Ramakrishna, R. Talukdar, S.V. Rana, S.K. Sinha, M. Chen, N. Kim, G. Holtmann, Asian-Pacific consensus on small intestinal bacterial overgrowth in gastrointestinal disorders: An initiative of the Indian Neurogastroenterology and Motility Association, Indian Journal of Gastroenterology 41(5) (2022) 483-507. | Excluded | Title and abstract | Irrelevant |
| 68] Q. Yue, L. Tao, Y. Hou, C. Zhang, Y. Wang, M. Hong, C.-Z. Li, Assay of miRNA in cell samples using enhanced resonance light scattering technique based on self aggregation of magnetic nanoparticles, Nanomedicine 13(18) (2018) 2301-2310. | Excluded | Title and abstract | Irrelevant |
| 69] S. Acharya, R.C. Jena, S.J. Das, C. Pradhan, P.K. Chand, Assessment of air pollution tolerance index of some selected roadside plants of Bhubaneswar city of Odisha State in India, Journal of Environmental Biology 38(6) (2017) 1397-1403. | Excluded | Title and abstract | Irrelevant |
| 70] Actrn, Assessment of Dietary compliance in Type-2 diabetes mellitus patients by sending text message reminders, https://trialsearch.who.int/Trial2.aspx?TrialID=ACTRN12612000538842 (2012). | Excluded | Title and abstract | Irrelevant |
| 71] M. Atar, R.T. Baran, O. Pirgon, N. Aslan, ASSESSMENT OF RESTLESS LEGS SYNDROME DEVELOPMENT AND POOR SLEEP QUALITY IN NON-ANEMIC CHILDREN WITH VITAMIN D DEFICIENCY, Hormone Research in Paediatrics 88 (2017) 95-95. | Included | Full text | Relationship between vitamins and RLS |
| 72] A. Simeonova, N. Neykov, V. Toncheva, I. Stoyanov, G. Savov, V. Vutova, N. Mihnev, M. Mihov, E. Dimitrova, P. Dacheva, D. Jancheva, I. Staikov, Assessment of severity and therapeutical response in 70 patients with idiopathic and symptomatic form of restless leg syndrome, Journal of Sleep Research 23 (2014) 228-228. | Excluded | Title and abstract | Irrelevant |
| 73] P. Gerdhem, K.A. Ringsberg, K.J. Obrant, K. Akesson, Association between 25-hydroxy vitamin D levels, physical activity, muscle strength and fractures in the prospective population-based OPRA Study of Elderly Women, Osteoporosis international 16(11) (2005) 1425‐1431. | Excluded | Title and abstract | Irrelevant |
| 74] S. Saetung, S. Reutrakul, L.-o. Chailurkit, R. Rajatanavin, B. Ongphiphadhanakul, H. Nimitphong, The Association between Daytime Napping Characteristics and Bone Mineral Density in Elderly Thai Women without Osteoporosis, Scientific reports 8 (2018). | Excluded | Title and abstract | Irrelevant |
| 75] C.H. Yun, S.K. Lee, H. Kim, H.K. Park, S.H. Lee, C. Shin, Association between irritable bowel syndrome and restless legs syndrome in general population, Sleep and Biological Rhythms 9(4) (2011) 326. | Excluded | Title and abstract | Irrelevant |
| 76] S. Sun, C. Liu, Y. Jia, J. Wu, H. Li, X. Li, Y. Zhao, Association Between Migraine Complicated With Restless Legs Syndrome and Vitamin D, Front Neurol 12 (2021) 777721. | Included | Full text | Relationship between vitamins and RLS |
| 77] O. Ortancil, A. Sanli, R. Eryuksel, A. Basaran, H. Ankarali, Association between serum ferritin level and fibromyalgia syndrome, European Journal of Clinical Nutrition 64(3) (2010) 308-312. | Excluded | Title and abstract | Irrelevant |
| 78] C. Varım, B.A. Aca, M.S. Uyanık, T. Acar, N. Alagoz, A. Nalbant, T. Kaya, H. Ergenc, Association between the neutrophil-to-lymphocyte ratio,a new marker of systemic inflammation,and restless legs syndrome, Singapore Medical Journal 57(9) (2016) 514-516. | Included | Full text | Relationship between vitamins and RLS |
| 79] S. Wali, S. Alsafadi, B. Abaalkhail, I. Ramadan, B. Abulhamail, M. Kousa, R. Alshamrani, H. Faruqui, A. Faruqui, M. Alama, M. Hamed, The Association Between Vitamin D Level and Restless Legs Syndrome: A Population-Based Case-Control Study, Journal of clinical sleep medicine : JCSM : official publication of the American Academy of Sleep Medicine 14(4) (2018) 557-564. | Included | Full text | Relationship between vitamins and RLS |
| 80] F.J. Jiménez-Jiménez, E. García-Martín, H. Alonso-Navarro, C. Martínez, M. Zurdo, L. Turpín-Fenoll, J. Millán-Pascual, T. Adeva-Bartolomé, E. Cubo, F. Navacerrada, A. Rojo-Sebastián, L. Rubio, S. Ortega-Cubero, P. Pastor, M. Calleja, J.F. Plaza-Nieto, B. Pilo-De-La-Fuente, M. Arroyo-Solera, E. García-Albea, J.A.G. Agúndez, Association Between Vitamin D Receptor rs731236 (Taq1) Polymorphism and Risk for Restless Legs Syndrome in the Spanish Caucasian Population, Medicine 94(47) (2015) e2125. | Excluded | Full text | No relevant outcome |
| 81] P.L. Gordon, J.W. Doyle, K.L. Johansen, Association of 1,25-dihydroxyvitamin D levels with physical performance and thigh muscle cross-sectional area in chronic kidney disease stage 3 and 4, Journal of renal nutrition 22(4) (2012) 423‐433. | Excluded | Title and abstract | Irrelevant |
| 82] L.D. Carbone, B. Gonzalez, S. Miskevics, C. Ray, B. Etingen, M. Guihan, B.C. Craven, V. George, F.M. Weaver, Association of Bisphosphonate Therapy With Incident of Lower Extremity Fractures in Persons With Spinal Cord Injuries or Disorders, Archives of physical medicine and rehabilitation 101(4) (2020) 633‐641. | Excluded | Title and abstract | Irrelevant |
| 83] T. Higuchi, M. Abe, M. Mizuno, T. Yamazaki, H. Suzuki, M. Moriuchi, O. Oikawa, E. Okawa, H. Ando, K. Okada, Association of restless legs syndrome with oxidative stress and inflammation in patients undergoing hemodialysis, Sleep medicine 16(8) (2015) 941-948. | Excluded | Full text | Data couldn't be separately extracted |
| 84] A. Coenders, D. Champion, P. Hannaford, T. Jaaniste, W. Qiu, Associations between chronic pain disorders in adolescents and history of functional pain syndromes: A case-control study, Anaesthesia and intensive care 39(4) (2011) 741. | Excluded | Title and abstract | Irrelevant |
| 85] M.J.F. Reis, T.P. Veloso, F.M.S. Coelho, Atypical manifestation of restless legs syndrome as cold sensation in lower limbs and trunk: A case report, Sleep Science 12 (2019) 61. | Excluded | Title and abstract | Irrelevant |
| 86] B. Frauscher, W.N. Löscher, B. Högl, W. Poewe, M. Kofler, Auditory startle reaction is disinhibited in idiopathic restless legs syndrome, Sleep 30(4) (2007) 489-493. | Excluded | Title and abstract | Irrelevant |
| 87] Nct, Auricular Vagus Nerve Stimulation For Fibromyalgia Syndrome, https://clinicaltrials.gov/show/NCT04260906 (2020). | Excluded | Title and abstract | Irrelevant |
| 88] R.P. Allen, M.J. Armstrong, C. Trenkwalder, P.C. Zee, J.W. Winkelman, Author response: Practice guideline summary: Treatment of restless legs syndrome in adults: Report of the Guideline Development, Dissemination, and Implementation Subcommittee of the American Academy of Neurology, Neurology 88(24) (2017) 2337-2338. | Excluded | Title and abstract | Irrelevant |
| 89] R.C. Lee, H. Kang, C.L. Darling, D. Fried, Automated assessment of the remineralization of artificial enamel lesions with polarization-sensitive optical coherence tomography, Biomedical Optics Express 5(9) (2014) 2950-2962. | Excluded | Title and abstract | Irrelevant |
| 90] A. Oldani, M. Zucconi, R. Asselta, M. Modugno, M.T. Bonati, L. Dalprà, M. Malcovati, M.L. Tenchini, S. Smirne, L. Ferini-Strambi, Autosomal dominant nocturnal frontal lobe epilepsy -: A video-polysomnographic and genetic appraisal of 40 patients and delineation of the epileptic syndrome, Brain : a journal of neurology 121 (1998) 205-223. | Excluded | Title and abstract | Irrelevant |
| 91] J.R. Harrison, N.B. Clark, Avian medullary bone in organ culture: effects of vitamin D metabolites on collagen synthesis, Calcified tissue international 39(1) (1986) 35-43. | Excluded | Title and abstract | Irrelevant |
| 92] P. Ryan, P. Papanek, M.E. Csuka, M.E. Brown, S. Hopkins, S. Lynch, V. Scheer, A. Schlidt, K. Yan, P. Simpson, et al., Background and method of the Striving to be Strong study a RCT testing the efficacy of a m-health self-management intervention, Contemporary clinical trials 71 (2018) 80‐87. | Excluded | Title and abstract | Irrelevant |
| 93] F. Dezoteux, D. Staumont-Salle, Bacterial acute non necrosing cellulitis (erysipelas) in adult, Revue De Medecine Interne 42(3) (2021) 186-192. | Excluded | Title and abstract | Irrelevant |
| 94] W.R. Pigeon, M. Yurcheshen, Behavioral Sleep Medicine Interventions for Restless Legs Syndrome and Periodic Limb Movement Disorder, Sleep Medicine Clinics 4(4) (2009) 487-494. | Excluded | Title and abstract | Irrelevant |
| 95] R. Silvestri, O.S. Ipsiroglu, Behavioral sleep medicine-The need for harmonization of clinical best practice outcome measures in children and adolescents with intellectual or developmental disabilities and restless sleep, Front Psychiatry 13 (2022) 1003019. | Excluded | Title and abstract | Irrelevant |
| 96] R. Silvestri, O.S. Ipsiroglu, Behavioral sleep medicine—The need for harmonization of clinical best practice outcome measures in children and adolescents with intellectual or developmental disabilities and restless sleep, Frontiers in Psychiatry 13 (2022). | Excluded | Title and abstract | Irrelevant |
| 97] Actrn, Benefits and safety of IRon supplementation with MAlaria chemoprevention to children in Malawi (IRMA) - A randomised controlled trial, https://trialsearch.who.int/Trial2.aspx?TrialID=ACTRN12620000386932 (2020). | Excluded | Title and abstract | Irrelevant |
| 98] A. Evans, Benign musculoskeletal paediatric leg-'Just growing pains'-or is it? Reducing the chance of missing anything 'nasty' by adopting the 5 I'S: A clinical model, Internal medicine journal 50(SUPPL 2) (2020) 7. | Excluded | Title and abstract | Irrelevant |
| 99] P. Marceau, F.S. Hould, M. Potvin, S. Lebel, S. Biron, Biliopancreatic diversion (duodenal switch procedure), European journal of gastroenterology & hepatology 11(2) (1999) 99-103. | Excluded | Title and abstract | Irrelevant |
| 100] J.N. Yun, H.S. Kan, J.S. Yeun, J.H. Kim, M. Lee, N. Kim, T.Y. Oh, S.K. Nam, Y.S. Choi, I.S. Kwon, et al., Bioequivalence for a Fixed-Dose Combination Formulation of Bazedoxifene and Cholecalciferol Compared With the Corresponding Single Entities Given Together, Clinical pharmacology in drug development 10(8) (2021) 850‐858. | Excluded | Title and abstract | Irrelevant |
| 101] Nct, Biofilm Accumulation in Zirconia vs. Titanium Implants, https://clinicaltrials.gov/ct2/show/NCT06075017 (2023). | Excluded | Title and abstract | Irrelevant |
| 102] K.H. Schaller, J. Angerer, Biological monitoring in the occupational setting--relationship to cadmium exposure, IARC scientific publications (118) (1992) 53-63. | Excluded | Title and abstract | Irrelevant |
| 103] Y. Bastidas, N. Valera, Y. Solano Rojas, C. Vasquez, Biological parameters of <i>Olla timberlakei</i> Vandenberg (Coleoptera: Coccinellidae) feed with artificial diets, Revista Chilena de Entomologia 45(4) (2019) 619-627. | Excluded | Title and abstract | Irrelevant |
| 104] C. Chomienne, M. Cornic, S. Castaigne, P. Lefebvre, H. de The, A. Dejean, L. Degos, Biological parameters of the efficiency of retinoic acid in acute leukemia, Comptes rendus des seances de la Societe de biologie et de ses filiales 185(6) (1991) 456-63. | Excluded | Title and abstract | Irrelevant |
| 105] Nct, Biometabolic Outcomes After Weight Loss Surgery: an Individualized Approach, https://clinicaltrials.gov/show/NCT04841057 (2021). | Excluded | Title and abstract | Irrelevant |
| 106] B.N. Singh, A.K.S. Rawat, W. Khan, A.H. Naqvi, B.R. Singh, Biosynthesis of Stable Antioxidant ZnO Nanoparticles by <i>Pseudomonas aeruginosa</i> Rhamnolipids, PloS one 9(9) (2014). | Excluded | Title and abstract | Irrelevant |
| 107] B.N. Singh, A.K. Rawat, W. Khan, A.H. Naqvi, B.R. Singh, Biosynthesis of stable antioxidant ZnO nanoparticles by Pseudomonas aeruginosa rhamnolipids, PloS one 9(9) (2014) e106937. | Excluded | Title and abstract | Irrelevant |
| 108] T. Hurley, Z. Zareen, P. Stewart, C. McDonnell, D. McDonald, E. Molloy, Bisphosphonate use in children with cerebral palsy, Cochrane Database of Systematic Reviews (7) (2021). | Excluded | Title and abstract | Irrelevant |
| 109] F.K. Matovu, L. Wattanachanya, M. Beksinska, J.M. Pettifor, K. Ruxrungtham, Bone health and HIV in resource-limited settings: a scoping review, Current Opinion in Hiv and Aids 11(3) (2016) 306-325. | Excluded | Title and abstract | Irrelevant |
| 110] V. Zikan, M. Tyblova, I. Raska, Jr., E. Havrdova, M. Luchavova, D. Michalska, A.A. Kubena, Bone Mineral Density and Body Composition in Men With Multiple Sclerosis Chronically Treated With Low-Dose Glucocorticoids, Physiological Research 61(4) (2012) 405-417. | Excluded | Title and abstract | Irrelevant |
| 111] L. Lillo-Triguero, A. del Castillo, J. Guzman de Villoria, M.J. Moran-Jimenez, A. Guillem, R. Peraita-Adrados, Brain iron accumulation in a blood donor family with restless legs syndrome, Revista de neurologia 68(3) (2019) 107-110. | Excluded | Title and abstract | Irrelevant |
| 112] R. Peraita-Adrados, A. Del Castillo, J. Gúzmande Villoria, M.J. Morán Jiménez, L. Lillo-Triguero, A. Guillem, Brain iron accumulation with restless legs syndrome in a donor blood Spanish family, Journal of Sleep Research 23 (2014) 221. | Excluded | Title and abstract | Irrelevant |
| 113] Actrn, BRAIN Training Trial: balance, Resistance, or INterval Training Trial: a Randomised Controlled Trial of Three Exercise Modalities in Mild Cognitive Impairment, (2017). | Excluded | Title and abstract | Irrelevant |
| 114] L. Ding, T. Zhang, C. Dong, J. Ren, Brightness Analysis per Moving Particle: <i>In</i> <i>Situ</i> Analysis of Alkaline Phosphatase in Living Cells, Analytical Chemistry 94(12) (2022) 5181-5189. | Excluded | Title and abstract | Irrelevant |
| 115] L. Ding, T. Zhang, C. Dong, J. Ren, Brightness Analysis per Moving Particle: In Situ Analysis of Alkaline Phosphatase in Living Cells, Anal Chem 94(12) (2022) 5181-5189. | Excluded | Title and abstract | Irrelevant |
| 116] S. von Manitius, D. Flügel, B. Gievers Steinlein, M. Schnelle, U. von Mandach, A.P. Simões-Wüst, Bryophyllum pinnatum in the treatment of restless legs syndrome: A case series documented with polysomnography, Clinical Case Reports 7(5) (2019) 1012-1020. | Excluded | Title and abstract | Irrelevant |
| 117] S.K. Praharaj, Bupropion and iron for restless leg syndrome: Do they have efficacy similar to ropinirole?, Annals of Indian Academy of Neurology 20(2) (2017) 166-167. | Excluded | Title and abstract | Irrelevant |
| 118] T.A. Schwitalla, R.A. Patel, G. Catalano, A. Nirmalani-Gandhy, Bupropion Hydrochloride Sustained Release and Diurnal Enuresis: A Previously Unreported Adverse Effect, Clinical neuropharmacology 42(6) (2019) 211-213. | Excluded | Title and abstract | Irrelevant |
| 119] Burosumab (crysvita°) in tumourinduced osteomalacia, Prescrire international 32(254) (2023) 295-296. | Excluded | Title and abstract | Irrelevant |
| 120] S.M. Jan de Beur, P.D. Miller, T.J. Weber, M. Peacock, K. Insogna, R. Kumar, F. Rauch, D. Luca, T. Cimms, M.S. Roberts, J. San Martin, T.O. Carpenter, Burosumab for the Treatment of Tumor-Induced Osteomalacia, Journal of Bone and Mineral Research 36(4) (2021) 627-635. | Excluded | Title and abstract | Irrelevant |
| 121] Y.N. Lamb, Burosumab: First Global Approval, Drugs 78(6) (2018) 707-714. | Excluded | Title and abstract | Irrelevant |
| 122] E. Gaffney-Stomberg, L. Lutz, J. Hughes, N. Murphy, L. Marchitelli, S. Cable, A. Young, J. McClung, A calcium and vitamin D fortified food product improves bone adaptation during military training, FASEB journal 28(1) (2014). | Excluded | Title and abstract | Irrelevant |
| 123] A. Papadaki, C. Vardavas, C. Hatzis, A. Kafatos, Calcium, nutrient and food intake of Greek Orthodox Christian monks during a fasting and non-fasting week, Public Health Nutrition 11(10) (2008) 1022-1029. | Excluded | Title and abstract | Irrelevant |
| 124] Nct, Can Correction of Low Vitamin D Status in Infancy Program for a Leaner Body Composition?, https://clinicaltrials.gov/show/NCT02563015 (2015). | Excluded | Title and abstract | Irrelevant |
| 125] J.M. Montpellier, J. Montpellier, Cancer in the Algerian South and the Sahara, Cahiers medicaux de l'union francaise; revue medico-chirurgicale de la France d'outre-mer 3(17) (1948) 103-12. | Excluded | Title and abstract | Irrelevant |
| 126] S. De Waele, S. Van Belle, Cancer-related fatigue, Acta Clinica Belgica 65(6) (2010) 378-385. | Excluded | Title and abstract | Irrelevant |
| 127] P. Subramaniam, N. Nandan, Cariogenic Potential of Pediatric Liquid Medicaments-An <i>in vitro</i> Study, Journal of Clinical Pediatric Dentistry 36(4) (2012) 357-362. | Excluded | Title and abstract | Irrelevant |
| 128] P. Subramaniam, N. Nandan, Cariogenic potential of pediatric liquid medicaments--an in vitro study, The Journal of clinical pediatric dentistry 36(4) (2012) 357-362. | Excluded | Title and abstract | Irrelevant |
| 129] W.P. Schmitt, S. Rohatgi, M. Matiello, Case 15-2023: A 33-Year-Old Man with Paresthesia of the Arms and Legs, New England Journal of Medicine 388(20) (2023) 1893-1900. | Excluded | Title and abstract | Irrelevant |
| 130] S.R. Dodu, J.O. Pobee, A case of burning (or painful) feet syndrome--without obvious dietary deficiency, Ghana medical journal 1 (1962) 50-3. | Excluded | Title and abstract | Irrelevant |
| 131] A. Kaneko, K. Takei, K. Enomoto, T. Mitsui, K. Nomura, S. Iwasaki, T. Maruki, K. Shimazu, A case of Creutzfeldt-Jakob disease exhibiting athetosis in the early stage], No to shinkei = Brain and nerve 51(10) (1999) 887-90. | Excluded | Title and abstract | Irrelevant |
| 132] R. Izumi, N. Suzuki, K. Kato, H. Warita, M. Tateyama, I. Nakashima, Y. Itoyama, A Case of McArdle Disease: Efficacy of Vitamin B6 on Fatigability and Impaired Glycogenolysis, Internal Medicine 49(15) (2010) 1623-1625. | Excluded | Title and abstract | Irrelevant |
| 133] C. Varim, B.A. Acar, T.A. Acar, N.A. Alagoz, A case of osteomalacia initially followed as restless leg syndrome for 6 months, Biomedical Research-India 27(4) (2016) 1284-1287. | Excluded | Title and abstract | Irrelevant |
| 134] D. Mangan, A case report of niacin in the treatment of restless legs syndrome, Medical hypotheses 73(6) (2009) 1072. | Excluded | Title and abstract | Irrelevant |
| 135] X. Liu, Z. Fan, X. Chen, Y. Zhang, F. He, X. Ma, Q. Ke, Case report: A longitudinal study of an unusual rapidly progressive dementia case, Frontiers in Neurology 14 (2023). | Excluded | Title and abstract | Irrelevant |
| 136] P. PoudelJaishi, S.K. Neupane, P.K. Neupane, Case report: Hyperthyroid hypokalemic periodic paralysis, Annals of medicine and surgery (2012) 78 (2022) 103759-103759. | Excluded | Title and abstract | Irrelevant |
| 137] V.G.H. Evidente, J.N. Caviness, C.H. Adler, Case Studies in Movement Disorders, Seminars in neurology 23(3) (2003) 277-284. | Excluded | Title and abstract | Irrelevant |
| 138] S. Okamoto, T. Takegami, T. Mano, A case with peculiar involuntary movement of the toes and discomfort of the foot, so called 'painful legs and moving toes' (Japanese), Clinical Neurology 14(11) (1974) 829-834. | Excluded | Title and abstract | Irrelevant |
| 139] D.M. Morens, A. Grandinetti, C.I. Waslien, C.B. Park, G.W. Ross, L.R. White, Case-control study of idiopathic Parkinson's disease and dietary vitamin E intake, Neurology 46(5) (1996) 1270-1274. | Excluded | Title and abstract | Irrelevant |
| 140] J.M. Melville, K.L. Hoffman, H.E. Jarrard, J.C. Weeks, Cell culture of mechanoreceptor neurons innervating proleg sensory hairs in <i>Manduca sexta</i> larvae, and co-culture with target motoneurons, Cell and Tissue Research 311(1) (2003) 117-130. | Excluded | Title and abstract | Irrelevant |
| 141] J. Torres, Jr., R.M. Klein, H.N. Tung, A.L. Chapman, Cell cycle kinetics of uninfected and feline leukemia virus-infected canine lymphoma cell lines: effects of methotrexate treatment, Virchows Archiv. B, Cell pathology including molecular pathology 33(2) (1980) 139-53. | Excluded | Title and abstract | Irrelevant |
| 142] B.L. Grimaldi, The central role of magnesium deficiency in Tourette's syndrome: causal relationships between magnesium deficiency, altered biochemical pathways and symptoms relating to Tourette's syndrome and several reported comorbid conditions, Medical hypotheses 58(1) (2002) 47-60. | Excluded | Title and abstract | Irrelevant |
| 143] R.B. Mueller, M. Gengenbacher, S. Richter, J. Dudler, B. Möller, J. von Kempis, Change from subcutaneous to intravenous abatacept and back in patients with rheumatoid arthritis as simulation of a vacation: A prospective phase IV, open-label trial (A-BREAK), Arthritis Research and Therapy 18(1) (2016). | Excluded | Title and abstract | Irrelevant |
| 144] Y. Manios, G. Moschonis, K. Koutsikas, S. Papoutsou, I. Petraki, E. Bellou, A. Naoumi, S. Kostea, S. Tanagra, Changes in body composition following a dietary and lifestyle intervention trial: the postmenopausal health study, Maturitas 62(1) (2009) 58‐65. | Excluded | Title and abstract | Irrelevant |
| 145] A. Rossi, B. Decchi, Changes in Ib heteronymous inhibition to soleus motoneurones during cutaneous and muscle nociceptive stimulation in humans, Brain research 774(1-2) (1997) 55-61. | Excluded | Title and abstract | Irrelevant |
| 146] M. Hettchen, S. von Stengel, M. Kohl, M.H. Murphy, M. Shojaa, M. Ghasemikaram, L. Bragonzoni, F. Benvenuti, C. Ripamonti, M.G. Benedetti, et al., Changes in Menopausal Risk Factors in Early Postmenopausal Osteopenic Women After 13 Months of High-Intensity Exercise: the Randomized Controlled ACTLIFE-RCT, Clinical interventions in aging 16 (2021) 83‐96. | Excluded | Title and abstract | Irrelevant |
| 147] G. Vincent, S. Lamon, N. Gant, P.J. Vincent, J.R. MacDonald, J.F. Markworth, J.A. Edge, A.J.R. Hickey, Changes in mitochondrial function and mitochondria associated protein expression in response to 2-weeks of high intensity interval training, Frontiers in physiology 6(FEB) (2015). | Excluded | Title and abstract | Irrelevant |
| 148] R.A. Field, O.A. Young, G.W. Asher, D.M. Foote, Characteristics of male fallow deer muscle at a time of sex-related muscle growth, Growth 49(2) (1985) 190-201. | Excluded | Title and abstract | Irrelevant |
| 149] X. Wang, Y. Zhang, S. Gui, J. Huang, J. Cao, Z. Li, Q. Li, X. Chu, Characterization of Lipid-Based Lyotropic Liquid Crystal and Effects of Guest Molecules on Its Microstructure: a Systematic Review, AAPS PharmSciTech 19(5) (2018) 2023-2040. | Excluded | Title and abstract | Irrelevant |
| 150] Nct, Chewing Gum Containing Vitamin-c to Treat Emesis Gravidarum, https://clinicaltrials.gov/show/NCT04284696 (2020). | Excluded | Title and abstract | Irrelevant |
| 151] Nct, Cholecalciferol Supplementation in Restless Leg Syndrome in Patients With Chronic Kidney Disease, https://clinicaltrials.gov/show/NCT03063190 (2017). | Excluded | Title and abstract | Duplication |
| 152] M.H. Beheshti Moghadam, A.E. Aziza, G. Cherian, Choline and methionine supplementation in layer hens fed flaxseed: effects on hen production performance, egg fatty acid composition, tocopherol content, and oxidative stability, Poultry science 100(9) (2021) 101299. | Excluded | Title and abstract | Irrelevant |
| 153] L.E. Webb, R.C. Johnson, Choline in plasma measured by liquid-chromatography with electrochemical detection, Clinical biochemistry 19(4) (1986) 212-215. | Excluded | Title and abstract | Irrelevant |
| 154] W.L. Stockland, L.G. Blaylock, Choline requirement of pregnant sows and gilts under restricted feeding conditions, Journal of animal science 39(6) (1974) 1113-16. | Excluded | Title and abstract | Irrelevant |
| 155] D. Cattaert, E. Pearlstein, F. Clarac, Cholinergic control of the walking network in the crayfish Procambarus clarkii, Journal of Physiology Paris 89(4-6) (1996) 209-220. | Excluded | Title and abstract | Irrelevant |
| 156] Z.F. Zeng, Y.R. Liang, Y. Chen, X.N. Jing, S.D. Peng, E.X. Tao, Chronic back pain cured by low-dose levodopa: Is it a variant of restless legs syndrome?, Journal of Pain Research 11 (2018) 277-279. | Excluded | Title and abstract | Irrelevant |
| 157] A.V. Chibalin, J.A. Heiny, B. Benziane, A.V. Prokofiev, A.V. Vasiliev, V.V. Kravtsova, I.I. Krivoi, Chronic Nicotine Modifies Skeletal Muscle Na,K-ATPase Activity through Its Interaction with the Nicotinic Acetylcholine Receptor and Phospholemman, PloS one 7(3) (2012). | Excluded | Title and abstract | Irrelevant |
| 158] V. Popescu, V. Cauni, M.S. Petrutescu, M.M. Rustin, R. Bocai, C.R. Turculet, H. Doran, T. Patrascu, A.M. Lazar, D. Cretoiu, V.N. Varlas, B. Mastalier, Chronic Wound Management: From Gauze to Homologous Cellular Matrix, Biomedicines 11(9) (2023). | Excluded | Title and abstract | Irrelevant |
| 159] L. Verde, L. Barrea, C. Vetrani, E. Frias-Toral, S.P. Chapela, R. Jayawardena, G. de Alteriis, A. Docimo, S. Savastano, A. Colao, G. Muscogiuri, Chronotype and Sleep Quality in Obesity: How Do They Change After Menopause?, Current obesity reports 11(4) (2022) 254-262. | Excluded | Title and abstract | Irrelevant |
| 160] J. Latorre, A. Rosendo, L. Olba, E. Sala Planell, Ciclonicate in the atherosclerotic vascular disease of the lower limbs, Current therapeutic research, clinical and experimental 36(5 I) (1984) 970‐978. | Excluded | Title and abstract | Irrelevant |
| 161] T.A. Lonergan, A circadian rhythm in the rate of light-induced electron flow in three leguminous species, Plant physiology 68(5) (1981) 1041-6. | Excluded | Title and abstract | Irrelevant |
| 162] R.M. Elias, M.A. Dalboni, A.C.E. Coelho, R.M.A. Moyses, CKD-MBD: from the Pathogenesis to the Identification and Development of Potential Novel Therapeutic Targets, Current Osteoporosis Reports 16(6) (2018) 693-702. | Excluded | Title and abstract | Irrelevant |
| 163] J. Takaki, T. Nishi, M. Nangaku, H. Shimoyama, T. Inada, N. Matsuyama, H. Kumano, T. Kuboki, Clinical and psychological aspects of restless legs syndrome in uremic patients on hemodialysis, American Journal of Kidney Diseases 41(4) (2003) 833-839. | Excluded | Full text | Data couldn't be separately extracted |
| 164] Nct, Clinical Burden of Anemia in Inflammatory Bowel Disease: therapeutic Trial (RIDART II), https://clinicaltrials.gov/show/NCT04587141 (2020). | Excluded | Title and abstract | Irrelevant |
| 165] H. Zhao, J. Ma, N. Zhang, T. Feng, The clinical characteristics of Parkinson′s disease patients with concomitant periodic limb movements in sleep, National Medical Journal of China 103(23) (2023) 1793-1796. | Excluded | Title and abstract | Irrelevant |
| 166] H.Q. Zhao, J. Ma, N. Zhang, T. Feng, The clinical characteristics of Parkinson's disease patients with concomitant periodic limb movements in sleep], Zhonghua yi xue za zhi 103(23) (2023) 1793-1796. | Excluded | Title and abstract | Irrelevant |
| 167] Nct, Clinical Effects of Nutrition on Metabolic Risk Factors and Mechanisms, https://clinicaltrials.gov/show/NCT02405806 (2015). | Excluded | Title and abstract | Irrelevant |
| 168] A.T. Euctr, CLINICAL EFFICACY AND SAFETY OF TAZAROTENE CREAM 0.05% IN THE INITIAL AND MAINTENANCE THERAPIES OF LAMELLAR ICHTHYOSIS (LI), https://trialsearch.who.int/Trial2.aspx?TrialID=EUCTR2010-022284-35-AT (2011). | Excluded | Title and abstract | Irrelevant |
| 169] L. Hong, J. Zhang, J. Shen, Clinical efficacy of different doses of lipo-prostaglandin E1 in the treatment of painful diabetic peripheral neuropathy, Journal of diabetes and its complications 29(8) (2015) 1283‐1286. | Excluded | Title and abstract | Irrelevant |
| 170] M.L. Paul, R.K. Khan, Z.H. Khan, M.M. Rahman, M. Sinha, Z. Parvez, C.K. Saha, Clinical patterns of polyneuropathy attending in a tertiary level of hospital, Journal of Medicine (Bangladesh) 15(1) (2014) 3-8. | Excluded | Title and abstract | Irrelevant |
| 171] Isrctn, Clinical study to evaluate the efficacy of probiotic product Bio-Kult Infantis as concomitant treatment of acute infectious diarrhoea in children, https://trialsearch.who.int/Trial2.aspx?TrialID=ISRCTN12919460 (2016). | Excluded | Title and abstract | Irrelevant |
| 172] ChiCtr, Clinical trial of Xuhanting granule in the treatment of children's recurrent respiratory tract infections, http://www.who.int/trialsearch/Trial2.aspx?TrialID=ChiCTR2100044017 (2021). | Excluded | Title and abstract | Irrelevant |
| 173] S. Medical Advisory, Clinical utility of vitamin d testing: an evidence-based analysis, Ontario health technology assessment series 10(2) (2010) 1-93. | Excluded | Title and abstract | Irrelevant |
| 174] L.V.D. Camargo, M.S.D. Carvalho, S.K. Shinjo, A.S.B.D. Oliveira, E. Zanoteli, Clinical, Histological, and Immunohistochemical Findings in Inclusion Body Myositis, BioMed research international 2018 (2018). | Excluded | Title and abstract | Irrelevant |
| 175] J. El Helou, E. Sarkis, E. Khneisser, H. Azar, D. Chelala, Clinico-biological characteristics in restless legs syndrome in a population of haemodialysis patients - Role of vitamin C deficiency, European journal of neurology 23 (2016) 744-744. | Included | Full text | Relationship between vitamins and RLS |
| 176] D.J. Read, T.G. Feest, M.A. Nassim, Clonazepam: effective treatment for restless legs syndrome in uraemia, British Medical Journal 283(6296) (1981) 885-886. | Excluded | Title and abstract | Irrelevant |
| 177] X. Hu, X. Wei, J. Ling, J. Chen, Cobalt: An Essential Micronutrient for Plant Growth?, Frontiers in Plant Science 12 (2021). | Excluded | Title and abstract | Irrelevant |
| 178] Y. Ji, Z. Wang, X. Ju, F. Deng, F. Yang, R. He, Co-encapsulation of rutinoside and <i>β</i>-carotene in liposomes modified by rhamnolipid: Antioxidant activity, antibacterial activity, storage stability, and in vitro gastrointestinal digestion, Journal of food science 88(5) (2023) 2064-2077. | Excluded | Title and abstract | Irrelevant |
| 179] Y. Ji, Z. Wang, X. Ju, F. Deng, F. Yang, R. He, Co-encapsulation of rutinoside and β-carotene in liposomes modified by rhamnolipid: Antioxidant activity, antibacterial activity, storage stability, and in vitro gastrointestinal digestion, Journal of food science 88(5) (2023) 2064-2077. | Excluded | Title and abstract | Irrelevant |
| 180] A. Altintop Geçkil, R. Aydoğan Baykara, Coexistence of obstructive sleep apnea syndrome and fibromyalgia, Tuberkuloz ve toraks 70(1) (2022) 37-43. | Excluded | Title and abstract | Irrelevant |
| 181] W. Qubty, D.L. Renaud, Cognitive impairment associated with low ferritin responsive to iron supplementation, Pediatric Neurology 51(6) (2014) 831-833. | Excluded | Title and abstract | Irrelevant |
| 182] W. Balaja, P. Schmidt, A. Fenando, Cold agglutinin disease: A case report with atypical clinical findings, SAGE Open Medical Case Reports 11 (2023). | Excluded | Title and abstract | Irrelevant |
| 183] C. Chang, L.G. Karagounis, Y. Yu, J. Yin, L. Donato-Capel, M. Shevlyakova, W. Sauret, M. Beaumont, H. Huang, E. Offord, et al., Combining nutritional supplementation and progressive physical activity program improves functionality and quality of life in healthy 50y+ volunteers with knee joint discomfort: a baseline-control trial, Osteoarthritis and cartilage 25(Supplement 1) (2017) S293‐S294. | Excluded | Title and abstract | Irrelevant |
| 184] I.C. Dunican, J.H. Walsh, Comment on Gratwicke et al. Nutritional Interventions to Improve Sleep in Team-Sport Athletes: A Narrative Review. <i>Nutrients</i> 2021, <i>13</i>, 1586, Nutrients 13(9) (2021). | Excluded | Title and abstract | Irrelevant |
| 185] I.C. Dunican, J.H. Walsh, Comment on gratwicke et al. Nutritional interventions to improve sleep in team-sport athletes: A narrative review. nutrients 2021, 13, 1586, Nutrients 13(9) (2021). | Excluded | Title and abstract | Irrelevant |
| 186] Nct, Common Limb Length in One-anastomosis Gastric Bypass, https://clinicaltrials.gov/show/NCT04357119 (2020). | Excluded | Title and abstract | Irrelevant |
| 187] F. Thabet, B. Tabarki, Common sleep disorders in children: assessment and treatment, Neurosciences 28(2) (2023) 85-90. | Excluded | Title and abstract | Irrelevant |
| 188] P.M. Nodine, E.E. Matthews, Common sleep disorders: Management strategies and pregnancy outcomes, Journal of Midwifery and Women's Health 58(4) (2013) 368-377. | Excluded | Title and abstract | Irrelevant |
| 189] L.H. Li, H.B. Chen, L.P. Zhang, Z.W. Wang, C.P. Wang, A community-based investigation on restless legs syndrome in a town in China, Sleep medicine 13(4) (2012) 342-345. | Excluded | Title and abstract | Irrelevant |
| 190] R. Magdy, A.S. Othman, E.H. Elsebaie, R.M. Elsayed, W. Abdelrahman, S. Shalaby, M. Saraya, S.E.-S. Abd El-Ghani, Y.K. Ayoub, A. Elshall, A. Elmazny, Comorbid conditions in Egyptian patients with migraine, Neurological research 45(12) (2023) 1100-1110. | Excluded | Title and abstract | Irrelevant |
| 191] Company fires worker after test reveals AIDS drug in his blood, AIDS policy & law 11(2) (1996) 6-7. | Excluded | Title and abstract | Irrelevant |
| 192] F.R. Euctr, Comparaison entre la neutralisation de l'anticoagulation à titre préventif et la neutralisation de l'anticoagulation après constation d'une hémorragie chez des patients traités par antivitamine K après un traumatisme cranien léger, https://trialsearch.who.int/Trial2.aspx?TrialID=EUCTR2013-000421-31-FR (2015). | Excluded | Title and abstract | Irrelevant |
| 193] K. Vishwakarma, J. Kalra, T. Sharma, R. Gupta, Comparative analysis of the efficacy of ropinirole versus bupropion for the treatment of restless leg syndrome, Indian journal of pharmacology 45 (2013) S90. | Excluded | Title and abstract | Irrelevant |
| 194] M.S. Al-Jondeby, I.T. Cabaguing, A.A. Pajarillo, F.A. Hawas, D.H. Mousa, M.H. Al-Sulaiman, F.A. Shaheen, A.A. Al-Khader, Comparative crossover controlled study using polysulphone and vitamin E coated dialyzers, Saudi medical journal 24(3) (2003) 265‐268. | Excluded | Title and abstract | Irrelevant |
| 195] D.G. Scarpelli, G. Tremblay, A.G. Pearse, A comparative cytochemical and cytologic study of vitamin D induced nephrocalcinosis, The American journal of pathology 36 (1960) 331-53. | Excluded | Title and abstract | Irrelevant |
| 196] C.W. Huang, M.J. Lee, L.J. Wang, P.T. Lee, Y.K. Tu, C.W. Hsu, P.Y. Lin, Comparative efficacy and acceptability of treatments for restless legs syndrome in end-stage renal disease: a systematic review and network meta-analysis, Nephrology, dialysis, transplantation : official publication of the European Dialysis and Transplant Association - European Renal Association 35(9) (2020) 1609-1618. | Excluded | Title and abstract | Irrelevant |
| 197] M. Nankar, H. Walimbe, M.N. Ahmed Bijle, U. Kontham, A. Kamath, S. Muchandi, Comparative evaluation of cariogenic and erosive potential of commonly prescribed pediatric liquid medicaments: an in vitro study, The journal of contemporary dental practice 15(1) (2014) 20-5. | Excluded | Title and abstract | Irrelevant |
| 198] C. Pieterse, A Comparative Study of the Homoeopathic Complex Remedy <em>Spascupreel</em> and the Homoeopathic Simplex Remedy<em> Zincum metallicum in a D6 Potency</em> in the Treatment of Restless Leg Syndrome, 1999. | Excluded | Full text | No relevant outcome |
| 199] S. Rafie, M. Jafari, A Comparative study on the effects of vitamin C and Pramipexole on restless legs syndrome treatment in hemodialysis patients: a randomized, doubleblind, placebo-controlled trial, International journal of pharmaceutical research and allied sciences 5(2) (2016) 128‐134. | Included | Full text | Vetamin therapy for RLS |
| 200] Nct, Compared Effect of a Fruit Milk Shake With a Protein-Carbohydrate Supplement on Recovery After Resistance Exercise, https://clinicaltrials.gov/show/NCT01555775 (2012). | Excluded | Title and abstract | Irrelevant |
| 201] F. Mohammadzadeh, E. Saberi Noghabi, R. Noori, S.A. Ahmadi, M. Azarang, A. Delshad Noghabi, Comparing the Effect of Acupressure at the Spleen-10 (Xuehai) Acupoint and Vitamin E on Primary Dysmenorrhea, Medical acupuncture 34(5) (2022) 325‐330. | Excluded | Title and abstract | Irrelevant |
| 202] F. Al Aisari, H. Al-Hashmi, W.A. Mula-Abed, Comparison between serum holotranscobalamin and total vitamin B12 as indicators of vitamin B12 status, Oman Medical Journal 25(1) (2010) 9-12. | Excluded | Title and abstract | Irrelevant |
| 203] H.A. Durham, C.A. Lovelady, R.J. Brouwer, K.M. Krause, T. Ostbye, Comparison of dietary intake of overweight postpartum mothers practicing breastfeeding or formula feeding, Journal of the American Dietetic Association 111(1) (2011) 67‐74. | Excluded | Title and abstract | Irrelevant |
| 204] C.J. Fuller, S.M. Lucas, N. Murinova, C.M. Douville, E. Tolentino, L.A. Braam, E.A. McGee, J.T. Jesurum, Comparison of interictal platelet activation in episodic migraine aura with and without patent foramen ovale, Cephalalgia : an international journal of headache 31 (2011) 63. | Excluded | Title and abstract | Irrelevant |
| 205] Isrctn, A comparison of intravenous iron and placebo (NaCl) for treatment of Restless Legs Syndrome (RLS), https://trialsearch.who.int/Trial2.aspx?TrialID=ISRCTN82469428 (2008). | Excluded | Title and abstract | Irrelevant |
| 206] S.S. Zadeh, K. Begum, Comparison of nutrient intake by sleep status in selected adults in Mysore, India, Nutrition Research and Practice 5(3) (2011) 230-235. | Excluded | Title and abstract | Irrelevant |
| 207] K. Okura, G.J. Lavigne, N. Huynh, C. Manzini, D. Fillipini, J.Y. Montplaisir, Comparison of sleep variables between chronic widespread musculoskeletal pain, insomnia, periodic leg movements syndrome and control subjects in a clinical sleep medicine practice, Sleep medicine 9(4) (2008) 352-361. | Excluded | Title and abstract | Irrelevant |
| 208] H.L. Young, A.A. Stevens, E. Converse, R.G. Mair, A comparison of temporal decay in place memory tasks in rats (Rattus norvegicus) with lesions affecting thalamus, frontal cortex, or the hippocampal system, Behavioral Neuroscience 110(6) (1996) 1244-1260. | Excluded | Title and abstract | Irrelevant |
| 209] M. Pappa, H. Pappas, E. Kokkolou, A. Aimilios, A. Duni, K.P. Rapsomanikis, O. Balafa, G. Dimakopoulos, P. Steiropoulos, L. Pnevmatikos, A. Konstantinidis, M. Mitsis, E. Ntounousi, COMPARISON OF THE CHARACTERISTICS AND SEVERITY OF RESTLESS LEG SYNDROME AND INSOMNIA IN PATIENTS WITH CHRONIC KIDNEY DISEASE AND RENAL TRANSPLANT RECIPIENTS, Nephrology Dialysis Transplantation 35 (2020) 541-541. | Excluded | Title and abstract | Irrelevant |
| 210] Irct2017070134806N, Comparison of the effect of magnesium and vitamin B6 on the improvement of restless leg syndrome, https://trialsearch.who.int/Trial2.aspx?TrialID=IRCT2017070134806N1 (2017). | Excluded | Title and abstract | Irrelevant |
| 211] Actrn, Comparison of the effectiveness of three oral analgesic combinations in adults presenting to the Emergency Department with moderate pain from acute limb injury, https://trialsearch.who.int/Trial2.aspx?TrialID=ACTRN12610000588099 (2010). | Excluded | Title and abstract | Irrelevant |
| 212] P. Christensen, R. Frederiksen, H. Bliddal, B.F. Riecke, E.M. Bartels, M. Henriksen, T. Juul-S Rensen, H. Gudbergsen, K. Winther, A. Astrup, et al., Comparison of three weight maintenance programs on cardiovascular risk, bone and vitamins in sedentary older adults, Obesity (Silver Spring, Md.) 21(10) (2013) 1982‐1990. | Excluded | Title and abstract | Irrelevant |
| 213] S. Atalay, J.M.P.A. Van Den Reek, A.A. Den Broeder, L.J. Van Vugt, M.E. Otero, M.D. Njoo, J.M. Mommers, P.M. Ossenkoppele, M.I. Koetsier, M.A. Berends, P.C.M. Van De Kerkhof, H.M.M. Groenewoud, W. Kievit, E.M.G.J. De Jong, Comparison of Tightly Controlled Dose Reduction of Biologics with Usual Care for Patients with Psoriasis: A Randomized Clinical Trial, JAMA Dermatology 156(4) (2020) 393-400. | Excluded | Title and abstract | Irrelevant |
| 214] S.R. Ruschkowski, F.E. Robinson, K.M. Cheng, L.E. Hart, Comparison of two multiple blood sampling regimens using an indwelling vascular access device for investigations of the hen's ovulatory cycle and calcium metabolism, Poultry science 72(1) (1993) 172-84. | Excluded | Title and abstract | Irrelevant |
| 215] S. Khorsand Ahmadi, M. Mahmoodian Moghadam, P. Mokaberi, M. Reza Saberi, J. Chamani, A comparison study of the interaction between β-lactoglobulin and retinol at two different conditions: spectroscopic and molecular modeling approaches, Journal of biomolecular structure & dynamics 33(9) (2015) 1880-98. | Excluded | Title and abstract | Irrelevant |
| 216] Irct2014071218451N, comparisone between vitaminC versus pramipexole effect on restless leg syndrome treatment in hemodialysis patients, https://trialsearch.who.int/Trial2.aspx?TrialID=IRCT2014071218451N1 (2014). | Excluded | Title and abstract | Irrelevant |
| 217] S.N. Deshpande, D.R. Simkin, Complementary and Integrative Approaches to Sleep Disorders in Children, Child and adolescent psychiatric clinics of North America 32(2) (2023) 243-272. | Excluded | Title and abstract | Irrelevant |
| 218] J.J. Hill, N.M. Wolfman, Composition containing growth and differentiation factor-associated serum protein-1, useful for treating e.g. muscular dystrophy or diabetes, also for diagnosis, Wyeth; Wyeth Corp; Wyeth Llc; Hill J J; Wolfman N M. | Excluded | Title and abstract | Irrelevant |
| 219] O. Yacoby-Zeevi, Z.O. Yacoby, Composition to treat disease associated with loss of dopamine or dopaminergic neurons e.g. Parkinson's disease and Huntington's disease, comprises carbidopa, at least two antioxidants in which one is ascorbic acid or its salt, and carrier, Neuroderm Ltd; Yacoby-Zeevi O; Neuroderm Co Ltd. | Excluded | Title and abstract | Irrelevant |
| 220] L. Velázquez-Pérez, R. Rodríguez-Labrada, J.C. García-Rodríguez, L.E. Almaguer-Mederos, T. Cruz-Mariño, J.M. Laffita-Mesa, A comprehensive review of spinocerebellar ataxia type 2 in Cuba, Cerebellum (London, England) 10(2) (2011) 184-198. | Excluded | Title and abstract | Irrelevant |
| 221] J. Law, J.B. Li, L. Zhou, Conditions associated with small fiber sensory neuropathy, Neurology 66(5) (2006) A44-A44. | Excluded | Title and abstract | Irrelevant |
| 222] jRct, Confirmatory study of EN-P09 in patients with enteral tube feeding, https://trialsearch.who.int/Trial2.aspx?TrialID=JPRN-jRCT2031210285 (2021). Excluded Title and abstract Irrelevant | Excluded | Title and abstract | Irrelevant |
| 223] B. Peng, Z. Zhang, J.R. Wang, M. Li, Q. Zhang, X. Mei, Confocal Raman micro-spectral evidence and physicochemical evaluation of triamterene salts, The Analyst 144(2) (2019) 530-535. | Excluded | Title and abstract | Irrelevant |
| 224] D.L. Picchietti, J.G. Hensley, J.L. Bainbridge, K.A. Lee, M. Manconi, J.A. McGregor, R.M. Silver, C. Trenkwalder, A.S. Walters, G. Int Restless Legs Syndrome Study, Consensus clinical practice guidelines for the diagnosis and treatment of restless legs syndrome/Willis-Ekbom disease during pregnancy and lactation, Sleep Medicine Reviews 22 (2015) 64-77. | Excluded | Title and abstract | Irrelevant |
| 225] J. Ahmed, B.R. Giri, L. Thomas, H. Al-Attar, M. Maniruzzaman, Continuous manufacturing of vitamin D3 and iron enriched granules by means of a novel twin-screw dry granulation process, Powder Technology 412 (2022). | Excluded | Title and abstract | Irrelevant |
| 226] D. Bohrer, P.C. Do Nascimento, R. Binotto, E. Becker, S. Pomblum, Contribution of the raw material to the aluminum contamination in parenterals, Journal of Parenteral and Enteral Nutrition 26(6) (2002) 382-388. | Excluded | Title and abstract | Irrelevant |
| 227] S.Z. Fadem, F. Al-Saghir, G. Zollner, S. Swan, Converting hemodialysis patients from intravenous paricalcitol to intravenous doxercalciferol - A dose equivalency and titration study, Clinical nephrology 70(4) (2008) 319‐324. | Excluded | Title and abstract | Irrelevant |
| 228] M. Willerslev-Olsen, J. Lorentzen, K. Røhder, A. Ritterband-Rosenbaum, M. Justiniano, A. Guzzetta, A.V. Lando, A.B. Jensen, G. Greisen, S. Ejlersen, et al., COpenhagen Neuroplastic TRaining against Contractures in Toddlers (CONTRACT): protocol of an open-label randomised clinical trial with blinded assessment for prevention of contractures in infants with high risk of cerebral palsy, BMJ open 11(7) (2021) e044674. | Excluded | Title and abstract | Irrelevant |
| 229] P. Haueis, S. Russmann, O.I. Zorina, R. Grohmann, G.A. Kullak-Ublick, E. Jaquenoud Sirot, H. Russmann, Coprescription of levodopa with antipsychotics in a population of 84 596 psychiatric inpatients from 1994 to 2008, Pharmacopsychiatry 45(4) (2012) 127-132. | Excluded | Title and abstract | Irrelevant |
| 230] Nct, Correcting GUT microbioTa by Combined Supplementation of fibERs and bIotiN to Improve Microbiome and Optimize Bariatric Surgery Outcomes, https://clinicaltrials.gov/show/NCT05832190 (2023). | Excluded | Title and abstract | Irrelevant |
| 231] F.J. Jiménez-Jiménez, G. Amo, H. Alonso-Navarro, M. Calleja, M. Díez-Fairén, I. Alvarez, P. Pastor, J.F. Plaza-Nieto, S. Navarro-Muñoz, L. Turpín-Fenoll, J. Millán-Pascual, M. Recio-Bermejo, R. García-Ruiz, E. García-Albea, J.A.G. Agúndez, E. García-Martín, Correction to: Serum vitamin D, vitamin D receptor and binding protein genes polymorphisms in restless legs syndrome, Journal of neurology 268(4) (2021) 1473. | Excluded | Title and abstract | Irrelevant |
| 232] F.J. Jiménez-Jiménez, G. Amo, H. Alonso-Navarro, M. Calleja, M. Díez-Fairén, I. Alvarez, P. Pastor, J.F. Plaza-Nieto, S. Navarro-Muñoz, L. Turpín-Fenoll, J. Millán-Pascual, M. Recio-Bermejo, R. García-Ruiz, E. García-Albea, J.A.G. Agúndez, E. García-Martín, Correction to: Serum vitamin D, vitamin D receptor and binding protein genes polymorphisms in restless legs syndrome (Journal of Neurology, (2021), 268, 4, (1461-1472), 10.1007/s00415-020-10312-9), Journal of neurology 268(4) (2021) 1473. | Excluded | Title and abstract | Irrelevant |
| 233] A.M. Evans, T. Berde, L. Karimi, P. Ranade, N. Shah, R. Khubchandani, Correlates and predictors of paediatric leg pain: a case-control study, Rheumatol Int 38(7) (2018) 1251-1258. | Included | Full text | Relationship between vitamins and RLS |
| 234] A.M. Evans, T. Berde, L. Karimi, P. Ranade, N. Shah, R. Khubchandani, Correlates and predictors of paediatric leg pain: a case–control study, Rheumatology International 38(7) (2018) 1251-1258. | Excluded | Title and abstract | Duplication |
| 235] C. Geng, Z. Yang, X. Kong, P. Xu, H. Zhang, Correlation between vitamin D and poor sleep status in restless legs syndrome, Front Endocrinol (Lausanne) 13 (2022) 994545. | Excluded | Full text | No relevant outcome |
| 236] P. Morganti, G. Agostini, G. Fabrizi, The cosmetic use of an ancient peat of thermal origin, Journal of applied cosmetology 19(1) (2001) 21‐30. | Excluded | Title and abstract | Irrelevant |
| 237] A. Kucuk, R.A. Baykara, A. Tuzcu, A. Omma, M.C. Cure, E. Cure, G.K. Acet, E. Dogan, Could ferritin, vitamin B12, and vitamin D play a role in the etiopathogenesis of fibromyalgia syndrome?, Romanian journal of internal medicine = Revue roumaine de medecine interne 59(4) (2021) 384-393. | Excluded | Title and abstract | Irrelevant |
| 238] A.A. Tony, E.A. Tony, S.B. Ali, A.M. Ezzeldin, A.A. Mahmoud, COVID-19-associated sleep disorders: A case report, Neurobiology of Sleep and Circadian Rhythms 9 (2020). | Excluded | Title and abstract | Irrelevant |
| 239] A.M. Whiteley, Cramps, stiffness and restless legs, The Practitioner 226(1368) (1982) 1085-1189. | Excluded | Title and abstract | Irrelevant |
| 240] T.N. Kekelidze, J.M. Benzecry, R.C. Deth, CREATINE LEVELS AFFECT FOLATE - DEPENDENT PHOSPHOLIPID METHYLATION IN HUMAN NEUROBLASTOMA CELLS, Society for Neuroscience Abstract Viewer and Itinerary Planner 2002 (2002) Abstract No. 305.20-Abstract No. 305.20. | Excluded | Title and abstract | Irrelevant |
| 241] L.I. Varga, N. Ako-Agugua, J. Colasante, L. Hertweck, T. Houser, J. Smith, A.A. Watty, S. Nagar, R.B. Raffa, Critical review of ropinirole and Pramipexole - Putative dopamine D 3-receptor selective agonists - For the treatment of RLS, Journal of clinical pharmacy and therapeutics 34(5) (2009) 493‐505. | Excluded | Title and abstract | Irrelevant |
| 242] D. Seyhan Erdoğan, G. Benbir Şenel, A. Gündüz, B.P. Uçar, T. Elverdi, A. Salihoğlu, M.C. Ar, Ş. Öngören, Z. Başlar, A.E. Eşkazan, A cross-sectional study on restless legs syndrome (RLS) in polycythemia vera (PV): is iron deficiency the only culprit?, Neurological research 45(12) (2023) 1144-1151. | Excluded | Title and abstract | Irrelevant |
| 243] L. Nimmagadda, G.K. Narayanaswamy, Cryptic red light signal regulates ascorbic acid in soybean, Journal of Plant Physiology 166(3) (2009) 329-332. | Excluded | Title and abstract | Irrelevant |
| 244] A.O. Ozdemir, A. Tamayo, C. Munoz, B. Dias, J.D. Spence, Cryptogenic stroke and patent foramen ovale: Clinical clues to paradoxical embolism, Journal of the neurological sciences 275(1-2) (2008) 121-127. | Excluded | Title and abstract | Irrelevant |
| 245] S.M. Tan, A curious case of restless legs syndrome masquerading as akathisia, Parkinsonism and Related Disorders 46 (2018) e58. | Excluded | Title and abstract | Irrelevant |
| 246] W.A. Hening, Current Guidelines and Standards of Practice for Restless Legs Syndrome, American Journal of Medicine 120(1 SUPPL. 1) (2007) S22-S27. | Excluded | Title and abstract | Irrelevant |
| 247] G.K. Sakkas, C.D. Giannaki, C. Karatzaferi, M. Maridaki, Y. Koutedakis, G.M. Hadjigeorgiou, I. Stefanidis, Current trends in the management of uremic restless legs syndrome: a systematic review on aspects related to quality of life, cardiovascular mortality and survival, Sleep Med Rev 21 (2015) 39-49. | Excluded | Title and abstract | Irrelevant |
| 248] J.F. Morley, H.I. Hurtig, Current understanding and management of Parkinson disease: Five new things, Neurology 75(18 SUPPL.1) (2010) S9-S15. | Excluded | Title and abstract | Irrelevant |
| 249] A. Rossi, B. Decchi, Cutaneous nociceptive facilitation of Ib heteronymous pathways to lower limb motoneurones in humans, Brain research 700(1-2) (1995) 164-72. | Excluded | Title and abstract | Irrelevant |
| 250] U. Raucci, O. Borrelli, G. Di Nardo, R. Tambucci, P. Pavone, S. Salvatore, M.E. Baldassarre, D.M. Cordelli, R. Falsaperla, E. Felici, M.A.N. Ferilli, S. Grosso, S. Mallardo, D. Martinelli, P. Quitadamo, L. Pensabene, C. Romano, S. Savasta, A. Spalice, C. Strisciuglio, A. Suppiej, M. Valeriani, L. Zenzeri, A. Verrotti, A. Staiano, M.P. Villa, M. Ruggieri, P. Striano, P. Parisi, Cyclic Vomiting Syndrome in Children, Frontiers in Neurology 11 (2020). | Excluded | Title and abstract | Irrelevant |
| 251] A. Sharma, M.L. Kramer, P.F. Wick, D. Liu, S. Chari, S. Shim, W. Tan, D. Ouellette, M. Nagata, C.J. DuRand, M. Kotb, R.C. Deth, D4 dopamine receptor-mediated phospholipid methylation and its implications for mental illnesses such as schizophrenia, Molecular psychiatry 4(3) (1999) 235-46. | Excluded | Title and abstract | Irrelevant |
| 252] H.L. Ngo, K.H. Quesenberry, Day length and media effects on <i>Arachis pintoi</i> regeneration <i>in vitro</i>, Soil and Crop Science Society of Florida Proceedings 59 (2000) 90-93. | Excluded | Title and abstract | Irrelevant |
| 253] N. Furudate, Y. Komada, M. Kobayashi, S. Nakajima, Y. Inoue, Daytime dysfunction in children with restless legs syndrome, Journal of the neurological sciences 336(1-2) (2014) 232-236. | Excluded | Title and abstract | Irrelevant |
| 254] Nct, D-dimer Levels During and After Anticoagulation in Patients With a Previous Venous Thromboembolism: effects on the Risk of Recurrence, https://clinicaltrials.gov/show/NCT00266045 (2005). | Excluded | Title and abstract | Irrelevant |
| 255] E. Kilic, G. Halac, S. Kesgin, K. Celik, M. Cikrikcioglu, A. Erek Toprak, M. Nasifov, B. Gulen, G. Kocaman, N. Ozaras, Decrease of Urotensin-II activity in women with restless legs syndrome, Clinical chemistry 63 (2017) S45. | Excluded | Title and abstract | Irrelevant |
| 256] M. O'Keeffe, S.J. Kamper, L. Montgomery, A. Williams, A. Martiniuk, B. Lucas, A.B. Dario, M.S. Rathleff, L. Hestbaek, C.M. Williams, Defining Growing Pains: A Scoping Review, Pediatrics 150(2) (2022). | Excluded | Title and abstract | Irrelevant |
| 257] W.D. Biggar, L. Politano, V.A. Harris, L. Passamano, J. Vajsar, B. Alman, A. Palladino, L.I. Comi, G. Nigro, Deflazacort in Duchenne muscular dystrophy: a comparison of two different protocols, Neuromuscular Disorders 14(8-9) (2004) 476-482. | Excluded | Title and abstract | Irrelevant |
| 258] A.M. Kenny, R.S. Boxer, A. Kleppinger, J. Brindisi, R. Feinn, J.A. Burleson, Dehydroepiandrosterone combined with exercise improves muscle strength and physical function in frail older women, Journal of the American Geriatrics Society 58(9) (2010) 1707‐1714. | Excluded | Title and abstract | Irrelevant |
| 259] E. Aragüés Ortiz De Zárate, P. Malo Ocejo, L. Pacheco Yáñez, M. Etxebeste Portugal, M. Aragüés Figuero, Delusion of parasitosis. Review and clinical cases, Anales de Psiquiatria 15(6) (1999) 241-245. | Excluded | Title and abstract | Irrelevant |
| 260] L.S. Kimsey, Delusional Infestation and Chronic Pruritus: A Review, Acta Dermato-Venereologica 96(3) (2016) 298-302. | Excluded | Title and abstract | Irrelevant |
| 261] J. Ramirez-Bermudez, M. Espinola-Nadurille, N. Loza-Taylor, Delusional parasitosis in neurological patients, General Hospital Psychiatry 32(3) (2010) 294-299. | Excluded | Title and abstract | Irrelevant |
| 262] K. Trigka, P. Dousdampanis, C. Fourtounas, Delusional parasitosis: A rare cause of pruritus in hemodialysis patients, International Journal of Artificial Organs 35(5) (2012) 400-403. | Excluded | Title and abstract | Irrelevant |
| 263] M. De Deyn, Q.X. Ng, Delusional parasitosis: A scoping review, British Journal of Dermatology 185(3) (2021) e108. | Excluded | Title and abstract | Irrelevant |
| 264] A. Skott, Delusions of dermal parasitosis: Ekbom's syndrome (Swedish), NORD.PSYKIAT.T. 29(2) (1975) 115-131. | Excluded | Title and abstract | Irrelevant |
| 265] K. Kedzierska-Kapuza, U. Szczuko, H. Stolinska, D.R. Bakaloudi, W. Wierzba, M. Szczuko, Demand for Water-Soluble Vitamins in a Group of Patients with CKD versus Interventions and Supplementation-A Systematic Review, Nutrients 15(4) (2023). | Excluded | Title and abstract | Irrelevant |
| 266] F.C.V. Potocnik, Dementia, South African Journal of Psychiatry 19(3) (2013) 141-152. | Excluded | Title and abstract | Irrelevant |
| 267] H.Z. Movat, S.G. Steinberg, F.M. Habal, N.S. Ranadive, Demonstration of a kinin-generating enzyme in the lysosomes of human polymorphonuclear leukocytes, Laboratory investigation; a journal of technical methods and pathology 29(6) (1973) 669-84. | Excluded | Title and abstract | Irrelevant |
| 268] T. Sohrabi, M. Asadzadeh-Lotfabad, Z. Shafie, Z.A. Tehranizadeh, M.R. Saberi, J. Chamani, Description of the calf thymus DNA-malathion complex behavior by multi-spectroscopic and molecular modeling techniques: EMF at low and high frequency approaches, Iranian Journal of Basic Medical Sciences 24(10) (2021) 1346-1357. | Excluded | Title and abstract | Irrelevant |
| 269] J. Burns, S. Ramchandren, M.M. Ryan, M. Shy, R.A. Ouvrier, Determinants of reduced health-related quality of life in pediatric inherited neuropathies, Neurology 75(8) (2010) 726-731. | Excluded | Title and abstract | Irrelevant |
| 270] A. Miyazaki, M. Takahashi, T. Shuo, H. Eto, H. Kondo, Determination of optimal 25-hydroxyvitamin D cutoff values for the evaluation of restless legs syndrome among pregnant women, Journal of clinical sleep medicine : JCSM : official publication of the American Academy of Sleep Medicine 19(1) (2023) 73-83. | Included | Full text | Relationship between vitamins and RLS |
|  | Excluded | Title and abstract | Irrelevant |
| 271] B. Yang, T. Zhang, W. Tan, P. Liu, Z. Ding, Q. Cao, Determination of rhodium by resonance light-scattering technique coupled with solid phase extraction using Rh(III) ion-imprinted polymers as sorbent, Talanta 105 (2013) 124-30. | Excluded | Title and abstract | Irrelevant |
| 272] A. Sravanthi, M. Sunitha Reddy, A. Jaswanth, Development and in vitro evaluation of a zero order drug releasing transdermal system of rotigotine, International Journal of Pharmaceutical Sciences Review and Research 66(1) (2021) 54-64. | Excluded | Title and abstract | Irrelevant |
| 273] K. Ćwiek-Ludwicka, M. Gromulska, Development of food and nutrition sciences in the 100-year history of the National Institute of Hygiene in Poland], Przeglad epidemiologiczny 72(4) (2018) 537-547. | Excluded | Title and abstract | Irrelevant |
| 274] R.E. Salas, C.E. Gamaldo, Diagnostic and therapeutic considerations in sleep disorders case studies and commentary, Journal of Clinical Outcomes Management 18(3) (2011) 129-144. | Excluded | Title and abstract | Irrelevant |
| 275] A. Garg, P. Chilakamarri, B.B. Koo, Diagnostic and Treatment Considerations in Restless Legs Syndrome Complicated by Diabetic Neuropathy, Current diabetes reports 21(12) (2021). | Excluded | Title and abstract | Irrelevant |
| 276] J.G.G. Hou, J.P. Chang, M.T. Lin, Diencephalic dopaminergic neuron involvement in restless legs syndrome;: an <i>in vivo</i> rat model study, Neurology 64(6) (2005) A262-A262. | Excluded | Title and abstract | Irrelevant |
| 277] J.H. Hankin, V. Rawlings, Diet and breast cancer: a review, The American journal of clinical nutrition 31(11) (1978) 2005-16. | Excluded | Title and abstract | Irrelevant |
| 278] S. Bingham, J.H. Cummings, N.I. McNeil, Diet and health of people with an ileostomy. 1. Dietary assessment, The British journal of nutrition 47(3) (1982) 399-406. | Excluded | Title and abstract | Irrelevant |
| 279] M.J. Khan, U. Ullah, B. Shahzad, G. Khubana, R. Mehdi, H. Ohly, M.R. Broadley, M. Zaman, M.H. Zia, H.J. McArdle, et al., Dietary characteristics of adult women participating in the BiZiFED trial (Biofortified Zinc Flour to Eliminate Zinc Deficiency), Pakistan, Proceedings of the Nutrition Society 77(OCE4) (2018). | Excluded | Title and abstract | Irrelevant |
| 280] J. Makurat, E.C. Kretz, F.T. Wieringa, C. Chamnan, M.B. Krawinkel, Dietary Diversity in Cambodian Garment Workers: the Role of Free Lunch Provision, Nutrients 10(8) (2018). | Excluded | Title and abstract | Irrelevant |
| 281] Nct, Dietary Intervention and Assessment of Obesity-related Gene Methylation Levels in Overweight Women, https://clinicaltrials.gov/show/NCT04523532 (2020). | Excluded | Title and abstract | Irrelevant |
| 282] B. Szepesi, R.A. Freedland, Dietary regulation of pyruvate kinase synthesis in rat liver, The Journal of nutrition 95(4) (1968) 591-602. | Excluded | Title and abstract | Irrelevant |
| 283] K. Kordas, D. Olney, S. Khalfan, P. Kariger, S. Sazawal, R. Black, J. Tielsch, R.J. Stoltzfus, Differences in hemoglobin are not associated with sleep patterns among Zanzibari infants, Faseb Journal 19(5) (2005) A1493-A1493. | Excluded | Title and abstract | Irrelevant |
| 284] H. Iwaki, C. Blauwendraat, H.L. Leonard, M.B. Makarious, J.J. Kim, G. Liu, J. Maple-Grødem, J.C. Corvol, L. Pihlstrøm, M. van Nimwegen, L. Smolensky, N. Amondikar, S.J. Hutten, M. Frasier, K.D.H. Nguyen, J. Rick, S. Eberly, F. Faghri, P. Auinger, K.M. Scott, R. Wijeyekoon, V.M. Van Deerlin, D.G. Hernandez, R.J. Gibbs, A.G. Day-Williams, A. Brice, G. Alves, A.J. Noyce, O.B. Tysnes, J.R. Evans, D.P. Breen, K. Estrada, C.E. Wegel, F. Danjou, D.K. Simon, O.A. Andreassen, B. Ravina, M. Toft, P. Heutink, B.R. Bloem, D. Weintraub, R.A. Barker, C.H. Williams-Gray, B.P. van de Warrenburg, J.J. Van Hilten, C.R. Scherzer, A.B. Singleton, M.A. Nalls, Differences in the Presentation and Progression of Parkinson's Disease by Sex, Movement Disorders 36(1) (2021) 106-117. | Excluded | Title and abstract | Irrelevant |
| 285] S. Chokroverty, Differential Diagnoses of Restless Legs Syndrome/Willis-Ekbom Disease Mimics and Comorbidities, Sleep Medicine Clinics 10(3) (2015) 249-262. | Excluded | Title and abstract | Irrelevant |
| 286] I. Türkan, M. Bor, F. Özdemir, H. Koca, Differential responses of lipid peroxidation and antioxidants in the leaves of drought-tolerant <i>P-acutifolius</i> Gray and drought-sensitive <i>P-vulgaris</i> L. subjected to polyethylene glycol mediated water stress, Plant Science 168(1) (2005) 223-231. | Excluded | Title and abstract | Irrelevant |
| 287] A.Q. Rana, F. Khan, A. Mosabbir, W. Ondo, Differentiating nocturnal leg cramps and restless legs syndrome, Expert review of neurotherapeutics 14(7) (2014) 813-818. | Excluded | Title and abstract | Irrelevant |
| 288] B. Guldager, O. Faergeman, S.J. Jørgensen, E. Nexø, R. Jelnes, Disodium-ethylene diamine tetraacetic acid (EDTA) has no effect on blood lipids in atherosclerotic patients. A randomized, placebo-controlled study, Danish medical bulletin 40(5) (1993) 625‐627. | Excluded | Title and abstract | Irrelevant |
| 289] H.Z. Al-Sharif, A.P. Thomas, Distinguishing between kleine-levin syndrome and bipolar disorder, a delayed diagnosis of a rare sleep disorder, Sleep 42 (2019) A424. | Excluded | Title and abstract | Irrelevant |
| 290] J. Delrue, B. Soenens, S. Morbée, M. Vansteenkiste, L. Haerens, Do athletes' responses to coach autonomy support and control depend on the situation and athletes' personal motivation?, Psychology of sport and exercise 43 (2019) 321‐332. | Excluded | Title and abstract | Irrelevant |
| 291] L.A. Hershey, Do women with restless legs syndrome have less bone loss?, Neurology 86(13) (2016) e144-e146. | Excluded | Title and abstract | Irrelevant |
| 292] Drks, Does a whole-food, vegan diet provide the body with a sufficient supply of vitamin B12 and vitamin D? A follow-up study, https://trialsearch.who.int/Trial2.aspx?TrialID=DRKS00004994 (2013). | Excluded | Title and abstract | Irrelevant |
| 293] H. Martin, A.A. Sayer, K. Jameson, H. Syddall, E.M. Dennison, C. Cooper, S. Robinson, Does diet influence physical performance in community-dwelling older people? Findings from the Hertfordshire Cohort Study, Age and ageing 40(2) (2011) 181-186. | Excluded | Title and abstract | Irrelevant |
| 294] G.M. Rosen, S. Morrissette, A. Larson, P. Stading, T.L. Barnes, Does Improvement of Low Serum Ferritin Improve Symptoms of Restless Legs Syndrome in a Cohort of Pediatric Patients?, Journal of clinical sleep medicine : JCSM : official publication of the American Academy of Sleep Medicine 15(8) (2019) 1149-1154. | Excluded | Full text | Without control |
| 295] Actrn, Does resistance exercise training improve muscle strength and function in acute burn injury?, https://trialsearch.who.int/Trial2.aspx?TrialID=ACTRN12614001156673 (2014). | Excluded | Title and abstract | Irrelevant |
| 296] R. Wang, M. Alen, Z. Yu, P. Wiklund, S.M. Cheng, T. Törmäkangas, P. Chen, S. Cheng, Does serum 25-hydroxyvitamin D influence muscle development during puberty in girls? A 7-year longitudinal study, PloS one 8(12) (2013) e82124. | Excluded | Title and abstract | Irrelevant |
| 297] A.J. Schou, C. Heuck, O.D. Wolthers, Does vitamin D administered to children with asthma treated with inhaled glucocorticoids affect short-term growth or bone turnover?, Pediatric pulmonology 36(5) (2003) 399‐404. | Excluded | Title and abstract | Irrelevant |
| 298] A. Stefani, T. Mitterling, G. Weiss, B. Hoegl, Does vitamin D play a role in restless legs syndrome/Willis Ekbom disease?, European journal of neurology 23 (2016) 51-51. | Included | Full text | Relationship between vitamins and RLS |
| 299] M. Anstead, Does your sleepy patient have restless legs syndrome?, Journal of Respiratory Diseases 19(7) (1998) 563-570. | Excluded | Title and abstract | Irrelevant |
| 300] Q.C. Du, Y.Y. Ge, W.L. Xiao, W.F. Wang, Dopamine agonist responsive burning mouth syndrome: Report of eight cases, World Journal of Clinical Cases 9(23) (2021) 6916-6921. | Excluded | Title and abstract | Irrelevant |
| 301] A. de Donato, V. Buonincontri, G. Borriello, G. Martinelli, P. Mone, The Dopamine System: Insights between Kidney and Brain, Kidney & blood pressure research 47(8) (2022) 493-505. | Excluded | Title and abstract | Irrelevant |
| 302] ChiCtr, A double blinded randomized cross-over study of vitamin k2 in the management of nocturnal leg cramps(NLC) in the elderly, https://trialsearch.who.int/Trial2.aspx?TrialID=ChiCTR2200059622 (2022). | Excluded | Title and abstract | Irrelevant |
| 303] G.B. Euctr, A double-blind, parallel group, randomized, placebo controlled study of the efficacy of Circadin® 2mg in alleviating sleep disturbances in patients with mild to moderate Alzheimer Disease (AD) treated with Acetylcholinesterase (AChE) inhibitor. - Sleep problems in patients with mild to moderate Alzheimers Disease, https://trialsearch.who.int/Trial2.aspx?TrialID=EUCTR2009-014388-38-GB (2010). | Excluded | Title and abstract | Irrelevant |
| 304] B.E. Euctr, A double-blind, placebo controlled, randomized, comparative, mono-centre trial to assess the impact of a monthly administration of 50.000 IU of Vitamin D during 6 months on Vitamin D level in healthy young adult volunteers. - D-plus, https://trialsearch.who.int/Trial2.aspx?TrialID=EUCTR2010-022454-17-BE (2010). | Excluded | Title and abstract | Irrelevant |
| 305] C. Desnuelle, M. Dib, C. Garrel, A. Favier, A double-blind, placebo-controlled randomized clinical trial of alpha-tocopherol (vitamin E) in the treatment of amyotrophic lateral sclerosis. ALS riluzole-tocopherol Study Group, Amyotrophic lateral sclerosis and other motor neuron disorders 2(1) (2001) 9‐18. | Excluded | Title and abstract | Irrelevant |
| 306] C. Desnuelle, M. Dib, C. Garrel, A. Favier, A double-blind, placeho-controlled randomized clinical trial of alpha-tocopherol (vitamin E) in the treatment of amyotrophic lateral sclerosis, Amyotrophic lateral sclerosis and other motor neuron disorders 2(1) (2001) 9‐18. | Excluded | Title and abstract | Irrelevant |
| 307] K. Vishwakarma, J. Kalra, R. Gupta, M. Sharma, T. Sharma, A double-blind, randomized, controlled trial to compare the efficacy and tolerability of fixed doses of ropinirole, bupropion, and iron in treatment of restless legs syndrome (Willis-Ekbom disease), Annals of Indian Academy of Neurology 19(4) (2016) 472-477. | Excluded | Title and abstract | Irrelevant |
| 308] A.J. Cruz-Jentoft, B. Boland, L. Rexach, Drug therapy optimization at the end of life, Drugs and Aging 29(6) (2012) 511-521. | Excluded | Title and abstract | Irrelevant |
| 309] V. Rajashekaraiah, A.B. Ananthakrishna, Drug-induced thrombocytopenia – etiology and alternative therapeutic approaches, European Journal of Clinical and Experimental Medicine 21(3) (2023) 617-626. | Excluded | Title and abstract | Irrelevant |
| 310] S.R. Davis, F. Jane, Drugs for the treatment of menopausal symptoms, Expert opinion on pharmacotherapy 11(8) (2010) 1329-1341. | Excluded | Title and abstract | Irrelevant |
| 311] M.M. Verduijn, H. Folmer, L.W. Draijer, The Dutch College of General Practitioners pharmacotherapeutic guidelines on nocturnal leg cramps, Huisarts en Wetenschap 51(10) (2008) 491-495. | Excluded | Title and abstract | Irrelevant |
| 312] Nct, Dysfunction of Nutritive Blood Flow as a Determinant of Anabolic Resistance in Older People, https://clinicaltrials.gov/show/NCT01734616 (2012). | Excluded | Title and abstract | Irrelevant |
| 313] Nct, Early Intervention in Infants With Cerebral Palsy, https://clinicaltrials.gov/show/NCT04250454 (2020). | Excluded | Title and abstract | Irrelevant |
| 314] Y. Yang, C. Wang, Y. Xiang, J. Lu, T. Penzel, Editorial: Mental Disorders Associated With Neurological Diseases, Frontiers in Psychiatry 11 (2020). | Excluded | Title and abstract | Irrelevant |
| 315] Isrctn, Effect of 25-hydroxy vitamin D on inflammation and bone-turnover in critically ill patients, https://trialsearch.who.int/Trial2.aspx?TrialID=ISRCTN24385496 (2009). | Excluded | Title and abstract | Irrelevant |
| 316] J.V. Soriano, T.S. Gomez, M.B. Martinez, P.L. Casanova, J.A. Bellon, J.M.C. Herrero, E.D. Lupianez, F. De Haro Fernandez, E.P. Perez, J.R. Lopez, et al., Effect of a specific nutritional supplement (Balnimax®) on lower extremity ulcers of venous etiology and pressure ulcers, Gerokomos 27(1) (2016) 27‐32. | Excluded | Title and abstract | Irrelevant |
| 317] A.V. Rawlings, T.J. Stephens, J.H. Herndon, M. Miller, Y. Liu, K. Lombard, The effect of a vitamin A palmitate and antioxidant-containing oil-based moisturizer on photodamaged skin of several body sites, Journal of cosmetic dermatology 12(1) (2013) 25‐35. | Excluded | Title and abstract | Irrelevant |
| 318] Nct, Effect of Aerobic Exercise and Diet on Anthropometric and Hematological Measurements in Obese Anemic Women, https://clinicaltrials.gov/show/NCT05165680 (2021). | Excluded | Title and abstract | Irrelevant |
| 319] Nct, Effect of Allopurinol Administration on the Prevention of Muscle Mass Loss in Subject Immobilized, https://clinicaltrials.gov/show/NCT01987570 (2013). | Excluded | Title and abstract | Irrelevant |
| 320] Isrctn, Effect of an antioxidant cosmetic skin cream on healthy participants, https://trialsearch.who.int/Trial2.aspx?TrialID=ISRCTN49855247 (2016). | Excluded | Title and abstract | Irrelevant |
| 321] I. Sadowska-Bartosz, G. Bartosz, Effect of Antioxidants on the Fibroblast Replicative Lifespan in Vitro, Oxidative Medicine and Cellular Longevity 2020 (2020). | Excluded | Title and abstract | Irrelevant |
| 322] Actrn, Effect of blackcurrant consumption on physical fitness, bioenergetics and natural immunity during high intensity training; involvement of mitochondrial adaptation?, http://www.who.int/trialsearch/Trial2.aspx?TrialID=ACTRN12614000019606 (2014). | Excluded | Title and abstract | Irrelevant |
| 323] O. Ekinci, S. Yanik, B. Terzioʇlu, E. Yilmaz Akyüz, A. Dokuyucu, S. Erdem, The effect of calcium B-hydroxy-B-methylbutyrate, vitamin D and protein supplementation on postoperative immobilization in elderly malnourished patients with hip fracture: a randomized controlled study, Clinical nutrition (Edinburgh, Scotland) 34 (2015) S102. | Excluded | Title and abstract | Irrelevant |
| 324] R.K. Campbell, K.M. Hurley, A.A. Shamim, S. Shaikh, Z.T. Chowdhury, S. Mehra, S. De Pee, T. Ahmed, K.P. West, P. Christian, Effect of complementary food supplementation on breastfeeding and home diet in rural Bangladeshi children, American journal of clinical nutrition 104(5) (2016) 1450‐1458. | Excluded | Title and abstract | Irrelevant |
| 325] Actrn, Effect of Cosmos caudatus Extract Supplementation on Various Health Related Parameters among Older Adults with Muscle Loss, https://trialsearch.who.int/Trial2.aspx?TrialID=ACTRN12623000046606 (2023). | Excluded | Title and abstract | Irrelevant |
| 326] S. Chakraborty, R.Y. Sathe, J.H. Chormale, A. Dangi, P.V. Bharatam, A.K. Bansal, Effect of Deep Eutectic System (DES) on Oral Bioavailability of Celecoxib: In Silico, In Vitro, and In Vivo Study, Pharmaceutics 15(9) (2023). | Excluded | Title and abstract | Irrelevant |
| 327] H. Selye, E. Bajusz, Effect of denervation on experimentally induced changes in the growth of bone and muscle, The American journal of physiology 192(2) (1958) 297-300. | Excluded | Title and abstract | Irrelevant |
| 328] J. Ruiz Esparza Cisneros, J.J. Vasconcelos-Ulloa, D. González-Mendoza, G. Beltrán-González, R. Díaz-Molina, Effect of dietary intervention with a legume-based food product on malondialdehyde levels, HOMA index, and lipid profile, Endocrinologia, diabetes y nutricion 67(4) (2020) 235‐244. | Excluded | Title and abstract | Irrelevant |
| 329] Isrctn, Effect of dietary nitrate ingestion in stable angina, https://trialsearch.who.int/Trial2.aspx?TrialID=ISRCTN72085021 (2015). | Excluded | Title and abstract | Irrelevant |
| 330] S. Newman, S. Leeson, The effect of dietary supplementation with 1,25-dihydroxycholecalciferol or vitamin C on the characteristics of the tibia of older laying hens, Poultry Science 78(1) (1999) 85-90. | Excluded | Title and abstract | Irrelevant |
| 331] A.C. Ogbonna, A.S. Chaudhry, L. Asher, Effect of dietary vitamin D3 and ultraviolet-B light on the behaviour and growth of broilers challenged with social isolation stress, Applied Animal Behaviour Science 268 (2023). | Excluded | Title and abstract | Irrelevant |
| 332] E. Dzendolet, EFFECT OF DRAMAMINE ON THE OBJECTIVE ELECTRICAL VESTIBULOGRAM, Perceptual and motor skills 18 (1964) 465-8. | Excluded | Title and abstract | Irrelevant |
| 333] T. Saito, Y. Mori, O. Irei, K. Baba, S. Nakajo, E. Itoi, Effect of eldecalcitol on muscle function and fall prevention in Japanese postmenopausal women: a randomized controlled trial, Journal of orthopaedic science 26(1) (2021) 173‐178. | Excluded | Title and abstract | Irrelevant |
| 334] R. Williams, R.M. Lin, G.J. Huang, H.A. Tran, M.A. Poles, F. Francois, The effect of fiber, folate, and exercise on the risk for colon polyps in a multiethnic colon cancer screening population, Gastroenterology 128(4) (2005) A299-A299. | Excluded | Title and abstract | Irrelevant |
| 335] Nct, Effect of Folic Acid Supplementation in Pregnant Women Having Thalassaemia Trait, https://clinicaltrials.gov/show/NCT04310059 (2020). | Excluded | Title and abstract | Irrelevant |
| 336] S. Stratton, A. Gerritsen, C. Loos, K. Urschel, Effect of forage type and ration balancer protein content on measures of whole-body protein metabolism in growing horses consuming a predominantly forage diet, Journal of equine veterinary science 76 (2019) 76. | Excluded | Title and abstract | Irrelevant |
| 337] D.L. Hsu, EFFECT OF GERMINATION ON FUNCTIONAL AND NUTRITIONAL PROPERTIES OF DRY PEAS, LENTILS, AND FABA BEANS, 1981. | Excluded | Title and abstract | Irrelevant |
| 338] Irct20090822002365N, The effect of Gut Makeover diet with or without yoga on weight management, https://trialsearch.who.int/Trial2.aspx?TrialID=IRCT20090822002365N24 (2020). | Excluded | Title and abstract | Irrelevant |
| 339] R.H. Hartman, G. Matrone, G.H. Wise, Effect of high dietary manganese on hemoglobin formation, The Journal of nutrition 57(3) (1955) 429-39. | Excluded | Title and abstract | Irrelevant |
| 340] Irct138712101720N, Effect of L-arginine and selenium supplementation on metabolic syndrome components in obese women with hypocaloric diet enriched in legumes, https://trialsearch.who.int/Trial2.aspx?TrialID=IRCT138712101720N1 (2009). | Excluded | Title and abstract | Irrelevant |
| 341] D.K. Andrews, W.D. Berry, J. Brake, Effect of lighting program and nutrition on feather replacement of molted single comb White Leghorn hens, Poultry science 66(10) (1987) 1635-9. | Excluded | Title and abstract | Irrelevant |
| 342] P.D. Neves, F.G. Graciolli, I.B. Oliveira, R.A. Bridi, R.M. Moysés, R.M. Elias, Effect of Mineral and Bone Metabolism on Restless Legs Syndrome in Hemodialysis Patients, Journal of clinical sleep medicine : JCSM : official publication of the American Academy of Sleep Medicine 13(1) (2017) 89-94. | Included | Full text | Relationship between vitamins and RLS |
| 343] J. Brown, E. Merritt, C.N. Mowa, S. McAnulty, Effect of moringa oleifera on bone density in post-menopausal women, FASEB journal 30(no pagination) (2016). | Excluded | Title and abstract | Irrelevant |
| 344] G. Gennarelli, C. Paschero, S. Canosa, C. Benedetto, A. Revelli, Effect of Myo-Inositol and Alpha-Lipoic Acid on oocyte morphology and embryo morphokinetics: a prospective preliminary analysis of 40 overweight patients undergoing ICSI treatment, Human reproduction (Oxford, England) 35(SUPPL 1) (2020) i256. | Excluded | Title and abstract | Irrelevant |
| 345] G. Otoo, Y. Adam, Effect of nutrition education with an emphasis on consumption of iron-rich foods on hemoglobin levels of pregnant women in Ghana, FASEB journal 30 (2016). | Excluded | Title and abstract | Irrelevant |
| 346] Umin, Effect of oral ingestion of Vitamin D3 on lower limb function, bone markers, blood pressure, indicators of the innate immunity system and QOL in post-menopausal Japanese women, https://trialsearch.who.int/Trial2.aspx?TrialID=JPRN-UMIN000019304 (2015). | Excluded | Title and abstract | Irrelevant |
| 347] Nct, Effect of Parathyroidectomy on Sleep, https://clinicaltrials.gov/show/NCT00501215 (2007). | Excluded | Title and abstract | Irrelevant |
| 348] S. Macher, C. Herster, M. Holter, M. Moritz, E.M. Matzhold, T. Stojakovic, T.R. Pieber, P. Schlenke, C. Drexler, K. Amrein, The effect of parenteral or oral iron supplementation on fatigue, sleep, quality of life and restless legs syndrome in iron-deficient blood donors: A secondary analysis of the ironwoman rct, Nutrients 12(5) (2020). | Excluded | Title and abstract | Irrelevant |
| 349] Irct20170501033743N, Effect of physical activity on postpartum health, https://trialsearch.who.int/Trial2.aspx?TrialID=IRCT20170501033743N3 (2021). | Excluded | Title and abstract | Irrelevant |
| 350] Nct, Effect of Postoperative Oral Carbohydrate Administration in Total Keen Arthroplasty Elderly Patients, https://clinicaltrials.gov/show/NCT05603364 (2022). | Excluded | Title and abstract | Irrelevant |
| 351] Nct, The Effect of Progressive Muscle Relaxation Exercise Training Given to Pregnant Women With Restless Leg Syndrome, https://clinicaltrials.gov/show/NCT04853771 (2021). | Excluded | Title and abstract | Irrelevant |
| 352] N. Corsico, A. Nardone, M.R. Lucreziotti, L.G. Spagnoli, D. Pesce, T. Aureli, M.E. Di Cocco, A. Miccheli, F. Conti, E. Arrigoni Martelli, Effect of propionyl-L-carnitine in a rat model of peripheral arteriopathy: a functional, histologic, and NMR spectroscopic study, Cardiovascular drugs and therapy 7(2) (1993) 241-51. | Excluded | Title and abstract | Irrelevant |
| 353] D. Michalská, J.J. Stepan, B.R. Basson, I. Pavo, The effect of raloxifene after discontinuation of long-term alendronate treatment of postmenopausal osteoporosis, Journal of clinical endocrinology and metabolism 91(3) (2006) 870‐877. | Excluded | Title and abstract | Irrelevant |
| 354] J.L. Liu, H.M. Zhu, Q.R. Huang, Z.L. Zhang, H.L. Li, Y.J. Qin, Y. Zhang, D.L. Wei, J.H. Lu, H. Liu, et al., Effect of raloxifene hydrochloride on bone mineral density, bone metabolism and serum lipids in Chinese postmenopausal women with osteoporosis, Zhonghua yi xue za zhi 84(4) (2004) 269‐273. | Excluded | Title and abstract | Irrelevant |
| 355] Irct138811103226N, The effect of reflexology on premenstrual syndrome, https://trialsearch.who.int/Trial2.aspx?TrialID=IRCT138811103226N1 (2012). | Excluded | Title and abstract | Irrelevant |
| 356] J. Huang, Y. Chen, B. Dong, W. Kong, J. Zhang, W. Xue, D. Liu, Y. Huang, Effect of remote ischaemic preconditioning on renal protection in patients undergoing laparoscopic partial nephrectomy: a 'blinded' randomised controlled trial, BJU international 112(1) (2013) 74‐80. | Excluded | Title and abstract | Irrelevant |
| 357] M.W. Esch, R.A. Easter, J.M. Bahr, Effect of riboflavin deficiency on estrous cyclicity in pigs, Biology of reproduction 25(3) (1981) 659-65. | Excluded | Title and abstract | Irrelevant |
| 358] J. Kalra, K. Vishwakarma, T. Sharma, D.C. Dhasmana, R. Gupta, Effect of Ropinirole and Bupropion on sleep in patients with restless legs syndrome, Indian journal of physiology and pharmacology 57(5) (2013) 146. | Excluded | Title and abstract | Irrelevant |
| 359] C. James, J. Wiseman, L. Asher, The effect of supplementary ultraviolet wavelengths on the performance of broiler chickens, Poultry Science 99(11) (2020) 5517-5525. | Excluded | Title and abstract | Irrelevant |
| 360] S. Kumar, G. Sahni, H.K. Singh Chawla, D. Singh, Effect of teriparatide in fracture healing in lower limbs - An interventional study, Journal of clinical and diagnostic research 15(4) (2021) RC01‐RC04. | Excluded | Title and abstract | Irrelevant |
| 361] R.B.R. jt, Effect of the use of L-Arginine, Caffeine or Creatine supplements associated with physical activity in women with Polycystic Ovary Syndrome with Metabolic Syndrome, https://trialsearch.who.int/Trial2.aspx?TrialID=RBR-9832jt (2018). | Excluded | Title and abstract | Irrelevant |
| 362] Irct2016082129463N, The effect of treatment with vitamin E compared with relaxation in the treatment of restless legs syndrome (RLS) in HD patients, https://trialsearch.who.int/Trial2.aspx?TrialID=IRCT2016082129463N1 (2016). | Excluded | Title and abstract | Irrelevant |
| 363] Nct, The Effect of Very Low Calorie Diet With and Without Exercise on Muscle Synthesis in Middle-aged Overweight Male, https://clinicaltrials.gov/show/NCT03116256 (2017). | Excluded | Title and abstract | Irrelevant |
| 364] Nct, The Effect of Vitamin C and E Therapy on Restless Leg Syndrome in Patients With End Stage Renal Disease on Haemodialysis, https://clinicaltrials.gov/show/NCT05350124 (2022). | Excluded | Title and abstract | Irrelevant |
| 365] Ctri, The Effect of Vitamin C on Post-Operative opioid Requirement in orthopaedic lower limb surgery, https://trialsearch.who.int/Trial2.aspx?TrialID=CTRI/2018/04/013474 (2018). | Excluded | Title and abstract | Irrelevant |
| 366] M. Khassaf, A. McArdle, C. Esanu, A. Vasilaki, F. McArdle, R.D. Griffiths, D.A. Brodie, M.J. Jackson, Effect of vitamin C supplements on antioxidant defence and stress proteins in human lymphocytes and skeletal muscle, Journal of physiology 549(Pt 2) (2003) 645‐652. | Excluded | Title and abstract | Irrelevant |
| 367] Irct20190126042496N, Effect of vitamin D in enhanced recovery after brain surgery, http://www.who.int/trialsearch/Trial2.aspx?TrialID=IRCT20190126042496N1 (2019). | Excluded | Title and abstract | Irrelevant |
| 368] M. Tutuncu, M. Tutuncu, The effect of vitamin D on restless legs syndrome: prospective self-controlled case study, Sleep & breathing = Schlaf & Atmung 24(3) (2020) 1101-1106. | Included | Full text | Relationship between vitamins and RLS |
| 369] S. Wali, B. Abalkhail, K. Aljammali, H. Sabbahi, B. Alotaiby, The effect of vitamin d replacement therapy in restless legs syndrome: a randomized trial, Chest 152(4) (2017) A1069. | Included | Full text | Vetamin therapy for RLS |
| 370] S. Wali, A. Shukr, A. Boudal, A. Alsaiari, A. Krayem, The effect of vitamin D supplements on the severity of restless legs syndrome, Sleep and Breathing (2014). | Excluded | Title and abstract | Duplication |
| 371] S. Wali, A. Shukr, A. Boudal, A. Alsaiari, A. Krayem, The effect of vitamin D supplements on the severity of restless legs syndrome, Sleep & breathing = Schlaf & Atmung 19(2) (2015) 579-83. | Excluded | Full text | Without control |
| 372] S. Wali, A. Shukr, A. Boudal, A. Alsaiari, A. Krayem, The effect of vitamin D supplements on the severity of restless legs syndrome (vol 19, pg 579, 2015), Sleep and Breathing 19(4) (2015) 1483-1483. | Excluded | Title and abstract | Duplication |
| 373] E. Arató, M. Kürthy, L. Sínay, G. Kasza, G. Menyhei, P. Hardi, S. Masoud, K. Ripp, K. Szilágyi, I. Takács, et al., Effect of vitamin E on reperfusion injuries during reconstructive vascular operations on lower limbs, Clinical hemorheology and microcirculation 44(2) (2010) 125‐136. | Excluded | Title and abstract | Irrelevant |
| 374] Nct, Effect of Vitamin K2 in the Treatment of Nocturnal Leg Cramps in the Elderly, https://clinicaltrials.gov/show/NCT05547750 (2022). | Excluded | Title and abstract | Irrelevant |
| 375] Nct, Effect of Whole Body Electromyostimulation and/or Protein Supplementation on Sarcopenic Obesity in 70+, https://clinicaltrials.gov/show/NCT02857660 (2016). | Excluded | Title and abstract | Irrelevant |
| 376] T. Higuchi, T. Ueno, S. Uchiyama, S. Matsuki, M. Ogawa, K. Takamatsu, Effect of γ-tocopherol supplementation on premenstrual symptoms and natriuresis: a randomized, double-blind, placebo-controlled study, BMC complementary medicine and therapies 23(1) (2023) 136. | Excluded | Title and abstract | Irrelevant |
| 377] S. Kapoor, Effective treatment of restless legs syndrome in patients on haemodialysis, Nephrology 18(1) (2013) 78. | Excluded | Title and abstract | Irrelevant |
| 378] K.V. Chang, W.T. Wu, K.C. Huang, D.S. Han, Effectiveness of early versus delayed exercise and nutritional intervention on segmental body composition of sarcopenic elders - A randomized controlled trial, Clinical nutrition (Edinburgh, Scotland) 40(3) (2021) 1052‐1059. | Excluded | Title and abstract | Irrelevant |
| 379] Actrn, Effectiveness of Multicomponent Physical Exercise in old adults after hospitalization: short vs long supervised programs, https://trialsearch.who.int/Trial2.aspx?TrialID=ACTRN12619000093189 (2019). | Excluded | Title and abstract | Irrelevant |
| 380] A. Cataldi, V. Gasbarro, R. Viaggi, R. Soverini, E. Gresta, F. Mascoli, Effectiveness of the association of alphatocopherol, rutin, melilotus, and centella asiatica in the treatment of patients affected by chronic venous insufficiency, Minerva cardioangiologica 49(2) (2001) 159‐163. | Excluded | Title and abstract | Irrelevant |
| 381] Q. Sun, V.W. Lou, A. Dai, C. To, S.Y. Wong, The Effectiveness of the Young–Old Link and Growth Intergenerational Program in Reducing Age Stereotypes, Research on social work practice 29(5) (2019) 519‐528. | Excluded | Title and abstract | Irrelevant |
| 382] A. Sedin, M. Landin Olsson, L. Cloetens, The effects of 2-year consumption of a new Nordic diet on weight loss maintenance in subjects with obesity: a randomised controlled intervention study, Obesity reviews 21(SUPPL 1) (2020). | Excluded | Title and abstract | Irrelevant |
| 383] A.H. Haghighi, M. Shojaee, R. Askari, S. Abbasian, P. Gentil, The effects of 12 weeks resistance training and vitamin D administration on neuromuscular joint, muscle strength and power in postmenopausal women, Physiology & behavior (2023) 114419. | Excluded | Title and abstract | Irrelevant |
| 384] Actrn, Effects of 12-week dairy-derived nutritional supplementation combined with physical activity on health and physical function in healthy middle aged women after a 2-week period of reduced activity, http://www.who.int/trialsearch/Trial2.aspx?TrialID=ACTRN12616001714471 (2016). | Excluded | Title and abstract | Irrelevant |
| 385] T.H. Suominen, J. Edgren, A. Salpakoski, M. Arkela, M. Kallinen, T. Cervinka, T. Rantalainen, T. Tormakangas, A. Heinonen, S. Sipila, Effects of a Home-Based Physical Rehabilitation Program on Tibial Bone Structure, Density, and Strength After Hip Fracture: a Secondary Analysis of a Randomized Controlled Trial, JBMR plus 3(6) (2019). | Excluded | Title and abstract | Irrelevant |
| 386] M.S. Koozehchian, S.V. Hosseini, H. Eynavi, G. Mabrey, G. Owlia, J. Chandler, Effects of Acute Preworkout Supplement Ingestion on Hemodynamic Responses, Cognitive Function, and Exercise Performance in Resistance-Trained Males, FASEB journal 34(SUPPL 1) (2020). | Excluded | Title and abstract | Irrelevant |
| 387] R.R. Hacker, M.P. Stefanovic, T.R. Batra, Effects of cold exposure on growing pigs: growth, body composition and 17-ketosteroids, Journal of animal science 37(3) (1973) 739-44. | Excluded | Title and abstract | Irrelevant |
| 388] Nct, Effects of Continuous Versus Single Dose Spinal Anesthesia in Octagenerians Undergoing Hip Surgery, https://clinicaltrials.gov/show/NCT05418374 (2022). | Excluded | Title and abstract | Irrelevant |
| 389] G. Aynaci, Z. Guksu, The effects of dietary folate and iron supplementation on restless legs and preeclampsia in pregnancy, Progress in Nutrition 21(2) (2019) 398-405. | Included | Full text | Relationship between vitamins and RLS |
| 390] R.B.R. bqsw, Effects of different physical activities programs on osteoporosis related-variables in older women, https://trialsearch.who.int/Trial2.aspx?TrialID=RBR-6bqsw8 (2015). | Excluded | Title and abstract | Irrelevant |
| 391] S. Oesen, B. Halper, M. Hofmann, W. Jandrasits, B. Franzke, E.M. Strasser, A. Graf, H. Tschan, N. Bachl, M. Quittan, et al., Effects of elastic band resistance training and nutritional supplementation on physical performance of institutionalised elderly--A randomized controlled trial, Experimental gerontology 72 (2015) 99‐108. | Excluded | Title and abstract | Irrelevant |
| 392] Nct, Effects of ElevATP on Body Composition and Athletic Performance, https://clinicaltrials.gov/show/NCT02819219 (2016). | Excluded | Title and abstract | Irrelevant |
| 393] W. Kemmler, M. Kohl, M. Frohlich, K. Engelke, S. von Stengel, D. Schoene, Effects of High-Intensity Resistance Training on Fitness and Fatness in Older Men With Osteosarcopenia, Frontiers in physiology 11 (2020). | Excluded | Title and abstract | Irrelevant |
| 394] W. Kemmler, M. Kohl, M. Fröhlich, F. Jakob, K. Engelke, S. von Stengel, D. Schoene, Effects of High-Intensity Resistance Training on Osteopenia and Sarcopenia Parameters in Older Men with Osteosarcopenia-One-Year Results of the Randomized Controlled Franconian Osteopenia and Sarcopenia Trial (FrOST), Journal of bone and mineral research 35(9) (2020) 1634‐1644. | Excluded | Title and abstract | Irrelevant |
| 395] Nct, Effects of Increased Greek Yogurt Consumption in Youth and Young Adult Athletes, https://clinicaltrials.gov/show/NCT05922462 (2023). | Excluded | Title and abstract | Irrelevant |
| 396] P.J. De Kam, W.L. Luo, L. Wenning, L. Ratcliffe, C.M. Sisk, J. Royalty, W. Radziszewski, J.A. Wagner, E. Lai, The effects of laropiprant on the antiplatelet activity of co-administered clopidogrel and aspirin, Platelets 25(7) (2014) 480‐487. | Excluded | Title and abstract | Irrelevant |
| 397] F. Akharume, K. Singh, L. Sivanandan, Effects of liquid smoke infusion on osmotic dehydration kinetics and microstructural characteristics of apple cubes, Journal of Food Engineering 246 (2019) 51-57. | Excluded | Title and abstract | Irrelevant |
| 398] J.N. Zou, X.J. Jin, Y.X. Zhang, C.Y. Ren, M.C. Zhang, M.X. Wang, Effects of melatonin on photosynthesis and soybean seed growth during grain filling under drought stress, Photosynthetica 57(2) (2019) 512-520. | Excluded | Title and abstract | Irrelevant |
| 399] S.H. Moon, H.M. Park, C.S. Shin, H.Y. Chung, Y.S. Chung, B.K. Yoon, J.S. Chang, M.I. Kang, J.Y. Park, H.K. Yoon, Effects of monthly risedronate with cholecalciferol on 25-hydroxyvitamin d level and bone markers in Korean patients with osteoporosis, Journal of bone and mineral research 26 (2011). | Excluded | Title and abstract | Irrelevant |
| 400] R.A. Dossa, E.A. Ategbo, J.M. van Raaij, C. de Graaf, J.G. Hautvast, Effects of multivitamin-multimineral supplementation on appetite of stunted young Beninese children, Appetite 39(2) (2002) 111‐117. | Excluded | Title and abstract | Irrelevant |
| 401] Nct, Effects of Niacin on Good Cholesterol in People With Peripheral Arterial Disease, https://clinicaltrials.gov/show/NCT01391377 (2011). | Excluded | Title and abstract | Irrelevant |
| 402] Z. Gunes, The Effects of Nutrition on Sleep and Sleep Complaints among Elderly Persons, 2015. | Excluded | Title and abstract | Irrelevant |
| 403] S. Navid, M. Hilmi, A.Q. Sazili, A. Sheikhlar, Effects of Papaya Leaf Meal, Pineapple Skin Meal and Vitamin D<sub>3</sub> Supplementation on Meat Quality of Spent Layer Chicken, Journal of Animal and Veterinary Advances 9(22) (2010) 2873-2876. | Excluded | Title and abstract | Irrelevant |
| 404] D. Young, P.Y. Ng, D. Cheng, L.C. Hong, Effects of Physical Activity Intervention for Chinese People With Severe Mental Illness, Research on social work practice 29(7) (2019) 796‐807. | Excluded | Title and abstract | Irrelevant |
| 405] R.B.R. ykyws, Effects of physical exercise and vitamin D supplementation on muscle mass of hemodialysis patients, https://trialsearch.who.int/Trial2.aspx?TrialID=RBR-8ykyws (2015). | Excluded | Title and abstract | Irrelevant |
| 406] Nct, The Effects of Post-Conditioning and Administration of Vitamin C on Intramuscular High Energy Phosphate Levels, https://clinicaltrials.gov/show/NCT00534924 (2007). | Excluded | Title and abstract | Irrelevant |
| 407] Nct, Effects of Pulses Through the Gut Microbiome and Bioavailability of Bioactive Compounds, https://clinicaltrials.gov/ct2/show/NCT05999136 (2023). | Excluded | Title and abstract | Irrelevant |
| 408] Nct, Effects of Replacing Red Meat With Legumes on Biomarkers of Chronic Diseases in Healthy Men (Leg4Life), https://clinicaltrials.gov/show/NCT04599920 (2020). | Excluded | Title and abstract | Irrelevant |
| 409] T. Pimrat, P. Arpaporn, L. Sunee, V. Paranee, S. Sarah Anne, Effects of School-Based Participation Program to Prevent Multiple Risk Behaviors in Thai Male Adolescents, Pacific Rim international journal of nursing research 23(3) (2019) 228‐242. | Excluded | Title and abstract | Irrelevant |
| 410] M. Leeman, R.P.M. Gadiot, J.M.A. Wijnand, E. Birnie, J.A. Apers, L.U. Biter, M. Dunkelgrun, Effects of standard v. very long Roux limb Roux-en-Y gastric bypass on nutrient status: a 1-year follow-up report from the Dutch Common Channel Trial (DUCATI) Study, British journal of nutrition 123(12) (2020) 1434‐1440. | Excluded | Title and abstract | Irrelevant |
| 411] M. Drey, A. Zech, E. Freiberger, T. Bertsch, W. Uter, C.C. Sieber, K. Pfeifer, J.M. Bauer, Effects of Strength Training versus Power Training on Physical Performance in Prefrail Community-Dwelling Older Adults, Gerontology 58(3) (2012) 197-204. | Excluded | Title and abstract | Irrelevant |
| 412] Kct, Effects of the strengthening exercise to bone marrow density, muscle mass and quality in patients with osteosarcopenia, https://trialsearch.who.int/Trial2.aspx?TrialID=KCT0008291 (2023). | Excluded | Title and abstract | Irrelevant |
| 413] K. Zhu, H. Greenfield, X. Du, Q. Zhang, G. Ma, X. Hu, C.T. Cowell, D.R. Fraser, Effects of two years' milk supplementation on size-corrected bone mineral density of Chinese girls, Asia Pacific journal of clinical nutrition 17 Suppl 1 (2008) 147‐150. | Excluded | Title and abstract | Irrelevant |
| 414] Nct, Effects of Vitamin D on Skeletal Muscle Strength in Resistance Trained Adult Females, https://clinicaltrials.gov/show/NCT05489666 (2022). | Excluded | Title and abstract | Irrelevant |
| 415] Actrn, Effects of vitamin D supplementation in physically active adults, https://trialsearch.who.int/Trial2.aspx?TrialID=ACTRN12620000896976 (2020). | Excluded | Title and abstract | Irrelevant |
| 416] R. Daly, S. Pirotta, D. Kidgell, Effects of vitamin D supplementation on neural plasticity, serum brain-derived neurotrophic factor (bdnf) and functional performance in older adults: a 10-week double-blinded, placebo controlled randomised trial, Journal of bone and mineral research 28 (2013). | Excluded | Title and abstract | Irrelevant |
| 417] G.L. Close, J. Leckey, M. Patterson, W. Bradley, D.J. Owens, W.D. Fraser, J.P. Morton, The effects of vitamin D(3) supplementation on serum total 25 OH]D concentration and physical performance: a randomised dose-response study, British journal of sports medicine 47(11) (2013) 692‐696. | Excluded | Title and abstract | Irrelevant |
| 418] Ctri, Efficacy and Safety of Long-Term (6 Months) InnohepÂ® Treatment Versus Anticoagulation with a Vitamin K Antagonist (Warfarin) for the Treatment of Acute Venous Thromboembolism in Cancer Patients / IN 0901 INT, https://trialsearch.who.int/Trial2.aspx?TrialID=CTRI/2010/091/000598 (2010). | Excluded | Title and abstract | Irrelevant |
| 419] Z. Yueyue, S. Xiaoxia, L. Dexue, L. Ruige, Efficacy and safety of vitamin D2 supplementation on diabetic peripheral neuropathy: a multicentre, randomised, double-blind trial, The lancet diabetes and endocrinology 4(SPEC. ISSUE 3) (2016) S29. | Excluded | Title and abstract | Irrelevant |
| 420] Ctri, Efficacy and Safety of WellmuneÂ® Capsules in Diabetes Mellitus Patients, https://trialsearch.who.int/Trial2.aspx?TrialID=CTRI/2022/05/042729 (2022). | Excluded | Title and abstract | Irrelevant |
| 421] B. Zederfeldt, I. Borg, K. Haeger, Efficacy and tolerance of Flunixin (SCH 14714) in the treatment of postoperative pain, with observations on the methodology of postoperative pain studies, British journal of anaesthesia 49(5) (1977) 467‐471. | Excluded | Title and abstract | Irrelevant |
| 422] E. Soltanimehr, E. Bahrampour, Z. Yousefvand, Efficacy of diode and CO2 lasers along with calcium and fluoride-containing compounds for the remineralization of primary teeth, BMC oral health 19(1) (2019) N.PAG. | Excluded | Title and abstract | Irrelevant |
| 423] Nct, EFFICACY OF MORUS ALBA FRUIT EXTRACTS AND CHLORHEXIDINE ON SALIVARY STREPTOCOCCUS MUTANS AND pH LEVELS, https://clinicaltrials.gov/show/NCT05052775 (2021). | Excluded | Title and abstract | Irrelevant |
| 424] Nct, Efficacy of Prebiotic and Probiotic Dietary Modulation in Schizophrenic Disorders, https://clinicaltrials.gov/show/NCT04366401 (2020). | Excluded | Title and abstract | Irrelevant |
| 425] T. Nakamura, T. Matsuo, T. Fukuda, S. Yamato, K. Yamaguchi, I. Kinoshita, T. Matsuzaki, Y. Nishiura, K. Nagasato, T. Narita-Masuda, H. Nakamura, K. Satoh, H. Sasaki, H. Sakai, A. Kawakami, Efficacy of prosultiamine treatment in patients with human T lymphotropic virus type I-associated myelopathy/tropical spastic paraparesis: results from an open-label clinical trial, BMC medicine 11 (2013). | Excluded | Title and abstract | Irrelevant |
| 426] D.C. Mahan, L.H. Penhale, J.H. Cline, A.L. Moxon, A.W. Fetter, J.T. Yarrington, Efficacy of supplemental selenium in reproductive diets on sow and progeny performance, Journal of animal science 39(3) (1974) 536-43. | Excluded | Title and abstract | Irrelevant |
| 427] S.O. Wali, B. Abaalkhail, F. Alhejaili, S.R. Pandi-Perumal, Efficacy of vitamin D replacement therapy in restless legs syndrome: a randomized control trial, Sleep and breathing (no pagination) (2018). | Excluded | Title and abstract | Irrelevant |
| 428] S.O. Wali, B. Abaalkhail, F. Alhejaili, S.R. Pandi-Perumal, Efficacy of vitamin D replacement therapy in restless legs syndrome: a randomized control trial, Sleep & breathing = Schlaf & Atmung 23(2) (2019) 595-601. | Excluded | Title and abstract | Irrelevant |
| 429] K. Alimoradi, B. Nikooyeh, A.A. Ravasi, M. Zahedirad, N. Shariatzadeh, A. Kalayi, T.R. Neyestani, Efficacy of vitamin D supplementation in physical performance of Iranian elite athletes, International journal of preventive medicine 10(1) (2019). | Excluded | Title and abstract | Irrelevant |
| 430] M.K. Fallahzadeh, H. Akbari, S. Sohrabi Nazari, M.M. Sagheb, Efficacy of vitamins C, E and their combination for treatment of restless legs syndrome in hemodialysis patients; A randomized, double-blind, placebo-controlled trial, Iranian journal of kidney diseases 5 (2011) 22‐23. | Excluded | Full text | Duplication |
| 431] M.M. Sagheb, B. Dormanesh, M.K. Fallahzadeh, H. Akbari, S. Sohrabi Nazari, S.T. Heydari, S. Behzadi, Efficacy of vitamins C, E, and their combination for treatment of restless legs syndrome in hemodialysis patients: a randomized, double-blind, placebo-controlled trial, Sleep medicine 13(5) (2012) 542-5. | Included | Full text | Vetamin therapy for RLS |
| 432] L. Seefried, F. Genest, J. Baumann, A. Heidemeier, R. Meffert, F. Jakob, Efficacy of Zoledronic Acid in the Treatment of Nonmalignant Painful Bone Marrow Lesions: a Triple-Blind, Randomized, Placebo-Controlled Phase III Clinical Trial (ZoMARS), Journal of bone and mineral research 37(3) (2022) 420‐427. | Excluded | Title and abstract | Irrelevant |
| 433] E.S. Euctr, EFFICIENCY AND SAFETY OF CLINAVIT PAEDIATRIC AT A WEIGHT DEPENDANT DOSE ADMINISTERED DAILY FOR 5 CONTINUOUS DAYS ON 4 VITAMINS BLOOD LEVEL IN NEONATES AND CHILDREN UP TO 11 YEARS OF AGE RECEIVING PARENTERAL NUTRITION. A PROSPECTIVE, MULTICENTRE, RANDOMISED, COMPARATIVE, DOUBLE BLIND PHASE III STUDY IN THERAPEUTIC USE VERSUS SOLUVIT AND VITALIPID INFANT CARRIED OUT IN PARALLEL GROUPS. EFICACIA Y SEGURIDAD DE CLINAVIT PEDIÁTRICO, A UNA DOSIS DEPENDIENTE DEL PESO, ADMINISTRADO A DIARIO DURANTE 5 DÍAS CONSECUTIVOS, MEDIDAS POR LA CONCENTRACIÓN SANGUÍNEA DE 4 VITAMINAS EN NEONATOS Y NIÑOS DE HASTA 11 AÑOS DE EDAD TRATADOS CON NUTRICIÓN PARENTERAL. ESTUDIO EN FASE III PROSPECTIVO, MULTICÉNTRICO, ALEATORIZADO, COMPARATIVO, ABIERTO Y DE GRUPOS PARALELOS EN USO TERAPÉUTICO FRENTE A SOLUVIT Y VITALIPID INFANT, https://trialsearch.who.int/Trial2.aspx?TrialID=EUCTR2004-001569-16-ES (2006). | Excluded | Title and abstract | Irrelevant |
| 434] M. Olari, G. Le Vacon, M. Follet, Ekbom syndrome - Cultural aspects from a clinical case, European Psychiatry 26 (2011). | Excluded | Title and abstract | Irrelevant |
| 435] S. Nascimento, H. Simião, T. Mendonça, M. Silva, Ekbom syndrome -a case report, European Psychiatry 64 (2021) S538-S539. | Excluded | Title and abstract | Irrelevant |
| 436] D. Paiva Pajares, C. Hernández Peláez, A. Martínez Muelas, M. López Isern, T. Castelló Pons, Ekbom's syndrome in an HIV man: a case report, European Psychiatry 65 (2022) S589. | Excluded | Title and abstract | Irrelevant |
| 437] P.T. Sobash, K. Vedala, C.M. McClain, C. Oster, Electrolyte Replacement in Bartter Syndrome With Abnormal Small Bowel: A Case Report, Journal of Investigative Medicine High Impact Case Reports 8 (2020). | Excluded | Title and abstract | Irrelevant |
| 438] W. Djoenaidi, S.L. Notermans, Electrophysiologic evaluation of beri-beri polyneuropathy, Electromyography and clinical neurophysiology 30(2) (1990) 97-103. | Excluded | Title and abstract | Irrelevant |
| 439] A. Dzaja, R. Wehrle, M. Lancel, T. Pollmaecher, Elevated Estradiol Plasma Levels in Women with Restless Legs during Pregnancy, Sleep 32(2) (2009) 169-174. | Excluded | Full text | No relevant outcome |
| 440] C. Trenkwalder, Emergency call from gynecologists: How to treat restless legs syndrome during pregnancy?, European journal of neurology 20(9) (2013) 1223-1224. | Excluded | Title and abstract | Irrelevant |
| 441] S. Perlman, Emerging Therapies in Friedreich’s Ataxia: A Review, touchREVIEWS in Neurology 18(1) (2022) 32-37. | Excluded | Title and abstract | Irrelevant |
| 442] Y. Cheng, L. Illum, J. Bond, P. Watts, Emulsion composition useful for the delivery of a benzodiazepine drug comprises an aqueous phase, an emulsion stabilizer, and an oil phase comprising emulsion, vitamin E and the drug, West Pharm Services Drug Delivery & Clin. | Excluded | Title and abstract | Irrelevant |
| 443] R.L. Ruff, J. Weissmann, Endocrine myopathies, Neurologic clinics 6(3) (1988) 575-92. | Excluded | Title and abstract | Irrelevant |
| 444] Nct, Energy Drink Effects on Performance, Mood, and Cardiovascular Outcomes, https://clinicaltrials.gov/show/NCT05559372 (2022). | Excluded | Title and abstract | Irrelevant |
| 445] A.A. Sharif, Entacapone in restless legs syndrome, Movement Disorders 17(2) (2002) 421. | Excluded | Title and abstract | Irrelevant |
| 446] M. Rechichi, S. Daya, V. Scorcia, A. Meduri, G. Scorcia, Epithelial-disruption collagen crosslinking for keratoconus: one-year results, Journal of cataract and refractive surgery 39(8) (2013) 1171‐1178. | Excluded | Title and abstract | Irrelevant |
| 447] S. Wali, A. Shukr, A. Boudal, A. Alsaiari, A. Krayem, Erratum to: The effect of vitamin D supplements on the severity of restless legs syndrome, Sleep & breathing = Schlaf & Atmung 19(4) (2015) 1483. | Excluded | Title and abstract | Duplication |
| 448] S. Wali, A. Shukr, A. Boudal, A. Alsaiari, A. Krayem, Erratum to: The effect of vitamin D supplements on the severity of restless legs syndrome (Int Urogynecol J, (2015), 19, 2, 579–83, 10.1007/s11325-014-1049-y), Sleep and Breathing 19(4) (2015) 1483. | Excluded | Title and abstract | Duplication |
| 449] J.P. Hintze, D. Gault, Escitalopram for recurrent isolated sleep paralysis, Journal of Sleep Research 29(6) (2020). | Excluded | Title and abstract | Irrelevant |
| 450] R.K. Yang, T. Uchiyama, S.K. Watkins, X.N. Han, M.P. Fink, Ethyl pyruvate reduces liver injury in a murine model of extrahepatic cholestasis, Shock 22(4) (2004) 369-375. | Excluded | Title and abstract | Irrelevant |
| 451] Irct20100102002954N, Evaluation effects of Vitamin C and Ferrous Sulfate in Restless leg treatment of hemodialysis patients, https://trialsearch.who.int/Trial2.aspx?TrialID=IRCT20100102002954N15 (2019). | Excluded | Title and abstract | Irrelevant |
| 452] Nct, Evaluation of a Folic Acid Wound Treatment (FAWT) for Chronic, Early-Stage Diabetic Foot Ulcer (DFU) Healing, https://clinicaltrials.gov/show/NCT04723134 (2021). | Excluded | Title and abstract | Irrelevant |
| 453] S. Ripley, C.R. Ronzio, C. Cozad, D.R. Cozad, M.J. Morgan, V.D. Mikhail, R.A. Ronzio, Evaluation of a multidisciplinary rehabilitation program for fibromyalgia: A pilot study, Today's Therapeutic Trends 21(2) (2003) 159-184. | Excluded | Title and abstract | Irrelevant |
| 454] Nct, Evaluation of a Supplement for Weight Management in Obese and Overweight Individuals, https://clinicaltrials.gov/show/NCT03812211 (2019). | Excluded | Title and abstract | Irrelevant |
| 455] X. Zhang, H. Xiao, Y. Chen, Evaluation of a WeChat‐based life review programme for cancer patients: a quasi‐experimental study, Journal of advanced nursing (john wiley & sons, inc.) 75(7) (2019) 1563‐1574. | Excluded | Title and abstract | Irrelevant |
| 456] A.Z.A. Tak, Y. Şengül, Evaluation of inflammation with neutrophil-to-lymphocyte ratio and platelet-to-lymphocyte ratio in restless legs syndrome, Turk Noroloji Dergisi 24(3) (2018) 259-263. | Excluded | Full text | No specific data |
| 457] A. Kısabay, U.S. Sarı, T. Korkmaz, G. Dinçhorasan, H. Yılmaz, D. Selçuki, Evaluation of neurodegeneration through visual evoked potentials in restless legs syndrome, Acta neurologica Belgica 116(4) (2016) 605-613. | Excluded | Title and abstract | Irrelevant |
| 458] T. Çakır, G. Doğan, V. Subaşı, M.B. Filiz, N. Ülker, K. Doğan Ş, N.F. Toraman, An evaluation of sleep quality and the prevalence of restless leg syndrome in vitamin D deficiency, Acta neurologica Belgica 115(4) (2015) 623-7. | Included | Full text | Relationship between vitamins and RLS |
| 459] E.T. Kornegay, T.N. Meacham, Evaluation of supplemental choline for reproducing sows housed in total confinement on concrete or in dirt lots, Journal of animal science 37(2) (1973) 506-9. | Excluded | Title and abstract | Irrelevant |
| 460] M. Çam, U. Kutluana, Evaluation of upper endoscopic findings in patients with restless legs syndrome and gastric complaints, Arquivos de neuro-psiquiatria 78(4) (2020) 217-223. | Included | Full text | Relationship between vitamins and RLS |
| 461] P.N. Kannegaard, S. van der Mark, P. Eiken, B. Abrahamsen, Excess mortality in men compared with women following a hip fracture. National analysis of comedications, comorbidity and survival, Age and ageing 39(2) (2010) 203-209. | Excluded | Title and abstract | Irrelevant |
| 462] I. Aricò, L. Campolo, M. Caffarelli, L. Mirci, M. Restuccia, R. Silvestri, Excessive daytime sleepiness and vitamin d deficiency in a cohort of patients with obstructive sleep apnea, Sleep 40 (2017) A238. | Excluded | Title and abstract | Irrelevant |
| 463] P. Siarnik, M. Jurik, M. Hardonova, K. Klobucnikova, J. Veverka, P. Surda, P. Turcani, B. Kollar, Excessive Daytime Sleepiness in Sleep Apnea: Any Role of Testosterone or Vitamin D?, Physiological Research 69(5) (2020) 907-917. | Excluded | Title and abstract | Irrelevant |
| 464] L. Sylvester, S. Paik, Excessive sleepiness and its impact on shift workers: An internet survey of shift workers and patients with shift work disorder, Journal of general internal medicine 27 (2012) S187-S188. | Excluded | Title and abstract | Irrelevant |
| 465] R. Lane, A. Harwood, L. Watson, G.C. Leng, Exercise for intermittent claudication, Cochrane Database of Systematic Reviews (12) (2017). | Excluded | Title and abstract | Irrelevant |
| 466] E. Blotnick, L. Anglister, EXERCISE MODULATES SYNAPTIC ACETYLCHOLINESTERASE AT NEUROMUSCULAR JUNCTIONS, Neuroscience 319 (2016) 221-232. | Excluded | Title and abstract | Irrelevant |
| 467] G. Gravier, J.G. Steinberg, P.J. Lejeune, S. Delliaux, R. Guieu, Y. Jammes, Exercise-induced oxidative stress influences the motor control during maximal incremental cycling exercise in healthy humans, Respiratory Physiology & Neurobiology 186(3) (2013) 265-272. | Excluded | Title and abstract | Irrelevant |
| 468] J. Sampson, L. Baird, J. Stevens, N. Matsunami, M. Leppert, Exome Sequencing of Individuals with Early Onset Familial Restless Legs Syndrome (Willis-Ekbom Disease), Neurology 78 (2012). | Excluded | Title and abstract | Irrelevant |
| 469] J. Sampson, L. Baird, J. Stevens, N. Matsunami, M. Leppert, Exome sequencing of two individuals with early onset familial ekbom syndrome, Sleep medicine 12 (2011) S17. | Excluded | Title and abstract | Irrelevant |
| 470] Umin, Exploratory Clinical Trial of EPI-743 in Japanese MELAS Syndrome Patients, https://trialsearch.who.int/Trial2.aspx?TrialID=JPRN-UMIN000010783 (2013). | Excluded | Title and abstract | Irrelevant |
| 471] F. Babayan-Mashhadi, A. Rezvani-Noghani, P. Mokaberi, Z. Amiri-Tehranizadeh, M.R. Saberi, J. Chamani, Exploring the binding behavior mechanism of vitamin B(12) to α-Casein and β-Casein: multi-spectroscopy and molecular dynamic approaches, Journal of biomolecular structure & dynamics (2023) 1-18. | Excluded | Title and abstract | Irrelevant |
| 472] F. Babayan-Mashhadi, A. Rezvani-Noghani, P. Mokaberi, Z. Amiri-Tehranizadeh, M.R. Saberi, J. Chamani, Exploring the binding behavior mechanism of vitamin B<sub>12</sub> to α-Casein and β-Casein: multi-spectroscopy and molecular dynamic approaches, Journal of biomolecular structure & dynamics (2023). | Excluded | Title and abstract | Irrelevant |
| 473] P. Zeng, T. Wang, L. Zhang, F. Guo, Exploring the causes of augmentation in restless legs syndrome, Front Neurol 14 (2023) 1160112. | Excluded | Title and abstract | Irrelevant |
| 474] P.J. Blanco, R.P. Holliman, P.L. Ceballos, J.L. Farnam, Exploring the Impact of Child-Centered Play Therapy on Academic Achievement of At-Risk Kindergarten Students, International journal of play therapy 28(3) (2019) 133‐143. | Excluded | Title and abstract | Irrelevant |
| 475] M. Kolbel, F.J. Kirkham, R.K. Iles, H. Stotesbury, E. Halstead, C. Brenchley, S. Sahota, D. Dimitriou, Exploring the relationship of sleep, cognition, and cortisol in sickle cell disease, Comprehensive psychoneuroendocrinology 10 (2022) 100128-100128. | Excluded | Title and abstract | Irrelevant |
| 476] A. Garcia-Quintanilla, D. Miranzo-Navarro, Extraintestinal manifestations of celiac disease: 33-mer gliadin binding to glutamate receptor GRINA as a new explanation, BioEssays 38(5) (2016) 427-439. | Excluded | Title and abstract | Irrelevant |
| 477] R.C. Popa, A. Savin, I. Ungureanu, O. Gavrilescu, M. Dranga, A.M. Chiosa, O. Jigaranu, I. Popa, C. Mihai, C. Cijevschi-Prelipcean, Factors associated with restless legs syndrome in patients with ulcerative colitis: A study in a tertiary care center in Romania, Journal of Crohn's and Colitis 11 (2017) S179. | Excluded | Full text | No specific data |
| 478] W.C. Graafmans, M.E. Ooms, H.M. Hofstee, P.D. Bezemer, L.M. Bouter, P. Lips, Falls in the elderly: a prospective study of risk factors and risk profiles, American journal of epidemiology 143(11) (1996) 1129‐1136. | Excluded | Title and abstract | Irrelevant |
| 479] K. Aoki, Y. Washimi, N. Fujimori, K. Maruyama, N. Yanagisawa, Familial idiopathic vitamin E deficiency associated with cerebellar atrophy, Rinsho shinkeigaku = Clinical neurology 30(9) (1990) 966-71. | Excluded | Title and abstract | Irrelevant |
| 480] C.K. Akpinar, H. Turker, D. Aygun, E. Aytac, Familial restless legs syndrome: A family with all female patients, Archives of Iranian medicine 20(2) (2017) 105-107. | Excluded | Title and abstract | Irrelevant |
| 481] Nct, Fasting-mimicking Diet and Periodontitis (FMD), https://clinicaltrials.gov/ct2/show/NCT06074861 (2023). | Excluded | Title and abstract | Irrelevant |
| 482] P. Maggi, B. Solarino, P. Cassano, L. Tattoli, A. Leone, E. Maselli, G. Angarano, Fatal fulminant hepatitis following administration of clarithromycin in a patient chronically treated with antipsycotic drugs, Immunopharmacology and Immunotoxicology 35(1) (2013) 191-194. | Excluded | Title and abstract | Irrelevant |
| 483] T.J. Braley, R.D. Chervin, Fatigue in Multiple Sclerosis: Mechanisms, Evaluation, and Treatment, Sleep 33(8) (2010) 1061-1067. | Excluded | Title and abstract | Irrelevant |
| 484] M. Hirshkowitz, Fatigue, sleepiness, and safety: Definitions, assessment, methodology, Sleep Medicine Clinics 8(2) (2013) 183-189. | Excluded | Title and abstract | Irrelevant |
| 485] V. Colombo, D. Pagani, I. Gregorio, "Feeding With Love": the Severe Anemias and Neurodisabilities in Bolivian Population, 9th International Congress on Cerebral Palsy - Mental and Physical Activity are Imperative, Bled, SLOVENIA, 2013, pp. 13-18. | Excluded | Title and abstract | Irrelevant |
| 486] Fertility 2021Barriers and breakthroughs6–10th January 2021 Online: The joint conference of the Association of Reproductive & Clinical Scientists, British Fertility Society, and the Society for Reproduction & Fertility, Human Fertility 24(1) (2021) 46-69. | Excluded | Title and abstract | Irrelevant |
| 487] C.P. Motley, M.L. Maxwell, Fibromyalgia: Helping your patient while maintaining your sanity, Primary Care - Clinics in Office Practice 37(4) (2010) 743-755. | Excluded | Title and abstract | Irrelevant |
| 488] Nct, Fiix-prothrombin Time for Monitoring Warfarin, https://clinicaltrials.gov/show/NCT01565239 (2012). | Excluded | Title and abstract | Irrelevant |
| 489] B. Prasad, M. Gagarinova, A. Sharma, Five Things to Know About Restless Legs Syndrome in Patients on Dialysis, Canadian journal of kidney health and disease 10 (2023). | Excluded | Title and abstract | Irrelevant |
| 490] P.S. Kishnani, C. Rockman-Greenberg, F. Rauch, M.T. Bhatti, S. Moseley, A.E. Denker, E. Watsky, M.P. Whyte, Five-year efficacy and safety of asfotase alfa therapy for adults and adolescents with hypophosphatasia, Bone 121 (2019) 149‐162. | Excluded | Title and abstract | Irrelevant |
| 491] M.M. Maues, G. Couturier, Floral biology and reproductive phenology of the camu-camu (Myrciaria dubia (H.B.K.) McVaugh, Myrtaceae) in the State of Para, Brazil, Revista Brasileira de Botanica 25(4) (2002) 441-448. | Excluded | Title and abstract | Irrelevant |
| 492] R.C. Deth, M. Waly, FOLATE - DEPENDENT PHOSPHOLIPID METHYLATION IS INCREASED BY IGF - 1 AND REDUCED BY ETHANOL AND Pi3 KINASE INHIBITORS, Society for Neuroscience Abstract Viewer and Itinerary Planner 2002 (2002) Abstract No. 305.19-Abstract No. 305.19. | Excluded | Title and abstract | Irrelevant |
| 493] M. Audebert, J.P. Gendre, Y. Le Quintrec, Folate and the nervous system, Semaine des Hopitaux 55(31-32) (1979) 1383-1387. | Excluded | Title and abstract | Irrelevant |
| 494] M. Audebert, J.P. Gendre, Y. Le Quintrec, Folate and the nervous system (author's transl)], La semaine des hopitaux : organe fonde par l'Association d'enseignement medical des hopitaux de Paris 55(31-32) (1979) 1383-7. | Excluded | Title and abstract | Irrelevant |
| 495] M.I. Botez, Folate deficiency and neurological disorders in adults, Medical hypotheses 2(4) (1976) 135-40. | Excluded | Full text | No relevant outcome |
| 496] M.I. Botez, B. Lambert, Folate deficiency and restless-legs syndrome in pregnancy, The New England journal of medicine 297(12) (1977) 670. | Included | Full text | Relationship between vitamins and RLS |
| 497] M.I. Botez, F. Fontaine, T. Botez, J. Bachevalier, Folate responsive neurological and mental disorders: report of 16 cases. Neuropsychological correlates of computerized transaxial tomography and radionuclide cisternography in folic acid deficiencies, European neurology 16(1-6) (1977) 230-246. | Excluded | Title and abstract | Irrelevant |
| 498] M.I. Botez, F. Fontaine, T. Botez, J. Bachevalier, Folate-responsive neurological and mental disorders: report of 16 cases. Neuropsychological correlates of computerized transaxial tomography and radionuclide cisternography in folic acid deficiencies, European neurology 16(1-6) (1977) 230-46. | Excluded | Title and abstract | Irrelevant |
| 499] G.S. Kelly, Folates: supplemental forms and therapeutic applications, Alternative medicine review : a journal of clinical therapeutic 3(3) (1998) 208-20. | Excluded | Title and abstract | Irrelevant |
| 500] P. Salerno, F. Bianchi, A. Pierini, F. Baldi, P. Carbone, A. Mantovani, D. Taruscio, Folic acid and congenital malformation: scientific evidence and public health strategies, Annali di igiene 20(6) (2008) 519‐530. | Excluded | Title and abstract | Irrelevant |
| 501] Nct, Folic Acid Dosage and Malformations Reduction, https://clinicaltrials.gov/show/NCT01244347 (2010). | Excluded | Title and abstract | Irrelevant |
| 502] M.I. Botez, B. Lambert, Folic acid, pregancy and the nervous system (general review)], L'union medicale du Canada 108(8) (1979) 939-49. | Excluded | Title and abstract | Irrelevant |
| 503] M.I. Botez, B. Lambert, Folic acid, pregnancy and the nervous system, Union Medicale du Canada 108(8) (1979) 939-949. | Excluded | Title and abstract | Irrelevant |
| 504] Y. Tian, J.C. Li, J.X. Zhu, N. Zhu, H.M. Zhang, L. Liang, L. Sun, Folic Acid-Targeted Etoposide Cubosomes for Theranostic Application of Cancer Cell Imaging and Therapy, Medical science monitor : international medical journal of experimental and clinical research 23 (2017) 2426-2435. | Excluded | Title and abstract | Irrelevant |
| 505] Nct, Folinic Acid and Vascular Reactivity in HIV, https://clinicaltrials.gov/show/NCT01768182 (2013). | Excluded | Title and abstract | Irrelevant |
| 506] H. Martinez, H. Pachon, V. Kancherla, G.P.J. Oakley, Jr., Food Fortification With Folic Acid for Prevention of Spina Bifida and Anencephaly: The Need for a Paradigm Shift in Evidence Evaluation for Policy-Making, American journal of epidemiology 190(10) (2021) 1972-1976. | Excluded | Title and abstract | Irrelevant |
| 507] Y.M. Báez-Santos, A. Otte, E.A. Mun, B.K. Soh, C.G. Song, Y.N. Lee, K. Park, Formulation and characterization of a liquid crystalline hexagonal mesophase region of phosphatidylcholine, sorbitan monooleate, and tocopherol acetate for sustained delivery of leuprolide acetate, International journal of pharmaceutics 514(1) (2016) 314-321. | Excluded | Title and abstract | Irrelevant |
| 508] Nct, Fortified Cheese and Yogurt Products and Vitamin D Status in Young Children? Phase 2, https://clinicaltrials.gov/show/NCT02387892 (2015). | Excluded | Title and abstract | Irrelevant |
| 509] L.M. Paterson, D. Barker, S. Cro, P. Mozgunov, R. Phillips, C. Smith, L. Nahar, S. Paterson, A.R. Lingford-Hughes, FORWARDS-1: an adaptive, single-blind, placebo-controlled ascending dose study of acute baclofen on safety parameters in opioid dependence during methadone-maintenance treatment-a pharmacokinetic-pharmacodynamic study, Trials 23(1) (2022). | Excluded | Title and abstract | Irrelevant |
| 510] L. Baldelli, F. Provini, Fragmentary Hypnic Myoclonus and Other Isolated Motor Phenomena of Sleep, Sleep Medicine Clinics 16(2) (2021) 349-361. | Excluded | Title and abstract | Irrelevant |
| 511] S. Civi, R. Kutlu, S. Tokgöz, Frequency, severity and risk factors for restless legs syndrome in healthcare personnel, Neurosciences 17(3) (2012) 230-235. | Included | Full text | Relationship between vitamins and RLS |
| 512] D.F. Branquinho, M. Pinto-Gouveia, S. Mendes, C. Sofia, From past sailors' eras to the present day: scurvy as a surprising manifestation of an uncommon gastrointestinal disease, BMJ case reports 2015 (2015). | Excluded | Title and abstract | Irrelevant |
| 513] From Preserve's 2009 drug review: A recap of which new drugs to avoid, Prescrire international 19(106) (2010) 76-80. | Excluded | Title and abstract | Irrelevant |
| 514] S. Imamura, C. Kushida, Gabapentin enacarbil (XP13512/GSK1838262) as an alternative treatment to dopaminergic agents for restless legs syndrome, Expert opinion on pharmacotherapy 11(11) (2010) 1925-1932. | Excluded | Title and abstract | Irrelevant |
| 515] J. Lipson, S. Lavoie, D. Zimmerman, Gabapentin-induced myopathy in 2 patients on short daily hemodialysis, American Journal of Kidney Diseases 45(6) (2005) e100-e104. | Excluded | Title and abstract | Irrelevant |
| 516] B.J. Nergaard, B.G. Leifsson, J. Hedenbro, H. Gislason, Gastric bypass with long alimentary limb or long pancreato-biliary limb--long-term results on weight loss, resolution of co-morbidities and metabolic parameters, Obesity surgery 24(10) (2014) 1595‐1602. | Excluded | Title and abstract | Irrelevant |
| 517] C. Jobin, C. Larochelle, H. Parpal, P.K. Coyle, P. Duquette, Gender issues in multiple sclerosis: An update, Women's Health 6(6) (2010) 797-820. | Excluded | Title and abstract | Irrelevant |
| 518] O. Schaefer, J.F. Timmermans, R.D. Eaton, A.R. Matthews, General and nutritional health in two Eskimo populations at different stages of acculturation, Canadian journal of public health = Revue canadienne de sante publique 71(6) (1980) 397-405. | Excluded | Title and abstract | Irrelevant |
| 519] R.A. Marrie, H. Hanwell, General health issues in multiple sclerosis: Comorbidities, secondary conditions, and health behaviors, CONTINUUM Lifelong Learning in Neurology 19(4) (2013) 1046-1057. | Excluded | Title and abstract | Irrelevant |
| 520] J. Chen, Q. Luo, G. Li, Y. Huang, J. Ma, Genetic Association Study of Restless Legs Syndrome in Chinese Population, European neurology 81(1-2) (2019) 47-55. | Excluded | Title and abstract | Irrelevant |
| 521] S. Kaneko, M. Okada, H. Iwasa, K. Yamakawa, S. Hirose, Genetics of epilepsy: current status and perspectives, Neuroscience Research 44(1) (2002) 11-30. | Excluded | Title and abstract | Irrelevant |
| 522] F.J. Jiménez-Jiménez, H. Alonso-Navarro, E. García-Martín, J.A.G. Agúndez, Genetics of restless legs syndrome: An update, Sleep Med Rev 39 (2018) 108-121. | Excluded | Title and abstract | Irrelevant |
| 523] H. Xu, F. Liu, Z. Li, X. Li, Y. Liu, N. Li, X. Zhang, Z. Gao, X. Zhang, Y. Liu, J. Zou, L. Meng, S. Liu, H. Zhu, X. Tang, H. Wu, K. Su, B. Chen, D. Yu, H. Ye, H. Chen, H. Yi, S. Yin, J. Guan, Y. Shi, Genome-Wide Association Study of Obstructive Sleep Apnea and Objective Sleep-related Traits Identifies Novel Risk Loci in Han Chinese Individuals, American Journal of Respiratory and Critical Care Medicine 206(12) (2022) 1534-1545. | Excluded | Title and abstract | Irrelevant |
| 524] S. Hausman-Cohen, C. Bilich, S. Kapoor, E. Maristany, A. Stefani, A. Wilcox, Genomics as a Clinical Decision Support Tool for Identifying and Addressing Modifiable Causes of Cognitive Decline and Improving Outcomes: Proof of Concept Support for This Personalized Medicine Strategy, Frontiers in aging neuroscience 14 (2022). | Excluded | Title and abstract | Irrelevant |
| 525] Gerontology forum: An update on the literature, Drugs and Aging 23(8) (2006) 681-692. Excluded Title and abstract Irrelevant | Excluded | Title and abstract | Irrelevant |
| 526] F. Irvine, A. Almond, S. Robertson, K. Donaldson, R. Flynn, C. Isles, Getting the right medicine: A survey of haemodialysis patients in SW Scotland, Pharmaceutical Journal 283(7579) (2009) 575-576. | Excluded | Title and abstract | Irrelevant |
| 527] S. Marshall, Growing pains are usually benign, Pharmaceutical Journal 287(7665-7666) (2011) 170. | Excluded | Title and abstract | Irrelevant |
| 528] F.B. Tinning, L. Hestbæk, A.N. Andersen, Growing pains are well-known, but the cause remains unclear], Ugeskrift for laeger 179(16) (2017). | Excluded | Title and abstract | Irrelevant |
| 529] R.M. Lowe, P.J. Hashkes, Growing pains: A noninflammatory pain syndrome of early childhood, Nature Clinical Practice Rheumatology 4(10) (2008) 542-549. | Excluded | Title and abstract | Irrelevant |
| 530] M.P. Mohanta, Growing pains: Practitioners' dilemma, Indian Pediatrics 51(5) (2014) 379-383. | Excluded | Title and abstract | Irrelevant |
| 531] D. Schnabel, M. Zivicnjak, H. Staude, D. Haffner, Growth hormone treatment (rhGH) in severely growth retarded children with x-linked hypophosphatemic rickets - Results of a 3 year randomized, controlled study, Bone 45 (2009) S54. | Excluded | Title and abstract | Irrelevant |
| 532] C.J. McGrath, Growth in HIV-1 infected and uninfected children in Kenya, 2011. | Excluded | Title and abstract | Irrelevant |
| 533] A.A. Mehl, A.O. Damião, S.D. Viana, C.P. Andretta, Hard-to-heal wounds: a randomised trial of an oral proline-containing supplement to aid repair, Journal of wound care 30(1) (2021) 26‐31. | Excluded | Title and abstract | Irrelevant |
| 534] Y. Sugita, Has the disease identity of restless legs syndrome developed or been distorted? Astronauts in zero gravity may know the answer, Internal medicine journal 41(9) (2011) 706-707. | Excluded | Title and abstract | Irrelevant |
| 535] A. Dotevall, E. Krantz, M.L. Barrenas, K. Landin-Wilhelmsen, Hearing and Balance Exceed Initial Bone Mineral Density in Predicting Incident Fractures: a 25-Year Prospective Observational Study in Menopausal Women With Osteoporosis, JBMR plus 6(1) (2022). | Excluded | Title and abstract | Irrelevant |
| 536] D.K. Euctr, The HEAT-study (The Herlev Enzalutamide versus Abiraterone Toxicity-study), https://trialsearch.who.int/Trial2.aspx?TrialID=EUCTR2017-000099-27-DK (2017). | Excluded | Title and abstract | Irrelevant |
| 537] A. Hunt, K. Ibrahim, M.J.H. Rahmani, The Heidenhain variant of Creutzfeldt-Jakob disease, British journal of hospital medicine 79(12) (2018) 712-713. | Excluded | Title and abstract | Irrelevant |
| 538] T.A. Özcan, K. Aksöz, N. Aksoy, T. Noyan, Helicobacter pylori infection in patients with neurological symptoms of vitamin B12 deficiency, Journal of Neurological Sciences 30(3) (2013) 487-493. | Excluded | Title and abstract | Irrelevant |
| 539] J.C. Barton, V.D. Wooten, R.T. Acton, Hemochromatosis and iron therapy of Restless Legs Syndrome, Sleep medicine 2(3) (2001) 249-251. | Excluded | Title and abstract | Irrelevant |
| 540] A. Olmez, H. Topaloglu, Hereditary spastic paraplegia, Milli Nevrologiya Jurnali (1) (2013) 13-17. | Excluded | Title and abstract | Irrelevant |
| 541] A.M. Afifi, L. Ellis, R.G. Huntsman, M.I. Said, High dose ascorbic acid in the management of thalassaemia leg ulcers--a pilot study, British journal of dermatology 92(3) (1975) 339‐341. | Excluded | Title and abstract | Irrelevant |
| 542] Isrctn, High dose Coenzyme Q10 and vitamin E therapy in Friedreich's ataxia, https://trialsearch.who.int/Trial2.aspx?TrialID=ISRCTN87024790 (2007). | Excluded | Title and abstract | Irrelevant |
| 543] M. Graf, D. Ecker, R. Horowski, B. Kramer, P. Riederer, M. Gerlach, C. Hager, A.C. Ludolph, G. Becker, J. Osterhage, et al., High dose vitamin E therapy in amyotrophic lateral sclerosis as add-on therapy to riluzole: results of a placebo-controlled double-blind study, Journal of neural transmission (Vienna, Austria : 1996) 112(5) (2005) 649‐660. | Excluded | Title and abstract | Irrelevant |
| 544] G. Birgegard, K. Schneider, J. Ulfberg, High incidence of iron depletion and restless leg syndrome (RLS) in regular blood donors: intravenous iron sucrose substitution more effective than oral iron, Vox Sanguinis 99(4) (2010) 354-361. | Excluded | Title and abstract | Irrelevant |
| 545] M. Minár, D. Petrleničová, P. Valkovič, Higher prevalence of restless legs syndrome/Willis-Ekbom disease in multiple sclerosis patients is related to spinal cord lesions, Mult Scler Relat Disord 12 (2017) 54-58. | Included | Full text | Relationship between vitamins and RLS |
| 546] Isrctn, A high-intensity functional exercise program for older people with dementia and living in residential care facilities (The Umeå Dementia and Exercise Study ? The UMDEX Study), https://trialsearch.who.int/Trial2.aspx?TrialID=ISRCTN31767087 (2011). | Excluded | Title and abstract | Irrelevant |
| 547] Y. Kuang, S. Chen, Y. Long, Highly sensitive and selective determination of hydrogen sulfide by resonance light scattering technique based on silver nanoparticles, Anal Bioanal Chem 409(16) (2017) 4001-4008. | Excluded | Title and abstract | Irrelevant |
| 548] M.A. Raj, N.S.K. Gowthaman, S.A. John, Highly sensitive interference-free electrochemical determination of pyridoxine at graphene modified electrode: Importance in Parkinson and Asthma treatments, Journal of Colloid and Interface Science 474 (2016) 171-178. | Excluded | Title and abstract | Irrelevant |
| 549] T. Yoshikawa, T. Nakamura, K. Yanai, Histamine N-Methyltransferase in the Brain, International journal of molecular sciences 20(3) (2019). | Excluded | Title and abstract | Irrelevant |
| 550] J. Ganguly, P.S. Joshi, S.K. Murthy, E. Unni, G.F. David, T.C. Anand Kumar, Histological effects of vitamin A deprivation on estrogen-induced development of chick oviduct, Indian journal of experimental biology 21(2) (1983) 69-72. | Excluded | Title and abstract | Irrelevant |
| 551] C.G. Bachmann, N. Guth, K. Helmschmied, V.W. Armstrong, W. Paulus, S. Happe, Homocysteine in restless legs syndrome, Sleep medicine 9(4) (2008) 388-92. | Excluded | Full text | Data couldn't be separately extracted |
| 552] L.M. Pautrier, R. Gonin, Horny Verrucoma of the Foot, Remarkable for the Size and Variety of Dyskeratosis Lesions, Cancer-like Cells and Abundance of Mitosis, in a Patient Elsewhere with Multiple Epitheliomatosis and Horn Papilloma of the Face, Dermatologica 92(1) (1946) 67-70. | Excluded | Title and abstract | Irrelevant |
| 553] J. Muralitharan, V. Nagarajan, U. Ravichandran, Hyperemesis Gravidarum With Paraparesis and Tetany, Cureus 13(8) (2021) e17014-e17014. | Excluded | Title and abstract | Irrelevant |
| 554] A. Reffo, C. Gabelli, Hyperhomocysteinemia and Dementia Associated With Severe Cortical Atrophy, but No Amyloid Burden, Journal of geriatric psychiatry and neurology 35(1) (2022) 57-61. | Excluded | Title and abstract | Irrelevant |
| 555] D. Ghosh, R.K. Dhiman, A. Kohli, S.R. Naik, Hypokalemic periodic paralysis in association with tropical sprue: a case report, Acta neurologica Scandinavica 90(5) (1994) 371-3. | Excluded | Title and abstract | Irrelevant |
| 556] A. Schuetzenduebel, M. Stadler, D. Wallner, A. von Tiedemann, A hypothesis on physiological alterations during plant ontogenesis governing susceptibility of winter barley to ramularia leaf spot, Plant Pathology 57(3) (2008) 518-526. | Excluded | Title and abstract | Irrelevant |
| 557] P.P. Sharma, S. Kumar, K. Kaushik, A. Singh, I.K. Singh, M. Grishina, K.C. Pandey, P. Singh, V. Potemkin, Poonam, G. Singh, B. Rathi, <i>In silico</i> validation of novel inhibitors of malarial aspartyl protease, plasmepsin V and antimalarial efficacy prediction, Journal of biomolecular structure & dynamics 40(18) (2022) 8352-8364. | Excluded | Title and abstract | Irrelevant |
| 558] R.S. Molday, L.L. Molday, Identification and characterization of multiple forms of rhodopsin and minor proteins in frog and bovine rod outer segment disc membranes. Electrophoresis, lectin labeling, and proteolysis studies, The Journal of biological chemistry 254(11) (1979) 4653-60. | Excluded | Title and abstract | Irrelevant |
| 559] C. Wu, X. Yang, L. Feng, F. Wang, H. Tang, Y. Yin, Identification of key leaf color-associated genes in <i>Gleditsia sinensis</i> using bioinformatics, Horticulture Environment and Biotechnology 60(5) (2019) 711-720. | Excluded | Title and abstract | Irrelevant |
| 560] P. Kanta, N.R. Gopinathan, Idiopathic Growing Pains in Pediatric Patients: Review of Literature, Clinical Pediatrics 58(1) (2019) 5-9. | Excluded | Title and abstract | Irrelevant |
| 561] J. Ma, J. Chen, IL1B Polymorphism is associated with essential tremor but not restless legs syndrome in chinese population, Sleep medicine 64 (2019) S236. | Excluded | Title and abstract | Irrelevant |
| 562] Y. Yang, L. Wang, J. Yin, X. Wang, S. Cheng, X. Lang, X. Wang, H. Qu, C. Sun, J. Wang, R. Zhang, Immunoproteomic analysis of <i>Brucella</i> <i>melitensis</i> and identification of a new immunogenic candidate protein for the development of brucellosis subunit vaccine, Molecular Immunology 49(1-2) (2011) 175-184. | Excluded | Title and abstract | Irrelevant |
| 563] Huzmeli C .The relationship between restless leg syndrome and 25-hydroxy D vitamin in hemodialysis patients[J]. J Clin Anal Med 2018;9(1): 65-8 | Included | Full text | Relationship between vitamins and RLS |
| 564] Actrn, Impact loading training in people with chronic obstructive pulmonary disease: a pilot randomised controlled trial examining the feasibility and tolerance of this intervention and its effect on bone health, https://trialsearch.who.int/Trial2.aspx?TrialID=ACTRN12620001085965 (2020). | Excluded | Title and abstract | Irrelevant |
| 565] Nct, The Impact of Consumption of Eggs in the Context of Plant-Based Diets on Endothelial Function, Diet Quality, and Cardio-Metabolic Risk Factors in Adults at Risk for Type 2 Diabetes, https://clinicaltrials.gov/show/NCT04316429 (2020). | Excluded | Title and abstract | Irrelevant |
| 566] L. Shi, M. Hudges, N. Yurgin, K.S. Boye, Impact of dose frequency on compliance and health outcomes: A literature review (1966-2006), Expert Review of Pharmacoeconomics and Outcomes Research 7(2) (2007) 187-202. | Excluded | Title and abstract | Irrelevant |
| 567] Nct, Impact of Intravenous Iron Treatment of Preoperative Anemia in Patients With LEAD (IRONPAD), https://clinicaltrials.gov/show/NCT04083755 (2019). | Excluded | Title and abstract | Irrelevant |
| 568] Isrctn, The impact of nutrition standards for school meals on the diets of schoolchildren in Cambodia, https://trialsearch.who.int/Trial2.aspx?TrialID=ISRCTN79659112 (2023). | Excluded | Title and abstract | Irrelevant |
| 569] R.S.S. Santos, R.M.A. Moyses, R.M. Elias, Impact of Restless Leg Syndrome on mortality in patients on hemodialysis: still debatable, Sleep medicine 22 (2016) 102. | Excluded | Title and abstract | Irrelevant |
| 570] Nct, Impact of Vitamin C on Pain Relief After an Emergency Department Visit for Acute Musculoskeletal Pain, https://clinicaltrials.gov/show/NCT05555576 (2022). | Excluded | Title and abstract | Irrelevant |
| 571] J.T. Cramer, A.J. Cruz-Jentoft, F. Landi, M. Hickson, M. Zamboni, S.L. Pereira, D.S. Hustead, V.A. Mustad, Impacts of High-Protein Oral Nutritional Supplements Among Malnourished Men and Women with Sarcopenia: a Multicenter, Randomized, Double-Blinded, Controlled Trial, Journal of the American Medical Directors Association 17(11) (2016) 1044‐1055. | Excluded | Title and abstract | Irrelevant |
| 572] Y.K. Kim, I.Y. Yoon, J.M. Kim, S.H. Jeong, K.W. Kim, Y.K. Shin, B.S. Kim, S.E. Kim, The implication of nigrostriatal dopaminergic degeneration in the pathogenesis of REM sleep behavior disorder, European journal of neurology 17(3) (2010) 487-492. | Excluded | Title and abstract | Irrelevant |
| 573] M.A. Topçuoglu, F.S. Buonanno, Importance of jugular valve incompetence in contrast transcranial Doppler ultrasonography for the diagnosis of patent foramen ovale, Journal of Neuroimaging 13(3) (2003) 272-275. | Excluded | Title and abstract | Irrelevant |
| 574] C.J. Earley, The importance of oral iron therapy in restless legs syndrome, Sleep medicine 10(9) (2009) 945-946. | Excluded | Title and abstract | Irrelevant |
| 575] J. Hsiao, Y.C. Shen, Impressive Response with Brexpiprazole in Ekbom's syndrome, European Psychiatry 65 (2022) S795. | Excluded | Title and abstract | Irrelevant |
| 576] L. Boateng, I. Ashley, A. Ohemeng, M. Asante, M. Steiner-Asiedu, Improving Blood Retinol Concentrations with Complementary Foods Fortified with Moringa oleifera Leaf Powder - A Pilot Study, Yale journal of biology and medicine 91(2) (2018) 83‐94. | Excluded | Title and abstract | Irrelevant |
| 577] T. S, An in Vitro Assessment of Cariogenic and Erosive Potential of Pediatric Liquid Medicaments on Primary Teeth: A Comparative Study, 2018. | Excluded | Title and abstract | Irrelevant |
| 578] T. Singana, N.K. Suma, An In Vitro Assessment of Cariogenic and Erosive Potential of Pediatric Liquid Medicaments on Primary Teeth: A Comparative Study, International journal of clinical pediatric dentistry 13(6) (2020) 595-599. | Excluded | Title and abstract | Irrelevant |
| 579] K.L. Fritz, C.M. Seppanen, M.S. Kurzer, A. Csallany Saari, The in vivo antioxidant activity of soybean isoflavones in human subjects, Nutrition research (New York, N.Y.) 23(4) (2003) 479‐487. | Excluded | Title and abstract | Irrelevant |
| 580] F.B. Balci, A. Mutlu, A.O. Cokar, The incidence and severity of restless legs syndrome in patients with multiple sclerosis, and its clinical impact, Multiple Sclerosis Journal 23(3) (2017) 1022-1023. | Excluded | Title and abstract | Irrelevant |
| 581] M.P.P. Sukel, N.S. Breekveldt-Postma, J.A. Erkens, P.D. van der Linden, A.B. Beiderbeck, J.W.W. Coebergh, R.M.C. Herings, Incidence of cardiovascular events in breast cancer patients receiving chemotherapy in clinical practice, Pharmacoepidemiology and drug safety 17(2) (2008) 125-134. | Excluded | Title and abstract | Irrelevant |
| 582] A.C. Chess, D.J. Bucci, Increased concentration of cerebral kynurenic acid alters stimulus processing and conditioned responding, Behavioural brain research 170(2) (2006) 326-332. | Excluded | Title and abstract | Irrelevant |
| 583] U. Tanriverdi, E.K. Kizilkilic, C. Boluk, G. Benbirsenel, D. Karadeniz, INFLAMMATORY BIOMARKERS IN SLEEP-RELATED BRUXISM, European journal of neurology 29 (2022) 835-835. | Excluded | Title and abstract | Irrelevant |
| 584] M.H. Mosli, L.M. Bukhari, A.A. Khoja, N.A. Ashour, H.R. Aljahdali, O.A. Khoja, F.F. Alhejaili, O.I. Saadah, Inflammatory bowel disease and restless leg syndrome, Neurosciences (Riyadh, Saudi Arabia) 25(4) (2020) 301-307. | Excluded | Title and abstract | Irrelevant |
| 585] A. Jaehne, T. Unbehaun, B. Feige, D. Riemann, The Influence of 8 and 16 mg Nicotine Patches on Sleep in Healthy Non-Smokers, Pharmacopsychiatry 47(2) (2014) 73-78. | Excluded | Title and abstract | Irrelevant |
| 586] R.B.R. hpjjk, Influence of a training protocol with vibrating platform of different amplitudes on postural balance, gait speed, muscle strength and risk of falls in the elderly: randomized crossover trial, https://trialsearch.who.int/Trial2.aspx?TrialID=RBR-2hpjjk (2019). | Excluded | Title and abstract | Irrelevant |
| 587] Nct, The Influence of Food Matrix Delivery System on the Bioavailability of Vitamin D3, https://clinicaltrials.gov/show/NCT03783273 (2018). | Excluded | Title and abstract | Irrelevant |
| 588] T. Giza, M. Hanicka, N. Hornik, A. Jelonek, H. Rembiesa, THE INFLUENCE OF RADIOACTIVE STRONTIUM ON THE COURSE OF HEALING OF EXPERIMENTAL RICKETS IN RATS TREATED WITH VITAMIN D2, Internationale Zeitschrift fur Vitaminforschung. International journal of vitamin research. Journal international de vitaminologie 33 (1963) 227-54. | Excluded | Title and abstract | Irrelevant |
| 589] Nct, Influence of Rivaroxaban for Intermittent Claudication and Exercise Tolerance in Patients With Symptomatic PAD, https://clinicaltrials.gov/show/NCT04305028 (2020). | Excluded | Title and abstract | Irrelevant |
| 590] M.M. Michalczyk, A. Golas, A. Maszczyk, P. Kaczka, A. Zajac, Influence of sunlight and oral d3 supplementation on serum 25(Oh)d concentration and exercise performance in elite soccer players, Nutrients 12(5) (2020). | Excluded | Title and abstract | Irrelevant |
| 591] S. Koussoulakos, H.J. Anton, Influence of vitamin A palmitate on the growth of regenerating Triturus alpestris forelimbs, Biological structures and morphogenesis 1(3) (1988) 124-9. | Excluded | Title and abstract | Irrelevant |
| 592] K.L. Kendall, J.R. Moon, C.M. Fairman, B.D. Spradley, C.Y. Tai, P.H. Falcone, L.R. Carson, M.M. Mosman, J.M. Joy, M.P. Kim, et al., Ingesting a preworkout supplement containing caffeine, creatine, beta-alanine, amino acids, and B vitamins for 28 days is both safe and efficacious in recreationally active men, Nutrition research (New York, N.Y.) 34(5) (2014) 442‐449. | Excluded | Title and abstract | Irrelevant |
| 593] K.L. Kendall, J.R. Moon, C.M. Fairman, B.D. Spradley, C.Y. Tai, P.H. Falcone, L.R. Carson, M.M. Mosman, J.M. Joy, M.P. Kim, et al., Ingesting a preworkout supplement containing caffeine, creatine, β-alanine, amino acids, and B vitamins for 28 days is both safe and efficacious in recreationally active men, Nutrition research (New York, N.Y.) 34(5) (2014) 442‐449. | Excluded | Title and abstract | Irrelevant |
| 594] E. Van der Kleijn, Inhibin®, a review, TGO - Tijdschrift voor Therapie Geneesmiddel en Onderzoek 14(1) (1989) 20-21. | Excluded | Title and abstract | Irrelevant |
| 595] S.C. Tseng, L.W. Hirst, M. Farazdaghi, W.R. Green, Inhibition of conjunctival transdifferentiation by topical retinoids, Investigative ophthalmology & visual science 28(3) (1987) 538-42. | Excluded | Title and abstract | Irrelevant |
| 596] X.G. Song, X.P. She, J. Wang, Inhibition of darkness-induced stomatal closure by ethylene involves a removal of hydrogen peroxide from guard cells of Vicia faba, Russian Journal of Plant Physiology 59(3) (2012) 372-380. | Excluded | Title and abstract | Irrelevant |
| 597] L.B. Tulloch, J. Howie, K.J. Wypijewski, C.R. Wilson, W.G. Bernard, M.J. Shattock, W. Fuller, The Inhibitory Effect of Phospholemman on the Sodium Pump Requires Its Palmitoylation, Journal of Biological Chemistry 286(41) (2011) 36020-36031. | Excluded | Title and abstract | Irrelevant |
| 598] Y. Sher, J.R. Maldonado, An Insatiable Desire for Tofu: A Case of Restless Legs and Unusual Pica in Iron Deficiency Anemia, Psychosomatics 55(6) (2014) 680-685. | Excluded | Title and abstract | Irrelevant |
| 599] P.J. Hauri, Insomnia, Clinics in Chest Medicine 19(1) (1998) 157-168. | Excluded | Title and abstract | Irrelevant |
| 600] E.L. Sutton, Insomnia, Medical Clinics of North America 98(3) (2014) 565-581. | Excluded | Title and abstract | Irrelevant |
| 601] B. Yadav, V. Kaur, O.P. Narayan, S.K. Yadav, A. Kumar, D.P. Wankhede, Integrated omics approaches for flax improvement under abiotic and biotic stress: Current status and future prospects, Frontiers in Plant Science 13 (2022). | Excluded | Title and abstract | Irrelevant |
| 602] Nct, An Intensive Lifestyle Intervention Program in CKD (Move to Health 2), https://clinicaltrials.gov/show/NCT02842957 (2016). | Excluded | Title and abstract | Irrelevant |
| 603] M. Choi, M. Mintz, D. Bates, Interaction between warfarin and apple juice, Canadian Journal of Hospital Pharmacy 69(1) (2016) 42-44. | Excluded | Title and abstract | Irrelevant |
| 604] C.J. Fuller, S.M. Lucas, N. Murinova, C.M. Douville, E. Tolentino, L.A. Braam, E.A. McGee, J.T. Jesurum, Interictal platelet activation in episodic migraine with aura with right-to-left circulatory shunt, Headache 51 (2011) 38. | Excluded | Title and abstract | Irrelevant |
| 605] Nct, Intermittent Pneumatic Compression of the Thigh, https://clinicaltrials.gov/show/NCT05659394 (2022). | Excluded | Title and abstract | Irrelevant |
| 606] Nct, International ALLIANCE Study of Therapies to Prevent Progression of COVID-19, https://clinicaltrials.gov/show/NCT04395768 (2020). | Excluded | Title and abstract | Irrelevant |
| 607] K. Zarabian, A. Wannon, M. Chin, M. Kogan, The intersection between integrative medicine and neuropathic pain: A case report, Explore 18(2) (2022) 165-169. | Excluded | Title and abstract | Irrelevant |
| 608] Nct, Intervention Trial to Measure the Effect of Individual Prenatal Information Combined With Mobile Phones, https://clinicaltrials.gov/show/NCT02084680 (2014). | Excluded | Title and abstract | Irrelevant |
| 609] S. Gopaluni, M. Sherif, N.A. Ahmadouk, Interventions for chronic kidney disease-associated restless legs syndrome, Cochrane Database of Systematic Reviews 2013(9) (2013). | Excluded | Title and abstract | Irrelevant |
| 610] S. Gopaluni, M. Sherif, N.A. Ahmadouk, Interventions for chronic kidney disease-associated restless legs syndrome, The Cochrane database of systematic reviews 11(11) (2016) Cd010690. | Excluded | Title and abstract | Irrelevant |
| 611] P.M. Sinclair, Interventions for chronic kidney disease-associated restless legs syndrome, International journal of evidence-based healthcare 16(3) (2018) 182-184. | Excluded | Title and abstract | Irrelevant |
| 612] S. Gopaluni, M. Sherif, N.A. Ahmadouk, Interventions for chronic kidney disease‐associated restless legs syndrome, Cochrane Database of Systematic Reviews (11) (2016). | Excluded | Title and abstract | Irrelevant |
| 613] L. Luo, K. Zhou, J. Zhang, L. Xu, W. Yin, Interventions for leg cramps in pregnancy, Cochrane Database of Systematic Reviews (12) (2020). | Excluded | Title and abstract | Irrelevant |
| 614] S.C. Palmer, E.Y.M. Chung, D.O. McGregor, F. Bachmann, G.F.M. Strippoli, Interventions for preventing bone disease in kidney transplant recipients, Cochrane Database of Systematic Reviews (10) (2019). | Excluded | Title and abstract | Irrelevant |
| 615] S.R. Lewis, M.W. Pritchard, L.J. Estcourt, S.J. Stanworth, X.L. Griffin, Interventions for reducing red blood cell transfusion in adults undergoing hip fracture surgery: an overview of systematic reviews, Cochrane Database of Systematic Reviews (6) (2023). | Excluded | Title and abstract | Irrelevant |
| 616] N. Glaser, M. Brandner, E. Feistkorn, K. Begemann, H. Desel, Intoxications with food supplements: An underestimated risk?, Clinical Toxicology 56(6) (2018) 565. | Excluded | Title and abstract | Irrelevant |
| 617] V. Markova, R. Hansen, L.L. Thomsen, A. Pinborg, T. Moos, C. Holm, Intravenous iron isomaltoside versus oral iron supplementation for treatment of iron deficiency in pregnancy: protocol for a randomised, comparative, open-label trial, Trials 21(1) (2020) 742. | Excluded | Title and abstract | Irrelevant |
| 618] Irct20141208020249N, Intravenous Vitamin C Supplementation on the Quality of Sleep, Itching,Restlessleg Syndrome in Patients Undergoing Hemodialysis, https://trialsearch.who.int/Trial2.aspx?TrialID=IRCT20141208020249N4 (2018). | Excluded | Title and abstract | Irrelevant |
| 619] T.C. Napier, S. Tedford, G. Ruber, S. Rokosik, The intrinsic rewarding properties of pramipexole in rats: Comparison with l-dopa, effects on risk-taking and associative learning, and alterations following dorsal striatal dopamine deafferentation, Neuropsychopharmacology 36 (2011) S308. | Excluded | Title and abstract | Irrelevant |
| 620] R. Winegar, Investigating Vitamin D Levels and Sleep Quality, 2020. | Excluded | Title and abstract | Irrelevant |
| 621] R. Khalil, W.J. Al-Awaida, H.J. Al-Ameer, Y. Jarrar, A. Imraish, O. Al Bawareed, R. Qawadri, F. Al Madhoon, L. Obeidat, Investigation of <i>ACE rs4646994</i>, <i>MTHFR rs1801133</i> and <i>VDR rs2228570</i> Genotypes in Jordanian Patients with Fibromyalgia Syndrome, Endocrine Metabolic & Immune Disorders-Drug Targets 21(10) (2021) 1920-1928. | Excluded | Title and abstract | Irrelevant |
| 622] J.M. Armson, G.A. Horridge, AN INVESTIGATION OF FACTOR S OF CRUSTACEA, Journal of neurochemistry 11 (1964) 387-95. | Excluded | Title and abstract | Irrelevant |
| 623] Nct, Iron Absorption From Complementary Food Fortificants (CFFs) and Acceptability of CFFs by Beninese Children, https://clinicaltrials.gov/show/NCT01321099 (2011). | Excluded | Title and abstract | Irrelevant |
| 624] P. Chen, M. Totten, Z. Zhang, H. Bucinca, K. Erikson, A. Santamaría, A.B. Bowman, M. Aschner, Iron and manganese-related CNS toxicity: mechanisms, diagnosis and treatment, Expert review of neurotherapeutics 19(3) (2019) 243-260. | Excluded | Title and abstract | Irrelevant |
| 625] J.R. Connor, S.M. Patton, K. Oexle, R.P. Allen, Iron and restless legs syndrome: treatment, genetics and pathophysiology, Sleep medicine 31 (2017) 61-70. | Excluded | Title and abstract | Irrelevant |
| 626] D. Picchietti, Iron deficiency and periodic limb movements in sleep (PLMS), Sleep medicine 10(2) (2009) 265-266. | Excluded | Title and abstract | Irrelevant |
| 627] C. Borgna-Pignatti, M. Marsella, Iron deficiency in infancy and childhood, Pediatric Annals 37(5) (2008) 329-337. | Excluded | Title and abstract | Irrelevant |
| 628] S.T. O'Keeffe, Iron deficiency with normal ferritin levels in restless legs syndrome, Sleep medicine 6(3) (2005) 281-282. | Excluded | Title and abstract | Irrelevant |
| 629] S.T. O'Keeffe, K. Gavin, J.N. Lavan, Iron status and restless legs syndrome in the elderly, Age and ageing 23(3) (1994) 200-3. | Included | Full text | Relationship between vitamins and RLS |
| 630] N. Koleini, J.S. Shapiro, J. Geier, H. Ardehali, Ironing out mechanisms of iron homeostasis and disorders of iron deficiency, Journal of Clinical Investigation 131(11) (2021). | Excluded | Title and abstract | Irrelevant |
| 631] H.A. Bischoff-Ferrari, M. Conzelmann, H.B. Stähelin, W. Dick, M.G. Carpenter, A.L. Adkin, R. Theiler, M. Pfeifer, J.H. Allum, Is fall prevention by vitamin D mediated by a change in postural or dynamic balance?, Osteoporosis international 17(5) (2006) 656‐663. | Excluded | Title and abstract | Irrelevant |
| 632] A. Coutsoudis, D. Sanders, M.A. Dhansay, M.E. van Stuijvenberg, C.S. Benn, Is it time for South Africa to end the routine high-dose vitamin A supplementation programme?, Samj South African Medical Journal 109(12) (2019) 907-910. | Excluded | Title and abstract | Irrelevant |
| 633] A.K. Mandal, T. Abernathy, S.N. Nelluri, V. Stitzel, Is quinine effective and safe in leg cramps?, Journal of clinical pharmacology 35(6) (1995) 588-93. | Excluded | Title and abstract | Irrelevant |
| 634] Y. Sugita, Is restless legs syndrome an entirely neurological disorder?, The European journal of general practice 14(1) (2008) 45-6. | Excluded | Title and abstract | Irrelevant |
| 635] A. Bilgic¸, N. Samanci-Karaman, A. Akman-Karakaş, E. Yilmaz, E. Alpsoy, Is restless legs syndrome associated with psoriasis?, Journal of the European Academy of Dermatology and Venereology 27 (2013) 71-72. | Excluded | Full text | No specific data |
| 636] H. Davis, Is the information out there? What the UKMi new medicines portfolio offers, Pharmaceutical Journal 274(7331) (2005) 19-20. | Excluded | Title and abstract | Irrelevant |
| 637] S. Arzhan, M.E. Roumelioti, M.L. Unruh, Itch and Ache on Dialysis: New Approaches to Manage Uremic Pruritus and Restless Legs, Blood purification 49(1-2) (2020) 222-227. | Excluded | Title and abstract | Irrelevant |
| 638] Anonymous, Joint Congress of European Neurology, Istanbul, TURKEY, May 31 -June 03, 2014, Journal of neurology 261(Suppl. 1) (2014) S4-S488. | Excluded | Title and abstract | Irrelevant |
| 639] N. Alli, E. Gungor, Keratitis, ichthyosis and deafness (KID) syndrome, International Journal of Dermatology 36(1) (1997) 37-39. | Excluded | Title and abstract | Irrelevant |
| 640] Nct, Ketogenic Diet vs ACTH for the Treatment of Children With West Syndrome, https://clinicaltrials.gov/show/NCT05279118 (2022). | Excluded | Title and abstract | Irrelevant |
| 641] J. Peret, D. Fau, A. Girard-Globa, G. Bourdel, R. Jacquot, Kinetic of metabolic process during the nyctohemeral period in the rat given separate feedings, Comptes rendus hebdomadaires des seances de l'Academie des sciences. Serie D: Sciences naturelles 271(23) (1970) 2197-200. | Excluded | Title and abstract | Irrelevant |
| 642] L. Lim, D.S. Dinner, N. Foldvary-Schaefer, Laboratory evaluation in restless legs syndrome, Sleep 28 (2005) A273-A273. | Excluded | Title and abstract | Irrelevant |
| 643] Actrn, Ladies' Exercise Training and Supplement Study: LET'S MOVE To Improve Muscle Health and Function, http://www.who.int/trialsearch/Trial2.aspx?TrialID=ACTRN12617000383369 (2017). | Excluded | Title and abstract | Irrelevant |
| 644] Y. Li, C. Dong, D. Cun, J. Liu, R. Xiang, L. Fang, Lamellar Liquid Crystal Improves the Skin Retention of 3-<i>O</i>-Ethyl-Ascorbic Acid and Potassium 4-Methoxysalicylate <i>In Vitro</i> and <i>In Vivo</i> for Topical Preparation, AAPS PharmSciTech 17(3) (2016) 767-777. | Excluded | Title and abstract | Irrelevant |
| 645] Y. Li, C. Dong, D. Cun, J. Liu, R. Xiang, L. Fang, Lamellar Liquid Crystal Improves the Skin Retention of 3-O-Ethyl-Ascorbic Acid and Potassium 4-Methoxysalicylate In Vitro and In Vivo for Topical Preparation, AAPS PharmSciTech 17(3) (2016) 767-77. | Excluded | Title and abstract | Irrelevant |
| 646] V. Selvaraj, B.R. Gandra, P. Gunasekar, I. Alsakaf, Lamotrigine for the treatment of restless LEG syndrome, CNS Spectrums 21(1) (2016) 77-78. | Excluded | Title and abstract | Irrelevant |
| 647] Ctri, A large study in kids from birth to 18 years of age that have a clot in their lungs, limbs, to test a new drug (edoxaban) compared to current drugs used for treatment and to prevent further clots in your body, https://trialsearch.who.int/Trial2.aspx?TrialID=CTRI/2018/01/011249 (2018). | Excluded | Title and abstract | Irrelevant |
| 648] Ctri, A large study in kids from birth to 18 years of age that have a documented clot in their lung or limbs, to test a new drug (edoxaban) used for treatment or the prevention of recurrent clots in your body, (2019). | Excluded | Title and abstract | Irrelevant |
| 649] X. Meng, C. Liu, C. Cao, Z. Zheng, Q. Su, Y. Liu, L-ascorbyl palmitate modify the crystallization behavior of palm oil: Mechanism and application, Lwt-Food Science and Technology 122 (2020). | Excluded | Title and abstract | Irrelevant |
| 650] Y. Li, L. Guo, W. Lu, Laser ablation-enhanced transdermal drug delivery, Photonics and Lasers in Medicine 2(4) (2013) 315-322. | Excluded | Title and abstract | Irrelevant |
| 651] K. Hidaka, M. Adachi, R. Kuroki, S. Tokai, K. Akaji, Y. Tsuda, Y. Kiso, Lead optimization of allophenylnorstatine-containing inhibitors as therapeutic drug and application to peptidomimic protease probe, Journal of Peptide Science 18 (2012) S34-S35. | Excluded | Title and abstract | Irrelevant |
| 652] W. Blättler, H. Partsch, Leg compression and ambulation is better than bed rest for the treatment of acute deep venous thrombosis, International angiology 22(4) (2003) 393‐400. | Excluded | Title and abstract | Irrelevant |
| 653] S. Ayres, Jr., R. Mihan, Leg cramps (systremma0 and "restless legs" syndrome. Response to vitamin E (tocopherol), California medicine 111(2) (1969) 87-91. | Excluded | Full text | Without control |
| 654] J.G. Hensley, Leg Cramps and Restless Legs Syndrome During Pregnancy, Journal of Midwifery and Women's Health 54(3) (2009) 211-218. | Excluded | Title and abstract | Irrelevant |
| 655] Y.C. Terrie, Leg cramps: A sign of underlying illness?, 2017. | Excluded | Title and abstract | Irrelevant |
| 656] J.D. Riley, S.J. Antony, Leg cramps: differential diagnosis and management, Am Fam Physician 52(6) (1995) 1794-8. | Excluded | Title and abstract | Irrelevant |
| 657] M. Marano, V. Pozzilli, A. Magliozzi, G. Tabacco, A.M. Naciu, A. Palermo, V. Di Lazzaro, Leg restlessness and hyperparathyroidism in Parkinson's disease, a further clue to RLS pathogenesis?, Front Neurol 14 (2023) 1113913. | Included | Full text | Relationship between vitamins and RLS |
| 658] L.Z. Almeida, Legacies of Early-Life Experiences on Individual, Cohort, and Population Performance of Lake Erie Walleye, 2021. | Excluded | Title and abstract | Irrelevant |
| 659] M. Li, D. Huang, Y. Zhou, J. Zhang, X. Lin, J. Chen, The legacy effects of PM<sub>2.5</sub> depositon on <i>Nerium Oleande</i>r L, Chemosphere 281 (2021). | Excluded | Title and abstract | Irrelevant |
| 660] A.M. Diephnger, F.S. Kundt, M. Kloesch, Lege artis eye coverage performed during gynecological/urological laser surgery. Results of a survey of nursing experts, Anaesthesist 69(2) (2020) 117-121. | Excluded | Title and abstract | Irrelevant |
| 661] S. Sevim, M. Demirkiran, M. Terzi, N. Yuceyar, B. Tasdelen, E. Idiman, M. Kurtuncu, C. Boz, D. Tuncel, R. Karabudak, A. Siva, C. Ozcan, M. Neyal, B.K. Goksel, M. Balal, S. Sen, O. Ekmekci, D. Kara, S. Turkish Multiple Sclerosis, Let's raise the awareness of MS specialists concerning the frequency and impact of RLS in MS and consequently the life quality of patients with MS: Striking results of the 'RELOMS-T' Study, Neurology 90 (2018). | Excluded | Title and abstract | Irrelevant |
| 662] N.K. Sethi, Letter re: Practice guideline summary: Treatment of restless legs syndrome in adults: Report of the Guideline Development, Dissemination, and Implementation Subcommittee of the American Academy of Neurology, Neurology 88(24) (2017) 2337. | Excluded | Title and abstract | Irrelevant |
| 663] T.V. Taylor, A.T. Raftery, J.B. Elder, C. Loveday, I.W. Dymock, A.C. Gibbs, J. Jeacock, S.B. Lucas, M.A. Pell, Leucocyte ascorbate levels and postoperative deep venous thrombosis, British journal of surgery 66(8) (1979) 583‐585. | Excluded | Title and abstract | Irrelevant |
| 664] M. Nyberg, J.R. Blackwell, R. Damsgaard, A.M. Jones, Y. Hellsten, S.P. Mortensen, Lifelong physical activity prevents an age-related reduction in arterial and skeletal muscle nitric oxide bioavailability in humans, Journal of Physiology-London 590(21) (2012) 5361-5370. | Excluded | Title and abstract | Irrelevant |
| 665] I. Delfino, Light scattering methods for tracking gold nanoparticles aggregation induced by biotin-neutravidin interaction, Biophysical Chemistry 177 (2013) 7-13. | Excluded | Title and abstract | Irrelevant |
| 666] G. Wollensak, C. Mazzotta, T. Kalinski, S. Sel, Limbal and Conjunctival Epithelium After Corneal Cross-linking Using Riboflavin and UVA, Cornea 30(12) (2011) 1448-1454. | Excluded | Title and abstract | Irrelevant |
| 667] K. Archontogeorgis, E. Nena, P. Steiropoulos, Linking Vitamin D and Sleep, 2020. | Excluded | Title and abstract | Irrelevant |
| 668] R. Böttger, G. Pauli, P.H. Chao, N. Al Fayez, L. Hohenwarter, S.D. Li, Lipid-based nanoparticle technologies for liver targeting, Advanced Drug Delivery Reviews 154-155 (2020) 79-101. | Excluded | Title and abstract | Irrelevant |
| 669] N. Tachibana, Living with Restless Legs Syndrome/Willis-Ekbom Disease, Sleep Medicine Clinics 10(3) (2015) 359-367. | Excluded | Title and abstract | Irrelevant |
| 670] K. Uusi-Rasi, R. Patil, S. Karinkanta, K. Tokola, P. Kannus, C. Lamberg-Allardt, H. Sievanen, Long-term effects of vitamin D and multimodal exercise on prevention of injurious falls in older women. A 2-year follow-up after intervention, Journal of bone and mineral research 31(Supplement 1) (2017). | Excluded | Title and abstract | Irrelevant |
| 671] N. Simakajornboon, L. Kheirandish-Gozal, D. Sharon, L. Murry, M. Abraham, A long-term follow-up study of periodic limb movement disorders in children after iron therapy, Sleep 29 (2006) A76-A77. | Excluded | Title and abstract | Irrelevant |
| 672] M.T. Cáceres-Redondo, F. Carrillo, M.J. Lama, I. Huertas-Fernández, L. Vargas-González, M. Carballo, P. Mir, Long-term levodopa/carbidopa intestinal gel in advanced Parkinson's disease, Journal of neurology 261(3) (2014) 561-569. | Excluded | Title and abstract | Irrelevant |
| 673] S. Chokroverty, Long-term management issues in restless legs syndrome, Movement Disorders 26(8) (2011) 1378-1385. | Excluded | Title and abstract | Irrelevant |
| 674] A look back at pharmaceuticals in 2006: Aggressive advertising cannot hide the absence of therapeutic advances, Prescrire international 16(88) (2007) 80-86. | Excluded | Title and abstract | Irrelevant |
| 675] S. Manorenj, S. Manorenj, S. Jawalkar, B. Naaz, A. Kumar, M.S.U. Bilal, Lower dose of oral, direct thrombin inhibitor, a novel therapy in cerebral venous sinus thrombosis - A case series study, Annals of Indian Academy of Neurology 22(SUPPL 1) (2019) S38‐S39. | Excluded | Title and abstract | Irrelevant |
| 676] M.P. Barros, E.J.H. Bechara, Luciferase and urate may act as antioxidant defenses in larval <i>Pyrearinus termitilluminans</i> (Elateridae: Coleoptera) during natural development and upon 20-hydroxyecdysone treatment, Photochemistry and Photobiology 71(5) (2000) 648-654. | Excluded | Title and abstract | Irrelevant |
| 677] C. Trenkwalder, M. Canelo, Management of augmentatiion in patients with restless legs syndrome, Sleep 28 (2005) A278-A278. | Excluded | Title and abstract | Irrelevant |
| 678] K. Czapla, Management of chronic renal insufficiency in frail older patients who are unfit for renal replacement therapy, Reviews in Clinical Gerontology 13(1) (2003) 25-37. | Excluded | Title and abstract | Irrelevant |
| 679] V.S. Ram, A.Z. Fenves, Management of hypertension in hemodialysis patients, Current Hypertension Reports 11(4) (2009) 292-298. | Excluded | Title and abstract | Irrelevant |
| 680] D. Santini, G. Armento, R. Giusti, M. Ferrara, C. Moro, F. Fulfaro, P. Bossi, F. Arena, C.I. Ripamonti, Management of orphan symptoms: ESMO Clinical Practice Guidelines for diagnosis and treatment †, ESMO Open 5(6) (2020). | Excluded | Title and abstract | Irrelevant |
| 681] R. Moretti, P. Caruso, M. Tecchiolli, S. Gazzin, C. Tiribelli, Management of restless legs syndrome in chronic liver disease: A challenge for the correct diagnosis and therapy, World journal of hepatology 10(3) (2018) 379-387. | Excluded | Title and abstract | Irrelevant |
| 682] C. Goldstein, Management of Restless Legs Syndrome/Willis-Ekbom Disease in Hospitalized and Perioperative Patients, Sleep Medicine Clinics 10(3) (2015) 303-310. | Excluded | Title and abstract | Irrelevant |
| 683] K.B. Im, S. Strader, M.E. Dyken, Management of sleep disorders in stroke, Current Treatment Options in Neurology 12(5) (2010) 379-395. | Excluded | Title and abstract | Irrelevant |
| 684] X.Z. Yuan, R.M. Yang, X.P. Wang, Management perspective of wilson’s disease: Early diagnosis and individualized therapy, Current Neuropharmacology 19(4) (2021) 465-485. | Excluded | Title and abstract | Irrelevant |
| 685] C. Garbazza, M. Manconi, Management Strategies for Restless Legs Syndrome/Willis-Ekbom Disease During Pregnancy, Sleep medicine clinics 13(3) (2018) 335-348. | Excluded | Title and abstract | Irrelevant |
| 686] C. Morlidge, T. Richards, Managing chronic renal disease, Pharmaceutical Journal 266(7147) (2001) 655-657. | Excluded | Title and abstract | Irrelevant |
| 687] S.Y. Lim, Managing the peripheral problems in PD, Journal of Parkinson's Disease 9(1) (2019) 42. | Excluded | Title and abstract | Irrelevant |
| 688] I.J. Wang, S.-Y. Chu, C.-Y. Wang, P.-J. Wang, W.-L. Hwu, Maple syrup urine disease presenting with neonatal status epilepticus: Report of one case, Acta Paediatrica Taiwanica 44(4) (2003) 246-248. | Excluded | Title and abstract | Irrelevant |
| 689] E.F. Ramadan, M. Grisdale, M. Morais, Maternal Vitamin B(12) Levels During Pregnancy and Their Effects on Maternal Neurocognitive Symptoms: A Systematic Review, Journal of obstetrics and gynaecology Canada : JOGC = Journal d'obstetrique et gynecologie du Canada : JOGC 44(4) (2022) 390-394.e3. | Excluded | Title and abstract | Irrelevant |
| 690] A. Sert, C. Kilicaslan, E.S. Solak, S. Arslan, Mean platelet volume in children with Reye-like syndrome, Platelets 26(3) (2015) 212-215. | Excluded | Title and abstract | Irrelevant |
| 691] L. Boateng, A. Ohemeng, M. Asante, M. Steiner-Asiedu, Measuring blood retinol concentrations of infants fed with complementary foods fortified with Moringa Oleifera leaf powder-a pilot study, Annals of nutrition & metabolism 71 (2017) 655. | Excluded | Title and abstract | Irrelevant |
| 692] J.M. Crowther, A. Sieg, P. Blenkiron, C. Marcott, P.J. Matts, J.R. Kaczvinsky, A.V. Rawlings, Measuring the effects of topical moisturizers on changes in stratum corneum thickness, water gradients and hydration in vivo, British journal of dermatology 159(3) (2008) 567‐577. | Excluded | Title and abstract | Irrelevant |
| 693] M. Cygler, Medec 2005: Allergy, antibiotherapy, vaccines, obesity, restless legs syndrome, vitamin D and fluorine, chronic diseases, association with non-lucrative goal, Journal de Pediatrie et de Puericulture 18(5) (2005) 243-258. | Excluded | Title and abstract | Irrelevant |
| 694] A. Kai, E.L. Speight, J.A.A. Langtry, Median lip fissure of the upper lip: First report of surgical treatment by excision and Z-plasty repair, British Journal of Dermatology 171 (2014) 86. | Excluded | Title and abstract | Irrelevant |
| 695] J. Yang, J. Guo, Y. Tang, L. Huang, J. Wiley, Z. Zhou, R. Whittemore, The mediating effect of coping styles and self‐efficacy between perceived stress and satisfaction with QOL in Chinese adolescents with type 1 diabetes, Journal of advanced nursing (john wiley & sons, inc.) 75(7) (2019) 1439‐1449. | Excluded | Title and abstract | Irrelevant |
| 696] B.L. Langdahl, T. Harsløf, Medical treatment of osteoporotic vertebral fractures, Therapeutic Advances in Musculoskeletal Disease 3(1) (2011) 17-29. | Excluded | Title and abstract | Irrelevant |
| 697] J. Bajramovic Fejzic, S.E. Tett, Medication management reviews for people from the former Yugoslavia now resident in Australia, Pharmacy World and Science 26(5) (2004) 271-276. | Excluded | Title and abstract | Irrelevant |
| 698] J.F. Pagel, Medications and their effects on sleep, Primary Care - Clinics in Office Practice 32(2) (2005) 491-509. | Excluded | Title and abstract | Irrelevant |
| 699] J. Paccalin, F.J. Traissac, C. Wone, J. Cardinaud, Medicinal abuse and potassium, Therapie 28(6) (1973) 1143-52. | Excluded | Title and abstract | Irrelevant |
| 700] Nct, A Mental Health Intervention for a Community Program Called the PowerObesity, https://clinicaltrials.gov/show/NCT05605028 (2022). | Excluded | Title and abstract | Irrelevant |
| 701] S. Yan, Z. Tian, H. Zhao, C. Wang, Y. Pan, N. Yao, Y. Guo, H. Wang, B. Li, W. Cui, A meta-analysis: Does vitamin D play a promising role in sleep disorders?, Food Science & Nutrition 8(10) (2020) 5696-5709. | Excluded | Title and abstract | Irrelevant |
| 702] B. Yang, H. Yin, J. Wang, J. Gan, J. Li, R. Han, M. Pei, L. Song, H. Yang, A metabolic biomarker panel of restless legs syndrome in peritoneal dialysis patients, Metabolomics : Official journal of the Metabolomic Society 18(11) (2022). | Excluded | Title and abstract | Irrelevant |
| 703] D.W. Sickles, T.G. Oblak, Metabolic variation among alpha-motoneurons innervating different muscle-fiber types. I. Oxidative enzyme activity, Journal of neurophysiology 51(3) (1984) 529-37. | Excluded | Title and abstract | Irrelevant |
| 704] C. Zhou, H.M. Jia, Y.T. Liu, M. Yu, X. Chang, Y.M. Ba, Z.M. Zou, Metabolism of glycerophospholipid, bile acid and retinol is correlated with the early outcomes of autoimmune hepatitis, Molecular bioSystems 12(5) (2016) 1574-85. | Excluded | Title and abstract | Irrelevant |
| 705] J.S. Miller, L. Rodriguez-Saona, K.V. Hackshaw, Metabolomics in central sensitivity syndromes, Metabolites 10(4) (2020). | Excluded | Title and abstract | Irrelevant |
| 706] C. Petra, R. Jan, L. Iva, K. Martina, F. Jorga, S. Jiri, Methotrexate-induced ulcerations in a patient with psoriasis, Journal of the American Academy of Dermatology 81(4) (2019) AB19. | Excluded | Title and abstract | Irrelevant |
| 707] N.W. Hodgson, M.I. Waly, M.S. Trivedi, V.A. Power-Charnitsky, R.C. Deth, Methylation-related metabolic effects of D4 dopamine receptor expression and activation, Translational psychiatry 9(1) (2019) 295. | Excluded | Title and abstract | Irrelevant |
| 708] F. Sarayloo, A. Dionne-Laporte, H. Catoire, D. Rochefort, G. Houle, J.P. Ross, F. Akcimen, R.D.B. Oliveira, G. Turecki, P.A. Dion, G.A. Rouleau, Mineral absorption is an enriched pathway in a brain region of restless legs syndrome patients with reduced <i>MEIS1</i> expression, PloS one 14(11) (2019). | Excluded | Full text | No relevant outcome |
| 709] F. Sarayloo, A. Dionne-Laporte, H. Catoire, D. Rochefort, G. Houle, J.P. Ross, F. Akçimen, R. Barros Oliveira, G. Turecki, P.A. Dion, G.A. Rouleau, Mineral absorption is an enriched pathway in a brain region of restless legs syndrome patients with reduced MEIS1 expression, PloS one 14(11) (2019) e0225186. | Excluded | Title and abstract | Duplication |
| 710] A. Fois, A. Chatrenet, E. Cataldo, F. Lippi, A. Kaniassi, J. Vigreux, L. Froger, E. Mongilardi, I. Capizzi, M. Biolcati, E. Versino, G.B. Piccoli, Moderate protein restriction in advanced CKD: A feasible option in an elderly, high-comorbidity population. a stepwise multiple-choice system approach, Nutrients 11(1) (2019). | Excluded | Title and abstract | Irrelevant |
| 711] S. Lee, W. Jung, S. Eom, H.D. Yeom, H.-D. Park, J.H. Lee, Molecular Regulation of Betulinic Acid on α3β4 Nicotinic Acetylcholine Receptors, Molecules 26(9) (2021). | Excluded | Title and abstract | Irrelevant |
| 712] Y. Honda, A monozygotic twin pair completely discordant for narcolepsy, with sleep deprivation as a possible precipitating factor, Sleep and Biological Rhythms 1(2) (2003) 147-149. | Excluded | Title and abstract | Irrelevant |
| 713] K. Shin, S.H. Park, W. Park, H.J. Baek, Y.J. Lee, S.W. Kang, J.Y. Choe, W.H. Yoo, Y.B. Park, J.S. Song, et al., Monthly Oral Ibandronate Reduces Bone Loss in Korean Women With Rheumatoid Arthritis and Osteopenia Receiving Long-term Glucocorticoids: a 48-week Double-blinded Randomized Placebo-controlled Investigator-initiated Trial, Clinical therapeutics 39(2) (2017) 268‐278.e2. | Excluded | Title and abstract | Irrelevant |
| 714] G.T. Duval, A.-M. Schott, D. Sanchez-Rodriguez, F.R. Herrmann, C. Annweiler, Month-of-Birth Effect on Muscle Mass and Strength in Community-Dwelling Older Women: The French EPIDOS Cohort, Nutrients 14(22) (2022). | Excluded | Title and abstract | Irrelevant |
| 715] Nct, Moringa Oleifera (Drumstick Leaves) for Improving Haemoglobin, Vitamin A Status and Underweight Among Adolescent Girls in Rural Bangladesh: a Quasi-experimental Study, https://clinicaltrials.gov/show/NCT04156321 (2019). | Excluded | Title and abstract | Irrelevant |
| 716] N. Djokanovic, F. Garcia-Bournissen, G. Koren, MOTHERISK ROUNDS: Medications for Restless Legs Syndrome in Pregnancy, Journal of Obstetrics and Gynaecology Canada 30(6) (2008) 505-507. | Excluded | Title and abstract | Irrelevant |
| 717] R.A. Patel, D.A. Hall, S. Eichenseer, M. Bailey, Movement Disorders and Hematologic Diseases, Movement Disorders Clinical Practice 8(2) (2021) 193-207. | Excluded | Title and abstract | Irrelevant |
| 718] S.M. Kranick, E.M. Mowry, A. Colcher, S. Horn, L.I. Golbe, Movement disorders and pregnancy: A review of the literature, Movement Disorders 25(6) (2010) 665-671. | Excluded | Title and abstract | Irrelevant |
| 719] S. Hamed, K. Mohamed, S. Abd Elhameed, E. Moussa, H. Abozaid, A. Lang, A. Mohamed, J. Moussa, Movement Disorders Due to Selective Basal Ganglia Lesions with Uremia, Canadian Journal of Neurological Sciences 47(3) (2020) 350-365. | Excluded | Title and abstract | Irrelevant |
| 720] J.M. Miyasaki, A. Aldakheel, Movement disorders in pregnancy, CONTINUUM Lifelong Learning in Neurology 20(1) (2014) 148-161. | Excluded | Title and abstract | Irrelevant |
| 721] M.S.A. Smith, M.L. Evatt, Movement disorders in pregnancy, Neurologic Clinics 22(4 SPEC. ISS.) (2004) 783-798. | Excluded | Title and abstract | Irrelevant |
| 722] Y. Wang, Y.L. Gao, J. Wang, F. Li, J.X. Liu, MSGCA: Drug-Disease Associations Prediction Based on Multi-Similarities Graph Convolutional Autoencoder, IEEE Journal of Biomedical and Health Informatics 27(7) (2023) 3686-3694. | Excluded | Title and abstract | Irrelevant |
| 723] ChiCtr, Multi center clinical intervention study of HMB combined with Lactobacillus plantarum p-8 to accelerate the late rehabilitation of ICU-AW patients, http://www.who.int/trialsearch/Trial2.aspx?TrialID=ChiCTR2000040738 (2020). | Excluded | Title and abstract | Irrelevant |
| 724] E.J. Sirot, S. Harenberg, P. Vandel, C.A.M. Lima, P. Perrenoud, K. Kemmerling, D.F. Zullino, H. Hilleret, S. Crettol, M. Jonzier-Perey, K.P. Golay, M. Brocard, C.B. Eap, P. Baumann, Multicenter study on the clinical effectiveness, pharmacokinetics, and pharmacogenetics of mirtazapine in depression, Journal of Clinical Psychopharmacology 32(5) (2012) 622-629. | Excluded | Title and abstract | Irrelevant |
| 725] L. Ferini-Strambi, M. Manconi, R.S.G.T. Italian, Multicentre case-control study on restless legs syndrome in multiple sclerosis: The rems study, Sleep 31 (2008) A283-A283. | Included | Full text | Relationship between vitamins and RLS |
| 726] D. Cui, Multifunctional nanoprobes for targeted imaging and photodynamic therapy of gastric cancer, Photonics and Lasers in Medicine 4(4) (2015) 347-349. | Excluded | Title and abstract | Irrelevant |
| 727] Nct, Multimodal Prehabilitation for Lung Cancer Surgery, https://clinicaltrials.gov/ct2/show/NCT05955248 (2023). | Excluded | Title and abstract | Irrelevant |
| 728] Y. Li, K.L. Munger, S. Batool-Anwar, A. Ascherio, X. Gao, Multiple sclerosis and restless legs syndrome in middle-age women, Sleep medicine 12 (2011) S84. | Excluded | Full text | No relevant outcome |
| 729] T.J. Kuzniar, M.H. Silber, Multiple Skeletal Injuries Resulting From Uncontrolled Restless Legs Syndrome, Journal of Clinical Sleep Medicine 3(1) (2007) 60-61. | Excluded | Title and abstract | Irrelevant |
| 730] Multiple-choice questions-preferred responses, CONTINUUM Lifelong Learning in Neurology 19(4) (2013) 1122-1143. | Excluded | Title and abstract | Irrelevant |
| 731] W. Wirths, Muscle function tests using dynamometer measurements under the influence of deficient activity and restricted intake of calories and proteins, Internationale Zeitschrift fur angewandte Physiologie, einschliesslich Arbeitsphysiologie 27(2) (1969) 116-32. | Excluded | Title and abstract | Irrelevant |
| 732] H.C. Janssen, M.M. Samson, H.J. Verhaar, Muscle strength and mobility in vitamin D-insufficient female geriatric patients: a randomized controlled trial on vitamin D and calcium supplementation, Aging clinical and experimental research 22(1) (2010) 78‐84. | Excluded | Title and abstract | Irrelevant |
| 733] H.J.J. Verhaar, M.M. Samson, P.A.F. Jansen, P.L. de Vreede, J.W. Manten, S.A. Duursma, Muscle strength, functional mobility and vitamin D in older women, Aging-Clinical and Experimental Research 12(6) (2000) 455-460. | Excluded | Title and abstract | Irrelevant |
| 734] Y. Chen, Y. Liang, H. Guo, K. Meng, J. Qiu, D. Benardot, Muscle-Related Effect of Whey Protein and Vitamin D<sub>3</sub> Supplementation Provided before or after Bedtime in Males Undergoing Resistance Training, Nutrients 14(11) (2022). | Excluded | Title and abstract | Irrelevant |
| 735] N.K. Banerji, V.A.F. Martin, Myelo optico neuropathy following quinine poisoning, J.IRISH MED.ASS. 67(2) (1974) 46-47. | Excluded | Title and abstract | Irrelevant |
| 736] T. Paiva, T.R. Pinto, V. Ramos, M. Chaves, P.S. Nunes, Narcolepsy cataplexy atypical associations: Lucid dreaming and Diogenes syndrome, Journal of Sleep Research 23 (2014) 297-298. | Excluded | Title and abstract | Irrelevant |
| 737] A. Pollmann, Natura sanat, KIM - Komplementare und Integrative Medizin, Artztezeitschrift fur Naturheilverfahren 48(11) (2007) 6. | Excluded | Title and abstract | Irrelevant |
| 738] M. Tang, J. Ge, K.X. Sun, B. Cui, Y. Guan, J.W. Wang, A network meta-analysis of the effectiveness and safety of drugs for restless legs syndrome in dialysis patients, Semin Dial 35(4) (2022) 293-306. | Excluded | Title and abstract | Irrelevant |
| 739] F.J. Jiménez-Jiménez, H. Alonso-Navarro, E. García-Martín, J.A.G. Agúndez, Neurochemical features of idiopathic restless legs syndrome, Sleep Med Rev 45 (2019) 70-87. | Excluded | Title and abstract | Irrelevant |
| 740] H.M. Schipper, Neurodegeneration with brain iron accumulation - Clinical syndromes and neuroimaging, Biochimica et Biophysica Acta - Molecular Basis of Disease 1822(3) (2012) 350-360. | Excluded | Title and abstract | Irrelevant |
| 741] I.A. Malaty, M.C. Lansang, M.S. Okun, Neuroendocrinologic considerations in parkinson disease and other movement disorders, CONTINUUM Lifelong Learning in Neurology 15(2) (2009) 125-147. | Excluded | Title and abstract | Irrelevant |
| 742] S.A. Hamed, Neurologic conditions and disorders of uremic syndrome of chronic kidney disease: presentations, causes, and treatment strategies, Expert Review of Clinical Pharmacology 12(1) (2019) 61-90. | Excluded | Title and abstract | Irrelevant |
| 743] M.I. Botez, M. Cadotte, R. Beaulieu, L.P. Pichette, C. Pison, Neurologic disorders responsive to folic acid therapy, Can Med Assoc J 115(3) (1976) 217-23. | Excluded | Title and abstract | Irrelevant |
| 744] M.A. Rizzo, F. Frediani, A. Granata, B. Ravasi, D. Cusi, M. Gallieni, Neurological complications of hemodialysis: State of the art, Journal of Nephrology 25(2) (2012) 170-182. | Excluded | Title and abstract | Irrelevant |
| 745] S.M. Scott, Neuromuscular characteristics of multiple sclerosis patients, (2008). | Excluded | Title and abstract | Irrelevant |
| 746] K. Al-Hayk, T.E. Bertorini, Neuromuscular complications in uremics: A review, The neurologist 13(4) (2007) 188-196. | Excluded | Title and abstract | Irrelevant |
| 747] A.V. Krishnan, B.A. Pussell, M.C. Kiernan, Neuromuscular Disease in the Dialysis Patient: An Update for the Nephrologist, Seminars in Dialysis 22(3) (2009) 267-278. | Excluded | Title and abstract | Irrelevant |
| 748] J. Massa, B. Beaber, Y. Bronstein, Neuropathic pain as the presenting symptom of adult-onset Alexander's Disease, Neurology 88(16) (2017). | Excluded | Title and abstract | Irrelevant |
| 749] L. Lim, R.W. Shields, Neurophysiologic characteristics of Restless Legs Syndrome, Sleep 27 (2004) 306-306. | Excluded | Title and abstract | Irrelevant |
| 750] J. Durlach, P. Bac, V. Durlach, M. Bara, A. Guietbara, Neurotic, neuromuscular and autonomic nervous form of magnesium imbalance, Magnesium Research 10(2) (1997) 169-195. | Excluded | Title and abstract | Irrelevant |
| 751] J.S. Bryans, D.C. Horwell, C.O. Kneen, D.J. Wustrow, T.R. Bellioti, T. Capiris, T.R. Belliotti, J.S. Brgans, New 4(3)-substituted-4(3)-aminomethyl-(thio)pyran and -piperidine gabapentin analogs and their salts, Warner Lambert Co; Pfizer Inc; Warner Lambert Co Llc. | Excluded | Title and abstract | Irrelevant |
| 752] S.R. Akireddy, B.S. Bhatti, T.J. Cuthbertson, G.M. Dull, C.H. Miller, J.P. Mitchener, J.A. Munoz, P.A. Otten, B. Bhatti, T. Cuthbertson, G. Dull, C. Miller, J. Mitchener, J. Munoz, P. Otten, A. Munoz Yu, Y.Y.P. Mitchener, T.S. Cuthbertson, J.J.P. Mitchener, S. Rao Akireddy, A.P. Otten, A.J. Munoz, P.J.J. Mitchener, M.G. Dull, J.T. Cuthbertson, S.B. Bhatti, H.C. Miller, G.H. Miller, New acid addition salt, amorphous form or polymorphic form of (R)-5-((E)-2-pyrrolidin-3-ylvinyl)pyrimidine is useful for treating pain, inflammation, or CNS disorder, Targacept Inc; Oyster Point Pharma Inc. | Excluded | Title and abstract | Irrelevant |
| 753] T.R. Belliotti, J.S. Bryans, T. Capiris, D.C. Horwell, C.O. Kneen, D.J. Wustrow, New amines for treating e.g. epilepsy, hypokinesia, cranial disorders and neurodegenerative disorders, Warner Lambert Co; Pfizer Inc; Belliotti T R; Bryans J S; Capiris T; Horwell D C; Kneen C O; Wustrow D J; Warner Lambert Co Llc. | Excluded | Title and abstract | Irrelevant |
| 754] F.M. Martin, C.J. Flynn, S.J. Richards, F.M.L. Martin, C.J.L.A. Flynn, S.J.L. Richards, New azabicycloalkane compounds are nicotinic acetylcholine receptor agonists used for treating e.g. anxiety, panic disorder, depression, bipolar disorder, autism, sleep disorders, Parkinson's disease and schizophrenia, Lilly & Co Eli; Martin F M; Flynn C J; Richards S J; Lilly&Co Eli. | Excluded | Title and abstract | Irrelevant |
| 755] J.D. Bortz, New composition comprises iron, useful for preventing, stabilizing, reversing or treating disorders related to iron deficiency, Drugtech Corp. | Excluded | Title and abstract | Irrelevant |
| 756] C. Hendrix, K. Hendeurikseu, New compound comprising ester formed from inositol and niacin, useful e.g. for treating dyslipidemia, hypercholesterolemia, hyperlipidemia, hypertriglyceridemia and peripheral vascular diseases, Concourse Health Sci Llc; Hendrix C. | Excluded | Title and abstract | Irrelevant |
| 757] J.S. Bryans, D.C. Blackemore, S.C. Williams, D.C. Blakemore, New cyclic amino derivatives useful in the treatment of neurological conditions e.g. epilepsy and pain, Warner Lambert Co; Bryans J S; Blakemore D C; Williams S C. | Excluded | Title and abstract | Irrelevant |
| 758] S.K. Kakkos, E. Bouskela, A. Jawien, A.N. Nicolaides, New data on chronic venous disease: A new place for Cyclo 3® Fort, International Angiology 37(1) (2018) 85-92. | Excluded | Title and abstract | Irrelevant |
| 759] M.P. Allen, J.W. Coe, S. Liras, C.J. Odonnell, B.T. Oneill, C.J. O'Donnell, B.T. O'Neill, New heteroaryl fused azapolycyclic compounds useful in the treatment of disease mediated by neuronal nicotinic acetylcholine specific receptor sites e.g. inflammatory bowel disease and ulcerative colitis, Pfizer Prod Inc; Pfizer Inc. | Excluded | Title and abstract | Irrelevant |
| 760] F.I. Carroll, B.E. Blough, H.A. Navarro, S.W. Mascarella, A.Z. Muresan, M.I. Damaj, R.J. Lukas, B. Blough, H. Navarro, R.I. Lukas, New hydroxybupropion analogues are monoamine reuptake inhibitors useful e.g. to treat depression, obesity, bipolar disorder, attention deficit disorder, attention deficit/hyperactivity disorder, schizophrenia and schizoaffective disorder, Res Triangle Inst; Univ Virginia Commonwealth; Dignity Health Dba St Joseph's Hospital; Dignity Health Dba St Josephs Hospital; Dignity Health. | Excluded | Title and abstract | Irrelevant |
| 761] J.B. Sampson, L. Baird, B. Otterud, M. Leppert, A New Locus for Familial Restless Legs Syndrome on Chromosome 21 in a Large Utah Kindred, Neurology 74(9) (2010) A192-A192. | Excluded | Title and abstract | Irrelevant |
| 762] M. Guo, L.T. Wang, X. Wu, W. Xu, J.H. Yang, A new method for the determination of nucleic acid using an Eu3+- nicotinic acid complex as a resonance light scattering probe, Molecules 14(1) (2008) 10-8. | Excluded | Title and abstract | Irrelevant |
| 763] M. Guo, L.T. Wang, X. Wu, W. Xu, J.H. Yang, A new method for the determination of nucleic acid using an Eu3+- nicotinic acid complex as a resonance light scattering probe, Molecules (Basel, Switzerland) 14(1) (2009) 10-18. | Excluded | Title and abstract | Irrelevant |
| 764] M. Guo, L.-T. Wang, X. Wu, W. Xu, J.-H. Yang, A New Method for the Determination of Nucleic Acid Using an Eu<SUP>3+</SUP>-nicotinic Acid Complex as a Resonance Light Scattering Probe, Molecules 14(1) (2009) 10-18. | Excluded | Title and abstract | Irrelevant |
| 765] J.J. Hill, N.M. Wolfman, New pharmaceutical compositions having a growth and differentiation factor-8-associated serum protein 1, useful for treating muscular dystrophy, amyotrophic lateral sclerosis, diabetes, obesity and bone disorders, Wyeth. | Excluded | Title and abstract | Irrelevant |
| 766] C. Caccia, E. La Porta, S. Maestroni, P. Melloni, C. Sabido David, P. Salvati, New prolinamide derivatives are sodium and/or calcium channel blockers or selective monoamine oxidase B inhibitor useful to treat e.g. neuropathic pain, neuralgia, osteoarthritis, migraine, cognitive disorders and degenerative dementia, Newron Pharm Spa. | Excluded | Title and abstract | Irrelevant |
| 767] M. Bencherif, B.S. Bhatti, G.M. Dull, B. Fordham-Meier, J. Genus, J.W. James, K. Jordan, P.M. Lippiello, J.A. Munoz, M.G. Williams, J.A. Young, New solid acid addition salt of (2S)-(4E)-N-methyl-5-(3-(5-methoxypyridin)yl)-4-penten-2-amine, and polymorphic form and Form 3 of phosphate of the amine useful in the treatment of central nervous system disorders e.g. stroke, and pain, Targacept Inc. | Excluded | Title and abstract | Irrelevant |
| 768] P. Cole, New therapeutic directions: Presentations from the Movement Disorder Society's 13th International Congress of Parkinson's Disease and Movement Disorders, Drugs of the Future 34(8) (2009) 681-692. | Excluded | Title and abstract | Irrelevant |
| 769] S.J. Bamford, R.J. Gillespie, R.S. Todd, S. Bamford, R. Gillespie, R. Todd, J. Bamford Samantha, J. Gillespie Roger, S. Todd Richard, New triazolo(4,5-d)pyramidine derivatives useful for treating or preventing e.g. purine receptor hyper functioning, movement disorders, Parkinson's disease, Huntington's chorea, Wilson's disease, dyskinesias, neurological disorders, tremor, Vernalis R & D Ltd; Vernalis Res Ltd; Vernalis; Ligand Uk Dev Ltd. | Excluded | Title and abstract | Irrelevant |
| 770] S. Razdan, K. Pandita, V. Chopra, S. Koul, New-onset headache in an elderly man with uremia that improved only after correction of hyperphosphatemia ("uremic headache"): A case report, Journal of Medical Case Reports 5 (2011). | Excluded | Title and abstract | Irrelevant |
| 771] O. Mohiuddin, A.A. Khan, S.M.I. Shah, M.D.Z. Malick, S.F. Memon, S. Jalees, F. Yasmin, New-onset restless leg syndrome in a COVID-19 patient: a case report with literature review, The Pan African medical journal 38 (2021) 318. | Excluded | Title and abstract | Irrelevant |
| 772] Nct, NIAGEN and Persistent Chemotherapy-Induced Peripheral Neuropathy, https://clinicaltrials.gov/show/NCT04112641 (2019). | Excluded | Title and abstract | Irrelevant |
| 773] B.M. Churchill, P. Patri, The nitty-gritties of Kt/Vureacalculations in hemodialysis and peritoneal dialysis, Indian Journal of Nephrology 31(2) (2021) 97-110. | Excluded | Title and abstract | Irrelevant |
| 774] Y.A. Rajabally, J. Martey, No association between neuropathy and restless legs in Parkinson's disease, Acta neurologica Scandinavica 127(3) (2013) 216-20. | Excluded | Full text | No relevant outcome |
| 775] B. Kücükakin, M. Wilhelmsen, J. Lykkesfeldt, R.J. Reiter, J. Rosenberg, I. Gögenur, No effect of melatonin to modify surgical-stress response after major vascular surgery: a randomised placebo-controlled trial, European journal of vascular and endovascular surgery 40(4) (2010) 461‐467. | Excluded | Title and abstract | Irrelevant |
| 776] R.E. Allen, K.A. Kirby, Nocturnal leg cramps, Am Fam Physician 86(4) (2012) 350-5. | Excluded | Title and abstract | Irrelevant |
| 777] R.S. Monderer, W.P. Wu, M.J. Thorpy, Nocturnal leg cramps, Current Neurology and Neuroscience Reports 10(1) (2010) 53-59. | Excluded | Title and abstract | Irrelevant |
| 778] S. Ayres, Jr., R. Mihan, Nocturnal leg cramps (systremma): a progress report on response to vitamin E, Southern medical journal 67(11) (1974) 1308-12. | Excluded | Title and abstract | Irrelevant |
| 779] T. Araujo, F. Morneau-Sevigny, A. Valliere, NON-24-HOUR SLEEP-WAKE RHYTHM DISORDER: A SUCCESSFUL CASE OF A 50 YEARS-OLD BLIND WOMAN, Sleep 40 (2017) A463-A463. | Excluded | Title and abstract | Irrelevant |
| 780] N.M. Linardakis, N.R. Wainwright, Non-addictive composition useful for quitting smoking and treatment of sleep disorders comprises melatonin, valerian root, antioxidant component, and vitamin complex component, Linardakis N M; Wainwright N R. | Excluded | Title and abstract | Irrelevant |
| 781] U.H. Mitchell, Nondrug-related aspect of treating ekbom disease, formerly known as restless legs syndrome, Neuropsychiatric Disease and Treatment 7(1) (2011) 251-257. | Excluded | Title and abstract | Irrelevant |
| 782] R. Agarwal, Nonhematological benefits of iron, American journal of nephrology 27(6) (2007) 565-571. | Excluded | Title and abstract | Irrelevant |
| 783] C. Ammendolia, K.J. Stuber, E. Rok, R. Rampersaud, C.A. Kennedy, V. Pennick, I.A. Steenstra, L.K. de Bruin, A.D. Furlan, Nonoperative treatment for lumbar spinal stenosis with neurogenic claudication, Cochrane Database of Systematic Reviews (8) (2013). | Excluded | Title and abstract | Irrelevant |
| 784] D. Sharon, Nonpharmacologic Management of Restless Legs Syndrome (Willis-Ekbom Disease) Myths or Science, Sleep Medicine Clinics 10(3) (2015) 263-278. | Excluded | Title and abstract | Irrelevant |
| 785] R.R. Pillai Riddell, O. Bucsea, I. Shiff, C. Chow, H.G. Gennis, S. Badovinac, M. DiLorenzo-Klas, N.M. Racine, S. Ahola Kohut, D. Lisi, et al., Non‐pharmacological management of infant and young child procedural pain, Cochrane Database of Systematic Reviews (6) (2023). | Excluded | Title and abstract | Irrelevant |
| 786] L.K. Brown, M. Arora, Nonrespiratory Sleep Disorders Found in ICU Patients, Critical Care Clinics 24(3) (2008) 589-611. | Excluded | Title and abstract | Irrelevant |
| 787] A. Singh, G. Das, M. Kaur, B.N. Mallick, Noradrenaline Acting on Alpha1 Adrenoceptor as well as by Chelating Iron Reduces Oxidative Burden on the Brain: Implications With Rapid Eye Movement Sleep, Frontiers in Molecular Neuroscience 12 (2019). | Excluded | Title and abstract | Irrelevant |
| 788] K. Stiasny-Kolster, J.C. Möller, J. Zschocke, O. Bandmann, W. Cassel, W.H. Oertel, G.F. Hoffmann, Normal dopaminergic and serotonergic metabolites in cerebrospinal fluid and blood of restless legs syndrome patients, Mov Disord 19(2) (2004) 192-6. | Excluded | Title and abstract | Irrelevant |
| 789] W. Lu, C.Z. Huang, Y.F. Li, Novel assay of thiamine based on its enhancement of total internal reflected resonance light scattering signals of sodium dodecylbenzene sulfonate at the water/tetrachloromethane interface, Analytica Chimica Acta 475(1-2) (2002) 151-161. | Excluded | Title and abstract | Irrelevant |
| 790] W. Lu, C.Z. Huang, Y.F. Li, Novel assay of thiamine based on its enhancement of total internal reflected resonance light scattering signals of sodium dodecylbenzene sulfonate at the water/tetrachloromethane interface, Analytica Chimica Acta 475(1-2) (2003) 151-161. | Excluded | Title and abstract | Irrelevant |
| 791] J.W. Shin, J.S. Sunwoo, J.I. Byun, K.H. Baek, K.Y. Jung, Novel biomarkers for restless legs syndrome by proteomic analysis, Sleep medicine 64 (2019) S349-S350. | Excluded | Full text | No specific data |
| 792] E.G. Harrison, J.L. Keating, P. Morgan, Novel exercises for Restless Legs syndrome: A randomized, controlled trial, Journal of the American Board of Family Medicine 31(5) (2018) 783-794. | Excluded | Title and abstract | Irrelevant |
| 793] K.G. Claeys, A. Abicht, M. Häusler, S. Kleinle, M. Wiesmann, J.B. Schulz, R. Horvath, J. Weis, Novel genetic and neuropathological insights in neurogenic muscle weakness, ataxia, and retinitis pigmentosa (NARP), Muscle and Nerve 54(2) (2016) 328-333. | Excluded | Title and abstract | Irrelevant |
| 794] R.M. Salmerón-Campillo, M. Jaskulski, S. Lara-Cánovas, J.M. González-Méijome, N. López-Gil, Novel Method of Remotely Monitoring the Face-Device Distance and Face Illuminance Using Mobile Devices: a Pilot Study, Journal of ophthalmology (2019) 1‐9. | Excluded | Title and abstract | Irrelevant |
| 795] Nct, Nutraceutical Improvement of Glucose Metabolism, NAFLD and Insulin Resistance by Oat-fiber Supplementation in Type 2 Diabetes Mellitus Patients, https://clinicaltrials.gov/show/NCT05654805 (2022). | Excluded | Title and abstract | Irrelevant |
| 796] Actrn, Nutriceuticals in Duchenne muscular dystrophy, https://trialsearch.who.int/Trial2.aspx?TrialID=ACTRN12610000462088 (2010). | Excluded | Title and abstract | Irrelevant |
| 797] A.Z. Naqvi, R.B. Davis, K.J. Mukamal, Nutrient intake and peripheral artery disease in adults: Key considerations in cross-sectional studies, Clinical Nutrition 33(3) (2014) 443-447. | Excluded | Title and abstract | Irrelevant |
| 798] L.F. Mendez, H. Nguyen, J. Nguyen, A. Himstead, M.R. Lemm, E.S. Heide, R.M. Scherber, A. Choudhry, C.O. McKinney, R.A. Mesa, et al., The nutrient trial (nutritional Intervention among myeloproliferative neoplasms): feasibility phase, Blood 134 (2019). | Excluded | Title and abstract | Irrelevant |
| 799] N. Sinn, Nutritional and dietary influences on attention deficit hyperactivity disorder, Nutrition Reviews 66(10) (2008) 558-568. | Excluded | Title and abstract | Irrelevant |
| 800] Actrn, Nutritional biomarkers comparing a healthy versus a typical Australian diet: a Feeding Study in Australian Adults, https://trialsearch.who.int/Trial2.aspx?TrialID=ACTRN12622001321730 (2022). | Excluded | Title and abstract | Irrelevant |
| 801] A.R. Gaby, Nutritional treatments for restless legs syndrome, Integrative Medicine 6(3) (2007) 26-29. | Excluded | Title and abstract | Irrelevant |
| 802] D.B. Carr, B.R. Ott, The Older Adult Driver With Cognitive Impairment: "It's a Very Frustrating Life", Jama-Journal of the American Medical Association 303(16) (2010) 1632-1641. | Excluded | Title and abstract | Irrelevant |
| 803] Older australians can achieve high adherence to the mediterranean diet during a 6month randomised intervention; results from themedley study, Nutrients 9(6) (2017). | Excluded | Title and abstract | Irrelevant |
| 804] E.L. Bostock, C.I. Morse, K. Winwood, I.M. McEwan, G.L. Onambélé, Omega-3 Fatty Acids and Vitamin D in Immobilisation: part A- Modulation of Appendicular Mass Content, Composition and Structure, Journal of nutrition, health & aging 21(1) (2017) 51‐58. | Excluded | Title and abstract | Irrelevant |
| 805] E.L. Bostock, C.I. Morse, K. Winwood, I.M. McEwan, G.L. Onambélé, Omega-3 Fatty Acids and Vitamin D in Immobilisation: part B- Modulation of Muscle Functional, Vascular and Activation Profiles, Journal of nutrition, health & aging 21(1) (2017) 59‐66. | Excluded | Title and abstract | Irrelevant |
| 806] Actrn, Omega-3 supplements for improving peripheral nerve health in type-1 diabetes, http://www.who.int/trialsearch/Trial2.aspx?TrialID=ACTRN12618000705280 (2018). | Excluded | Title and abstract | Irrelevant |
| 807] K.R. Shieh, J.T. Pan, Ontogeny of the diurnal rhythm of tuberoinfundibular dopaminergic neuronal activity in peripubertal female rats: Possible involvement of cholinergic and opioidergic systems, Neuroendocrinology 68(6) (1998) 395-402. | Excluded | Title and abstract | Irrelevant |
| 808] M.D. Wheelock, M.A. Reid, H. To, D.M. White, K.L. Cropsey, A.C. Lahti, Open label smoking cessation with varenicline is associated with decreased glutamate levels and functional changes in anterior cingulate cortex: preliminary findings, Frontiers in pharmacology 5 (2014). | Excluded | Title and abstract | Irrelevant |
| 809] S.E. Euctr, An open-label randomized Phase II study of PA21 compared to sevelamer to evaluate the ability of PA21 to control serum phosphate levels and the tolerability in patients with chronic kidney disease undergoing hemodialysis, https://trialsearch.who.int/Trial2.aspx?TrialID=EUCTR2007-003565-40-SE (2007). | Excluded | Title and abstract | Irrelevant |
| 810] J.M. da Silva, Operacionalização dos Critérios STOPP/START e sua Aplicação em Idosos sob Terapia Anticoagulante: Experiência Profissionalizante na Vertente de Farmácia Comunitária, Hospitalar e Investigação, 2015. | Excluded | Title and abstract | Irrelevant |
| 811] N. de Jong, C.T. Hoendervangers, J.K. Bleeker, M.C. Ocké, The opinion of Dutch dietitians about functional foods, Journal of Human Nutrition and Dietetics 17(1) (2004) 55-62. | Excluded | Title and abstract | Irrelevant |
| 812] J. Li, X. Wang, Z. Feng, G. Huang, L. Yan, J. Ma, Optimization of aflatoxin B(1) removal efficiency of DNA by resonance light scattering spectroscopy, Spectrochimica acta. Part A, Molecular and biomolecular spectroscopy 292 (2023) 122398. | Excluded | Title and abstract | Irrelevant |
| 813] U. Boettger, J.P. de Vera, J. Fritz, I. Weber, H.W. Huebers, D. Schulze-Makuch, Optimizing the detection of carotene in cyanobacteria in a martian regolith analogue with a Raman spectrometer for the ExoMars mission, Planetary and Space Science 60(1) (2012) 356-362. | Excluded | Title and abstract | Irrelevant |
| 814] X. Wang, Y. Ma, X. Hui, M. Li, J. Li, J. Tian, Q. Wang, P. Yan, J. Li, P. Xie, et al., Oral direct thrombin inhibitors or oral factor Xa inhibitors versus conventional anticoagulants for the treatment of deep vein thrombosis, Cochrane Database of Systematic Reviews (4) (2023). | Excluded | Title and abstract | Irrelevant |
| 815] M. Li, J. Li, X. Wang, X. Hui, Q. Wang, S. Xie, P. Yan, J. Tian, J. Li, P. Xie, et al., Oral direct thrombin inhibitors or oral factor Xa inhibitors versus conventional anticoagulants for the treatment of pulmonary embolism, Cochrane Database of Systematic Reviews (4) (2023). | Excluded | Title and abstract | Irrelevant |
| 816] J.D. Bortz, Orally deliverable nonfood dosage system used to e.g. supply iron nutrition, comprises iron in form of e.g. iron grade and agent to mitigate gastrointestinal adverse effect of unabsorbed iron where agent comprises zinc or chelator component, Amip Llc; Albion Lab Inc; Balchem Corp. | Excluded | Title and abstract | Irrelevant |
| 817] J.Z. Ilich, J.E. Inglis, O.J. Kelly, D.L. McGee, Osteosarcopenic obesity is associated with reduced handgrip strength, walking abilities, and balance in postmenopausal women, Osteoporosis International 26(11) (2015) 2587-2595. | Excluded | Title and abstract | Irrelevant |
| 818] R. Bauersachs, N. Koitabashi, Overview of current evidence on the impact of the initial high dose of the direct factor xa inhibitor rivaroxaban on thrombus resolution in the treatment of venous thromboembolism, International heart journal 58(1) (2017) 6‐15. | Excluded | Title and abstract | Irrelevant |
| 819] Y. Ishizaki, Y. Ohta, A. Hanatani, S. Sakamoto, T. Mukobata, Y. Iwao, Y. Ota, G. Mukaihata, T. Mukouhata, Ishizakiyoichi, Otayoshihiro, Packaging material for patch preferably transdermal patch containing drug e.g. diuretics, for package structure, comprises inner layer made of polyethylene terephthalate, oxygen-absorbing layer and oxygen-barrier layer, Toyo Seikan Group Holdings Ltd; Nitto Denko Corp; Toyo Seikan Kaisha Ltd. | Excluded | Title and abstract | Irrelevant |
| 820] J.I. Sage, Pain in Parkinson's disease, Current Treatment Options in Neurology 6(3) (2004) 191-200. | Excluded | Title and abstract | Irrelevant |
| 821] F.H. Rossi, W. Liu, E. Geigel, S. Castaneda, E.M. Rossi, K. Schnacky, Painful legs and moving toes syndrome responsive to pregabalin, Journal of Postgraduate Medicine 61(2) (2015) 116-119. | Excluded | Title and abstract | Irrelevant |
| 822] Nct, Palm Tocotrienols in Chronic Hemodialysis (USA), https://clinicaltrials.gov/show/NCT02358967 (2015). | Excluded | Title and abstract | Irrelevant |
| 823] L.A. Romero Pareyón, R.F. De La Borbolla, M.D.M. Campos Fernández, L. Espinosa Ar, Parasitosis delirium, Dermatologia Revista Mexicana 52(6) (2008) 263-267. | Excluded | Title and abstract | Irrelevant |
| 824] A.Y. Avidan, Parasomnias and movement disorders of sleep, Seminars in neurology 29(4) (2009) 372-392. | Excluded | Title and abstract | Irrelevant |
| 825] R.S. Santos, F.M. Coelho, B.C. da Silva, F.G. Graciolli, W.V. Dominguez, F.L. de Menezes Montenegro, V. Jorgetti, R.M. Moysés, R.M. Elias, Parathyroidectomy Improves Restless Leg Syndrome in Patients on Hemodialysis, PloS one 11(5) (2016) e0155835. | Included | Full text | Relationship between vitamins and RLS |
| 826] M. Andréasson, N. Lagali, R.A. Badian, T.P. Utheim, F. Scarpa, A. Colonna, S. Allgeier, A. Bartschat, B. Köhler, R. Mikut, K.M. Reichert, G. Solders, K. Samuelsson, H. Zetterberg, K. Blennow, P. Svenningsson, Parkinson’s disease with restless legs syndrome—an in vivo corneal confocal microscopy study, npj Parkinson's Disease 7(1) (2021). | Included | Full text | Relationship between vitamins and RLS |
| 827] S. Chandra, K. Rao, A. Ghosh, J. Ray, H. Northrup, E. Stimming, Parkinsonism in Christianson syndrome: A unique presentation of a unique syndrome, Movement Disorders 33 (2018) S219. | Excluded | Title and abstract | Irrelevant |
| 828] S. Mudassir, A. Kumar, N. Sinha, A. Ranjan, Paroxysmal Kinesigenic Dyskinesia Secondary to Pseudohypoparathyroidism Responding to Correction of Calcium, Movement Disorders Clinical Practice 9(3) (2022) 386-389. | Excluded | Title and abstract | Irrelevant |
| 829] Z.A. Alyousif, Passive Leg Movement and NO-Mediated Vascular Function: The Impact of Obstructive Sleep Apnea (OSA), 2020. | Excluded | Title and abstract | Irrelevant |
| 830] B. Barun, Pathophysiological background and clinical characteristics of sleep disorders in multiple sclerosis, Clinical neurology and neurosurgery 115 (2013) S82-S85. | Excluded | Title and abstract | Irrelevant |
| 831] C. Altamura, I. Corbelli, M. de Tommaso, C. Di Lorenzo, G. Di Lorenzo, A. Di Renzo, M. Filippi, T.B. Jannini, R. Messina, P. Parisi, V. Parisi, F. Pierelli, I. Rainero, U. Raucci, E. Rubino, P. Sarchielli, L. Li, F. Vernieri, C. Vollono, G. Coppola, Pathophysiological Bases of Comorbidity in Migraine, Frontiers in human neuroscience 15 (2021). | Excluded | Title and abstract | Irrelevant |
| 832] F. Van Calenbergh, J. Van Hees, A patient with restless legs syndrome and periodic movements in sleep, Tijdschrift voor Geneeskunde 40(21) (1984) 1317-1320. | Excluded | Title and abstract | Irrelevant |
| 833] M. Zwakman, S.W.M. Weldam, S. Vervoort, J.J. Lammers, M.J. Schuurmans, Patients' perspectives on the COPD-GRIP intervention, a new nursing care intervention for COPD, BMC family practice 20(1) (2019) N.PAG. | Excluded | Title and abstract | Irrelevant |
| 834] B.A. Acar, M.A.G. Acar, T. Acar, C. Varım, A.N. Alagöz, E.B. Demiryürek, B. Doğan Güngen, Y. Güzey Aras, Patients with primary restless legs syndrome have higher prevalence of autonomic dysfunction and irritable bowel syndrome, Singapore Medical Journal 59(10) (2018) 539-544. | Excluded | Title and abstract | Irrelevant |
| 835] C. Martinez, H.W. Finnern, S. Rietbrock, S. Eaton, K.R. Chaudhuri, A.H.V. Schapira, Patterns of treatment for restless legs syndrome in primary care in the United Kingdom, Clinical therapeutics 30(2) (2008) 405-418. | Excluded | Title and abstract | Irrelevant |
| 836] J.S. Durmer, R.D. Chervin, Pediatric sleep medicine, CONTINUUM Lifelong Learning in Neurology 13(3 SLEEP DISORDERS) (2007) 153-200. | Excluded | Title and abstract | Irrelevant |
| 837] A.M. Reynolds, A.M. Spaeth, L. Hale, A.A. Williamson, M.K. LeBourgeois, S.D. Wong, L.E. Hartstein, J.C. Levenson, M. Kwon, C.N. Hart, A. Greer, C.E. Richardson, M. Gradisar, M.A. Clementi, S.L. Simon, L.M. Reuter-Yuill, D.L. Picchietti, S. Wild, L. Tarokh, K. Sexton-Radek, B.A. Malow, K.P. Lenker, S.L. Calhoun, D.A. Johnson, D. Lewin, M.A. Carskadon, Pediatric sleep: current knowledge, gaps, and opportunities for the future, Sleep 46(7) (2023). | Excluded | Title and abstract | Irrelevant |
| 838] M. Nakano, H. Kusaba, A. Makiyama, H. Ariyama, S. Arita, H. Oda, T. Esaki, K. Takayoshi, K. Uchino, S. Tamura, H. Kumagai, E. Iwama, T. Shirakawa, K. Mitsugi, S. Takaishi, K. Akashi, E. Baba, Pemetrexed combined with platinum-based chemotherapy for advanced malignant peritoneal mesothelioma: Retrospective analysis of six cases, Anticancer Research 34(1) (2014) 215-220. | Excluded | Title and abstract | Irrelevant |
| 839] G.M.S. Brandao, D.R. Junqueira, H.A. Rollo, M.L. Sobreira, Pentasaccharides for the treatment of deep vein thrombosis, Cochrane Database of Systematic Reviews (12) (2017). | Excluded | Title and abstract | Irrelevant |
| 840] A. Hort, H. Brand, Periconceptional multivitamin administration result in reduction of congenital abnormalities: adequate evidence for formulating national recommendations for Germany?, Gesundheitswesen (Bundesverband der Arzte des Offentlichen Gesundheitsdienstes (Germany)) 59(4) (1997) 248‐251. | Excluded | Title and abstract | Irrelevant |
| 841] M.A.E. Zagaria, Periodic limb movement disorder, restless legs syndrome, and pain, 2015, pp. 19-21. | Excluded | Title and abstract | Irrelevant |
| 842] D.L. Wilson, S.P. Walker, A.M. Fung, F.J. O’Donoghue, M. Barnes, M.E. Howard, Periodic limb movements in sleep during pregnancy: a common but benign disorder?, Sleep and Biological Rhythms 16(1) (2018) 11-20. | Excluded | Title and abstract | Irrelevant |
| 843] D.C. Williams, Periodic limb movements of sleep and the restless legs syndrome, Virginia medical quarterly : VMQ 123(4) (1996) 260-5. | Excluded | Title and abstract | Irrelevant |
| 844] S.N. Srivastava, S. Padhi, A. Domoshnitsky, Periodic solution of a bioeconomic fishery model by coincidence degree theory, Electronic Journal of Qualitative Theory of Differential Equations (29) (2023) 1-12. | Excluded | Title and abstract | Irrelevant |
| 845] T.C. Shen, B. Lebwohl, H. Verma, N. Kumta, C. Tennyson, S. Lewis, E. Scherl, A. Swaminath, K.M. Capiak, D. DiGiacomo, B.P. Bosworth, T.H. Brannagan, 3rd, P.H. Green, Peripheral neuropathic symptoms in celiac disease and inflammatory bowel disease, Journal of clinical neuromuscular disease 13(3) (2012) 137-45. | Excluded | Title and abstract | Irrelevant |
| 846] M. Andréasson, Peripheral Neuropathy and Altered Cobalamin Metabolism in Parkinson's Disease and Other Movement Disorders, 2022. | Excluded | Title and abstract | Irrelevant |
| 847] J. Yang, H. Wang, Y. Yuan, S. Fan, L. Li, C. Jiang, C. Mao, C. Shi, Y. Xu, Peripheral synucleinopathy in Parkinson disease with LRRK2 G2385R variants, Annals of Clinical and Translational Neurology 8(3) (2021) 592-602. | Excluded | Title and abstract | Irrelevant |
| 848] M. Choy, Pharmaceutical approval update, P and T 43(6) (2018) 326-327. | Excluded | Title and abstract | Irrelevant |
| 849] P.F. Dillon, R.S. Root-Bernstein, P. Dillon, R. Root-Bernstein, Pharmaceutical composition for treating a disorder, e.g. autism, in human or animal subject, comprises non-adrenergic aminergic compound (e.g. 2-(2-pyridyl)ethylamine) and complement compound (e.g. ascorbic acid), Univ Michigan State. | Excluded | Title and abstract | Irrelevant |
| 850] E.K. Flores, R. Henry, D.W. Stewart, Pharmacist's role in an interdisciplinary falls clinic, Southern medical journal 104(2) (2011) 143-146. | Excluded | Title and abstract | Irrelevant |
| 851] F.R. Euctr, Pharmacodynamic and clinical assessment of DC 982 GE (2,4 or 6 capsules per day) in patients with chronic venous disorders : randomised, placebo-controlled, dose effect, double blind, parallel group study, https://trialsearch.who.int/Trial2.aspx?TrialID=EUCTR2009-014681-25-FR (2009). | Excluded | Title and abstract | Irrelevant |
| 852] Q. Lv, X. Wang, T. Asakawa, X.P. Wang, Pharmacologic treatment of restless legs syndrome, Current Neuropharmacology 19(3) (2021) 371-381. | Excluded | Title and abstract | Irrelevant |
| 853] L.P. McLafferty, M. Spada, P. Gopalan, Pharmacologic Treatment of Sleep Disorders in Pregnancy, Sleep Medicine Clinics 17(3) (2022) 445-452. | Excluded | Title and abstract | Irrelevant |
| 854] D.G. Cacione, C.R. Macedo, F. do Carmo Novaes, J.C.C. Baptista-Silva, Pharmacological treatment for Buerger's disease, Cochrane Database of Systematic Reviews (5) (2020). | Excluded | Title and abstract | Irrelevant |
| 855] O. Bruni, M. Angriman, M.G. Melegari, R. Ferri, Pharmacotherapeutic management of sleep disorders in children with neurodevelopmental disorders, Expert opinion on pharmacotherapy 20(18) (2019) 2257-2271. | Excluded | Title and abstract | Irrelevant |
| 856] D.E. Euctr, A Phase 3 Study of an Investigational Drug, Lumasiran (ALN-GO1) with an Extended Dosing Period in Children and Adults with Primary Hyperoxaluria Type 1 Disease, https://trialsearch.who.int/Trial2.aspx?TrialID=EUCTR2018-001981-40-DE (2018). | Excluded | Title and abstract | Irrelevant |
| 857] S.I. Euctr, A Phase 3, Open-Label, Study to Evaluate Pharmacokinetics and Pharmacodynamics of Edoxaban and to compare the safety and efficacy of Edoxaban with standard of care treatment in Paediatric Patients confirmed as requiring treatment for a blood clot, https://trialsearch.who.int/Trial2.aspx?TrialID=EUCTR2016-000991-49-SI (2016). | Excluded | Title and abstract | Irrelevant |
| 858] Per, A PHASE 3, RANDOMIZED, DOUBLE-BLIND, PLACEBO CONTROLLED STUDY OF THE EFFICACY AND SAFETY OF ROXADUSTAT FOR THE TREATMENT OF ANEMIA IN CHRONIC KIDNEY DISEASE PATIENTS NOT ON DIALYSIS, https://trialsearch.who.int/Trial2.aspx?TrialID=PER-058-15 (2016). | Excluded | Title and abstract | Irrelevant |
| 859] Kct, A Phase 3, Randomized, Observer-blind, Multicenter Study to Evaluate the Immunogenicity and Safety of Novartis rMenB+OMV NZ Vaccine in Healthy Subjects Aged 11 to 17 years in Korea, https://trialsearch.who.int/Trial2.aspx?TrialID=KCT0001103 (2014). | Excluded | Title and abstract | Irrelevant |
| 860] I.T. Euctr, Phase II multicenter clinical trial, with treatment randomly assigned, in which patient nor physician are aware of the assigned treatment, active or placebo, to evaluate efficacy and safety of Velusetrag 15 mg (3 x 5 mg capsule) in patients with Chronic Intestinal Pseudo-Obstruction (CIPO), https://trialsearch.who.int/Trial2.aspx?TrialID=EUCTR2021-000854-24-IT (2021). | Excluded | Title and abstract | Irrelevant |
| 861] A.T. Euctr, A Phase II, randomised, multi-centric, multi-national clinical trial to evaluate the efficacy, tolerability, and safety of a fixed dose combination of Spironolactone, Pioglitazone & Metformin (SPIOMET) for adolescent girls and young adult women (AYAs) with polycystic ovary syndrome (PCOS), https://trialsearch.who.int/Trial2.aspx?TrialID=EUCTR2021-003177-58-AT (2021). | Excluded | Title and abstract | Irrelevant |
| 862] N.L. Euctr, A Phase IIb, Placebo-Controlled, Study to Optimize Diamyd® Therapy Administered into Lymph Nodes Combined with Vitamin D oral drops to Investigate the Impact on the Progression of Type 1 diabetes, https://trialsearch.who.int/Trial2.aspx?TrialID=EUCTR2017-001861-25-NL (2018). | Excluded | Title and abstract | Irrelevant |
| 863] S.E. Euctr, A Phase IIb, Placebo-Controlled, Study to Optimize Diamyd® Therapy Administered into Lymph Nodes Combined with Vitamin D oral drops to Investigate the Impact on the Progression of Type 1 diabetes, https://trialsearch.who.int/Trial2.aspx?TrialID=EUCTR2017-001861-25-SE (2017). | Excluded | Title and abstract | Irrelevant |
| 864] H. Birgens, H. Frederiksen, H.C. Hasselbalch, I.H. Rasmussen, O.J. Nielsen, L. Kjeldsen, H. Larsen, T. Mourits-Andersen, T. Plesner, D. Rønnov-Jessen, H. Vestergaard, T.W. Klausen, C. Schöllkopf, A phase III randomized trial comparing glucocorticoid monotherapy versus glucocorticoid and rituximab in patients with autoimmune haemolytic anaemia, British journal of haematology 163(3) (2013) 393-399. | Excluded | Title and abstract | Irrelevant |
| 865] Nct, Phase III Trial of Coenzyme Q10 in Mitochondrial Disease, https://clinicaltrials.gov/show/NCT00432744 (2007). | Excluded | Title and abstract | Irrelevant |
| 866] L.B. Tulloch, M.J. Shattock, W. Fuller, Phospholemman Palmitoylation: a Novel Means of Sodium Pump Regulation, Circulation 122(21) (2010). | Excluded | Title and abstract | Irrelevant |
| 867] D. Yin, W. Gao, S. Cui, Y. Gu, Photodynamic therapy based on upconversion nanoconstructs, Photonics and Lasers in Medicine 4(4) (2015) 322-325. | Excluded | Title and abstract | Irrelevant |
| 868] V.O. Mittova, A.U. Igamberdiev, Photoperiodical changes of the activity of enzymes of ascorbate-glutathione cycle and ascorbate oxidase in pea leaves, Plant Physiology (Rockville) 114(3 SUPPL.) (1997) 57-57. | Excluded | Title and abstract | Irrelevant |
| 869] K.-N. Koh, J.Y. Jeon, S.-S. Park, H.J. Im, H. Kim, M.S. Kang, Physeal Abnormalities in Children With High-risk Neuroblastoma Intensively Treated With/Without 13-Cis-Retinoic Acid, Journal of Pediatric Orthopaedics 41(9) (2021) E841-E848. | Excluded | Title and abstract | Irrelevant |
| 870] M. Pang, S. Kang, L. Liu, T. Ma, Z. Zheng, L. Cao, Physicochemical Properties and Cookie-Making Performance as Fat Replacer of Wax-Based Rice Bran Oil Oleogels, Gels 9(1) (2023). | Excluded | Title and abstract | Irrelevant |
| 871] Phytotherapy: Rarely mentioned spontaneously, Prescrire international 15(82) (2006) 63. | Excluded | Title and abstract | Irrelevant |
| 872] Actrn, Pictorial calendar for adherence to complementary feeding (PiC-Feed), https://trialsearch.who.int/Trial2.aspx?TrialID=ACTRN12620000774921 (2020). | Excluded | Title and abstract | Irrelevant |
| 873] Nct, Pilot of a Prebiotic and Probiotic Trial in Young Infants With Severe Acute Malnutrition, https://clinicaltrials.gov/show/NCT03666572 (2018). | Excluded | Title and abstract | Irrelevant |
| 874] A.F. Garza, R. Monroy-Maya, M. Soto-Ríos, G. Reyes-García, L. Carrillo-Alarcón, H. Ponce-Monter, E. Rangel-Flores, M.I. Ortiz, A pilot study of the effect of diclofenac with B vitamins for the treatment of acute pain following lower-limb fracture and surgery, Proceedings of the Western Pharmacology Society 51 (2008) 70‐72. | Excluded | Title and abstract | Irrelevant |
| 875] F.R. Euctr, A Pilot study to evaluate the Efficacy and Safety of GFT505 (80 mg) orally administered once daily for 28 days in atherogenic dyslipidaemic patients with abdominal obesity. A double blind, placebo-controlled and randomized study, https://trialsearch.who.int/Trial2.aspx?TrialID=EUCTR2008-005779-86-FR (2008). | Excluded | Title and abstract | Irrelevant |
| 876] Nct, Piloting Diet and Exercise Interventions in Older Hispanics With Diabetes, https://clinicaltrials.gov/show/NCT04132739 (2019). | Excluded | Title and abstract | Irrelevant |
| 877] E.H. Lee, Plant resistance mechanisms to air pollutants: rhythms in ascorbic acid production during growth under ozone stress, Chronobiology international 8(2) (1991) 93-102. | Excluded | Title and abstract | Irrelevant |
| 878] T.P. Griffin, M.N. Islam, D. Wall, J. Ferguson, D.G. Griffin, M.D. Griffin, P.M. O'Shea, Plasma dephosphorylated- uncarboxylated Matrix Gla-Protein (dp-ucMGP): reference intervals in Caucasian adults and diabetic kidney disease biomarker potential, Scientific reports 9 (2019). | Excluded | Title and abstract | Irrelevant |
| 879] K. Szadejko, K. Dziewiatowski, K. Szabat, P. Robowski, M. Schinwelski, E. Sitek, J. Slawek, Polyneuropathy in levodopa-treated Parkinson's patients, Journal of the neurological sciences 371 (2016) 36-41. | Excluded | Title and abstract | Irrelevant |
| 880] W.D. Chapman, M.C. Herink, M.H. Cameron, D. Bourdette, Polypharmacy in Multiple Sclerosis: Prevalence, Risks, and Mitigation Strategies, Current Neurology and Neuroscience Reports 23(9) (2023) 521-529. | Excluded | Title and abstract | Irrelevant |
| 881] C. Geng, Z. Yang, T. Zhang, P. Xu, H. Zhang, Polysomnographic nighttime features of Restless Legs Syndrome: A systematic review and meta-analysis, Frontiers in Neurology 13 (2022). | Excluded | Title and abstract | Irrelevant |
| 882] A.V. Gavrilenko, D.A. Voronov, Possibilities and results of using clopidogrel (Listab) in comprehensive treatment of patients with crural deep veins thrombosis, Angiologiia i sosudistaia khirurgiia = Angiology and vascular surgery 21(1) (2015) 91‐4; 96‐8. | Excluded | Title and abstract | Irrelevant |
| 883] I. Ignatov, C. Belden, S. Jacobson, D. Connor, M.N. Sabbagh, Possible Alzheimer's disease in an apolipoprotein E2 homozygote, Journal of Alzheimer's Disease 16(1) (2009) 35-38. | Excluded | Title and abstract | Irrelevant |
| 884] C. Geng, Z. Yang, P. Xu, H. Zhang, Possible association between vitamin B12 deficiency and restless legs syndrome, Clinical neurology and neurosurgery 223 (2022) 107477. | Included | Full text | Relationship between vitamins and RLS |
| 885] M. Oran, C. Unsal, Y. Albayrak, F. Tulubas, K. Oguz, O. Avci, N. Turgut, R. Alp, A. Gurel, Possible association between vitamin D deficiency and restless legs syndrome, Neuropsychiatr Dis Treat 10 (2014) 953-8. | Included | Full text | Relationship between vitamins and RLS |
| 886] M.I. Botez, B. Lambert, A possible correlation between restless legs syndrome and folate deficiency in pregnancy, Nutrition Reports International 18(2) (1978) 143-146. | Excluded | Full text | Duplication |
| 887] A. Mendes, V. Silva, Possible etiologies of restless legs syndrome in pregnancy: a narrative review, Sleep science (Sao Paulo, Brazil) 15(4) (2022) 471-479. | Excluded | Title and abstract | Irrelevant |
| 888] Z. Yavuz, S. Tezcan, M.K. Kumcu, B. Ölmez, C. Yücesan, Post-COVID-19 prolonged neurological symptoms and characteristics: A face-to-face survey study, Neurology Asia 27(4) (2022) 899-908. | Excluded | Title and abstract | Irrelevant |
| 889] Nct, Posterior Lumbar Stress Reaction in Pediatric Patients. Treatment With or Without Soft Spinal Brace?, https://clinicaltrials.gov/show/NCT04533178 (2020). | Excluded | Title and abstract | Irrelevant |
| 890] P. Tater, S. Pandey, Post-stroke Movement Disorders: Clinical Spectrum, Pathogenesis, and Management, Neurology India 69(2) (2021) 272-283. | Excluded | Title and abstract | Irrelevant |
| 891] M. Waly, R.C. Deth, Potent effects of ethanol on basal and D4 dopamine receptor-mediated phospholipid methylation, Society for Neuroscience Abstracts 26(1-2) (2000) Abstract No.-533.8. | Excluded | Title and abstract | Irrelevant |
| 892] I.B. Hutchinson, J. Parnell, H.G.M. Edwards, J. Jehlicka, C.P. Marshall, L.V. Harris, R. Ingley, Potential for analysis of carbonaceous matter on Mars using Raman spectroscopy, Planetary and Space Science 103 (2014) 184-190. | Excluded | Title and abstract | Irrelevant |
| 893] S.G. Zupcic, M. Zupcic, V. Duzel, T. Simurina, L. Sakic, I. Grubjesic, I.S. Udovic, V.M. Ferreri, D. Tonkovic, The potential role of micro-RNA-211 in the pathogenesis of sleep-related hypermotor epilepsy, Medical hypotheses 143 (2020). Excluded Title and abstract Irrelevant | Excluded | Title and abstract | Irrelevant |
| 894] T.H. Bothwell, A.P. MacPhail, The potential role of NaFeEDTA as an iron fortificant, International Journal for Vitamin and Nutrition Research 74(6) (2004) 421-434. | Excluded | Title and abstract | Irrelevant |
| 895] J.W. Winkelman, M.J. Armstrong, R.P. Allen, K.R. Chaudhuri, W. Ondo, C. Trenkwalder, P.C. Zee, G.S. Gronseth, D. Gloss, T. Zesiewicz, Practice guideline summary: Treatment of restless legs syndrome in adults, Neurology 87(24) (2016) 2585-2593. | Excluded | Title and abstract | Irrelevant |
| 896] J.W. Winkelman, M.J. Armstrong, R.P. Allen, K.R. Chaudhuri, W. Ondo, C. Trenkwalder, P.C. Zee, G.S. Gronseth, D. Gloss, T. Zesiewicz, Practice guideline summary: Treatment of restless legs syndrome in adults: Report of the Guideline Development, Dissemination, and Implementation Subcommittee of the American Academy of Neurology, Neurology 87(24) (2016) 2585-2593. | Excluded | Title and abstract | Irrelevant |
| 897] C. Dondé, L. Peter-Derex, F. Pitance, E. Cotte, P.M. Gonnaud, M. Saoud, Pramipexole-responsive acute restless arms syndrome after surgery under general anesthesia: Case report and literature review, Revue neurologique 173(4) (2017) 234-236. | Excluded | Title and abstract | Irrelevant |
| 898] L. Zhu, G. Duan, C. Yan, J. Wang, Prediction of microbe-drug associations based on chemical structures and the katz measure, Current Bioinformatics 16(6) (2021) 807-819. | Excluded | Title and abstract | Irrelevant |
| 899] A. Avihingsanon, P. Tangkijvanich, T. Apornpong, S. Jirajariyavej, K. Ruxrungtham, Predictors of advanced liver fibrosis among HIV/HCV coinfection and HCV mono-infection in Thailand: The role of HIV and hypovitaminosis D, Hepatology International 7 (2013) S750. | Excluded | Title and abstract | Irrelevant |
| 900] G.M.P. LaPosta, S.K. Creech, A. Macdonald, C.T. Taft, Predictors of Session Attendance in Intimate Partner Violence Treatment for Trauma-Exposed Veterans, Partner abuse 10(3) (2019) 283‐297. | Excluded | Title and abstract | Irrelevant |
| 901] T. Tunç, Y.S. Karadağ, F. Doğulu, L.E. Inan, Predisposing factors of restless legs syndrome in pregnancy, Mov Disord 22(5) (2007) 627-31. | Included | Full text | Relationship between vitamins and RLS |
| 902] Y. Saito, Y. Takekuma, M. Furuta, M. Sugawara, Pregabalin Attenuates Carboplatin-Induced Akathisia-Like Neuropathy: A Novel Case Report, Case Reports in Oncology 14(3) (2021) 1418-1421. | Excluded | Title and abstract | Irrelevant |
| 903] M. Manconi, V. Govoni, A. De Vito, N.T. Economou, E. Cesnik, G. Mollica, E. Granieri, Pregnancy as a risk factor for restless legs syndrome, Sleep medicine 5(3) (2004) 305-8. | Excluded | Title and abstract | Irrelevant |
| 904] D.B. Kirsch, A.Y. Avidan, Pregnancy associated with daytime sleepiness and nighttime restlessness, Sleep medicine 6(5) (2005) 473-474. | Excluded | Title and abstract | Irrelevant |
| 905] I. Arico, L. Campolo, R. Silvestri, Preliminary data on vitamin D deficiency and treatment in a cohort of Sicilian RLS/WED patients, Journal of Sleep Research 25 (2016) 217-217. | Excluded | Full text | Without control |
| 906] S. Ioannou, A.L. Williams, Preliminary fMRI findings concerning the influence of 5-HTP on food selection, Brain and behavior 7(1) (no pagination) (2017). | Excluded | Title and abstract | Irrelevant |
| 907] S.K. Severino, M.L. Moline, Premenstrual syndrome. Identification and management, Drugs 49(1) (1995) 71-82. | Excluded | Title and abstract | Irrelevant |
| 908] P. Ziegler, P. Geisser, Preparation, useful e.g. to treat iron deficiency conditions such as chronic inflammatory bowel disease, comprises iron (III)-complex compound and redox-active substance e.g. ascorbic acid, vitamin E, cystein, rutin and flavone, Vifor Int Ag. | Excluded | Title and abstract | Irrelevant |
| 909] D. Collister, J.C. Rodrigues, L. Mbuagbaw, P.J. Devereaux, G. Guyatt, W. Herrington, M. Walsh, Prerandomization run-in periods in randomized controlled trials of chronic diseases: a methodological study, Journal of Clinical Epidemiology 128 (2020) 148-156. | Excluded | Title and abstract | Irrelevant |
| 910] K. Li, M. Brown, Prescribing in renal supportive care, Australian Prescriber 43(2) (2020) 57-60. | Excluded | Title and abstract | Irrelevant |
| 911] S.M. Mubeen, M.D. Ahsan, Prevalence and associated factors of restless leg syndrome (RLS) in Pakistani women during pregnancy, Journal of Obstetrics and Gynaecology 42(6) (2022) 1829-1834. | Excluded | Title and abstract | Irrelevant |
| 912] M. Vahdat, E. Sariri, S. Miri, M. Rohani, M. Kashanian, A. Sabet, B. Zamani, Prevalence and associated features of restless legs syndrome in a population of Iranian women during pregnancy, International journal of gynaecology and obstetrics: the official organ of the International Federation of Gynaecology and Obstetrics 123(1) (2013) 46-9. | Excluded | Full text | Data couldn't be separately extracted |
| 913] H. Eto, K. Nishihara, H. Kondo, Prevalence and clinical features of restless legs syndrome among Japanese pregnant women with complications, Journal of Sleep Research 25 (2016) 311-311. | Excluded | Full text | No relevant outcome |
| 914] A. Hatanaka, H. Eto, C. Kato, Y. Yamaguchi, H. Sakamoto, H. Kondo, Prevalence and clinical features of restless legs syndrome among Japanese pregnant women without gestational complications, Sleep and Biological Rhythms 15(2) (2017) 183-186. | Excluded | Full text | No specific data |
| 915] M. Mosli, L. Bukhari, A. Khoja, N. Ashour, H. Aljahdali, O. Khoja, Y. Qari, Prevalence and clinical predictors of restless leg syndrome in inflammatory bowel disease patients, Saudi Journal of Gastroenterology 26(SUPPL 2) (2020) S43-S44. | Excluded | Title and abstract | Irrelevant |
| 916] K. Albazli, A.S. Walters, E.G. Karroum, PREVALENCE AND IMPACT OF MUSCLE CRAMPS IN PATIENTS WITH RESTLESS LEGS, Sleep 45(SUPPL 1) (2022) A242. | Excluded | Title and abstract | Irrelevant |
| 917] C. Yoshimura, H. Amagase, M. Takewaka, K. Nakashima, H. Imaoka, T. Shigematsu, Y. Amagase, S. Handa, T. Ohkusa, M. Nishizaka, S. Ando, Prevalence and its temporal changes of restless legs syndrome during the pregnancy in current Japan, Sleep 38 (2015) A249. | Excluded | Title and abstract | Irrelevant |
| 918] B.L. Buda, P.F. Szatmary, G.A. Toth, Prevalence and management of restless legs syndrome during pregnancy in western Hungarian women, Journal of Sleep Research 15 (2006) 89-89. | Excluded | Title and abstract | Irrelevant |
| 919] A.S. Almeneessier, M. Alzahrani, A. Alsafi, R. Alotaibi, A.H. Olaish, A.S. BaHammam, Prevalence and predictors of restless legs syndrome in non-pregnant Saudi women of childbearing age, Sleep & breathing = Schlaf & Atmung 24(3) (2020) 1107-1113. | Included | Full text | Relationship between vitamins and RLS |
| 920] A. Abulhamail, A. AlShebli, L. Merdad, S. Wali, W. Jastaniah, B. Abaalkhail, Prevalence of and risk factors for obstructive sleep apnea in children with sickle cell: a multicentric cross sectional study, Annals of hematology 101(1) (2022) 43-57. | Excluded | Title and abstract | Irrelevant |
| 921] O. Alharbi, N. Azzam, M. Almadi, F. Angkaya, N. Sadaf, A. Aljebreen, A.B. Hammam, S. Al Saleh, Prevalence of restless leg syndrome among patients suffering from inflammatory bowel disease in Saudi Arabia, Inflammatory Bowel Diseases 22 (2016) S28. | Excluded | Title and abstract | Irrelevant |
| 922] A. Dimova, B. Nedanovska, V. Cicic, The prevalence of restless leg syndrome in Macedonian primary care population, Journal of the neurological sciences 238 (2005) S320-S320. | Excluded | Title and abstract | Irrelevant |
| 923] S.R. Bista, L. Smith, P.J. Murphy, J. Dickinson, Prevalence of restless legs syndrome (RLS) in cystic fibrosis (CF) patients, Pediatric Pulmonology 51 (2016) 361. | Excluded | Title and abstract | Irrelevant |
| 924] S. Alnaaim, F. Alghirash, A. Alenzi, M. Owaidh Abu Zahirah, T. Tashari, F. Hakami, R. Alsharif, The Prevalence of Restless Legs Syndrome Among Pregnant Women in Saudi Arabia, Cureus 15(8) (2023) e42883. | Included | Full text | Relationship between vitamins and RLS |
| 925] A.S. Almeneessie, N. Alyousefi, M. Alzahrani, A. Alsafi, R. Alotaibi, A.H. Olaish, Y. Sabr, A.S. Bahammam, Prevalence of restless legs syndrome among pregnant women: A case-control study, Annals of thoracic medicine 15(1) (2020) 9-14. | Included | Full text | Relationship between vitamins and RLS |
| 926] A. Bener, A. Al-Hamaq, A.F. Ağan, M. Öztürk, A. Ömer, The prevalence of restless legs syndrome and comorbid condition among patient with type 2 diabetic mellitus visiting primary healthcare, Journal of family medicine and primary care 8(12) (2019) 3814-3820. | Included | Full text | Relationship between vitamins and RLS |
| 927] V. Altunayoglu Cakmak, B. Koc, I. Nuhoglu, M. Topbas, S.Y. Ucuncu, O. Deger, S. Kamburoglu, S. Velioglu, Prevalence of restless legs syndrome in Trabzon in the northeast Black Sea Region of Turkey: co-morbidities, socioeconomic factors and biochemical parameters, Neurological research 37(9) (2015) 751-62. | Included | Full text | Relationship between vitamins and RLS |
| 928] V.A. Cakmak, B. Koc, I. Nuhoglu, M. Topbas, S. Gazioglu, Prevalence of restless legs syndrome in Trabzon, in the northeast Black Sea region of Turkey: associated factors, clinical characteristics and biochemical correlations, European journal of neurology 21 (2014) 531-531. | Excluded | Full text | Duplication |
| 929] X. Alvarado, Prevalence of restless legs syndrome secondary to hemodialysis unit clinicas hospital, Sleep medicine 14 (2013) e59. | Excluded | Full text | No specific data |
| 930] S. Harano, T. Hida, Y. Kaneita, E. Yokoyama, T. Tamaki, S. Takemura, Y. Osaki, K. Hayashi, Prevalence of restless legs syndrome with pregnancy and the relationship with sleep disorders in the Japanese large population, Sleep and Biological Rhythms 6(2) (2008) 102-109. | Excluded | Title and abstract | Irrelevant |
| 931] A.S. Almeneessier, M. Alangari, A. Aldubayan, A. Alsharidah, A. Altaki, A.H. Olaish, Y.S. Sabr, A.S. BaHammam, Prevalence of symptoms and risk of obstructive sleep apnea in Saudi pregnant women, Annals of thoracic medicine 15(3) (2020) 163-170. | Excluded | Title and abstract | Irrelevant |
| 932] K.W. Kim, I.Y. Yoon, S. Chung, Y.K. Shin, S.B. Lee, E.A. Choi, J.H. Park, J.M. Kim, Prevalence, comorbidities and risk factors of restless legs syndrome in the Korean elderly population - results from the Korean Longitudinal Study on Health and Aging, J Sleep Res 19(1 Pt 1) (2010) 87-92. | Included | Full text | Relationship between vitamins and RLS |
| 933] J.W. Mold, S.K. Vesely, B.A. Keyl, J.B. Schenk, M. Roberts, The prevalence, predictors, and consequences of peripheral sensory neuropathy in older patients, The Journal of the American Board of Family Practice 17(5) (2004) 309-18. | Excluded | Title and abstract | Irrelevant |
| 934] H. Turan, S. Aşkın Turan, Z. Butun, M. Kayapınar, The Prevalence, Severity, and Predictive Factors of Restless Legs Syndrome in Pregnancy, Cureus 15(9) (2023) e44884. | Included | Full text | Relationship between vitamins and RLS |
| 935] M. Siemiński, M. Karwacka, M. Potocka, A. Nitka-Siemińska, W.M. Nyka, Prevelance of restless legs syndrome in the population of pregnant women, Sen 6(2) (2006) 71-74. | Excluded | Full text | No relevant outcome |
| 936] Preventative therapy plays an important role in managing chronic daily headache in paediatric patients, Drugs and Therapy Perspectives 25(1) (2009) 10-13. | Excluded | Title and abstract | Irrelevant |
| 937] Ctri, Preventing poor height gain in young children by improving the present State Governments program for child nutrition, http://www.who.int/trialsearch/Trial2.aspx?TrialID=CTRI/2020/03/024183 (2020). | Excluded | Title and abstract | Irrelevant |
| 938] Ctri, Preventing poor height gain in young children by milk or pulses based food mix, http://www.who.int/trialsearch/Trial2.aspx?TrialID=CTRI/2020/06/025825 (2020). | Excluded | Title and abstract | Irrelevant |
| 939] Nct, Preventing Tobacco Relapse With Omega-3s Trial, https://clinicaltrials.gov/show/NCT03113370 (2017). | Excluded | Title and abstract | Irrelevant |
| 940] Nct, Prevention of Oral Mucositis After Using Oral Topical Vitamin E Versus Voriconazole and Levofloxacin in Pediatric Cancer Patients Receiving Chemotherapy, https://clinicaltrials.gov/show/NCT03613389 (2018). | Excluded | Title and abstract | Irrelevant |
| 941] P. Giglio, N. Undevia, J.P. Spire, The primary parasomnias: A review for neurologists, The neurologist 11(2) (2005) 90-97. | Excluded | Title and abstract | Irrelevant |
| 942] C. Mesagno, J. Beckmann, V.V. Wergin, P. Gröpel, Primed to perform: comparing different pre-performance routine interventions to improve accuracy in closed, self-paced motor tasks, Psychology of sport and exercise 43 (2019) 73‐81. | Excluded | Title and abstract | Irrelevant |
| 943] J.H. Humphrey, K.P. West, Jr., Muhilal, L.C. See, G. Natadisastra, A. Sommer, A priming dose of oral vitamin A given to preschool children may extend protection conferred by a subsequent large dose of vitamin A, The Journal of nutrition 123(8) (1993) 1363-9. | Excluded | Title and abstract | Irrelevant |
| 944] M. Memarpoor-Yazdi, H. Mahaki, Probing the interaction of human serum albumin with vitamin B2 (riboflavin) and L-Arginine (L-Arg) using multi-spectroscopic, molecular modeling and zeta potential techniques, Journal of Luminescence 136 (2013) 150-159. | Excluded | Title and abstract | Irrelevant |
| 945] Nct, Probiotics in the Treatment of Iron Deficiency in Children With Restless Leg Syndrome, https://clinicaltrials.gov/show/NCT01617044 (2012). | Excluded | Title and abstract | Irrelevant |
| 946] N.E. Khan, J.A. Myers, A.L. Tuerk, W.R. Curtis, A process economic assessment of hydrocarbon biofuels production using chemoautotrophic organisms, Bioresource Technology 172 (2014) 201-211. | Excluded | Title and abstract | Irrelevant |
| 947] K. Pydynkowska, A. Faruga, J. Jankowski, PRODUCTION EFFICIENCY OF SLAUGHTER TURKEY-TOMS FED DIETS SUPPLEMENTED WITH CHOLECALCIFEROL AND 25-HYDROXYCHOLECALCIFEROL, Polish Journal of Natural Sciences 23(4) (2008) 779-789. | Excluded | Title and abstract | Irrelevant |
| 948] Nct, Production of Fortified Biscuit With Chickpea and Crushed Peanut for Improving Cognitive Performance, https://clinicaltrials.gov/show/NCT05281146 (2022). | Excluded | Title and abstract | Irrelevant |
| 949] F. Loscocco, G. Visani, E. Giacomini, A. Ruzzo, S. Galimberti, M.T. Voso, C. Finelli, E. Ciabatti, E. Fabiani, F. Graziano, S. Barulli, A. Volpe, D. Magro, P.P. Piccaluga, F. Fuligni, M. Vignetti, P. Fazi, A. Piciocchi, C. Clissa, E. Gabucci, M. Rocchi, M. Magnani, A. Isidori, Prognostic impact of TS, MTHFR and XRCC1 genetic variants in 113 patients with myelodysplastic syndromes, Blood 126(23) (2015) 1675. | Excluded | Title and abstract | Irrelevant |
| 950] M. Crespo-Yanguas, J. Lumpuy-Castillo, C. Espadas, C. Aragón-Valera, C. Vázquez, Ó. Lorenzo, A Program of Life-Style Modification Improved the Body Weight and Micronutrient Status in Obese Patients after Bariatric Surgery, Nutrients 15(17) (2023). | Excluded | Title and abstract | Irrelevant |
| 951] B. Ortel, E.V. Maytin, Progress in dermatology, Photonics and Lasers in Medicine 2(4) (2013) 265-267. | Excluded | Title and abstract | Irrelevant |
| 952] W. Wu, R. Xue, Progress in the research of restless legs syndrome, Chinese Journal of Contemporary Neurology and Neurosurgery 13(5) (2013) 387-391. | Excluded | Title and abstract | Irrelevant |
| 953] F. Couturaud, G. Pernod, C. Pison, P. Mismetti, O. Sanchez, G. Meyer, F. Parent, P. Girard, G. Simonneau, L. Drouet, et al., Prolongation of anti vitamin K treatment for 18 months versus placebo after 6 months treatment of a first episode of ideopathic pulmonary embolism: a mutlicentre, randomised double blind trail. The PADIS-EP Trial, Revue des maladies respiratoires 25(7) (2008) 885‐893. | Excluded | Title and abstract | Irrelevant |
| 954] M. Bielawiec, PROLONGATION OF INCREASED FIBRINOLYSIS WITH THE AID OF ORGANIC ACTIVATORS, Polski tygodnik lekarski (Warsaw, Poland : 1960) 19 (1964) 581-5. | Excluded | Title and abstract | Irrelevant |
| 955] Actrn, Prophylactic Use of Erythropoietin in patients receiving chemotherapy for cancer, https://trialsearch.who.int/Trial2.aspx?TrialID=ACTRN12614001082695 (2014). | Excluded | Title and abstract | Irrelevant |
| 956] T. Osaki, E. Ueta, K. Yoneda, J. Hirota, T. Yamamoto, Prophylaxis of oral mucositis associated with chemoradiotherapy for oral carcinoma by Azelastine hydrochloride (Azelastine) with other antioxidants, Head & neck 16(4) (1994) 331-9. | Excluded | Title and abstract | Irrelevant |
| 957] A. Bitenc-Jasiejko, K. Konior, K. Gonta, M. Duleba, D. Lietz-Kijak, Prophylaxis of Pain and Fractures within Feet in the Course of Osteoporosis: The Issue of Diagnosing, Pain research & management 2020 (2020). | Excluded | Title and abstract | Irrelevant |
| 958] D. Scott, L. Blizzard, J. Fell, C. Ding, T. Winzenberg, G. Jones, A prospective study of the associations between 25-hydroxyvitamin D, sarcopenia progression and physical activity in older adults, Clinical endocrinology 73(5) (2010) 581-587. | Excluded | Title and abstract | Irrelevant |
| 959] I.T. Euctr, A PROSPECTIVE, PILOT, CROSS-OVER STUDY TO ASSESS THE EFFICACY OF PARICALCITOL IN REDUCING PARATHYROID HORMONE LEVELS AND AMELIORATING MARKERS OF BONE REMODELLING IN RENAL TRANSPLANT RECIPIENTS WITH SECONDARY HYPERPARATHYROIDISM (APPLE STUDY) - ND, https://trialsearch.who.int/Trial2.aspx?TrialID=EUCTR2008-006380-36-IT (2008). | Excluded | Title and abstract | Irrelevant |
| 960] B.Z. Siegel, S.M. Siegel, T. Correa, C. Dagan, G. Galvez, L. LeeLoy, A. Padua, E. Yaeger, The protection of invertebrates, fish, and vascular plants against inorganic mercury poisoning by sulfur and selenium derivatives, Archives of environmental contamination and toxicology 20(2) (1991) 241-6. | Excluded | Title and abstract | Irrelevant |
| 961] Nct, Protein and Recovery From Exercise-induced Muscle Damage, https://clinicaltrials.gov/show/NCT02034721 (2014). | Excluded | Title and abstract | Irrelevant |
| 962] A.M. Holwerda, M. Overkamp, K.J.M. Paulussen, J.S.J. Smeets, J. van Kranenburg, E.M.P. Backx, A.P. Gijsen, J.P.B. Goessens, L.B. Verdijk, L.J.C. van Loon, Protein Supplementation after Exercise and before Sleep Does Not Further Augment Muscle Mass and Strength Gains during Resistance Exercise Training in Active Older Men, Journal of Nutrition 148(11) (2018) 1723-1732. | Excluded | Title and abstract | Irrelevant |
| 963] J. Gade, A.M. Beck, C. Bitz, B. Christensen, T.W. Klausen, A. Vinther, A. Astrup, Protein-enriched, milk-based supplement to counteract sarcopenia in acutely ill geriatric patients offered resistance exercise training during and after hospitalisation: study protocol for a randomised, double-blind, multicentre trial, BMJ open 8(2) (2018) e019210. | Excluded | Title and abstract | Irrelevant |
| 964] S.M. Patton, Y.W. Cho, T.W. Clardy, R.P. Allen, C.J. Earley, J.R. Connor, Proteomic analysis of the cerebrospinal fluid of patients with restless legs syndrome/Willis-Ekbom disease, Fluids and barriers of the CNS 10(1) (2013) 20. | Excluded | Title and abstract | Irrelevant |
| 965] K. Cederberg, U. Hanif, E. Leary, L. Schneider, A.M. Morse, A. Blackman, P. Schweitzer, S. Kotagal, R. Bogan, C. Kushida, E. Mignot, PROTEOMIC APPROACH FOR UNDERSTANDING THE MECHANISMS OF PERIODIC LIMB MOVEMENTS AND RESTLESS LEGS SYNDROME, Sleep 45 (2022) A244-A244. | Excluded | Title and abstract | Irrelevant |
| 966] T.P. Rakesh, Proton pump inhibitors: Use, misuse and concerns about long-term therapy, Clinical Journal of Gastroenterology 4(2) (2011) 53-59. | Excluded | Title and abstract | Irrelevant |
| 967] W. Cui, D.H. McGregor, S.P. Stark, O. Ulusarac, S.C. Mathur, Pseudoepitheliomatous hyperplasia-an unusual reaction following tattoo: report of a case and review of the literature, International Journal of Dermatology 46(7) (2007) 743-745. | Excluded | Title and abstract | Irrelevant |
| 968] H. Arshad, A. Arshad, M.Y. Hafiz, G. Muhammad, S. Khatri, F. Arain, Psychiatric Manifestations of Iron Deficiency Anemia- A Literature Review, European Psychiatry 66 (2023) S243-S244. | Excluded | Title and abstract | Irrelevant |
| 969] A.O. Adelufosi, O. Abayomi, T.M.F. Ojo, Pyridoxal 5 phosphate for neuroleptic‐induced tardive dyskinesia, Cochrane Database of Systematic Reviews (4) (2015). | Excluded | Title and abstract | Irrelevant |
| 970] N.T. Abokrysha, Quality of sleep, memory dysfunction and vitamin D deficiency in patients with restless legs syndrome in Saudi Arabia, Egyptian Journal of Neurology, Psychiatry and Neurosurgery 50(1) (2013) 81-88. | Excluded | Full text | Without control |
| 971] H. Zhang, Y. Wang, Z. Liu, E.W. Chui, A Quasi-Trial Investigation of an In-Service Training to Improve Social Workers' Professional Competence in China, Research on social work practice 29(5) (2019) 506‐518. | Excluded | Title and abstract | Irrelevant |
| 972] J.E. Letzen, M.L. Robinson, J.M. Saletin, R.B. Sheinberg, M.T. Smith, C.M. Campbell, Racial disparities in sleep-related cardiac function in young, healthy adults: Implications for cardiovascular-related health, Sleep 44(11) (2021). | Excluded | Title and abstract | Irrelevant |
| 973] B.L. Laube, D.Y. Chang, A.N. Blask, B.J. Rosenstein, Radioaerosol assessment of lung improvement in cystic fibrosis patients treated for acute pulmonary exacerbations, Chest 101(5) (1992) 1302-1308. | Excluded | Title and abstract | Irrelevant |
| 974] Pactr, RAF 6047, https://trialsearch.who.int/Trial2.aspx?TrialID=PACTR201803002999356 (2018). | Excluded | Title and abstract | Irrelevant |
| 975] T. Niessink, M. Janssen, C. Otto, T. Jansen, Raman Spectroscopy Integrated with Polarized Light Microscopy for Diagnosis of Crystallopathies, Arthritis and Rheumatology 74 (2022) 3566-3568. | Excluded | Title and abstract | Irrelevant |
| 976] Isrctn, A randomised double blind trial of add-on flunarizine to prevent the cognitive deterioration associated with infantile spasms, https://trialsearch.who.int/Trial2.aspx?TrialID=ISRCTN36757519 (2005). | Excluded | Title and abstract | Irrelevant |
| 977] A. Romera, M.A. Cairols, R. Vila-Coll, X. Martí, E. Colomé, A. Bonell, O. Lapiedra, A randomised open-label trial comparing long-term sub-cutaneous low-molecular-weight heparin compared with oral-anticoagulant therapy in the treatment of deep venous thrombosis, European journal of vascular and endovascular surgery 37(3) (2009) 349‐356. | Excluded | Title and abstract | Irrelevant |
| 978] Ctri, A Randomized control trial on primary school children to assess the efficacy of a protein snack with or without micronutrient fortification to promote vitamin B12 status, https://trialsearch.who.int/Trial2.aspx?TrialID=CTRI/2019/01/017075 (2019). | Excluded | Title and abstract | Irrelevant |
| 979] J. Wong, D. Gupta, A. Nadhim, S. Bhat, P. Polos, A randomized double-blind, placebo controlled trial with cross-over, to assess the efficacy of correcting vitamin D deficiency in improving the symptoms of restless legs syndrome (RLS), Sleep 43(SUPPL 1) (2020) A307‐A308. | Excluded | Full text | Study Protocol |
| 980] A.J. Schou, C. Heuck, O.D. Wolthers, A randomized, controlled lower leg growth study of vitamin D supplementation to healthy children during the winter season, Annals of human biology 30(2) (2003) 214‐219. | Excluded | Title and abstract | Irrelevant |
| 981] L. Grote, L. Leissner, J. Hedner, J. Ulfberg, A randomized, double-blind, placebo controlled, multi-center study of intravenous iron sucrose and placebo in the treatment of restless legs syndrome, Movement Disorders 24(10) (2009) 1445-1452. | Excluded | Title and abstract | Irrelevant |
| 982] P. Chan, T.Y. Huang, Y.J. Chen, W.P. Huang, Y.C. Liu, Randomized, double-blind, placebo-controlled study of the safety and efficacy of vitamin B complex in the treatment of nocturnal leg cramps in elderly patients with hypertension, Journal of clinical pharmacology 38(12) (1998) 1151-1154. | Excluded | Title and abstract | Irrelevant |
| 983] ChiCtr, A Randomized, Double-blind, Placebo-controlled, Multicenter Phase II Clinical Study to Evaluate the Efficacy and Safety of Benfotiamine Tablets in Patients with Mild-to-moderate Altheimer's Disease Treated with Donepezil Hydrochloride, (2018). | Excluded | Title and abstract | Irrelevant |
| 984] K.L. Insogna, K. Briot, E.A. Imel, P. Kamenický, M.D. Ruppe, A.A. Portale, T. Weber, P. Pitukcheewanont, H.I. Cheong, S. Jan de Beur, Y. Imanishi, N. Ito, R.H. Lachmann, H. Tanaka, F. Perwad, L. Zhang, C.Y. Chen, C. Theodore-Oklota, M. Mealiffe, J. San Martin, T.O. Carpenter, A Randomized, Double-Blind, Placebo-Controlled, Phase 3 Trial Evaluating the Efficacy of Burosumab, an Anti-FGF23 Antibody, in Adults With X-Linked Hypophosphatemia: Week 24 Primary Analysis, Journal of Bone and Mineral Research 33(8) (2018) 1383-1393. | Excluded | Title and abstract | Irrelevant |
| 985] I.E. Euctr, A Randomized, Open-Label, Phase 3 Study to Assess the Efficacy and Safety of KRN23 Versus Oral Phosphate and Active Vitamin D Treatment in Pediatric Patients with X-linked Hypophosphatemia (XLH), https://trialsearch.who.int/Trial2.aspx?TrialID=EUCTR2016-000600-29-IE (2016). | Excluded | Title and abstract | Irrelevant |
| 986] B. Hai Binh, P. Ramirez, D. Martinez-Puig, A randomized, placebo-controlled study to evaluate efficacy and safety of a dietary supplement containing mucopolysaccharides, collagen type I and vitamin C for management of different tendinopathies, Annals of the rheumatic diseases 73 (2014). | Excluded | Title and abstract | Irrelevant |
| 987] A. Bourla, F. Ferreri, T. Baudry, V. Panizzi, V. Adrien, S. Mouchabac, Rapid cycling bipolar disorder: Literature review on pharmacological treatment illustrated by a case report on ketamine, Brain and behavior 12(2) (2022). | Excluded | Title and abstract | Irrelevant |
| 988] E. Morsia, K.B. Garvey, S. Rupoli, G. Micucci, A.R. Scortechini, S. Ceglie, A. Olivieri, A rare case of acute promyelocytic leukemia originating from post essential thrombocytemia myelofibrosis, Haematologica 104 (2019) 127. | Excluded | Title and abstract | Irrelevant |
| 989] J.M. Ferro, F. Dentali, J.M. Coutinho, A. Kobayashi, J. Caria, M. Desch, M. Fraessdorf, H. Huisman, H.C. Diener, Rationale, design, and protocol of a randomized controlled trial of the safety and efficacy of dabigatran etexilate versus dose-adjusted warfarin in patients with cerebral venous thrombosis, International journal of stroke 13(7) (2018) 766‐770. | Excluded | Title and abstract | Irrelevant |
| 990] B. Schormair, C. Zhao, A.V. Salminen, K. Oexle, J. Winkelmann, Reassessment of candidate gene studies for idiopathic restless legs syndrome in a large genome-wide association study dataset of European ancestry, Sleep 45(8) (2022). | Excluded | Title and abstract | Irrelevant |
| 991] C.L. Wong, U.S. Dinish, M. Olivo, Recent advances in SPR and SERS for sensitive translational medical diagnostics, Photonics and Lasers in Medicine 4(2) (2015) 119-149. | Excluded | Title and abstract | Irrelevant |
| 992] S.N. Davison, B. Tupala, B.A. Wasylynuk, V. Siu, A. Sinnarajah, J. Triscott, Recommendations for the care of patients receiving conservative kidney management focus on management of CKD and symptoms, Clinical Journal of the American Society of Nephrology 14(4) (2019) 626-634. | Excluded | Title and abstract | Irrelevant |
| 993] S. Chen, P. Chan, S. Sun, H. Chen, B. Zhang, W. Le, C. Liu, G. Peng, B. Tang, L. Wang, Y. Cheng, M. Shao, Z. Liu, Z. Wang, X. Chen, M. Wang, X. Wan, H. Shang, Y. Liu, P. Xu, J. Wang, T. Feng, X. Chen, X. Hu, A. Xie, Q. Xiao, The recommendations of Chinese Parkinson's disease and movement disorder society consensus on therapeutic management of Parkinson's disease, Translational neurodegeneration 5(1) (2016). | Excluded | Title and abstract | Irrelevant |
| 994] R. Briggler, E. Matherne, C. Johnson, K. Boehmer, Recurrent Thrombi in an Obese Patient With History of Bariatric Surgery Despite Anti-Xa Therapy, Journal of Pharmacy Practice 35(5) (2022) 811-816. | Excluded | Title and abstract | Irrelevant |
| 995] M. Tyblova, V. Zikan, M. Luchavova, E. Havrdova, I. Raska, Jr., D. Michalska, A.A. Kubena, Reduced Bone Mineral Density in Women with Multiple Sclerosis, Ceska a Slovenska Neurologie a Neurochirurgie 76(1) (2013) 35-44. | Excluded | Title and abstract | Irrelevant |
| 996] M.A. Cikrikcioglu, Y. Sekin, G. Halac, E. Kilic, S. Kesgin, S. Aydin, N. Ozaras, O. Akan, K. Celik, J. Hamdard, M. Zorlu, C. Karatoprak, M. Cakirca, M. Kiskac, Reduced bone resorption and increased bone mineral density in women with restless legs syndrome, Neurology 86(13) (2016) 1235-41. | Included | Full text | Relationship between vitamins and RLS |
| 997] S. Skodda, T. Müller, Refractory epileptic seizures due to vitamin B6 deficiency in a patient with Parkinson's disease under duodopa® therapy, Journal of Neural Transmission 120(2) (2013) 315-318. | Excluded | Title and abstract | Irrelevant |
| 998] A.S. Tubbs, M.A. Grandner, D. Combs, Refractory insomnia in an adolescent with total blindness, Yale Journal of Biology and Medicine 92(2) (2019) 201-204. | Excluded | Title and abstract | Irrelevant |
| 999] I. Skok, M. Miskulin, G. Vrgoc, G. Zidak, Regional anesthesia for patient with restless legs syndrome: case report, Periodicum Biologorum 115(2) (2013) 253-255. | Excluded | Title and abstract | Irrelevant |
| 1000] M.I. Botez, J.M. Peyronnard, L. Bérubé, R. Labrecque, Relapsing neuropathy, cerebral atrophy and folate deficiency. A close association, Applied neurophysiology 42(3) (1979) 171-83. | Excluded | Title and abstract | Irrelevant |
| 1001] A. Çimen Atalar, The relationship between 25 (Oh) vitamin d level and the severity of disease and sleep quality in restless legs syndrome: Huzursuz bacaklar sendromunda 25 (oh) vitamin d düzeylerinin hastalik Şiddeti ve uyku kalitesi ile olan İlişkisi, Turk Noroloji Dergisi 25(2) (2019) 87-91. | Included | Full text | Relationship between vitamins and RLS |
| 1002] R. Bove, E. Secor, T. Vaughan, T. Chitnis, P. Wicks, P. De Jager, Relationship between body mass index and disease severity in an online multiple sclerosis population, Multiple Sclerosis 18(4) (2012) 85-86. | Excluded | Title and abstract | Irrelevant |
| 1003] N.B. Watts, P. Geusens, I.P. Barton, D. Felsenberg, Relationship between changes in BMD and nonvertebral fracture incidence associated with risedronate: reduction in risk of nonvertebral fracture is not related to change in BMD, Journal of bone and mineral research 20(12) (2005) 2097‐2104. | Excluded | Title and abstract | Irrelevant |
| 1004] R. Zhao, Y. Chen, W. Tan, M. Waly, A. Sharma, P. Stover, A. Rosowsky, B. Malewicz, R.C. Deth, Relationship between dopamine-stimulated phospholipid methylation and the single-carbon folate pathway, J Neurochem 78(4) (2001) 788-96. | Excluded | Title and abstract | Irrelevant |
| 1005] S. Batool-Anwar, A. Malhotra, J. Forman, J. Winkelman, Y. Li, X. Gao, THE RELATIONSHIP BETWEEN RESTLESS LEGS SYNDROME AND HYPERTENSION IN MIDDLE-AGED WOMEN, Sleep 34 (2011) A312-A312. | Excluded | Title and abstract | Irrelevant |
| 1006] Y. Çetinkaya, N. Çömez Yilmaz, R. Türkoǧlu, M. Gencer, H. Tireli, The relationship between tension-type headache patients with anemia and restlessleg syndrome, Journal of Neurological Sciences 26(3) (2009) 305-310. | Excluded | Title and abstract | Irrelevant |
| 1007] Y. Cetinkaya, N.C. Yilmaz, R. Turkoglu, M. Gencer, H. Tireli, The Relationship Between Tension-Type Headache Patients With Anemia and Restless-leg Syndrome, Journal of Neurological Sciences-Turkish 26(3) (2009) 305-310. | Excluded | Title and abstract | Irrelevant |
| 1008] G. Sağlam, G. Pektaş, S. Karakullukçu, B.A. Pektaş, D.S. Aykut, The relationship between vitamin d deficiency and restless legs syndrome in pregnancy, Journal of Turkish Sleep Medicine 7(2) (2020) 44-48. | Included | Full text | Relationship between vitamins and RLS |
| 1009] P. López-Méndez, M. Sosa-Henríquez, Á. Ruiz-Pérez, Relationship between vitamin D deficiency and visually evoked potentials in multiple sclerosis, Archivos de la Sociedad Espanola de Oftalmologia 91(5) (2016) 217-222. | Excluded | Title and abstract | Irrelevant |
| 1010] G. Gonzalez Santiago, J.E. Rodriguez-Ramos, F. Caraballo Garcia, J.R. Aleman-Ortiz, REM Sleep Behavior Disorder in a Patient with Parkinson Disease, American Journal of Respiratory and Critical Care Medicine 203(9) (2021). | Excluded | Title and abstract | Irrelevant |
| 1011] S. Walsh, Remote ischemic preconditioning reduces renal injury during endovascular aneurysm repair: randomized controlled trial, Vascular 17 (2009) S71‐S72. | Excluded | Title and abstract | Irrelevant |
| 1012] M. Gratwicke, K.H. Miles, D.B. Pyne, K.L. Pumpa, B. Clark, Reply to dunican, i.C.; walsh, j.h. comment on “gratwicke et al. nutritional interventions to improve sleep in team-sport athletes: A narrative review. nutrients 2021, 13, 1586”, Nutrients 13(9) (2021). | Excluded | Title and abstract | Irrelevant |
| 1013] M. Gratwicke, K.H. Miles, D.B. Pyne, K.L. Pumpa, B. Clark, Reply to Dunican, IC; Walsh, JH Comment on "Gratwicke et al. Nutritional Interventions to Improve Sleep in Team-Sport Athletes: A Narrative Review. <i>Nutrients</i> 2021, <i>13</i>, 1586", Nutrients 13(9) (2021). | Excluded | Title and abstract | Irrelevant |
| 1014] R.J. Woodhouse, Report from Great Britain, Pharmazeutische Industrie 71(11) (2009) 1919-1923. | Excluded | Title and abstract | Irrelevant |
| 1015] R. Garten, J. Lee, J. Groot, M. Rossman, H. Clifton, W. Wray, R. Richardson, Rescuing age-related vascular dysfunction: the impact of tetrahydrobiopterin and antioxidants, FASEB journal 30(no pagination) (2016). | Excluded | Title and abstract | Irrelevant |
| 1016] Nct, Residual Vein Thrombosis Establishes the Optimal Duration of Oral Anticoagulants, https://clinicaltrials.gov/show/NCT00438230 (2007). | Excluded | Title and abstract | Irrelevant |
| 1017] C. Corrales, Resistant unilateral restless leg syndrome associated with untreated sleep apnea, Sleep 37 (2014) A394. | Excluded | Title and abstract | Irrelevant |
| 1018] D.E. McCarty, Resolution of hypersomnia following identification and treatment of vitamin d deficiency, Journal of clinical sleep medicine : JCSM : official publication of the American Academy of Sleep Medicine 6(6) (2010) 605-8. | Excluded | Title and abstract | Irrelevant |
| 1019] R.T. Liu, J.H. Yang, X. Wu, Z.M. Li, S.X. Sun, F. Huang, Resonance Rayleigh light-scattering of thiamine hydrochloride-nucleic acids-cetyl trimethyl ammonium bromide systems and their analytical application, Journal of Trace and Microprobe Techniques 20(3) (2002) 363-376. | Excluded | Title and abstract | Irrelevant |
| 1020] M. Manconi, L. Ferini-Strambi, W.A. Hening, Response to Clinical Corners case (Sleep Medicine 6/2: 83-4):: Pregnancy associated with daytime sleepiness and nighttime restlessness, Sleep medicine 6(5) (2005) 477-478. | Excluded | Title and abstract | Irrelevant |
| 1021] P.T. Trzepacz, E.J. Violette, M.J. Sateia, Response to opioids in three patients with restless legs syndrome, American Journal of Psychiatry 141(8) (1984) 993-995. | Excluded | Title and abstract | Irrelevant |
| 1022] K. Vishwakarma, J. Kalra, R. Gupta, M. Sharma, T. Sharma, Response to reader's queries, Annals of Indian Academy of Neurology 20(2) (2017) 167-169. | Excluded | Title and abstract | Irrelevant |
| 1023] B.B. Koo, Restless Leg Syndrome Across the Globe: Epidemiology of the Restless Legs Syndrome/Willis-Ekbom Disease, Sleep Medicine Clinics 10(3) (2015) 189-205. | Excluded | Title and abstract | Irrelevant |
| 1024] G. Saricam, O. Saricam, Restless leg syndrome and migraine: Is there a common etiology?, Neurology Asia 26(4) (2021) 743-749. | Included | Full text | Relationship between vitamins and RLS |
| 1025] M. Terzi, D. Yazici, M. Onar, Restless leg syndrome and multiple sclerosis, Multiple Sclerosis 20(1) (2014) 467-468. | Excluded | Title and abstract | Irrelevant |
| 1026] N. Yaǧli, Z. Yücehan, İ. Karaarslan, D. Demiröz, S. Çalişir, İ. Eren, Restless leg syndrome developing due to usage of mirtazapine, Psychiatry and Clinical Psychopharmacology 29 (2019) 240. | Excluded | Title and abstract | Irrelevant |
| 1027] C.P.L. Hoo, W.L. Lau, C.H. Ko, Restless leg syndrome in a child and an adolescent with excellent responses to iron replacement therapy, Hong Kong Journal of Paediatrics 26(1) (2021) 38-41. | Excluded | Title and abstract | Irrelevant |
| 1028] S. Işıkay, N. Işıkay, H. Per, K.B. Çarman, H. Kocamaz, Restless leg syndrome in children with celiac disease, The Turkish journal of pediatrics 60(1) (2018) 70-75. | Included | Full text | Relationship between vitamins and RLS |
| 1029] S.G. Matar, Z.S. El-Nahas, H. Aladwan, M. Hasanin, S.M. Elsayed, A.Z. Nourelden, A.Y. Benmelouka, K.M. Ragab, Restless Leg Syndrome in Hemodialysis Patients: A Narrative Review, The neurologist 27(4) (2022) 194-202. | Excluded | Title and abstract | Irrelevant |
| 1030] S.A. Hamed, S.K. Abdulhamid, A.F. El-Hadad, M. Fawzy, M.A. Abd-Elhamed, Restless leg syndrome in patients with chronic kidney disease: a hospital-based study from Upper Egypt, International Journal of Neuroscience 133(3) (2023) 257-268. | Excluded | Full text | No relevant outcome |
| 1031] S. Bose, S. Jacob, Restless leg syndrome in patients with liver cirrhosis: Waiting for the shoe to drop, Neurology India 67(3) (2019) 657-659. | Excluded | Title and abstract | Irrelevant |
| 1032] S.S. Khan, J.L. Ortstadt, G.S. Carter, Restless leg syndrome/Willis Ekbom Disease in a patient with C282Y/H63D negative hemochromatosis, Sleep 39 (2016) A401-A402. | Excluded | Title and abstract | Irrelevant |
| 1033] P.E. Cotter, S.T. O'Keeffe, Restless leg syndrome: Is it a real problem?, Therapeutics and Clinical Risk Management 2(4) (2006) 465-475. | Excluded | Title and abstract | Irrelevant |
| 1034] A. Amir, R.M. Masterson, A. Halim, A. Nava, Restless Leg Syndrome: Pathophysiology, Diagnostic Criteria, and Treatment, Pain Medicine (United States) 23(5) (2022) 1032-1035. | Excluded | Title and abstract | Irrelevant |
| 1035] J. Wersall, Restless legs, Svenska lakartidningen 49(32) (1952) 2032-4. | Excluded | Title and abstract | Irrelevant |
| 1036] E. Filseth, Restless legs, Tidsskrift for den Norske Laegeforening 124(6) (2004) 832. | Excluded | Title and abstract | Irrelevant |
| 1037] F. Hürlimann, Restless legs and crampi in the night. Double blind study with Circonyl in patients with defective peripheric arterial circulation (author's transl), Schweizerische Rundschau fur Medizin Praxis 63(7) (1974) 194‐195. | Excluded | Title and abstract | Irrelevant |
| 1038] C. Bernick, L.Z. Stern, Restless legs syndrome, Western Journal of Medicine 145(2) (1986) 263-265. | Excluded | Title and abstract | Irrelevant |
| 1039] H.O. Dustmann, K. Steinbrueck, Restless legs syndrome, Orthopadische Praxis 16(8) (1980) 671-672. | Excluded | Title and abstract | Irrelevant |
| 1040] D. Garcia-Borreguero, Restless Legs Syndrome, Journal of the neurological sciences 238 (2005) S32-S33. | Excluded | Title and abstract | Irrelevant |
| 1041] J. Komar, E. Polay, The restless legs syndrome, Munchener medizinische Wochenschrift (1950) 112(31) (1970) 1412-5. | Excluded | Title and abstract | Irrelevant |
| 1042] S. Lesage, C.J. Earley, Restless legs syndrome, Current Treatment Options in Neurology 6(3) (2004) 209-219. | Excluded | Title and abstract | Irrelevant |
| 1043] P. Odin, M. Mrowka, M. Shing, Restless legs syndrome, European journal of neurology 9(SUPPL. 3) (2002) 59-67. | Excluded | Title and abstract | Irrelevant |
| 1044] S. Schofield, Restless legs syndrome, Hospital Pharmacy Practice 4(2) (1994) 75. | Excluded | Title and abstract | Irrelevant |
| 1045] H. Singh, S. Verma, S. Aggarwal, Restless Legs Syndrome, Journal, Indian Academy of Clinical Medicine 9(3) (2008) 188-192. | Excluded | Title and abstract | Irrelevant |
| 1046] K. Krauße, Restless legs syndrome - A poorly recognized syndrome, KIM - Komplementare und Integrative Medizin, Artztezeitschrift fur Naturheilverfahren 49(8) (2008) 31-33. | Excluded | Title and abstract | Irrelevant |
| 1047] G.W. Paulson, Restless legs syndrome - How to provide symptom relief with drug and nondrug therapies, Geriatrics 55(4) (2000) 35-+. | Excluded | Title and abstract | Irrelevant |
| 1048] D.S. Erdogan, G.B. Senel, A. Gunduz, B.P. Ucar, T. Elverdi, A. Salihoglu, M.C. Ar, S. Ongoren, Z. Baslar, A.E. Eskazan, Restless Legs Syndrome (RLS) in Patients (pts) with Polycythemia Vera (PV): Is Iron Deficiency (ID) the Only Culprit?, Blood 140 (2022) 12278-12279. | Excluded | Title and abstract | Irrelevant |
| 1049] A. Walters, D. Gabelia, B. Frauscher, Restless Legs Syndrome (Willis Ekbom Disease) and growing pains, are they the same thing ?: A side by side comparison of the diagnostic criteria for both and recommendations for future research, Sleep medicine 14 (2013) e300-e301. | Excluded | Title and abstract | Irrelevant |
| 1050] A.S. Walters, D. Gabelia, B. Frauscher, Restless legs syndrome (Willis-Ekbom disease) and growing pains: are they the same thing? A side-by-side comparison of the diagnostic criteria for both and recommendations for future research, Sleep medicine 14(12) (2013) 1247-52. | Excluded | Title and abstract | Irrelevant |
| 1051] S.O. Wali, A.F. Alkhouli, Restless legs syndrome among Saudi end-stage renal disease patients on hemodialysis, Saudi Medical Journal 36(2) (2015) 204-210. | Excluded | Full text | Data couldn't be separately extracted |
| 1052] S. Ma, X. Shang, Y. Guo, G. Liu, J. Yang, R. Xue, Restless legs syndrome and hypertension in Chinese pregnant women, Neurological Sciences 36(6) (2015) 877-881. | Excluded | Title and abstract | Irrelevant |
| 1053] S. Batool-Anwar, A. Malhotra, J. Forman, J. Winkelman, Y. Li, X. Gao, Restless legs syndrome and hypertension in middle-aged women, Hypertension (Dallas, Tex. : 1979) 58(5) (2011) 791-6. | Excluded | Full text | Data couldn't be separately extracted |
| 1054] L.I. Goulart, R.N. Delgado Rodrigues, M.F. Prieto Peres, Restless Legs Syndrome and Pain Disorders: What's in common?, Current Pain and Headache Reports 18(11) (2014). | Excluded | Title and abstract | Irrelevant |
| 1055] A.N. Rama, C.A. Kushida, Restless legs syndrome and periodic limb movement disorder, Medical Clinics of North America 88(3) (2004) 653-667. | Excluded | Title and abstract | Irrelevant |
| 1056] M. Karatas, Restless legs syndrome and periodic limb movements during sleep: Diagnosis and treatment, The neurologist 13(5) (2007) 294-301. | Excluded | Title and abstract | Irrelevant |
| 1057] A.S. Walters, Restless legs syndrome and periodic limb movements in sleep, CONTINUUM Lifelong Learning in Neurology 13(3 SLEEP DISORDERS) (2007) 115-138. | Excluded | Title and abstract | Irrelevant |
| 1058] B.R. Krueger, Restless legs syndrome and periodic movements of sleep, Mayo Clinic proceedings 65(7) (1990) 999-1006. | Excluded | Title and abstract | Irrelevant |
| 1059] M. Manconi, V. Govoni, A. De Vito, N.T. Economou, E. Cesnik, I. Casetta, G. Mollica, L. Ferini-Strambi, E. Granieri, Restless legs syndrome and pregnancy, Neurology 63(6) (2004) 1065-9. | Included | Full text | Relationship between vitamins and RLS |
| 1060] S. Ismailogullari, A. Ozturk, M.M. Mazicioglu, S. Serin, M. Gultekin, M. Aksu, Restless legs syndrome and pregnancy in Kayseri, Turkey: A hospital based survey, Sleep and Biological Rhythms 8(2) (2010) 137-143. | Excluded | Title and abstract | Irrelevant |
| 1061] J.-P. Neau, A. Porcheron, S. Mathis, A. Julian, J.-C. Meurice, J. Paquereau, G. Godeneche, J. Ciron, G. Bouche, Restless Legs Syndrome and Pregnancy: A Questionnaire Study in the Poitiers District, France, European neurology 64(5) (2010) 268-274. | Excluded | Full text | Data couldn't be separately extracted |
| 1062] P. Srivanitchapoom, S. Pandey, M. Hallett, Restless legs syndrome and pregnancy: a review, Parkinsonism Relat Disord 20(7) (2014) 716-22. | Excluded | Title and abstract | Irrelevant |
| 1063] J.-P. Neau, P. Marion, S. Mathis, A. Julian, G. Godeneche, D. Larrieu, J.-C. Meurice, J. Paquereau, P. Ingrand, Restless Legs Syndrome and Pregnancy: Follow-Up of Pregnant Women before and after Delivery, European neurology 64(6) (2010) 361-366. | Excluded | Full text | No relevant outcome |
| 1064] R. Gupta, M. Dhyani, T. Kendzerska, S.R. Pandi-Perumal, A.S. BaHammam, P. Srivanitchapoom, S. Pandey, M. Hallett, Restless legs syndrome and pregnancy: prevalence, possible pathophysiological mechanisms and treatment, Acta neurologica Scandinavica 133(5) (2016) 320-9. | Excluded | Title and abstract | Irrelevant |
| 1065] A.C. Winter, M. Schürks, R.J. Glynn, J.E. Buring, J.M. Gaziano, K. Berger, T. Kurth, Restless legs syndrome and risk of incident cardiovascular disease in women and men: Prospective cohort study, BMJ open 2(2) (2012). | Excluded | Title and abstract | Irrelevant |
| 1066] K.A. Lee, M.E. Zaffke, K. Baratte-Beebe, Possible association between vitamin B12 deficiency and restless legs syndrome: the role of folate and iron, Journal of women's health & gender-based medicine 10(4) (2001) 335-41. | Included | Full text | Relationship between vitamins and RLS |
| 1067] U. Goswami, S. Pusalavidyasagar, Restless legs syndrome associated with use of stevia nonnutritive sweetener, Journal of Clinical Sleep Medicine 6(10) (2020) 1819-1821. | Excluded | Title and abstract | Irrelevant |
| 1068] A. Yucel, H. Ozcan, N. Yucel, U. Aydinoglu, Restless legs syndrome due to use of mirtazapine and treatment with pramipexole, Klinik Psikofarmakoloji Bulteni 24 (2014) S138. | Excluded | Title and abstract | Irrelevant |
| 1069] I. Hudić, Z. Ercegović, L. Kamerić, A. Hadžimehedović, L. Mešalić, L.D. Hudić, E. Kačar, A. Muratović, RESTLESS LEGS SYNDROME DURING PREGNANCY AND EARLY PUERPERAL PERIOD IN WOMEN OF BOSNIA AND HERZEGOVINA, Acta Medica Saliniana 50(1-2) (2021) 52-55. | Excluded | Title and abstract | Irrelevant |
| 1070] S. Miri, M. Vahdat, E. Sariri, M. Rohani, A. Sabet, Restless legs syndrome during pregnancy: Clinical characteristics and outcomes in Iranian pregnant women, Movement Disorders 27 (2012) S403. | Excluded | Full text | Duplication |
| 1071] M. Maheswaran, C.A. Kushida, Restless legs syndrome in children, MedGenMed Medscape General Medicine 8(2) (2006). | Excluded | Title and abstract | Irrelevant |
| 1072] M.B. Filiz, S. Filiz, R.T. Baran, T. Çakır, Ş.K. Doğan, M. Parlak, N.F. Toraman, Restless legs syndrome in children with allergic rhinitis: A comparative study on frequency, severity and sleep quality, Turkish journal of physical medicine and rehabilitation 64(3) (2018) 198-204. | Excluded | Full text | No relevant outcome |
| 1073] G. Romano, M. Messa, G. Merlino, G. Ferrara, R. Mioni, S. Leonardi, C. Fregonese, A. Marega, I. Iskra, S. Lorenzut, G. Gigli, D. Montanaro, Restless legs syndrome in chronic hemodialysed patients: Preliminary data, NDT Plus 3 (2010) iii241-iii242. | Excluded | Title and abstract | Irrelevant |
| 1074] Y. Safarpour, N.D. Vaziri, B. Jabbari, Restless Legs Syndrome in Chronic Kidney Disease- a Systematic Review, Tremor Other Hyperkinet Mov (N Y) 13 (2023) 10. | Excluded | Title and abstract | Irrelevant |
| 1075] M. Aksu, S. Ismailogullari, B. Korkmaz, S. Korkmaz, M. Kocyigit, Restless legs syndrome in chronic renal failure patients under continuous ambulatory peritoneal dialysis, Parkinsonism and Related Disorders 15 (2009) S199. | Included | Full text | Relationship between vitamins and RLS |
| 1076] F. Porta, A. Neirotti, M. Spada, Restless legs syndrome in DNAJC12 deficiency, Neurological Sciences 44(6) (2023) 2167-2172. | Excluded | Title and abstract | Irrelevant |
| 1077] G.L. Gigli, M. Adorati, P. Dolso, A. Piani, M. Valente, S. Brotini, R. Budai, Restless legs syndrome in end-stage renal disease, Sleep medicine 5(3) (2004) 309-315. | Excluded | Full text | No relevant outcome |
| 1078] Nct, Restless Legs Syndrome in Hemodialysis Patients, https://clinicaltrials.gov/show/NCT03337529 (2017). | Excluded | Title and abstract | Irrelevant |
| 1079] A.E. Naini, M. Masoumi, M. Mortazavi, A. Gholamrezaei, B. Amra, Restless legs syndrome in patients on maintenance hemodialysis and peritoneal dialysis, Journal of Research in Medical Sciences 17 (2012) S265-S272. | Included | Full text | Relationship between vitamins and RLS |
| 1080] A. Önalan, Z. Matur, M. Pehlivan, G. Akman, Restless legs syndrome in patients with behçet’s disease and multiple sclerosis: Prevalence, associated conditions and clinical features, Noropsikiyatri Arsivi 57(1) (2020) 3-8. | Excluded | Title and abstract | Irrelevant |
| 1081] H. Kumru, E. Portell, M. Barrio, J. Santamaria, Restless legs syndrome in patients with sequelae of poliomyelitis, Parkinsonism and Related Disorders 20(10) (2014) 1056-1058. | Excluded | Title and abstract | Irrelevant |
| 1082] B.C. Yalcinkaya, C.B. Amirov, S. Saltik, G.B. Senel, Restless legs syndrome in pediatric onset multiple sclerosis, Mult Scler Relat Disord 56 (2021) 103295. | Included | Full text | Relationship between vitamins and RLS |
| 1083] M.C. Trindade, T. Bittencourt, G. Lorenzi-Filho, R.C. Alves, D.C. de Andrade, E.T. Fonoff, E. Bor-Seng-Shu, A.A. Machado, M.J. Teixeira, E.R. Barbosa, G.G. Tribl, Restless legs syndrome in Wilson's disease: frequency, characteristics, and mimics, Acta neurologica Scandinavica 135(2) (2017) 211-218. | Excluded | Title and abstract | Irrelevant |
| 1084] R.L. Page Ii, J.M. Ruscin, J.L. Bainbridge, A.A. Brieke, Restless legs syndrome induced by escitalopram: Case report and review of the literature, Pharmacotherapy 28(2) (2008) 271-280. | Excluded | Title and abstract | Irrelevant |
| 1085] J. Becker, F. Berger, K.A. Schindlbeck, D. Poddubnyy, P.M. Koch, J.C. Preiß, B. Siegmund, F. Marzinzik, J. Maul, Restless legs syndrome is a relevant comorbidity in patients with inflammatory bowel disease, Int J Colorectal Dis 33(7) (2018) 955-962. | Included | Full text | Relationship between vitamins and RLS |
| 1086] L.B. Weinstock, J.B. Brook, A.S. Walters, A. Goris, L.B. Afrin, G.J. Molderings, Restless legs syndrome is associated with long-COVID in women, Journal of Clinical Sleep Medicine 18(5) (2022) 1413-1418. | Excluded | Full text | No relevant outcome |
| 1087] S. Demirci, K. Demirci, A. Doğru, E.E. İnal, H.R. Koyuncuoğlu, M. Şahin, Restless legs syndrome is associated with poor sleep quality and quality of life in patients with ankylosing spondylitis: a questionnaire-based study, Acta neurologica Belgica 116(3) (2016) 329-336. | Included | Full text | Relationship between vitamins and RLS |
| 1088] H. Terzi, R. Terzi, B. Zeybek, M. Ergenoglu, S. Hacivelioglu, A. Akdemir, O. Yeniel, Restless legs syndrome is related to obstructive sleep apnea symptoms during pregnancy, Sleep and Breathing (2014) 1-6. | Excluded | Title and abstract | Duplication |
| 1089] H. Terzi, R. Terzi, B. Zeybek, M. Ergenoglu, S. Hacivelioglu, A. Akdemir, O. Yeniel, Restless legs syndrome is related to obstructive sleep apnea symptoms during pregnancy, Sleep & breathing = Schlaf & Atmung 19(1) (2015) 73-8. | Excluded | Full text | Data couldn't be separately extracted |
| 1090] S. Akpinar, Restless legs syndrome treatment with dopaminergic drugs, Clinical neuropharmacology 10(1) (1987) 69-79. | Excluded | Title and abstract | Irrelevant |
| 1091] S. Prakash, R.J. Bhanvadia, N.D. Shah, Restless legs syndrome with carbamazepine-induced osteomalacia: causal or casual association, Gen Hosp Psychiatry 32(2) (2010) 228.e1-3. | Excluded | Title and abstract | Irrelevant |
| 1092] G.W. Paulson, Restless legs syndrome. How to provide symptom relief with drug and nondrug therapies, Geriatrics 55(4) (2000) 35-8, 43-4, 47-8. | Excluded | Title and abstract | Irrelevant |
| 1093] N. Mavroudakis, Restless legs syndrome. Recommended drug management in 2007, Revue Medicale de Bruxelles 28(4) (2007) 325-328. | Excluded | Title and abstract | Irrelevant |
| 1094] G. Sünter, Ö. Kilinç, A. Berk, S. Akçabey, E. Saldüz, H. Öztürkçü, D.İ. Günal, K. Agan, Restless legs syndrome/willis-ekbom disease in multiple sclerosis patients with spinal cord lesions, Noropsikiyatri Arsivi 57(4) (2020) 299-302. | Included | Full text | Relationship between vitamins and RLS |
| 1095] R. Gupta, V. Lahan, D. Goel, Restless Legs Syndrome: A common disorder, but rarely diagnosed and barely treated - an Indian experience, Sleep medicine 13(7) (2012) 838-841. | Excluded | Title and abstract | Irrelevant |
| 1096] R. Gupta, V. Lahan, D. Goel, Restless Legs Syndrome: a common disorder, but rarely diagnosed and barely treated--an Indian experience, Sleep medicine 13(7) (2012) 838-41. | Excluded | Title and abstract | Irrelevant |
| 1097] P. Yeh, A.S. Walters, J.W. Tsuang, Restless legs syndrome: A comprehensive overview on its epidemiology, risk factors, and treatment, Sleep and Breathing 16(4) (2012) 987-1007. | Excluded | Title and abstract | Irrelevant |
| 1098] S. Chokroverty, J. Jankovic, Restless legs syndrome: A disease in search of identity, Neurology 52(5) (1999) 907-910. | Excluded | Title and abstract | Irrelevant |
| 1099] I. Machtey, Restless legs syndrome: A metabolic disorder? , Archives of internal medicine 156(20) (1996) 2386. | Excluded | Title and abstract | Irrelevant |
| 1100] P. Agarwal, A. Griffith, Restless legs syndrome: A unique case and essentials of diagnosis and treatment, MedGenMed Medscape General Medicine 10(12) (2008). | Excluded | Title and abstract | Irrelevant |
| 1101] A. Nasir, A.K. Khuwaja, Restless legs syndrome: Common but frequently unrecognized disorder in pregnancy, Journal of the Pakistan Medical Association 57(3) (2007) 164. | Excluded | Title and abstract | Irrelevant |
| 1102] G.R. Zanni, J.Y. Wick, Restless legs syndrome: Finding relief, 2010. | Excluded | Title and abstract | Irrelevant |
| 1103] J.F. Avecillas, J.A. Golish, C. Giannini, J.C. Yataco, Restless legs syndrome: Keys to recognition and treatment, Cleveland Clinic journal of medicine 72(9) (2005) 769-787. | Excluded | Title and abstract | Irrelevant |
| 1104] L.R. Patrick, Restless legs syndrome: pathophysiology and the role of iron and folate, Alternative medicine review : a journal of clinical therapeutic 12(2) (2007) 101-12. | Excluded | Title and abstract | Irrelevant |
| 1105] R.D.C. Silva Filho, C.F. Conti, M.M. De Oliveira, J.S. Valbuza, G.F. Do Prado, Restless Legs Syndrome: Review and up date, Revista Neurociencias 17(3) (2009) 263-269. | Excluded | Title and abstract | Irrelevant |
| 1106] A. Singh, S. Avasthi, R.P. Singh, Restless legs syndrome: Should we consider it as a differential diagnosis? Observations on 10 patients, Journal of Internal Medicine of India 9(2) (2006) 41-44. | Excluded | Title and abstract | Irrelevant |
| 1107] J. Wersall, Restless legs], Svenska lakartidningen 49(32) (1952) 2032-4. | Excluded | Title and abstract | Irrelevant |
| 1108] G. Neuhäuser, »Restless legs« and »burning feet«, Padiatrische Praxis 64(1) (2003) 31. | Excluded | Title and abstract | Irrelevant |
| 1109] D. Athauda, G. Leschziner, A restless night's sleep, BMJ (Online) 344(7844) (2012) 48. | Excluded | Title and abstract | Irrelevant |
| 1110] R. Gupta, V. Lahan, D. Goel, Restlessness in right upper limb as sole presentation of restless legs syndrome, Journal of neurosciences in rural practice 4(1) (2013) 78-80. | Excluded | Title and abstract | Irrelevant |
| 1111] J.F. Anson, W.G. Hinson, J.L. Pipkin, R.F. Kwarta, D.K. Hansen, J.F. Young, E.R. Burns, D.A. Casciano, Retinoic acid induction of stress proteins in fetal mouse limb buds, Developmental biology 121(2) (1987) 542-7. | Excluded | Title and abstract | Irrelevant |
| 1112] R.A. Merrill, Retinoic acid regulated genes in neuronal development, 2004. | Excluded | Title and abstract | Irrelevant |
| 1113] Y. Hou, J. Liu, M. Hong, X. Li, Y. Ma, Q. Yue, C.-Z. Li, A reusable aptasensor of thrombin based on DNA machine employing resonance light scattering technique, Biosensors & Bioelectronics 92 (2017) 259-265. | Excluded | Title and abstract | Irrelevant |
| 1114] R.W. Evans, Reversible palinopsia and the Alice in wonderland syndrome associated with topiramate use in migraineurs, Headache 46(5) (2006) 815-818. | Excluded | Title and abstract | Irrelevant |
| 1115] M.A. O'Neal, A Review of Women's Neurology, American Journal of Medicine 131(7) (2018) 735-744. | Excluded | Title and abstract | Irrelevant |
| 1116] M. Perkovič-Benedik, M. Zaletel, N. Pečarič-Meglič, T. Podnar, A right-to-left shunt and prothrombotic disorders in pediatric patients presenting with transient ischemic attack, European Journal of Pediatrics 172(2) (2013) 239-245. | Excluded | Title and abstract | Irrelevant |
| 1117] P.H. Chen, K.C. Liou, C.P. Chen, S.J. Cheng, Risk factors and prevalence rate of restless legs syndrome among pregnant women in Taiwan, Sleep medicine 13(9) (2012) 1153-7. | Excluded | Full text | Data couldn't be separately extracted |
| 1118] P. Lindmarker, S. Schulman, The risk of ipsilateral versus contralateral recurrent deep vein thrombosis in the leg. The DURAC Trial Study Group, Journal of internal medicine 247(5) (2000) 601‐606. | Excluded | Title and abstract | Irrelevant |
| 1119] R. Peraita-Adrados, P. Medrano-Martínez, L. Lillo-Triguero, RLS with plms in a child with hemolytic anemia caused by pyruvate kinase deficiency, Sleep medicine 64 (2019) S298. | Excluded | Title and abstract | Irrelevant |
| 1120] R. Peraita-Adrados, P. Medrano-Martinez, P. Peirano, C. Algarín, L. Lillo-Triguero, RLS with PLMS in a child with hemolytic anemia caused by pyruvate kinase deficiency, Sleep medicine 69 (2020) 100-102. | Excluded | Title and abstract | Irrelevant |
| 1121] Y. Zhang, X. She, G. Zhang, Role and interrelationship of PTPases and H<sub>2</sub>O<sub>2</sub> in light/dark-regulated stomatal movement in <i>Vicia faba</i>, Australian Journal of Botany 57(6) (2009) 486-494. | Excluded | Title and abstract | Irrelevant |
| 1122] P. Gossett, C. Maxwell, S. Hazelett, K. Allen, The role of a geriatric consult team in improving delirium outcomes, Journal of the American Geriatrics Society 58 (2010) S20. | Excluded | Title and abstract | Irrelevant |
| 1123] V. Beltran-Beltra, N. Benetó, T. Lapeña-Luzón, L.R. Rodríguez, F.V. Pallardó, P. Gonzalez-Cabo, Role of Adenosine Receptors in Rare Neurodegenerative Diseases with Motor Symptoms, Current Protein and Peptide Science 22(9) (2021) 675-694. | Excluded | Title and abstract | Irrelevant |
| 1124] B. Elstrott, L. Khan, S. Olson, V. Raghunathan, T. DeLoughery, J.J. Shatzel, The role of iron repletion in adult iron deficiency anemia and other diseases, European Journal of Haematology 104(3) (2020) 153-161. | Excluded | Title and abstract | Irrelevant |
| 1125] H. Lahoda Brodska, J. Klempir, J. Zavora, P. Kohout, The Role of Micronutrients in Neurological Disorders, Nutrients 15(19) (2023). | Excluded | Title and abstract | Irrelevant |
| 1126] C.K. Odoh, X. Guo, J.T. Arnone, X. Wang, Z.K. Zhao, The role of NAD and NAD precursors on longevity and lifespan modulation in the budding yeast, Saccharomyces cerevisiae, Biogerontology 23(2) (2022) 169-199. | Excluded | Title and abstract | Irrelevant |
| 1127] A. Becchetti, P. Aracri, S. Meneghini, S. Brusco, A. Amadeo, The role of nicotinic acetylcholine receptors in autosomal dominant nocturnal frontal lobe epilepsy, Frontiers in Physiology 6 (2015). | Excluded | Title and abstract | Irrelevant |
| 1128] N. Sinn, J. Rucklidge, The role of nutrition and diet in learning and behaviour of children with symptoms of attention deficit hyperactivity disorder, in: D. Benton (Ed.), Lifetime Nutritional Influences on Cognition, Behaviour and Psychiatric Illness2011, pp. 323-358. | Excluded | Title and abstract | Irrelevant |
| 1129] Ctri, ROLE OF VITAMIN C AND THIAMINE IN SEPSIS INDUCED REFRACTORY HYPOTENSION, https://trialsearch.who.int/Trial2.aspx?TrialID=CTRI/2021/02/031043 (2021). | Excluded | Title and abstract | Irrelevant |
| 1130] Nct, Role of Vitamin C at 6 Months on Incidence of Complex Regional Pain Syndrome Type I in Upper Limb Surgery, https://clinicaltrials.gov/show/NCT02390505 (2015). | Excluded | Title and abstract | Irrelevant |
| 1131] A. Otocka-Kmiecik, A. Król, The Role of Vitamin C in Two Distinct Physiological States: Physical Activity and Sleep, Nutrients 12(12) (2020). | Excluded | Title and abstract | Irrelevant |
| 1132] C.N. Homann, B. Homann, The Role of Vitamin D in Basal Ganglia Diseases, Journal of integrative neuroscience 21(6) (2022) 155. | Excluded | Title and abstract | Irrelevant |
| 1133] F. Prono, K. Bernardi, R. Ferri, O. Bruni, The Role of Vitamin D in Sleep Disorders of Children and Adolescents: A Systematic Review, International journal of molecular sciences 23(3) (2022). | Excluded | Title and abstract | Irrelevant |
| 1134] R. Silvestri, I. Arico, A. Brigandi, M. Cafarelli, L. Mirci, The role of vitamin D supplementation in Willis/Ekbom, Restless Leg Syndrome (WES/RLS). A new therapeutic option to improve symptoms and augmentation, Journal of Sleep Research 27 (2018). | Excluded | Full text | No specific data |
| 1135] Nct, Role of Vitamin D Therapy in Recovery From Early Neonatal Sepsis (Randomized Controlled Trial), https://clinicaltrials.gov/ct2/show/NCT05969327 (2023). | Excluded | Title and abstract | Irrelevant |
| 1136] P. Das, A. Chopra, S. Ibrahim, Ropinirole therapy for hypersomnia as a manifestation of periodic limb movement disorder: A case report, Sleep 36 (2013) A442-A443. | Excluded | Title and abstract | Irrelevant |
| 1137] R. Gomez Almendros, L. Kanaan Kanaan, R. Campos Del Portillo, B. Olivan Palacios, J.A. Rivera Bautista, P. Pla Sanchez, M.E. Pelayo Delgado, M.J. Garcia-Oria Serrano, Roux-en-Y gastric bypass vs one-anastomosis gastric bypass: post surgery nutritional values comparison, Obesity surgery 29(5) (2019) 56. | Excluded | Title and abstract | Irrelevant |
| 1138] J.M. Ferro, J.M. Coutinho, F. Dentali, A. Kobayashi, A. Alasheev, P. Canhao, D. Karpov, S. Nagel, L. Posthuma, J.M. Roriz, et al., Safety and efficacy of dabigatran etexilate vs dose-adjusted warfarin in patients with cerebral venous thrombosis: a randomized clinical trial, JAMA neurology (2019). | Excluded | Title and abstract | Irrelevant |
| 1139] Nct, Safety and Efficacy Study of Qualia Mind on Cognition in a Healthy Population, https://clinicaltrials.gov/show/NCT04389723 (2020). Excluded Title and abstract Irrelevant | Excluded | Title and abstract | Irrelevant |
| 1140] L.J. Lea, P.A. Hepburn, Safety evaluation of phytosterol-esters. Part 9: Results of a European post-launch monitoring programme, Food and Chemical Toxicology 44(8) (2006) 1213-1222. | Excluded | Title and abstract | Irrelevant |
| 1141] T. Yamamoto, M. Tsujimoto, H. Sowa, Safety of daily teriparatide treatment: a post hoc analysis of a Phase III study to investigate the possible association of teriparatide treatment with calcium homeostasis in patients with serum procollagen type I N-terminal propeptide elevation, Clinical interventions in aging 10 (2015) 1101‐1109. | Excluded | Title and abstract | Irrelevant |
| 1142] Salmonellosis paciferin: enterobactin, Nutrition Reviews 31(7) (1973) 218-220. | Excluded | Title and abstract | Irrelevant |
| 1143] Nct, Sarcopenia Improves the Muscle Mass and Muscle Strength of Patients With Liver Cirrhosis-Child C, https://clinicaltrials.gov/show/NCT03633279 (2018). | Excluded | Title and abstract | Irrelevant |
| 1144] G. Stephen, Screening for iron deficiency in frequent blood donors, American Family Physician 80(5) (2009) 441-442. | Excluded | Title and abstract | Irrelevant |
| 1145] S. Mondello, F.H. Kobeissy, Y. Mechref, J. Zhao, S. El Hayek, K. Zibara, M. Moresco, G. Plazzi, F.I.I. Cosentino, R. Ferri, Searching for Novel Candidate Biomarkers of RLS in Blood by Proteomic Analysis, Nature and science of sleep 13 (2021) 873-883. | Excluded | Title and abstract | Irrelevant |
| 1146] P. Bulbovas, M.C.S. Rinaldi, W.B.C. Delitt, M. Domingos, Seasonal variation in antioxidants in leaves of young plants of <i>Caesalpinia echinata</i> Lam. (brazilwood), Revista Brasileira de Botanica 28(4) (2005) 687-696. | Excluded | Title and abstract | Irrelevant |
| 1147] E. Yancar Demir, L. Sütçigil, Secondary delusional parasitosis: Reviewing on a case report, Anatolian Journal of Clinical Investigation 7(3) (2013) 171-175. | Excluded | Title and abstract | Irrelevant |
| 1148] L. Robertson, S.E. Yeoh, A. Ramli, Secondary prevention of recurrent venous thromboembolism after initial oral anticoagulation therapy in patients with unprovoked venous thromboembolism, Cochrane Database of Systematic Reviews (12) (2017). | Excluded | Title and abstract | Irrelevant |
| 1149] Y. Shen, C.J. Mao, C.F. Liu, Secondary restless legs syndrome, Chinese Journal of Contemporary Neurology and Neurosurgery 13(5) (2013) 392-397. | Excluded | Title and abstract | Irrelevant |
| 1150] J. Boateng, M. Verghese, L. Shackelford, L.T. Walker, J. Khatiwada, S. Ogutu, D.S. Williams, J. Jones, M. Guyton, D. Asiamah, F. Henderson, L. Grant, M. DeBruce, A. Johnson, S. Washington, C.B. Chawan, Selected fruits reduce azoxymethane (AOM)-induced aberrant crypt foci (ACF) in Fisher 344 male rats, Food and Chemical Toxicology 45(5) (2007) 725-732. | Excluded | Title and abstract | Irrelevant |
| 1151] M.K. Erman, Selected Sleep Disorders: Restless Legs Syndrome and Periodic Limb Movement Disorder, Sleep Apnea Syndrome, and Narcolepsy, Psychiatric Clinics of North America 29(4) (2006) 947-967. | Excluded | Title and abstract | Irrelevant |
| 1152] S.R. Ankireddy, J. Kim, Selective detection of dopamine in the presence of ascorbic acid via fluorescence quenching of InP/ZnS quantum dots, International journal of nanomedicine 10 Spec Iss(Spec Iss) (2015) 113-9. | Excluded | Title and abstract | Irrelevant |
| 1153] E. Vázquez-Moreno, R. Moreno-Santamaría, M. Altagracia-Martínez, J. Kravzov-Jinich, C. Ríos-Castañeda, Self-medication in the Mexican State of Veracruz, Journal of Pharmaceutical Finance, Economics and Policy 13(2) (2004) 45-63. | Excluded | Title and abstract | Irrelevant |
| 1154] F.E. Leon-Sarmiento, J.S. Leon-Ariza, D. Prada, D.S. Leon-Ariza, C.V. Rizzo-Sierra, Sensory aspects in myasthenia gravis: A translational approach, Journal of the neurological sciences 368 (2016) 379-388. | Excluded | Title and abstract | Irrelevant |
| 1155] T.P. Lam, B.K.W. Ng, F.W.P. Yu, E.K.L. Tsang, W.Y.W. Lee, F.T.F. Cheung, H.X. Chen, J.C.Y. Cheng, Serum 25(OH) vitamin d level is associated with treatment outcome of whole-body vibration (WBV) for osteopenia in girls with adolescent idiopathic scoliosis (AIS), Scoliosis 10 (2015). | Excluded | Title and abstract | Irrelevant |
| 1156] M.-L. Bird, K.D. Hill, I.K. Robertson, M.J. Ball, J. Pittaway, A.D. Williams, Serum 25(OH)D status, ankle strength and activity show seasonal variation in older adults: relevance for winter falls in higher latitudes, Age and ageing 42(2) (2013) 181-185. | Excluded | Title and abstract | Irrelevant |
| 1157] H. Balaban, K. Yıldız Ö, G. Çil, A. Şentürk İ, T. Erselcan, E. Bolayır, S. Topaktaş, Serum 25-hydroxyvitamin D levels in restless legs syndrome patients, Sleep medicine 13(7) (2012) 953-7 | Included | Full text | Relationship between vitamins and RLS |
| 1158] T. Barker, V.T. Henriksen, V.E. Rogers, R.H. Trawick, Serum cytokines and muscle strength after anterior cruciate ligament surgery are not modulated by high-doses of vitamins E (alpha- and gamma-tocopherol's) and C, Cytokine 74(2) (2015) 279‐286. | Excluded | Title and abstract | Irrelevant |
| 1159] K. Celik, M.A. Cikrikcioglu, G. Halac, E. Kilic, S. Ayhan, N. Ozaras, K. Yildiz, R.S. Yildiz, M. Zorlu, C. Karatoprak, M. Cakirca, M. Kiskac, Serum endocan levels in women with restless legs syndrome, Neuropsychiatr Dis Treat 11 (2015) 2919-25. | Excluded | Full text | Duplication |
| 1160] M. Morker, D. Gossett, T. McKnight, B. Patel, M. Patel, H. Attarian, Serum folic acid level and its relationship to gestational willis ekbom disease (Restless legs syndrome): A Pilot study, Journal of Reproductive Medicine 62(6) (2017) 593-597. | Included | Full text | Relationship between vitamins and RLS |
| 1161] O.J.G. Schiepers, M.P.J. van Boxtel, R.H.M. de Groot, J. Jolles, W.L.A.M. de Kort, D.W. Swinkels, F.J. Kok, P. Verhoef, J. Durga, Serum Iron Parameters, <i>HFE</i> C282Y Genotype, and Cognitive Performance in Older Adults: Results From the FACIT Study, Journals of Gerontology Series a-Biological Sciences and Medical Sciences 65(12) (2010) 1312-1321. | Excluded | Title and abstract | Irrelevant |
| 1162] M.A. Beydoun, A.A. Gamaldo, J.A. Canas, H.A. Beydoun, M.T. Shah, J.M. McNeely, A.B. Zonderman, Serum Nutritional Biomarkers and Their Associations with Sleep among US Adults in Recent National Surveys, PloS one 9(8) (2014). | Excluded | Title and abstract | Irrelevant |
| 1163] G. Halac, E. Kilic, M.A. Cikrikcioglu, K. Celik, A. Toprak-Erek, S. Keskin, I. Gultepe, R.S. Celik, N. Ozaras, A. Yildiz, S. Aydin, O. Akan, C. Karatoprak, Y. Sekin, T. Asil, Serum soluble lectin-like oxidized low-density lipoprotein receptor-1 levels in patients with restless legs syndrome, Bratisl Lek Listy 117(6) (2016) 316-20. | Included | Full text | Relationship between vitamins and RLS |
| 1164] S.M. Olama, M.K. Senna, M.M. Elarman, G. Elhawary, Serum vitamin D level and bone mineral density in premenopausal Egyptian women with fibromyalgia, Rheumatology International 33(1) (2013) 185-192. | Excluded | Title and abstract | Irrelevant |
| 1165] T.P. Lam, F.T.F. Cheung, Q.W.Y. Mak, F.W.P. Yu, K.M. Lee, B.K.W. Ng, L. Qin, J.C.Y. Cheng, Serum vitamin D level can affect the treatment outcome of whole-body vibration (WBV) for osteopenia in girls with adolescent idiopathic scoliosis (AIS), Journal of bone and mineral research 28 (2013). | Excluded | Title and abstract | Irrelevant |
| 1166] F.J. Jiménez-Jiménez, G. Amo, H. Alonso-Navarro, M. Calleja, M. Díez-Fairén, I. Álvarez-Fernández, P. Pastor, J.F. Plaza-Nieto, S. Navarro-Muñoz, L. Turpín-Fenoll, J. Millán-Pascual, M. Recio-Bermejo, R. García-Ruiz, E. García-Albea, J.A.G. Agúndez, E. García-Martín, Serum vitamin D, vitamin D receptor and binding protein genes polymorphisms in restless legs syndrome, Journal of neurology (2020). | Excluded | Title and abstract | Duplication |
| 1167] F.J. Jiménez-Jiménez, G. Amo, H. Alonso-Navarro, M. Calleja, M. Díez-Fairén, I. Álvarez-Fernández, P. Pastor, J.F. Plaza-Nieto, S. Navarro-Muñoz, L. Turpín-Fenoll, J. Millán-Pascual, M. Recio-Bermejo, R. García-Ruiz, E. García-Albea, J.A.G. Agúndez, E. García-Martín, Serum vitamin D, vitamin D receptor and binding protein genes polymorphisms in restless legs syndrome, Journal of neurology 268(4) (2021) 1461-1472. | Included | Full text | Relationship between vitamins and RLS |
| 1168] F.J. Jimenez-Jimenez, G. Amo, H. Alonso-Navarro, M. Calleja, M. Diez-Fairen, I. Alvarez, P. Pastor, J.F. Plaza-Nieto, S. Navarro-Munoz, L. Turpin-Fenoll, J. Millan-Pascual, M. Recio-Bermejo, R. Garcia-Ruiz, E. Garcia-Albea, J.A.G. Agundez, E. Garcia-Martin, Serum vitamin D, vitamin D receptor and binding protein genes polymorphisms in restless legs syndrome (vol 15, pg 531, 2020), Journal of neurology 268(4) (2021) 1473-1473. | Excluded | Title and abstract | Irrelevant |
| 1169] P.M. Musoke, P. Fergusson, Severe malnutrition and metabolic complications of HIV-infected children in the antiretroviral era: Clinical care and management in resource-limited settings, American Journal of Clinical Nutrition 94(6) (2011) 1716S-1720S. | Excluded | Title and abstract | Irrelevant |
| 1170] M. Wakai, Severe periodic limb movement disorder presented in chronic renal failure at the stage of pre-dialysis, Sleep and Biological Rhythms 6(1) (2008) 56-59. | Excluded | Title and abstract | Irrelevant |
| 1171] K.K. Gill, Y. Park, Severity of restless legs syndrome and sensory nerve dysfunction, Sleep 28 (2005) A264-A265. | Excluded | Title and abstract | Irrelevant |
| 1172] M. Tan, G. Bourjeily, Shaking up perspectives of restless legs syndrome in pregnancy, Journal of Clinical Sleep Medicine 13(7) (2017) 857-858. | Excluded | Title and abstract | Irrelevant |
| 1173] M. Muruganantham, A. Ganapathi, S. Amutha, G. Vengadesan, N. Selvaraj, Shoot regeneration from immature cotyledonary nodes in black gram <i>Vigna mungo</i> (L.) Hepper, Indian Journal of Biotechnology 4(4) (2005) 551-555. | Excluded | Title and abstract | Irrelevant |
| 1174] A.S. Walters, Simple Sleep-Related Movement Disorders of Childhood Including Benign Sleep Myoclonus of Infancy, Rhythmic Movement Disorder, and Childhood Restless Legs Syndrome and Periodic Limb Movements in Sleep, Sleep Medicine Clinics 2(3) (2007) 419-432. | Excluded | Title and abstract | Irrelevant |
| 1175] L.G. Young, R.P. Forshaw, G.C. Smith, Simplified diets based on barley for reproducing swine, Journal of animal science 37(4) (1973) 898-905. | Excluded | Title and abstract | Irrelevant |
| 1176] R. Almeida, M. Nora, Single anastomosis duodenal switch (SADI-S) versus Roux-en-y gastric bypass-defining a new gold standard in metabolic surgery dragons' den meets shark tank (proposals for randomized controlled trials), Obesity surgery 27(1) (2017) 248. | Excluded | Title and abstract | Irrelevant |
| 1177] T.D. Martin, M.S. Green, M.T. Whitehead, T.P. Scheett, M.J. Webster, G.M. Hudson, Six weeks of oral Echinacea purpurea supplementation does not enhance the production of serum erythropoietin or erythropoietic status in recreationally active males with above-average aerobic fitness, Applied physiology, nutrition & metabolism 44(7) (2019) 791‐795. | Excluded | Title and abstract | Irrelevant |
| 1178] G.B. Euctr, A six-month double-blind, randomized, placebo-controlled study investigating the safety and tolerability of deferiprone in participants with Friedreich's ataxia - Not applicable, https://trialsearch.who.int/Trial2.aspx?TrialID=EUCTR2007-003331-23-GB (2007). | Excluded | Title and abstract | Irrelevant |
| 1179] A. Chinoy, G.R. Vassallo, E.B. Wright, J. Eelloo, S. West, E. Hupton, P. Galloway, A. Pilkington, R. Padidela, M.Z. Mughal, The skeletal muscle phenotype of children with Neurofibromatosis Type 1-A clinical perspective, Journal of Musculoskeletal & Neuronal Interactions 22(1) (2022) 70-78. | Excluded | Title and abstract | Irrelevant |
| 1180] S.I. Filipiuc, A.N. Neagu, C.M. Uritu, B.I. Tamba, L.E. Filipiuc, I.M. Tudorancea, A.N. Boca, M.F. Hâncu, V. Porumb, W. Bild, The Skin and Natural Cannabinoids–Topical and Transdermal Applications, Pharmaceuticals 16(7) (2023). | Excluded | Title and abstract | Irrelevant |
| 1181] R. Silvestri, Sleep and ADHD: A complex and bidirectional relationship, Sleep Medicine Reviews 63 (2022). | Excluded | Title and abstract | Irrelevant |
| 1182] R. Doherty, S. Madigan, G. Warrington, J. Ellis, Sleep and nutrition interactions: Implications for athletes, Nutrients 11(4) (2019). | Excluded | Title and abstract | Irrelevant |
| 1183] T.C. Wetter, H. Brunner, V. Collado-Seidel, C. Trenkwalder, J. Winkelmann, Sleep and periodic limb movements in corticobasal degeneration, Sleep medicine 3(1) (2002) 33-36. | Excluded | Title and abstract | Irrelevant |
| 1184] S. Allen, Sleep and sleeping disorders, Pharmaceutical Journal 274(7336) (2005) 187-190. | Excluded | Title and abstract | Irrelevant |
| 1185] J. Hanson, Sleep apnea in children and adolescents pediatrics: How sleep disorders affect children, Journal of Clinical Sleep Medicine 7(3) (2011) 31-33. | Excluded | Title and abstract | Irrelevant |
| 1186] J. Kanclerska, M. Wieckiewicz, D. Nowacki, A. Szymanska-Chabowska, R. Poreba, G. Mazur, H. Martynowicz, Sleep architecture and vitamin D in hypertensives with obstructive sleep apnea: A polysomnographic study, Dental and medical problems (2023). | Excluded | Title and abstract | Irrelevant |
| 1187] W.C. Chen, P.S. Lim, W.C. Wu, H.C. Chiu, C.H. Chen, H.Y. Kuo, T.W. Tsai, P.I. Chien, Y.J. Su, Y.L. Su, S.H. Hung, H.F. Woods, Sleep behavior disorders in a large cohort of chinese (Taiwanese) patients maintained by long-term hemodialysis, American journal of kidney diseases : the official journal of the National Kidney Foundation 48(2) (2006) 277-84. | Excluded | Title and abstract | Irrelevant |
| 1188] H. Kondo, K. Tanio, Y. Nagaura, M. Nagayoshi, C. Mitoma, M. Furue, T. Maeda, Sleep disorders among Yusho patients highly intoxicated with dioxin-related compounds: A 140-case series, Environmental Research 166 (2018) 261-268. | Excluded | Title and abstract | Irrelevant |
| 1189] D.S. Lewin, M. Di Pinto, Sleep disorders and ADHD: Shared and common phenotypes, Sleep 27(2) (2004) 188-189. | Excluded | Title and abstract | Irrelevant |
| 1190] B.A. Phillips, N.A. Collop, C. Drake, F. Consens, A.N. Vgontzas, T.E. Weaver, Sleep disorders and medical conditions in women. Proceedings of the women & sleep workshop, national sleep foundation, Washington, DC, March 5-6, 2007, Journal of Women's Health 17(7) (2008) 1191-1199. | Excluded | Title and abstract | Irrelevant |
| 1191] M. Wallner, Sleep disorders in dialysis patients, Nieren- und Hochdruckkrankheiten 38(5) (2009) 212-217. | Excluded | Title and abstract | Irrelevant |
| 1192] V. Wooten, Sleep disorders in geriatric patients, Clinics in geriatric medicine 8(2) (1992) 427-439. | Excluded | Title and abstract | Irrelevant |
| 1193] N. Kadıoğlu, U.Y. Sert, S.G. Sariaslan, K. Mursel, S. Celen, Sleep Disorders in Pregnancy, Influencing Factors and Quality of Life, Zeitschrift fur Geburtshilfe und Neonatologie 226(1) (2022) 34-40. | Excluded | Title and abstract | Irrelevant |
| 1194] R. Asplund, Sleep disorders in the elderly, Drugs and Aging 14(2) (1999) 91-103. | Excluded | Title and abstract | Irrelevant |
| 1195] P. Daubian-Nosé, M.K. Frank, A.M. Esteues, Sleep disorders: A review of the interface between restless legs syndrome and iron metabolism, Sleep Science 7(4) (2014) 234-237. | Excluded | Title and abstract | Irrelevant |
| 1196] S. Roychowdhury, D.R. Forsyth, Sleep disturbance in Parkinson disease, Journal of Clinical Gerontology and Geriatrics 3(2) (2012) 53-61. | Excluded | Title and abstract | Irrelevant |
| 1197] T.M. Ward, D.W. Beebe, M.L. Chen, C.A. Landis, S. Ringold, K. Pike, C.A. Wallace, Sleep disturbances and neurobehavioral performance in juvenile idiopathic arthritis, Journal of Rheumatology 44(3) (2017) 361-367. | Excluded | Title and abstract | Irrelevant |
| 1198] P. Polo-Kantola, Sleep disturbances in pregnancy: Why and how should we manage them?, Acta Obstetricia et Gynecologica Scandinavica 101(3) (2022) 270-272. | Excluded | Title and abstract | Irrelevant |
| 1199] H. Hachul de Campos, L.C. Brandão, V. D'Almeida, B.H. Grego, L.R. Bittencourt, S. Tufik, E.C. Baracat, Sleep disturbances, oxidative stress and cardiovascular risk parameters in postmenopausal women complaining of insomnia, Climacteric 9(4) (2006) 312-9. | Excluded | Title and abstract | Irrelevant |
| 1200] J.M. Meers, S. Nowakowski, Sleep During Pregnancy, Current Psychiatry Reports 24(8) (2022) 353-357. | Excluded | Title and abstract | Irrelevant |
| 1201] S. Javaheri, Sleep dysfunction in heart failure, Current Treatment Options in Neurology 10(5) (2008) 323-335. | Excluded | Title and abstract | Irrelevant |
| 1202] K.A. Lee, Sleep dysfunction in women and its management, Current Treatment Options in Neurology 8(5) (2006) 376-386. | Excluded | Title and abstract | Irrelevant |
| 1203] M. Pavlova, L.S. Sheikh, Sleep in women, Seminars in neurology 31(4) (2011) 397-403. | Excluded | Title and abstract | Irrelevant |
| 1204] C. Veyssier-Belot, Sleep Odyssey, Punta del Este, Uruguay, October 2001, Revue de Medecine Interne 23(4) (2002) 351-354. | Excluded | Title and abstract | Irrelevant |
| 1205] T. Cetin, S. Yetkin, F. Ozgen, Sleep pattern in patients with β-thalassemia minor, Blood 108(11) (2006) 31B-31B. | Excluded | Title and abstract | Irrelevant |
| 1206] A. Posar, P. Visconti, Sleep problems in children with autism spectrum disorder, Pediatric Annals 49(6) (2020) e278-e282. | Excluded | Title and abstract | Irrelevant |
| 1207] N. Parikh, T. Roth, Sleep related eating disorder and situational stress, Sleep 36 (2013) A441. | Excluded | Title and abstract | Irrelevant |
| 1208] I. dos Reis Santos, A. Roberta Danaga, L. Macario Ferraz, J. Julioti Urbano, N. Teixeira Fonsêca, V. Fernandes, V.A. Thomaz Fernandes, V.C. Delgado Lopes, F.S.S. Leitão Filho, S. Roberto Nacif, A.K. Fachini Araujo, L.V. Franco Oliveira, Sleep, depression, and quality of life in end-stage renal disease patients undergoing hemodialysis: A case report, Clinical and Experimental Medical Letters 54(1) (2013) 141-146. | Excluded | Title and abstract | Irrelevant |
| 1209] D. Esposito, A. Belli, R. Ferri, O. Bruni, Sleeping without Prescription: Management of Sleep Disorders in Children with Autism with Non-Pharmacological Interventions and Over-the-Counter Treatments, Brain Sciences 10(7) (2020). | Excluded | Title and abstract | Irrelevant |
| 1210] T.M. Brown, Sleep-Related Leg Cramps: A Review and Suggestions for Future Research, Sleep Medicine Clinics 10(3) (2015) 385-392. | Excluded | Title and abstract | Irrelevant |
| 1211] T. Freedom, Sleep-Related Movement Disorders, Disease-a-Month 57(8) (2011) 438-447. | Excluded | Title and abstract | Irrelevant |
| 1212] G. Merlino, G.L. Gigli, Sleep-related movement disorders, Neurological Sciences 33(3) (2012) 491-513. | Excluded | Title and abstract | Irrelevant |
| 1213] M.H. Silber, Sleep-related movement disorders, CONTINUUM Lifelong Learning in Neurology 19(1) (2013) 170-184. | Excluded | Title and abstract | Irrelevant |
| 1214] S. Huda, M.A. Alam, P.K. Sharma, Smart nanocarriers-based drug delivery for cancer therapy: An innovative and developing strategy, Journal of Drug Delivery Science and Technology 60 (2020). | Excluded | Title and abstract | Irrelevant |
| 1215] C. Guerra-Galicia, J. Blanco-Galina, Social Network for the diagnosis of RLS, Movement Disorder 34 (2019) S237-S238. | Excluded | Title and abstract | Irrelevant |
| 1216] Some complementary therapies have possible effects on the motor symptoms of Parkinsons disease, Drugs and Therapy Perspectives 26(7) (2010) 10-13. | Excluded | Title and abstract | Irrelevant |
| 1217] I.A. Silver, Some factors affecting wound healing, Equine veterinary journal 5(2) (1973) 47-51. | Excluded | Title and abstract | Irrelevant |
| 1218] Nct, Soy Modulation of Immune Activation, LDL- Levels, and Lowering Inflammation by Pretzel Isoflavone Dietary Intervention, https://clinicaltrials.gov/show/NCT02818283 (2016). | Excluded | Title and abstract | Irrelevant |
| 1219] S. Andree, C. Reble, J. Helfmann, Spectral in vivo signature of carotenoids in visible light diffuse reflectance from skin in comparison to ex vivo absorption spectra, Photonics and Lasers in Medicine 2(4) (2013) 323-335. | Excluded | Title and abstract | Irrelevant |
| 1220] J.-n. Lu, J.-s. Li, G.-x. Huang, L.-j. Yan, J. Ma, Spectroscopic Analysis on the Interaction of Chrysene With Herring Sperm DNA and Its Influence Factors, Spectroscopy and Spectral Analysis 42(1) (2022) 210-214. | Excluded | Title and abstract | Irrelevant |
| 1221] N. Swain, P. Padhan, S. Patnaik, Spectrum of rheumatological disorders in children from a tertiary care hospital in Bhubaneswar, Odisha, Indian Journal of Public Health Research and Development 8(4) (2017) 246-252. | Excluded | Title and abstract | Irrelevant |
| 1222] Y. Ma, X. She, S. Yang, Sphingosine-1-phosphate (S1P) mediates darkness-induced stomatal closure through raising cytosol pH and hydrogen peroxide (H<sub>2</sub>O<sub>2</sub>) levels in guard cells in <i>Vicia faba</i>, Science China-Life Sciences 55(11) (2012) 974-983. | Excluded | Title and abstract | Irrelevant |
| 1223] Nct, SR34006 Compared to Placebo in Patients Who Have Completed 6 Months of Treatment for Symptomatic Pulmonary Embolism or Deep Vein Thrombosis, https://clinicaltrials.gov/show/NCT00071279 (2003). | Excluded | Title and abstract | Irrelevant |
| 1224] K.W. Pedersen, J. Hansen, J.B. Hasselstrøm, J.R. Jornil, Stability investigations of cytochrome P450 (CYP) enzymes immediately after death in a pig model support the applicability of postmortem hepatic CYP quantification, Pharmacology Research and Perspectives 9(5) (2021). | Excluded | Title and abstract | Irrelevant |
| 1225] V.V. Koblianskii, State of the eyes of operators of radiolocation stations and means of improving their visual work capacity, Voenno-meditsinskii zhurnal 4 (1972) 54-6. | Excluded | Title and abstract | Irrelevant |
| 1226] E. Ueta, K. Yoneda, T. Yamamoto, T. Osaki, Stomatitis-reducing effect of azeptin (azelastine hydrochloride) in concomitant chemo-radiotherapy, Journal of Japan Society for Cancer Therapy 29(6) (1994) 919-924. | Excluded | Title and abstract | Irrelevant |
| 1227] Nct, Strategies to Increase Antenatal Iron and Folic Acid Supplementation and Malaria Prophylaxis, https://clinicaltrials.gov/show/NCT04250428 (2020). | Excluded | Title and abstract | Irrelevant |
| 1228] L.A. Hershey, M.A. Karan, Strong bones and restless legs: New data about bone remodeling in women with RLS, Neurology 86(13) (2016) 1179-1180. | Excluded | Full text | No relevant outcome |
| 1229] Umin, Studies on effects of addition of medium-chain triglyceride (MCT) or long-chain triglyceride (LCT) to an amino acids- enriched supplement on muscle and cognitive function in elderly subjects, https://trialsearch.who.int/Trial2.aspx?TrialID=JPRN-UMIN000017567 (2015). | Excluded | Title and abstract | Irrelevant |
| 1230] Umin, Studies on effects of medium-chain triglyceride (MCT) on physical function in elderly subjects, https://trialsearch.who.int/Trial2.aspx?TrialID=JPRN-UMIN000023302 (2016). | Excluded | Title and abstract | Irrelevant |
| 1231] Ctri, A study of a food supplement to reverse feeling of tiredness after a viral infection, https://trialsearch.who.int/Trial2.aspx?TrialID=CTRI/2023/08/056433 (2023). | Excluded | Title and abstract | Irrelevant |
| 1232] R.B.R. ddc, Study of different types of physical training in people living with HIV, https://trialsearch.who.int/Trial2.aspx?TrialID=RBR-8ddc92 (2019). | Excluded | Title and abstract | Irrelevant |
| 1233] Nct, Study of Nutritional Supplementation in Patients With Unilateral Wet AMD, https://clinicaltrials.gov/show/NCT04756310 (2021). | Excluded | Title and abstract | Irrelevant |
| 1234] Irct201105016352N, Study of the effect of Tactile-kinesthetic stimulations on neonatal behaviors and physical growth in LBW neonates, https://trialsearch.who.int/Trial2.aspx?TrialID=IRCT201105016352N1 (2012). | Excluded | Title and abstract | Irrelevant |
| 1235] F. Parrado, A. Buzzi, A study of the efficacy and tolerability of a preparation containing Ruscus aculeatus in the treatment of chronic venous insufficiency of the lower limbs, Clinical drug investigation 18(4) (1999) 255‐261. | Excluded | Title and abstract | Irrelevant |
| 1236] Nct, Study of Vitamin C, Vitamin E and Their Combination to Treat Restless Legs Syndrome in Hemodialysis Patients, https://clinicaltrials.gov/show/NCT01125033 (2010). | Excluded | Full text | Duplication |
| 1237] S. Das, N. Chatterjee, A. Mandal, P.K. Datta, A Study on Restless Legs Syndrome in Patients with Chronic Kidney Disease in a Tertiary Care Hospital, Journal of the Indian Medical Association 121(12) (2023) 37-40. | Included | Full text | Relationship between vitamins and RLS |
| 1238] Z. Chen, T. Song, X. Chen, S. Wang, J. Chen, Study on the interaction between hematoporphyrin monomethyl ether and DNA and the determination of hematoporphyrin monomethyl ether using the resonance light scattering technique, Spectrochimica Acta Part a-Molecular and Biomolecular Spectroscopy 77(3) (2010) 605-611. | Excluded | Title and abstract | Irrelevant |
| 1239] S.E. Euctr, A Study to Assess the efficacy and the safety of Vamorolone in Boys with Duchenne Muscular Dystrophy (DMD), https://trialsearch.who.int/Trial2.aspx?TrialID=EUCTR2017-002704-27-SE (2018). | Excluded | Title and abstract | Irrelevant |
| 1240] C.Z. Euctr, Study to evaluate relapse prevention in subjects with dementia-related psychosis treated with pimavanserin compared to placebo, (2017). | Excluded | Title and abstract | Irrelevant |
| 1241] C. Costa, S. Scabini, A. Kaimal, W. Kasozi, J. Cusato, O. Mbabazi, J.B. Kafufu, E.S. Mwaka, G. Di Perri, M. Lamorde, A. Calcagno, B. Castelnuovo, Subclinical tubular impairment is common in art-treated HIV+ patients in Uganda, Topics in Antiviral Medicine 27(SUPPL 1) (2019) 265s-266s. | Excluded | Title and abstract | Irrelevant |
| 1242] P.M. Newberne, The subcommissural organ of the vitamin B12-deficient rat, The Journal of nutrition 76 (1962) 393-413. | Excluded | Title and abstract | Irrelevant |
| 1243] Nct, Substrate Cycling in Energy Metabolism, https://clinicaltrials.gov/show/NCT00361751 (2006). | Excluded | Title and abstract | Irrelevant |
| 1244] M.C. Schaeffer, E.F. Cochary, J.A. Sadowski, Subtle abnormalities of gait detected early in vitamin B6 deficiency in aged and weanling rats with hind leg gait analysis, Journal of the American College of Nutrition 9(2) (1990) 120-7. | Excluded | Title and abstract | Irrelevant |
| 1245] J.S. Rotenberg, K. Canard, M. DiFazio, Successful treatment of recalcitrant restless legs syndrome with botulinum toxin type-A, Journal of Clinical Sleep Medicine 2(3) (2006) 275-278. | Excluded | Title and abstract | Irrelevant |
| 1246] D. Kemlink, K. Šonka, M. Pretl, H. Benáková, T. Zima, S. Nevšímalová, Suggestive evidence of erythropoietin level abnormality in patients with sporadic and familial cases of the restless leqs syndrome, Neuroendocrinology Letters 28(5) (2007) 643-646. | Included | Full text | Relationship between vitamins and RLS |
| 1247] T. Sakai, T. Matsuishi, S. Yamada, H. Komori, H. Iwashita, Sulfamethoxazole-trimethoprim double-blind, placebo-controlled, crossover trial in Machado-Joseph disease: sulfamethoxazole-trimethoprim increases cerebrospinal fluid level of biopterin, Journal of neural transmission. General section 102(2) (1995) 159‐172. | Excluded | Title and abstract | Irrelevant |
| 1248] A. Tomillero, M.A. Moral, Summary, Methods and Findings in Experimental and Clinical Pharmacology 32(4) (2010) 247-288. | Excluded | Title and abstract | Irrelevant |
| 1249] M. Taj, SUN-245 ASSOCIATION OF RESTLESS LEG SYNDROME AND CHRONIC KIDNEY DISEASE- MINERAL AND BONE DISORDER IN HEMODIALYSIS PATIENTS, Kidney International Reports 5(3) (2020) S300. Excluded Title and abstract Irrelevant | Excluded | Title and abstract | Irrelevant |
| 1250] Isrctn, Sunlight exposure and vitamin D in the ageing population, https://trialsearch.who.int/Trial2.aspx?TrialID=ISRCTN14201277 (2016). | Excluded | Title and abstract | Irrelevant |
| 1251] Nct, Supplement Study: strength, Testosterone, Sexual Function, Quality of Life, https://clinicaltrials.gov/show/NCT03457740 (2018). | Excluded | Title and abstract | Irrelevant |
| 1252] A. Chanet, S. Verlaan, J. Salles, C. Giraudet, V. Patrac, V. Pidou, C. Pouyet, N. Hafnaoui, A. Blot, N. Cano, et al., Supplementing Breakfast with a Vitamin D and Leucine-Enriched Whey Protein Medical Nutrition Drink Enhances Postprandial Muscle Protein Synthesis and Muscle Mass in Healthy Older Men, Journal of nutrition 147(12) (2017) 2262‐2271. E | Excluded | Title and abstract | Irrelevant |
| 1253] G. Siska, Supplements Can Help Maintain Ideal Levels of Neurochemicals, 2020. | Excluded | Title and abstract | Irrelevant |
| 1254] X. Wei, L. Yang, X. Tang, Survey on restless legs syndrome in pregnant Chinese women, Sleep and Biological Rhythms 11(4) (2013) 286-287. | Excluded | Title and abstract | Irrelevant |
| 1255] Y.R. Bang, H.J. Jeon, H.Y. Park, I.Y. Yoon, Symptom persistence after iron normalization in women with restless legs syndrome, Psychiatry Investigation 15(4) (2018) 390-395. | Excluded | Title and abstract | Irrelevant |
| 1256] S. Das, Syndromes of excessive daytime sleepiness (EDS), Sleep and Vigilance 1(2) (2017) 126. | Excluded | Title and abstract | Irrelevant |
| 1257] R.A. Steenblik, M.J. Hurt, G.R. Jordan, R. Steenblik, M. Hurt, G. Jordan, S.M. Cape, A.S. Richard, J.H. Mark, R.J. Gregory, Synthetic magnification micro-optic system for passport, currency, has periodic planar array of image icon focusing elements such as non-cylindrical lenses having effective diameter in specific range, Nanoventions Inc; Steenblik R a; Hurt M J; Jordan G R; Nanoventions Inc Q; Nanoventions Holdings Llc; Visual Physics Llc. | Excluded | Title and abstract | Irrelevant |
| 1258] F.H. Burbank, M.L. Jones, A. Memmolo, System for generating counter-stimulation in patient, has controller driving vibration generator and terminating vibrations after period of vibration, and base holding generator adjacent to patient, where generator is attached to base, Sensory Neurostimulation Inc. | Excluded | Title and abstract | Irrelevant |
| 1259] G.H. Neild, R. Jha, D. Gude, S. Mandal, R. Batta, Tackling the 'brown' frown, Clinical kidney journal 5(2) (2012) 176-177. | Excluded | Title and abstract | Irrelevant |
| 1260] G.D. Weinstein, G.G. Krueger, N.J. Lowe, M. Duvic, D.J. Friedman, B.V. Jegasothy, J.L. Jorizzo, E. Shmunes, E.H. Tschen, D.A. Lew-Kaya, et al., Tazarotene gel, a new retinoid, for topical therapy of psoriasis: vehicle-controlled study of safety, efficacy, and duration of therapeutic effect, Journal of the American Academy of Dermatology 37(1) (1997) 85‐92. | Excluded | Title and abstract | Irrelevant |
| 1261] K.R. Rollakanti, S.C. Kanick, S.C. Davis, B.W. Pogue, E.V. Maytin, Techniques for fluorescence detection of protoporphyrin IX in skin cancers associated with photodynamic therapy, Photonics and Lasers in Medicine 2(4) (2013) 287-303. | Excluded | Title and abstract | Irrelevant |
| 1262] B. Otto, I. Ohad, K. Kloppstech, Temperature treatments of dark-grown pea seedlings cause an accelerated greening in the light at different levels of gene expression, Plant molecular biology 18(5) (1992) 887-96. | Excluded | Title and abstract | Irrelevant |
| 1263] Actrn, Testosterone supplementation combined with exercise program to improve muscle condition in men affected by inclusion body myositis, http://www.who.int/trialsearch/Trial2.aspx?TrialID=ACTRN12618000755235 (2018). | Excluded | Title and abstract | Irrelevant |
| 1264] A. Jadidi, A. Rezaei Ashtiani, A. Khanmohamadi Hezaveh, S.M. Aghaepour, Therapeutic effects of magnesium and vitamin B6 in alleviating the symptoms of restless legs syndrome: a randomized controlled clinical trial, BMC complementary medicine and therapies 23(1) (2022) 1. | Included | Full text | Vetamin therapy for RLS |
| 1265] A. Jadidi, A. Rezaei Ashtiani, A. Khanmohamadi Hezaveh, S.M. Aghaepour, Therapeutic effects of magnesium and vitamin B6 in alleviating the symptoms of restless legs syndrome: a randomized controlled clinical trial, BMC complementary medicine & therapies 23(1) (2023) 1‐6. | Excluded | Full text | Duplication |
| 1266] X.J. Fang, Therapeutic observation of acupoint thread embedding plus ultrashort waves for sleep disorders due to restless legs syndrome, Shanghai journal of acupuncture and moxibustion  shang hai zheng jiu za zhi] 35(1) (2016) 25‐26. 1267] Nct, Thiamine As An Adjuvant Therapy For Hyperlactatemia In Septic Shock Patients, https://clinicaltrials.gov/show/NCT03649009 (2018). | Excluded | Title and abstract | Irrelevant |
| 1268] A.M. Reisman, B.T. Robbins, D.E. Chou, M.S. Yugrakh, G.J. Gross, L. Privitera, T. Nazif, R.J. Sommer, Ticagrelor for refractory migraine/patent foramen ovale (TRACTOR): An open-label pilot study, Neurology 91(22) (2018) 1010-1017. | Excluded | Title and abstract | Irrelevant |
| 1269] L.J. Lubaki, G. Ghanem, P. Vereecken, E. Fouty, L. Benammar, J. Vadoud-Seyedi, M.L. Dell'Anna, S. Briganti, M. Picardo, M. Heenen, Time-kinetic study of repigmentation in vitiligo patients by tacrolimus or pimecrolimus, Archives of dermatological research 302(2) (2010) 131‐137. | Excluded | Title and abstract | Irrelevant |
| 1270] M. Skulinova, C. Lefebvre, P. Sobron, E. Eshelman, M. Daly, J.F. Gravel, J.F. Cormier, F. Chateauneuf, G. Slater, W. Zheng, A. Koujelev, R. Leveille, Time-resolved stand-off UV-Raman spectroscopy for planetary exploration, Planetary and Space Science 92 (2014) 88-100. | Excluded | Title and abstract | Irrelevant |
| 1271] V. Di Lazzaro, F. Pilato, A.P. Batocchi, D. Restuccia, G. Cammarota, P. Profice, Tired legs-a gut diagnosis, The Lancet 376(9754) (2010) 1798. | Excluded | Title and abstract | Irrelevant |
| 1272] Nct, To Study the Nutri-Genomic Response of Vit-D Supplementation in African-Americans, https://clinicaltrials.gov/show/NCT02802449 (2016). | Excluded | Title and abstract | Irrelevant |
| 1273] M.B. Oliveira, A.H. do Prado, J. Bernegossi, C.S. Sato, I.L. Brunetti, M.V. Scarpa, G.R. Leonardi, S.E. Friberg, M. Chorilli, Topical Application of Retinyl Palmitate-Loaded Nanotechnology-Based Drug Delivery Systems for the Treatment of Skin Aging, BioMed research international 2014 (2014). | Excluded | Title and abstract | Irrelevant |
| 1274] G.B. Euctr, Topical Retinoids and Diabetic Neuropathic Ulceration - Retinoid Study, https://trialsearch.who.int/Trial2.aspx?TrialID=EUCTR2006-005597-43-GB (2007). | Excluded | Title and abstract | Irrelevant |
| 1275] A.L. Olsen, J.J. Locascio, I. Tuncali, N. Laroussi, E. Abatzis, P. Kamenskaya, Y. Kuras, T. Yi, A. Videnovic, M.T. Hayes, G.P.H. Ho, J. Paulson, V. Khurana, T.M. Herrington, B.T. Hyman, D.J. Selkoe, J.H. Growdon, S.N. Gomperts, T. Riise, M.A. Schwarzschild, A.Y. Hung, A.M. Wills, C.R. Scherzer, Towards a phenome-wide view of Parkinson's disease, 2022. | Excluded | Title and abstract | Irrelevant |
| 1276] A. Olsen, J. Locascio, C. Scherzer, Towards defining the Parkinson's health-ome, Neurology 96(15) (2021). | Excluded | Title and abstract | Irrelevant |
| 1277] X. Yan, W.D. Wang, A.S. Walters, Q. Wang, Y.J. Liu, F.Y. Chu, Traditional Chinese medicine herbal preparations in restless legs syndrome (RLS) treatment: A review and probable first description of RLS in 1529, Sleep Medicine Reviews 16(6) (2012) 509-518. | Excluded | Title and abstract | Irrelevant |
| 1278] Nct, Training Intervention in a Controlled Population of Frail Elderly, https://clinicaltrials.gov/show/NCT02331459 (2015). | Excluded | Title and abstract | Irrelevant |
| 1279] W. Cawello, M. Emgenbroich, K. Kassner, A. Lappert, J.J. Leonhard, W. Mueller, H. Wolff, J. Leonhard, W. Muller, H.M. Wolff, H. Wolff, Transdermal therapeutic system useful for treating or preventing patients suffering from e.g. Parkinson's disease and cognitive disorders, contains backing layer, solvent-based self-adhesive matrix layer and release liner, Ucb Pharma Gmbh; Lts Lohmann Therapie-Systeme Ag; Lts Lohmann Therapie-Systeme Gmbh & Co; Ucb Pharm Co Ltd; Lts Lohmann Therapie Systeme Ag; Cawello W; Lappert a; Kassner K; Wolff H; Mueller W; Leonhard J J; Emgenbroich M; Ucb Biopharma Sprl. | Excluded | Title and abstract | Irrelevant |
| 1280] L. Priano, M.R. Gasco, A. Mauro, Transdermal treatment options for neurological disorders - Impact on the elderly, Drugs & aging 23(5) (2006) 357-375. | Excluded | Title and abstract | Irrelevant |
| 1281] J.C. Pereira, I.R. Rocha e Silva, M. Pradella-Hallinan, Transient Willis-Ekbom's disease (restless legs syndrome) during pregnancy may be caused by estradiol-mediated dopamine overmodulation, Medical hypotheses 80(2) (2013) 205-208. | Excluded | Title and abstract | Irrelevant |
| 1282] J.C. He, J.Y. Zheng, X. Li, Y. Yang, B.Y. Zhang, Y. Chen, X.F. Li, Y.M. Liu, Y. Cao, L. Zhao, T.C. Li, Transthoracic contrast echocardiography using vitamin B6 and sodium bicarbonate as contrast agents for the diagnosis of patent foramen ovale, The international journal of cardiovascular imaging 33(8) (2017) 1125-1131. | Excluded | Title and abstract | Irrelevant |
| 1283] S. Nakase, K. Yamazaki, E. Motomura, Trazodone advanced a delayed sleep phase of an elderly male: A case report, Sleep and Biological Rhythms 3(3) (2005) 169-171. | Excluded | Title and abstract | Irrelevant |
| 1284] B. Cocks, P. Hobman, M. McDonagh, A. Tester, A. Brown, M. Rowney, Treating a disorder characterized by elevated or dysregulated myostatin comprises administering an amount of angiogenin or an angiogenin agonist, Agric Victoria Services Pty Ltd; Murray Goulburn Coop Co Ltd; Mcdonagh M; Cocks B; Tester a; Hobman P; Saputo Dairy Australia Pty Ltd. | Excluded | Title and abstract | Irrelevant |
| 1285] B. Cocks, P. Hobman, M. McDonagh, A. Tester, Treating a disorder comprises orally administering angiogenin, Agric Victoria Services Pty Ltd; Murray Goulburn Coop Co Ltd. | Excluded | Title and abstract | Irrelevant |
| 1286] A. Gupta, G. Ajay, Treating iron deficiency that reduces/eliminates the dose of erythropoiesis stimulating agent to achieve/maintain target hemoglobin levels in subject suffering from anemia, comprises administering soluble ferric pyrophosphate composition, Charak Llc; Gupta A. | Excluded | Title and abstract | Irrelevant |
| 1287] R.J. Leo, Treatment considerations in neuropathic pain, Current Treatment Options in Neurology 8(5) (2006) 389-400. | Excluded | Title and abstract | Irrelevant |
| 1288] G. Meola, V. Sansone, Treatment in myotonia and periodic paralysis, Revue neurologique 160(5) (2004) S55-S69. | Excluded | Title and abstract | Irrelevant |
| 1289] P. Vogel, Treatment in restless legs syndrome, Aktuelle Neurologie 11(4) (1984) 111. | Excluded | Title and abstract | Irrelevant |
| 1290] A. Diamond, J. Jankovic, Treatment of advanced Parkinson's disease, Expert review of neurotherapeutics 6(8) (2006) 1181-1197. | Excluded | Title and abstract | Irrelevant |
| 1291] J. Finsterer, Treatment of central nervous system manifestations in mitochondrial disorders, European journal of neurology 18(1) (2011) 28-38. | Excluded | Title and abstract | Irrelevant |
| 1292] B. Yilbaş, Treatment of Depression with Vortioxetine in a Patient with Comorbid Major Depressive Disorder and Restless Legs Syndrome: A Case Report, Noropsikiyatri Arsivi 59(2) (2022) 164-166. | Excluded | Title and abstract | Irrelevant |
| 1293] G. Kirkilesis, S.K. Kakkos, C. Bicknell, S. Salim, K. Kakavia, Treatment of distal deep vein thrombosis, Cochrane Database of Systematic Reviews (4) (2020). | Excluded | Title and abstract | Irrelevant |
| 1294] A. Singh, R. Shah, R. Cholera, P. Mulky, Treatment of Ekbom Syndrome With Clozapine and Electroconvulsive Therapy, Cureus 14(10) (2022) e30469. | Excluded | Title and abstract | Irrelevant |
| 1295] P. Polo-Kantola, Treatment of menopausal sleep disorders, International Journal of Gynecology and Obstetrics 143 (2018) 136. | Excluded | Title and abstract | Irrelevant |
| 1296] T. Walton, K.W. Kolb, Treatment of nocturnal leg cramps and restless leg syndrome, Clinical pharmacy 10(6) (1991) 427-8. | Excluded | Title and abstract | Irrelevant |
| 1297] P.S. Connolly, E.A. Shirley, J.H. Wasson, D.W. Nierenberg, Treatment of nocturnal leg cramps. A crossover trial of quinine vs vitamin E, Archives of internal medicine 152(9) (1992) 1877‐1880. | Excluded | Title and abstract | Irrelevant |
| 1298] L. DelRosso, O. Bruni, Treatment of pediatric restless legs syndrome, 2019, pp. 237-253. | Excluded | Title and abstract | Irrelevant |
| 1299] B. Buyse, Treatment of periodic limb movements in sleep, Tijdschrift voor Geneeskunde 64(7) (2008) 376. | Excluded | Title and abstract | Irrelevant |
| 1300] D.K. Euctr, Treatment of pregnant women with iron deficiency, https://trialsearch.who.int/Trial2.aspx?TrialID=EUCTR2017-000776-29-DK (2017). | Excluded | Title and abstract | Irrelevant |
| 1301] Irct2014011216183N, treatment of Restless Leg Syndrome associated end stage renal disease, https://trialsearch.who.int/Trial2.aspx?TrialID=IRCT2014011216183N1 (2014). | Excluded | Title and abstract | Irrelevant |
| 1302] L. Ferini-Strambi, M. Manconi, Treatment of restless legs syndrome, Parkinsonism and Related Disorders 15(SUPPL. 4) (2010) S65-S70. | Excluded | Title and abstract | Irrelevant |
| 1303] P. Montagna, The treatment of restless legs syndrome, Neurological Sciences 28(SUPL.1) (2007) S61-S66. | Excluded | Title and abstract | Irrelevant |
| 1304] W. Hening, R. Allen, C. Earley, C. Kushida, D. Picchietti, M. Silber, The treatment of Restless Legs Syndrome and Periodic Limb Movement Disorder, Sleep 22(7) (1999) 970-999. | Excluded | Title and abstract | Irrelevant |
| 1305] C. Trenkwalder, W.A. Hening, P. Montagna, W.H. Oertel, R.P. Allen, A.S. Walters, J. Costa, K. Stiasny-Kolster, C. Sampaio, Treatment of restless legs syndrome: an evidence-based review and implications for clinical practice, Mov Disord 23(16) (2008) 2267-302. | Excluded | Title and abstract | Irrelevant |
| 1306] J. Winkelmann, R.P. Allen, B. Högl, Y. Inoue, W. Oertel, A.V. Salminen, J.W. Winkelman, C. Trenkwalder, C. Sampaio, Treatment of restless legs syndrome: Evidence-based review and implications for clinical practice (Revised 2017)§, Movement Disorders 33(7) (2018) 1077-1091. | Excluded | Title and abstract | Irrelevant |
| 1307] J. Montplaisir, O. Lapierre, H. Warnes, G. Pelletier, The treatment of the restless leg syndrome with or without periodic leg movements in sleep, Sleep 15(5) (1992) 391-395. | Excluded | Title and abstract | Irrelevant |
| 1308] C. Nagant de Deuxchaisnes, J.P. Devogelaer, G. Depresseux, J. Malghem, B. Maldague, Treatment of the vertebral crush fracture syndrome with enteric-coated sodium fluoride tablets and calcium supplements, Journal of bone and mineral research 5 Suppl 1 (1990) S5‐26. | Excluded | Title and abstract | Irrelevant |
| 1309] K. Ramakrishnan, D.C. Scheid, Treatment options for insomnia, South African Family Practice 49(8) (2007) 34-41. | Excluded | Title and abstract | Irrelevant |
| 1310] L. Ferini-Strambi, Treatment options for restless legs syndrome, Expert opinion on pharmacotherapy 10(4) (2009) 545-554. | Excluded | Title and abstract | Irrelevant |
| 1311] J.J. Chen, M.F. Lew, A. Siderowf, Treatment strategies and quality-of-care indicators for patients with Parkinson's disease, Journal of Managed Care Pharmacy 15(3 SUPPL.) (2009) S1-S21. | Excluded | Title and abstract | Irrelevant |
| 1312] A. O’Donnell, C. McParlin, S.C. Robson, F. Beyer, E. Moloney, A. Bryant, J. Bradley, C. Muirhead, C. Nelson-Piercy, D. Newbury-Birch, J. Norman, E. Simpson, B. Swallow, L. Yates, L. Vale, Treatments for hyperemesis gravidarum and nausea and vomiting in pregnancy: A systematic review and economic assessment, Health Technology Assessment 20(74) (2016) vii-268. | Excluded | Title and abstract | Irrelevant |
| 1313] Treatments for restless legs syndrome, The Western journal of medicine 145(4) (1986) 522-3. | Excluded | Title and abstract | Irrelevant |
| 1314] Nct, A Trial to Compare Efficacy and Tolerability of Plenvu® and Picoprep® as Cleansing Agents Before Colonoscopy, https://clinicaltrials.gov/show/NCT05436054 (2022). | Excluded | Title and abstract | Irrelevant |
| 1315] J. Arpa, I. Sanz-Gallego, F.J. Rodríguez-de-Rivera, F.J. Domínguez-Melcón, D. Prefasi, J. Oliva-Navarro, M. Moreno-Yangüela, Triple therapy with deferiprone, idebenone and riboflavin in Friedreich's ataxia - open-label trial, Acta neurologica Scandinavica 129(1) (2014) 32-40. | Excluded | Title and abstract | Irrelevant |
| 1316] B. Eyskens, W. Proesmans, B. Van Damme, L. Lateur, R. Bouillon, M. Hoogmartens, Tumour-induced rickets: a case report and review of the literature, European journal of pediatrics 154(6) (1995) 462-8. | Excluded | Title and abstract | Irrelevant |
| 1317] J. Liu, S. Liu, L. Hao, F. Liu, S. Mu, T. Wang, Uncovering the mechanism of Radix Paeoniae Alba in the treatment of restless legs syndrome based on network pharmacology and molecular docking, Medicine (United States) 101(46) (2022) E31791. | Excluded | Title and abstract | Irrelevant |
| 1318] W.S. Pray, G.E. Pray, Understanding and relieving restless legs syndrome, 2014, pp. 8-11. | Excluded | Title and abstract | Irrelevant |
| 1319] A.O. Rossetti, R. Heinzer, F. Espa, M. Tafti, Unilateral periodic leg movements during wakefulness and sleep after a parietal hemorrhage, Sleep medicine 9(4) (2008) 465-466. | Excluded | Title and abstract | Irrelevant |
| 1320] J. Giergielewicz, P. Kosciuczyk, I. Piwowarska, Unusual case of painful form of restless legs syndrome, Neurologia i neurochirurgia polska 7(5) (1973) 719-22. | Excluded | Title and abstract | Irrelevant |
| 1321] C.S. Tsai, Y.C. Chen, H.H. Chen, C.J. Cheng, S.H. Lin, An unusual cause of hypokalemic paralysis: Aristolochic acid nephropathy with Fanconi syndrome, American Journal of the Medical Sciences 330(3) (2005) 153-155. | Excluded | Title and abstract | Irrelevant |
| 1322] A. Kumar, Update in neuropsychiatry: Cognition, sleep and sleep-related disorders, Drug News and Perspectives 19(4) (2006) 243-247. | Excluded | Title and abstract | Irrelevant |
| 1323] P. Gonzalez-Latapi, R. Malkani, Update on Restless Legs Syndrome: from Mechanisms to Treatment, Current Neurology and Neuroscience Reports 19(8) (2019). | Excluded | Title and abstract | Irrelevant |
| 1324] B.F. Boeve, Update on the Diagnosis and Management of Sleep Disturbances in Dementia, Sleep Medicine Clinics 3(3) (2008) 347-360. | Excluded | Title and abstract | Irrelevant |
| 1325] U. Gröber, Updated and compact - Iron in restless legs syndrome, Deutsche Apotheker Zeitung 150(5) (2010) 104-107. | Excluded | Title and abstract | Irrelevant |
| 1326] D.C. Meyer, J. Singh, A.E. Jimenez, Uptake of serotonin and norepinephrine in hypothalamic and limbic brain regions during the estrous cycle and the effect of neurotoxin lesions on estrous cyclicity, Brain research bulletin 10(5) (1983) 639-45. | Excluded | Title and abstract | Irrelevant |
| 1327] L. Baccaglini, R.V. Lalla, A.J. Bruce, J.C. Sartori-Valinotti, M.C. Latortue, M. Carrozzo, R.S. Rogers, III, Urban legends: recurrent aphthous stomatitis, Oral Diseases 17(8) (2011) 755-770. | Excluded | Title and abstract | Irrelevant |
| 1328] J.L. Seifter, M.A. Samuels, Uremic encephalopathy and other brain disorders associated with renal failure, Seminars in neurology 31(2) (2011) 139-143. | Excluded | Title and abstract | Irrelevant |
| 1329] A. Bernardi, F. Biasia, P. Scapin, G. Bucciante, R. Dainese, F. Brazzo, R. Hanau, Uremic neuropathy: clinical evolution and therapeutic results in a 5-year period of observation, Giornale di clinica medica 63(1) (1982) 29-36. | Excluded | Title and abstract | Irrelevant |
| 1330] K. Gade, S. Blaschke, A. Rodenbeck, A. Becker, H. Anderson-Schmidt, S. Cohrs, Uremic restless legs syndrome (RLS) and sleep quality in patients with end-stage renal disease on hemodialysis: potential role of homocysteine and parathyroid hormone, Kidney & blood pressure research 37(4-5) (2013) 458-63. | Included | Full text | Relationship between vitamins and RLS |
| 1331] L.V. Packett, S.P. Coburn, URINE PROTEINS IN NUTRITIONALLY INDUCED OVINE UROLITHIASIS, American journal of veterinary research 26 (1965) 112-9. | Excluded | Title and abstract | Irrelevant |
| 1332] M. Fendt, D. Feuerbach, B. Gomez-Mancilla, C. Lopez-Lopez, K.H. McAllister, M.B. Gomez, L.C. Lopez, Use of alpha 7 nicotinic acetylcholine receptor agonist for the treatment, prevention or delay of progression of narcolepsy, excessive daytime sleepiness, sleep disruption or cataplexy, Novartis Ag. | Excluded | Title and abstract | Irrelevant |
| 1333] R. Cappelli, M. Nicora, T. Di Perri, Use of extract of Ruscus aculeatus in venous disease in the lower limbs, Drugs under experimental and clinical research 14(4) (1988) 277‐283. | Excluded | Title and abstract | Irrelevant |
| 1334] N. Cuellar, The use of medications, herbs, natural products and complementary and alternative practices and products (CAPPs) in persons with restless legs syndrome, Sleep 27 (2004) 318-318. | Excluded | Title and abstract | Irrelevant |
| 1335] B. Aranda, The use of vitamin B1 in medicaments, Aranda B. | Excluded | Title and abstract | Irrelevant |
| 1336] B. Kucukakin, J. Lykkesfeldt, H.J. Nielsen, R.J. Reiter, J. Rosenberg, I. Gogenur, Utility of melatonin to treat surgical stress after major vascular surgery - a safety study, Journal of pineal research 44(4) (2008) 426‐431. | Excluded | Title and abstract | Irrelevant |
| 1337] S. Sahu, M. Ghosh, D.K. Bhattacharyya, Utilization of unsaponifiable matter from rice bran oil fatty acid distillate for preparing an antioxidant-rich oleogel and evaluation of its properties, Grasas Y Aceites 71(1) (2020). | Excluded | Title and abstract | Irrelevant |
| 1338] A.C. Winter, K. Berger, R.J. Glynn, J.E. Buring, J.M. Gaziano, M. Schuerks, T. Kurth, Vascular Risk Factors, Cardiovascular Disease, and Restless Legs Syndrome in Men, American Journal of Medicine 126(3) (2013) 228-U166. | Excluded | Title and abstract | Irrelevant |
| 1339] Actrn, Vibration Training in Children with Cystic Fibrosis: function, Power, Bone, http://www.who.int/trialsearch/Trial2.aspx?TrialID=ACTRN12609000520235 (2009). | Excluded | Title and abstract | Irrelevant |
| 1340] J.M. Parish, Violent dreaming and antidepressant drugs: Or how paroxetine made me dream that i was fighting Saddam Hussein, Journal of Clinical Sleep Medicine 3(5) (2007) 529-531. | Excluded | Title and abstract | Irrelevant |
| 1341] Nct, Vitamin A and Very Low Birthweight Babies (VitAL), https://clinicaltrials.gov/show/NCT00417404 (2006). | Excluded | Title and abstract | Irrelevant |
| 1342] Nct, Vitamin B6, B12, Folic Acid and Exercise in Parkinson's Disease, https://clinicaltrials.gov/show/NCT01238926 (2010). | Excluded | Title and abstract | Irrelevant |
| 1343] L. Solomon, Vitamin B12 in the management of neuropathy: A retrospective study (S758), Journal of Pain and Symptom Management 45(2) (2013) 453-454. | Excluded | Full text | No relevant outcome |
| 1344] L.R. Solomon, Vitamin B12-responsive neuropathies: A case series, Nutritional Neuroscience 19(4) (2016) 162-168. | Excluded | Title and abstract | Irrelevant |
| 1345] M.J. Kretsch, H.E. Sauberlich, J.H. Skala, H.L. Johnson, Vitamin B-6 requirement and status assessment: young women fed a depletion diet followed by a plant- or animal-protein diet with graded amounts of vitamin B-6, American journal of clinical nutrition 61(5) (1995) 1091‐1101. | Excluded | Title and abstract | Irrelevant |
| 1346] Nct, Vitamin C 4 Care Homes, https://clinicaltrials.gov/show/NCT05122481 (2021). | Excluded | Title and abstract | Irrelevant |
| 1347] G. Paulsen, H. Hamarsland, K.T. Cumming, R.E. Johansen, J.J. Hulmi, E. Borsheim, H. Wiig, I. Garthe, T. Raastad, Vitamin C and E supplementation alters protein signalling after a strength training session, but not muscle growth during 10 weeks of training, Journal of physiology 592(24) (2014) 5391‐5408. | Excluded | Title and abstract | Irrelevant |
| 1348] Actrn, Vitamin D and Exercise to Improve Physical Function in Older Adults, http://www.who.int/trialsearch/Trial2.aspx?TrialID=ACTRN12616000563460 (2016). | Excluded | Title and abstract | Irrelevant |
| 1349] C.N. Homann, G. Ivanic, B. Homann, T.U. Purkart, Vitamin D and Hyperkinetic Movement Disorders: A Systematic Review, Tremor Other Hyperkinet Mov (N Y) 10 (2020) 32. | Excluded | Title and abstract | Irrelevant |
| 1350] P. López Méndez, M. Sosa Henríquez, Vitamin D and multiple sclerosis. Prevalence of hypovitaminosis D, Revista de Osteoporosis y Metabolismo Mineral 7(2) (2015) 71-78. | Excluded | Title and abstract | Irrelevant |
| 1351] D. Scott, P.R. Ebeling, K.M. Sanders, D. Aitken, T. Winzenberg, G. Jones, Vitamin D and physical activity status: associations with five-year changes in body composition and muscle function in community-dwelling older adults, Journal of clinical endocrinology and metabolism 100(2) (2015) 670‐678. | Excluded | Title and abstract | Irrelevant |
| 1352] K.L.J. Cederberg, R. Silvestri, A.S. Walters, Vitamin D and Restless Legs Syndrome: A Review of Current Literature, Tremor Other Hyperkinet Mov (N Y) 13 (2023) 12. | Excluded | Title and abstract | Irrelevant |
| 1353] B. Al-Shawwa, Z. Ehsan, D.G. Ingram, Vitamin D and sleep in children, Journal of Clinical Sleep Medicine 16(7) (2020) 1119-1123. | Excluded | Title and abstract | Irrelevant |
| 1354] F. Romano, G. Muscogiuri, E. Di Benedetto, V.V. Zhukouskaya, L. Barrea, S. Savastano, A. Colao, C. Di Somma, Vitamin D and Sleep Regulation: Is there a Role for Vitamin D?, Current pharmaceutical design 26(21) (2020) 2492-2496. | Excluded | Title and abstract | Irrelevant |
| 1355] K.A. Lee, A. Gomez, R.S. Zak, Vitamin D deficiency and restless legs syndrome during pregnancy: walking in sunshine?, Journal of clinical sleep medicine : JCSM : official publication of the American Academy of Sleep Medicine 19(1) (2023) 3-4. | Excluded | Title and abstract | Duplicatioin |
| 1356] K.A. Lee, A. Gomez, R.S. Zak, Vitamin D deficiency and restless legs syndrome during pregnancy: walking in sunshine? Commentary on Miyazaki A, Takahashi M, Shuo T, Eto H, Kondo H. Determination of optimal 25-hydroxyvitamin D cutoff values for the evaluation of restless legs syndrome among pregnant women. <i>J Clin Sleep Med</i>. 2023;19(1):73-83. doi: 10.5664/jcsm.10270, Journal of Clinical Sleep Medicine 19(1) (2023) 3-4. | Excluded | Full text | No relevant outcome |
| 1357] A.B. Krayem, S.O. Wali, A. Shukr, A. Boudal, A. Alsaiari, Vitamin D deficiency is associated with restless legs syndrome, Sleep 36 (2013) A242. | Excluded | Full text | Without control |
| 1358] G. Ganguly, Vitamin D deficiency, excessive daytime sleepiness: An epiphenomenon or a "chicken or an egg - Which came first" issue?, Journal of Clinical Sleep Medicine 9(5) (2013) 517-518. | Excluded | Title and abstract | Irrelevant |
| 1359] Nct, VItamin D Effect on Osteoarthritis Study, https://clinicaltrials.gov/show/NCT01176344 (2010). | Excluded | Title and abstract | Irrelevant |
| 1360] Nct, Vitamin D for Painful Nocturnal Leg Cramps, https://clinicaltrials.gov/show/NCT00715429 (2008). | Excluded | Title and abstract | Irrelevant |
| 1361] A. Stefani, T. Mitterling, G. Weiss, B. Högl, Vitamin D in a large sample of patients with restless legs syndrome: A casecontrol study, European journal of neurology 24 (2017) 77. | Excluded | Title and abstract | Duplication |
| 1362] A. Stefani, T. Mitterling, G. Weiss, B. Hoegl, Vitamin D in a large sample of patients with restless legs syndrome: A case-control study, European journal of neurology 24 (2017) 77-77. | Included | Full text | Relationship between vitamins and RLS |
| 1363] Nct, Vitamin D in the Treatment of Primary Restless Legs Syndrome, https://clinicaltrials.gov/show/NCT02256215 (2014). | Excluded | Full text | Duplication |
| 1364] T.P. Lam, B.K.W. Ng, L.W.H. Cheung, K.M. Lee, L. Qin, K.L. Liu, Q.W.Y. Mak, F.W.P. Yu, J.C.Y. Cheng, Vitamin D level can affect the therapeutic effects of whole-body vibration (WBV) for osteopenia in girls with adolescent idiopathic scoliosis (AIS), Osteoporosis international 24 (2013) S595‐S596. | Excluded | Title and abstract | Irrelevant |
| 1365] T.L. Järvinen, T.A. Järvinen, H. Sievänen, A. Heinonen, M. Tanner, X.H. Huang, A. Nenonen, J.J. Isola, M. Järvinen, P. Kannus, Vitamin D receptor alleles and bone's response to physical activity, Calcified tissue international 62(5) (1998) 413‐417. | Excluded | Title and abstract | Irrelevant |
| 1366] J. Valtuena, L. Gracia-Marco, G. Vicente-Rodriguez, M. Gonzalez-Gross, I. Huybrechts, J.P. Rey-Lopez, T. Mouratidou, I. Sioen, M.I. Mesana, A.E. Diaz Martinez, K. Widhalm, L.A. Moreno, H.S. Grp, Vitamin D status and physical activity interact to improve bone mass in adolescents. The HELENA Study, Osteoporosis International 23(8) (2012) 2227-2237. | Excluded | Title and abstract | Irrelevant |
| 1367] M. Abboud, Vitamin D Supplementation and Sleep: A Systematic Review and Meta-Analysis of Intervention Studies, Nutrients 14(5) (2022). | Excluded | Title and abstract | Irrelevant |
| 1368] Nct, Vitamin D Supplementation in Estonian Conscripts, https://clinicaltrials.gov/show/NCT04939636 (2021). | Excluded | Title and abstract | Irrelevant |
| 1369] Y. Cao, G. Jones, F. Cicuttini, T. Winzenberg, A. Wluka, J. Sharman, K. Nguo, C. Ding, Vitamin D supplementation in the management of knee osteoarthritis: study protocol for a randomized controlled trial, Trials 13 (2012) 131. | Excluded | Title and abstract | Irrelevant |
| 1370] L. Buratti, M.P. Luconi, G. Viticchi, L. Provinciali, M. Silvestrini, Vitamin D supplementation: a useful strategy for restless legs syndrome exacerbation in a patient with Turner syndrome, Neurological sciences : official journal of the Italian Neurological Society and of the Italian Society of Clinical Neurophysiology 38(6) (2017) 1135-1136. | Excluded | Full text | No relevant outcome |
| 1371] G.P. Novelli, C. Adembri, E. Gandini, S.Z. Orlandini, L. Papucci, L. Formigli, L.I. Manneschi, A. Quattrone, C. Pratesi, S. Capaccioli, Vitamin E protects human skeletal muscle from damage during surgical ischemia-reperfusion, American journal of surgery 173(3) (1997) 206‐209. | Excluded | Title and abstract | Irrelevant |
| 1372] Nct, Vitamin K2 Effect on Vascular Stiffening in Subjects With a Poor Vitamin K-status, https://clinicaltrials.gov/show/NCT02404519 (2015). | Excluded | Title and abstract | Irrelevant |
| 1373] Nct, Vitamin K2 Supplements for Muscle Recovery, https://clinicaltrials.gov/show/NCT05161975 (2021). | Excluded | Title and abstract | Irrelevant |
| 1374] C.F. Lippa, Vitamins and Alzheimer's disease: An easy (antioxidant) approach to intervention, American Journal of Alzheimer's Disease and other Dementias 28(2) (2013) 106-107. | Excluded | Title and abstract | Irrelevant |
| 1375] Nct, VLCD & Adjuvant Exercise Effect in Overweight Diabetic Men, https://clinicaltrials.gov/show/NCT04957589 (2021). | Excluded | Title and abstract | Irrelevant |
| 1376] R.A. Harrison, T. Vu, A.J. Hunter, Wernicke's encephalopathy in a patient with schizophrenia, Journal of general internal medicine 21(12) (2006) 1338-1338. | Excluded | Title and abstract | Irrelevant |
| 1377] I. Erol, F. Alehan, A. Gumus, West syndrome in an infant with vitamin B<sub>12</sub> deficiency in the absence of macrocytic anaemia, Developmental medicine and child neurology 49(10) (2007) 774-776. | Excluded | Title and abstract | Irrelevant |
| 1378] A.S. Walters, S. Patton, J. Connor, K. Bagai, A. Anderson, A. Bowman, M. Aschner, What about the other body metals in RLS?: Preliminary evidence for an increase in Zinc in Restless Legs Syndrome, Sleep 39 (2016) A229. | Excluded | Title and abstract | Irrelevant |
| 1379] D.W. Lewis, What was wrong with Tiny Tim?, American journal of diseases of children (1960) 146(12) (1992) 1403-7. | Excluded | Title and abstract | Irrelevant |
| 1380] D.P. Murphy, B. Graef, Which came first? Restless legs syndrome or insomnia?, Consultant 46(7) (2006) 761-764. | Excluded | Title and abstract | Irrelevant |
| 1381] Actrn, Whole-Body Vibration Treatment in Breast Cancer Survivors on Aromatase Inhibitor Therapy, http://www.who.int/trialsearch/Trial2.aspx?TrialID=ACTRN12611001094965 (2011). | Excluded | Title and abstract | Irrelevant |
| 1382] Actrn, Whole-Body Vibration Treatment in Prostate Cancer Survivors on Androgen Suppression Therapy, http://www.who.int/trialsearch/Trial2.aspx?TrialID=ACTRN12612000082808 (2012). | Excluded | Title and abstract | Irrelevant |
| 1383] B.J. Goldlist, Why blood remains so special, Geriatrics and Aging 6(4) (2003) 9. | Excluded | Title and abstract | Irrelevant |
| 1384] P.H. Heinermann, Yellow intraocular filters in fishes, Experimental biology 43(2) (1984) 127-47. | Excluded | Title and abstract | Irrelevant |
| 1385] S.S. Morkous, O.H. Lewis, D. Auckley, A young man who rocks and rolls at night, Annals of the American Thoracic Society 12(9) (2015) 1413-1415. | Excluded | Title and abstract | Irrelevant |
| 1386] S. Meneghini, D. Modena, G. Colombo, A. Coatti, N. Milani, L. Madaschi, A. Amadeo, A. Becchetti, The β2<SUP>V287L</SUP> nicotinic subunit linked to sleep-related epilepsy differently affects fast-spiking and regular spiking somatostatin-expressing neurons in murine prefrontal cortex, Progress in neurobiology 214 (2022). | Excluded | Title and abstract | Irrelevant |
| 1387] ا. محمدعلی, ا. ذبیحاله, م. سیامک, ش. غلامرضا, ر. احمد, تأثیر آموزش براساس تئوری انگیزش محافظت بر رفتارهای پیشگیریکننده از ابتلا به در دانشآموزان دبیرستانی شهر قم ،A آنفلوانزای نوع, Qom university of medical sciences journal 13(4) (2019) 22‐33. | Excluded | Title and abstract | Irrelevant |
| 1388] Suzuki S, Suzuki K, Miyamoto M, Miyamoto T, Watanabe Y, Takashima R, et al. Evaluation of contributing factors to restless legs syndrome in migraine patients. Journal of neurology 2011;258:2026-35. | Included | Full text | Relationship between vitamins and RLS |
| 1389] Högl B, Kiechl S, Willeit J, Saletu M, Frauscher B, Seppi K, et al. Restless legs syndrome -: A community-based study of prevalence, severity, and risk factors. Neurology 2005;64:1920-4. | Included | Full text | Relationship between vitamins and RLS |
| 1390] Yoshimura C, Arima H, Amagase H, Takewaka M, Nakashima K, Imaoka C, et al. Idiopathic and secondary restless legs syndrome during pregnancy in Japan: Prevalence, clinical features and delivery-related outcomes. PloS one 2021;16:e0251298. | Excluded | Full text | No specific data |
| 1391] Dadashpour S, Hajmiri MS, Roshani D. Effect of intravenous vitamin C supplementation on the quality of sleep, itching and restless leg syndrome in patients undergoing hemodialysis; A double-blind randomized clinical trial. J Nephropharmacol. 2018;7(2):131-136. | Excluded | Full text | No specific data |
